# Recent Advances in the Design of Inhibitors Targeting the Viral Entry and Replication of the SARS-CoV-2 Virus, Driven by In Silico Approaches

**DOI:** 10.3390/molecules31162877

**Published:** 2026-08-18

**Authors:** Phumelele Nodola, Patience S. S. Molefe, Potlaki F. Tseki, Njabulo J. Gumede

**Affiliations:** Department of Chemical and Physical Sciences, Faculty of Natural Sciences, iYunivesithi Walter Sisulu (iWS), Private Bag X01, Mthatha 5099, Eastern Cape, South Africa; pnodola@wsu.ac.za (P.N.); patiencemolefe0@gmail.com (P.S.S.M.); potlakitseki@gmail.com (P.F.T.)

**Keywords:** SARS-CoV-2, in silico, viral entry, viral replication, mutations, long-COVID

## Abstract

The SARS-CoV-2 pandemic has significantly impacted global health, politics, medicine, finance, and society. Since 2020, various mutations have been reported, leading to drug resistance in current treatments against different SARS-CoV-2 strains and a drastic increase in cases of long-COVID. This situation underscores the urgent need to develop targeted and effective drugs to combat the spread of SARS-CoV-2 strains and their mutants, manage long-COVID symptoms and prepare for future pandemics. Currently, the treatment of SARS-CoV-2 focuses on targeting the virus’s entry and replication mechanisms to disrupt its life cycle. This review examines approved drugs, clinical candidates, and inhibitors under development, along with their bioassay data, while highlighting associated challenges. It illustrates how inhibitors bind to active sites, providing insights and emphasizing the importance of in silico studies, such as molecular docking, molecular dynamics simulation, FEP+, WaterMap, and quantitative structure–activity relationship (QSAR) analyses, and their correlation with experimental studies in expediting the drug discovery process. The review aims to provide researchers with insights into the gaps that need to be addressed concerning mutations affecting viral entry and to prepare for future pandemics.

## 1. Introduction

The COVID-19 pandemic, caused by SARS-CoV-2, is the most recent global health crisis, marking the seventh human coronavirus [1]. SARS-CoV-2 is the most contagious, with approximately 772.4 million confirmed cases and around 7 million deaths as of December 2023 [1]. Initially identified in Wuhan, China, it rapidly spread globally [2]. Trans- mission occurs through contact with infected individuals via respiratory droplets [3]. The virus enters host cells by binding its spike protein to the ACE2 receptor, with TMPRSS2 facilitating viral entry [4,5]. ACE2 is expressed in various organs, making them susceptible to infection [6,7]. The virus affects all age groups, with a pronounced impact on older adults and individuals with comorbidities [8,9,10].

Adaptive immune responses mediated by T and B cells are critical in defending against the virus during the initial 7–10 days post-infection [7]. COVID-19 is further associated with long-term neurological consequences, exacerbated by the body’s immune system releasing excessive pro-inflammatory cytokines, known as a “cytokine storm”, in an attempt to mitigate neuroinflammation caused by the virus [10]. The persistence of SARS-CoV-2 symptoms, termed “long-COVID”, beyond the acute phase is linked with heart failure, obesity, hypertension, chronic kidney diseases, cardio-metabolic disorders, and atherosclerotic cardiovascular diseases [11]. SARS-CoV-2 was initially designated as a public health emergency of international concern (PHEIC) in January 2020, prompting global mitigation strategies such as lockdowns and social distancing, alongside accelerated therapeutic development [12,13,14,15]. Concurrently, the World Health Organization initiated the “Solidarity” trial, a comprehensive international effort designed to ascertain efficacious treatments for SARS-CoV-2, ultimately resulting in the granting of emergency use authorization (EUA) for several pre-existing antiviral therapies [16]. Computer-aided drug discovery has emerged as one of the most reliable approaches, given the urgent need for novel SARS-CoV-2 treatments. It concurrently reduces the probability of producing toxic drugs and facilitates optimization before experimental validation [17,18]. However, despite significant progress, no comprehensive therapeutic strategy fully addresses emerging variants and long-term complications. Current efforts increasingly focus on viral entry and replication targets. Notably, many antiviral agents incorporate heterocyclic scaffolds, which enhance pharmacokinetic and pharmacodynamic properties and support broad applications in drug development [13,18,19,20].

Unlike previous reviews that primarily provide broad overviews of SARS-CoV-2 biology [20,21,22,23] or summarize therapeutic strategies and clinical outcomes [23,24], as well as those focusing on individual computational techniques or specific targets such as ACE-2 [24], Mpro [25,26], and PLpro [27,28], the present review provides an integrated and critical perspective by systematically evaluating computational approaches used in SARS-CoV-2 drug discovery, including repurposing and de novo design strategies. It analyzes molecular interactions with key viral and host targets involved in infection and replication, while highlighting the strengths and limitations of these methods. Importantly, it emphasizes multi-target strategies such as the Cathepsin L and Mpro dual inhibition to achieve synergistic antiviral effects, offering both methodological insights and a forward-looking framework that goes beyond the predominantly descriptive nature of existing studies.

## 2. Threats Due to Mutations of Drug Targets Involved in SARS-CoV-2’s Viral Entry and Replication and Long COVID, an Unmet Health Challenge

SARS-CoV-2 has undergone numerous mutations since 2019, leading to variants that complicate global containment efforts. These variants are categorized based on their impact on transmission, disease severity, and intervention effectiveness as variants of concern (VOC), variants of interest, variants being monitored (VBM), and variants of high consequence (VOHC). Variants of concern show increased transmissibility, disease severity, and reduced efficacy of public health interventions [29]. The Omicron variants remain the most mutated strain, with 50 mutations across the viral genome and 26–32 mutations on the spike protein [30]. These mutations occur on the receptor-binding domain (RBD) of the spike protein, including N501Y, E484K, L452R, T478K, and E484Q [31]. The regulation of viral proliferation has been challenging as new variants develop resistance to existing treatments, affecting the effectiveness of potent drugs. The following mutants—S144M/F/A/G/Y, H172Q/F, M165T, E166V/G/A, and Q192T/S/L/A/I/P/H/V/W/C/F—have been identified on the Mpro’s binding site, interrupting the effectiveness of the most reliable SARS-CoV-2 inhibitor so far, “Nirmatrelvir” [32]. PLpro is less susceptible to mutations, with only three points of mutation identified and accounting for >20% of SARS-CoV-2 PLpro’s variant of concern A145D (alpha), K92N (beta), and K232Q (gamma) on the thumb region [28,33,34]. Five more mutations (P247S, Y264H, E263D, T265A, and Y268C) have been identified near the substrate-binding site. These mutations should be carefully considered in future PLpro-targeted drug design to ensure broad-spectrum efficacy and resistance resilience [35].

While most individuals recover from acute COVID-19, 31–69% experience long-term health issues called “long-COVID” [36,37]. The acute phase spans five weeks, with viral load peaking then declining [5,38]. SARS-CoV-2 disrupts host cells, causing impairments and cell death. The immune response triggers systemic hyperinflammation, leading to thrombosis and organ failure [5]. Long-COVID symptoms include fatigue, myalgia, cough, chest discomfort, and breathing difficulties, which may fluctuate [5,36,37]. After three weeks, the viral load becomes undetectable through RT-PCR. Untreated symptoms can permanently damage organs [5]. Complications include heart attacks, cognitive impairment, and organ dysfunction [5,39]. Healthcare centers manage long-COVID through symptom-based approaches [5]. While identifying drugs for SARS-CoV-2, addressing long-COVID mechanisms requires equal attention to viral mutations.

### 2.1. Small-Molecule Therapeutic Drugs Approved for the Treatment of SARS-CoV-2

**Figure 1:**
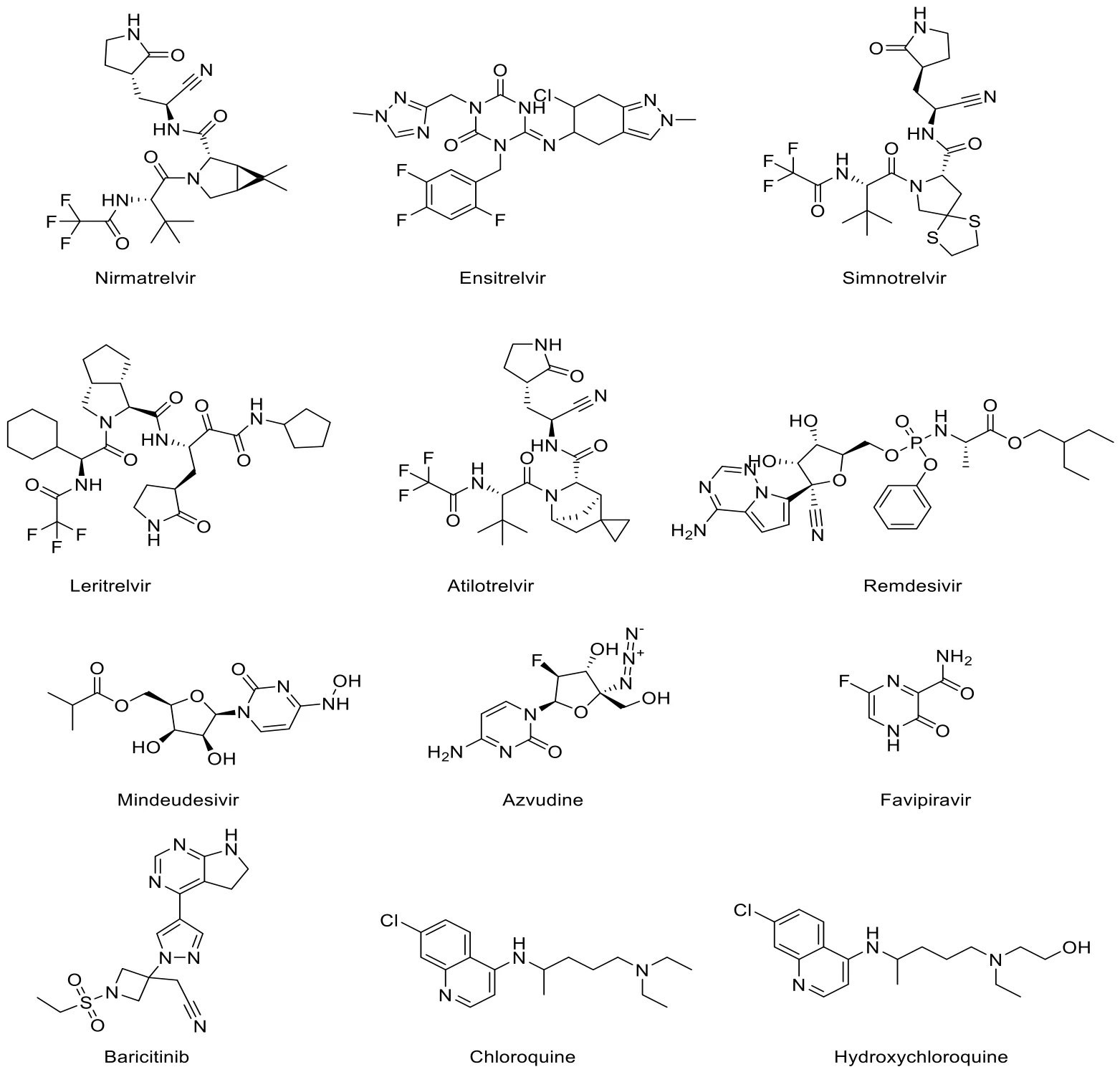
Approved and repurposed small molecules for SARS-CoV-2 and their molecular targets: Mpro inhibitors—Nirmatrelvir [49,50,51], Ensitrelvir [47,52,53], Simnotrelvir [54,55], Leritrelvir [47], Atilotrelvir [54]; RdRp—Remdesivir [55], Mindeudesivir [30], Azvudine [47], Favipiravir [56]; endosomal entry—baricitinib [57], Chloroquine [58], and Hydroxychloroquine [59].

Since 2019, researchers have worked to develop or repurpose drugs for SARS-CoV-2 treatments, leading to several drug approvals. Remdesivir, Hydroxychloroquine, and Ivermectin were repurposed from treating Hepatitis C, malaria, and infestations to manage SARS-CoV-2 [40,41]. However, the most recent finding is that although Chloroquine/Hydroxychloroquine (CQ/HCQ) and Ivermectin remain debatable as they may exhibit mechanistic activity against SARS-CoV-2, most phase III clinical trials have failed to demonstrate clinical benefit in COVID-19 patients, and overall evidence from these trials does not support their efficacy for treatment [42]. While Remdesivir proved effective, its intravenous administration requires hospital settings, limiting accessibility; oral formulations are undergoing development [43,44]. The Remdesivir drug affects the respiratory, gastrointestinal, cardiovascular, and reproductive systems [44]. New anti-SARS-CoV-2 drugs include Paxlovid, Xocova, and Molnupiravir [45,46]. China approved four domestic oral drugs: Azvudine, Leritrelvir, Simnotrelvir–Ritonavir, and Mindeudesivir [47] (Table 1, Figure 1). Most therapeutics now show resistance to new SARS-CoV-2 mutants, with efficacy ranging from 88% to below 21% [48]. Drug repurposing, the process of identifying new therapeutic uses for existing drugs, remains the most efficient strategy for the rapid and cost-effective development of broad-spectrum antivirals.

Remdesivir (see Figure 1) is an intravenously administered nucleoside analog used to inhibit the action of RNA polymerase in the treatment of COVID-19 for non-hospitalized patients with mild-to-moderate symptoms [55]. Its emergency use approval was first awarded in May 2020, and later that year, it was given full approval for the treatment of pediatric patients less than 12 years old [60]. Remdesivir is a prodrug metabolized within the host cells to the therapeutically active triphosphate metabolite that has a reasonable biochemical activity of IC_50_ = 770 nM and antiviral activity of EC_50_ = 6.2 µM as a delayed chain terminator blocking viral protein transcription [60,61]. Despite its initial promise, the clinical efficacy of Remdesivir remains debated, and multiple case reports have documented emerging resistance to Remdesivir, particularly in patients with underlying conditions undergoing prolonged treatment [44,62,63,64]. This is supported by Gandhi et al., who reported the de novo emergence of the nsp12 E802D mutation during remdesivir therapy in an immunocompromised patient with persistent COVID-19, resulting in reduced antiviral susceptibility and viral recrudescence [64]. In addition, Luo et al. demonstrated that other mutations, such as V557L, may have contributed to remdesivir resistance through structural mechanisms that affect RdRp function [65]. Baricitinib was the first Food and Drug Administration (FDA)-approved immunomodulatory treatment for COVID-19. On the 19th of November 2020, the FDA issued an Emergency Use Authorization (EUA) for baricitinib to be used in combination with Remdesivir; this decision was reviewed on 28 July 2021, and baricitinib was authorized to be used as a stand-alone treatment [66]. The in silico metabolism prediction of baricitinib suggested that structural alterations to the pyrimidine ring and the pyrrole ring may improve metabolic stability and safety in drug design [67].

Nirmatrelvir is an orally administered 3C-like protease (3CLpro/Mpro) inhibitor prescribed and co-packed with ritonavir to patients suffering from moderate-to-mild COVID-19. Its EUA was originally granted in 2020 by the US FDA before receiving full approval in 2023 for the treatment of adults at high risk of progression to severe COVID-19 symptoms [68]. It was manufactured as Paxlovid by Pfizer and distributed as part of the U.S. Department of Health and Human Services’ response to the COVID-19 pandemic. Nirmatrelvir was selected as an inhibitor due to its potent biochemical activity (K_i_ = 3.11 nM) and Vero E6 antiviral activity EC_50_ = 74.5 nM, achieved by its ability to form a reversible covalent thioimidate adduct with Mpro’s Cys145, and stabilizing a single binding mode with its target [69]. Co-administration of nirmatrelvir with subtherapeutic doses of ritonavir extends its clinical efficacy as ritonavir is an inhibitor of the major human hepatic drug-metabolizing enzyme cytochrome P450 3A4 (CYP3A4), responsible for the metabolism of the majority of drugs [70,71]. The inhibition of CYP3A4 boosts the therapeutic concentration of Ritonavir, with an intrinsic clearance value of 25 to 32.8 µL/min/mg [49,51]. Nirmatrelvir maintained a good potency even in the FRET assay and Cell-Based Antiviral Assay (ALI-HBEC), achieving EC_50_ values of 3.0 nM and 25.3 nM, respectively [50,51]. Nirmatrelvir is the most effective anti-SARS-CoV-2 approved drug, with an EC_50_ range between 3.0 ± 0.1 nM and 25.3 nM, followed by Molnupiravir, Hydroxychloroquine, and Remdesivir: with EC_50_ values of 0.4 µM, 0.72 µM, and 0.77 µM, respectively, as shown in Table 1. Despite its proven efficacy in reducing COVID-19 severity, Paxlovid (Nirmatrelvir/Ritonavir) faces several clinical challenges, most notably, drug–drug interactions in patients, primarily due to its co-administration with Ritonavir. Consequently, when it interacts with drugs that also inhibit CYP450 enzymes, it can lead to elevated levels of co-administered drugs, increasing the risk of toxicity or adverse effects, especially in patients taking anticoagulants, immunosuppressants, or certain cardiovascular drugs [72]. Notably, the highest percentage of Mpro inhibitors are covalent inhibitors that bind like Nirmatrelvir and require co-administration to improve their physicochemical properties.

Ensitrelvir, a non-peptidomimetic inhibitor with an IC_50_ of 0.013 µM, comparable to that of Nirmatrelvir, and moderate potency of 0.37 µM in the Vero cell line, has exhibited improved metabolic stability when compared to Nirmatrelvir [73]. As such, Ensitrelvir is currently approved by the Pharmaceuticals and Medical Devices Agency (PMDA), with an intrinsic clearance of 1.7 mL/min/Kg in rats and a good antiviral activity EC_50_ of 0.37 µM in a cell-based assay (VeroE6/TMPRSS2) [47,52,53]. However, these results were surpassed by Leritrelvir and Atilotrelvir, with both exhibiting an antiviral activity in the nanomolar range using the same assay [47,54]. Simnotrelvir also showed a good potency in the FRET assay with an EC_50_ value of 79 nM, comparable to that of Nirmatrelvir [62,63]. Notably, drugs targeting Mpro were more potent than other candidates since their inhibitory activity values were in the nanomolar range, and all covalent Mpro drug candidates have a P4-trifluoroacetyl moiety, facilitating hydrophobic interactions [47,51,52,53,54].

Chloroquine (CQ) and Hydroxychloroquine (HCQ) are two SARS-CoV-2 endosomal entry inhibitors with antiviral activity of 5.47 µM and 0.72 µM in the Vero cell line [59]. The potency of Hydroxychloroquine was improved by the introduction of a hydroxyl group on Chloroquine. This also improved the lipophilicity from a log P value of 4.37 to 3.8. It should be noted that, although LogP is commonly used to assess lipophilicity, oral bioavailability is influenced by multiple physicochemical parameters, including molecular weight, hydrogen bond donors and acceptors, and polar surface area, as described by Lipinski’s Rule of Five and Veber’s criteria [74]. According to the Lipinski Rule of Five, log P should be <5 for oral administration; however, the most acceptable range is 1.35–1.8 for intestinal and oral absorption, and for the central nervous system (CNS), it should be around 2 [75]. Some reports suggest that HCQ’s efficacy may be enhanced when combined with Azithromycin. However, other studies have raised concerns about the long-term and combined use of CQ or HCQ with other drugs, particularly Azithromycin, due to serious cardiac side effects. These include QT interval prolongation, arrhythmogenic risks, QRS widening, diabetes, etc., which can lead to potentially life-threatening heart complications [76,77]. The FDA revoked the emergency use authorization for Chloroquine and Hydroxychloroquine after determining that they are unlikely to be effective against COVID-19 and pose serious cardiac risks. This decision was based on updated scientific data and a request from the Biomedical Advanced Research and Development Authority (BARDA) [78]. The newer drug Molnupiravir received its full approval from the UK Medicines and Healthcare products Regulatory Agency (MHRA) in 2021 before it was given an EUA by the US FDA to treat adults above the age of 18 who tested positive for SARS-CoV-2 due to its impact on bone and cartilage growth [79,80].

Molnupiravir is also restricted for pregnant individuals because of its potential to cause serious fetal abnormalities and increase the risk of miscarriage [81]. Nonetheless, the most clinically relevant drugs for SARS-CoV-2 includes Remdesivir [23], Molnupiravir, and Nirmatrelvir/Ritonavir (Paxlovid), which have demonstrated therapeutic benefit; however, their effectiveness is limited by factors such as modest clinical efficacy in some patient groups, the emergence of viral resistance and variants, adverse side effects, significant drug–drug interactions, and limited accessibility in certain regions [82].

**Table 1:** Approved small molecules for SARS-CoV-2 and their pharmacodynamics and physicochemical attributes.

Name	Target	IC50	Ki	EC50	Assay	Metabolic Profiling (CLint)	Metabolite ID	Physicochemical Properties	Approval Status	Design Strategy	Reference
						(µL/min/mg)		LogD			
Nirmatrelvir	Mpro	ND*	3.11 nM	ND*	Inhibition of proteolytic activity of SARS-CoV-2 (FRET assay)	25	CYP3A4	1.9	FDA approved	Retention of pep-tidomimetic chemotype	[49,69]
	Mpro	ND*	ND*	3.0 ± 0.1 nM	Fluorescence assay and FRET-based assay	ND*	CYP3A4	ND	FDA approved	Retention of pep-tidomimetic chemotype	[50]
	Mpro	28.1 nM	ND*	25.3 nM	Cell-based antiviral assay (ALI-HBEC)	32.8	CYP3A4	1.5	FDA approved	Retention of pep-tidomimetic chemotype	[51]
Ensitrelvir	Mpro	0.013 µM	ND*	0.37 µM	Cell-based assay (VeroE6/TMPRSS2)	1.7 mL/min/Kg (rats)	ND*	ND*	PMDA approved	Non-peptidomimetic inhibitor	[47,52,53]
Simnotrelvir	Mpro	9 ± 1 nM	ND*	34 ± 9 nM	Cell-based assay (VeroE6)	ND*	ND*	ND*	NMPA approved	Retention of pep-tidomimetic chemotype	[83,84]
Leritrelvir	Mpro	ND*	8.4 ± 0.2 nM	95 nM	Cell-based assay (VeroE6)	ND*	CYP3A4	ND*	NMPA approved	Retention of pep-tidomimetic chemotype	[47]
Atilotrelvir	Mpro	3.0 nM	ND*	79 nM	Enzymatic activity, cell-based assay (Vero E6 cell)	ND*	CYP3A4	ND*	NMPA approved	Retention of pep-tidomimetic chemotype	[54]
Remdesivir	RdRp	ND*	ND*	0.77 μM	Cell-based assay (VeroE6)	ND*	ND*	ND*	FDA approved	Nucleoside analog	[55]
Molnupinavir	RdRp	0.3 µM	ND*	0.4 µM	Cell-based assay (Vero E6)	ND*	ND*	ND*	Approved in UK, EU in many countries	Non-peptidomimetic inhibitor	[30]
Mindeudesivir	RdRp	0.67 ± 0.24 μM	ND*	0.35 ± 0.09	Cell-based assay (A549)	ND*	116-NTP	ND*	NMPA approved	Non-peptidomimetic inhibitor	[47]
Azvudine	RdRp	ND*	ND*	4.31 µM	Cell-based assay (Vero E6)	ND*	CYP3A4	ND*	NMPA approved	Nucleoside reverse transcriptase inhibitor	[56]
Favipiravir	RdRp	ND*	ND*	61.88 μM	Cell-based assay (Vero E6)	ND*	T705M1, CYPs	ND*	Approved in many countries	Nucleoside analog	[57]
Baricitinib	Endosomal entry	5.9 nM	ND*	ND*	Cell-based and enzymatic assays	ND*	ND*	ND*	EUA by FDA	Non-peptidomimetic inhibitor	[58]
Chloroquine	Endosomal entry	ND*	ND*	5.47 μM	Cell-based assay (Vero E6)	ND*	CYP3A5, CYP2D6,	ND*	EUA by FDA, retracted	Non-peptidomimetic inhibitor	[69]
Hydroxychloroquine	Endosomal entry	ND*	ND*	0.72 μM	Cell-based assay (Vero E6)	ND*	CYP3A5, CYP2D6, CYP1A1	ND*	EUA by FDA, retracted	Non-peptidomimetic inhibi-	[47]

ND*: not determined, FDA: Food and Drug Administration, EUA: Emergency Use Authorization, NMPA: National Medical Products Administration, PMDA: Pharmaceuticals and Medical Devices Agency.

**Figure 2:**
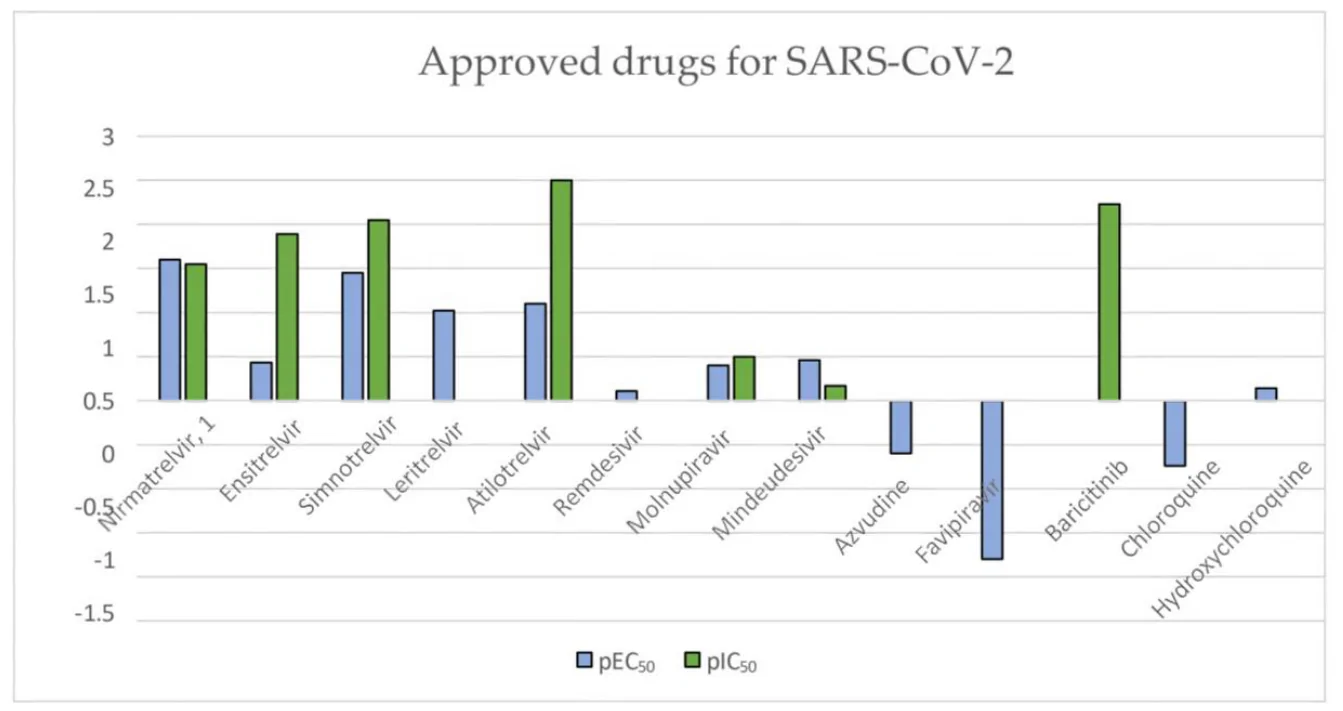
Approved small molecules targeting SARS-CoV-2 and their respective pIC_50_ and pEC_50_ values.

There is a reasonably good correlation between pEC_50_ and pIC_50_ values for the approved drugs, as shown in Figure 2; however, Mindeudesivir had negative values, showing poor efficacy. Nirmatrelvir, Ensitrelvir, Simnotrelvir, Atilotrelvir, and Molnupiravir had positive pIC_50_ and pEC_50_ values, with Atilotrelvir having the highest inhibitory activity amongst the rest (Table 1). Metabolic stability, log P, and logD are underreported specifically for SARS-CoV-2 since most of these drugs were repurposed. As shown in Table 1, only Nirmatrelvir had data for logD within the range of 1.5–1.9 and an intrinsic clearance of 25–32.8 µL/min/mg in human upon co-administration with Ritonavir, indicating good solubility with moderate clearance [49,51]. Azvudine is administered orally and showed better activity compared to Remdesivir, with minor side effects from the phase III clinical trial, and they both target RdRp. Favipiravir exhibited the lowest pEC_50,_ whereas Monupiravir had the highest pEC_50_ in the same cell-based assay (Vero E6). However, Mindeudesivir had the highest pEC_50_ values, although it was conducted in an A549 cell-based assay. RdRp inhibitors mentioned above played a crucial role in reducing SARS-CoV-2 viral replication and mitigating the severity of COVID-19 symptoms [71,85]. However, SARS-CoV-2 is an RNA virus characterized by a high mutation rate due to the low fidelity of its RNA-dependent RNA polymerase [86]. This frequent genetic variability poses a significant challenge, as mutations may alter the structure of viral proteins targeted by these drugs, potentially reducing their effectiveness and leading to antiviral resistance. Mindeudesiver (RdRp) and Hydroxychloroquine (endosomal entry) exhibited good potency in the nanomolar range. Despite its potency, Hydroxychloroquine and Chloroquine were withdrawn for the treatment of SARS-CoV-2 due to the side effects mentioned above.

### 2.2. Viral Entry Targets for SARS-CoV-2

**Figure 3:**
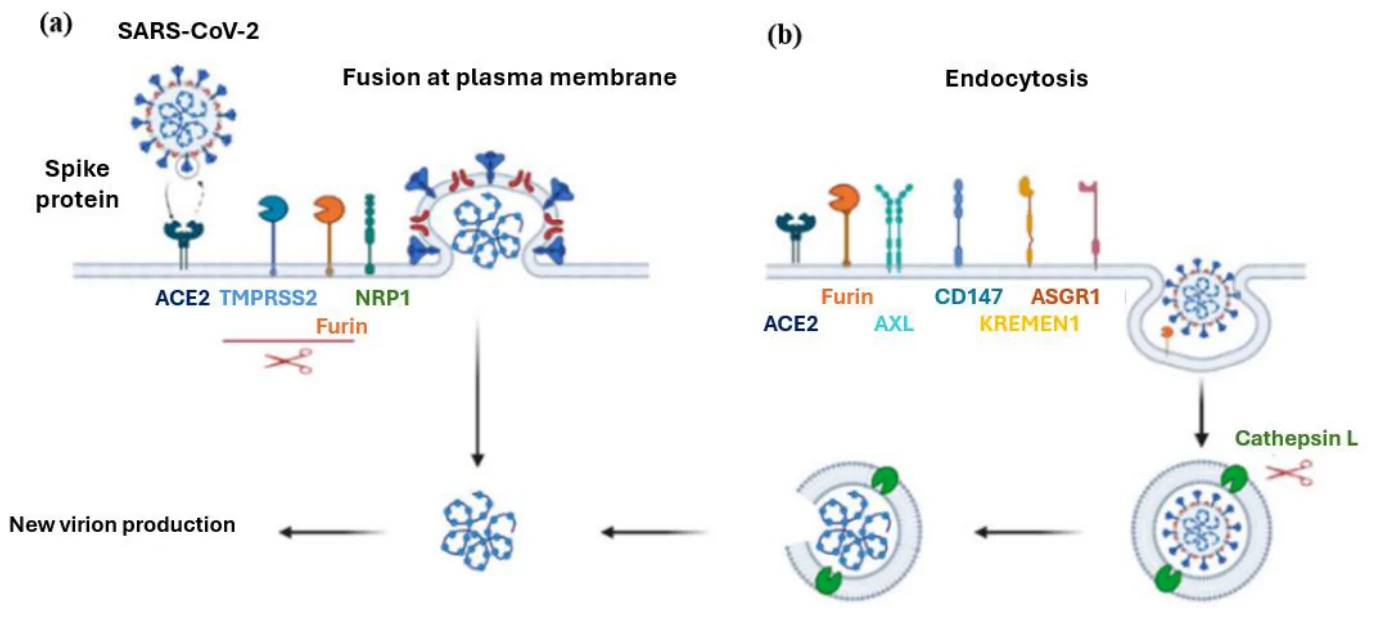
SARS-CoV-2 mechanism of entry pathways. (**a**) The SARS-CoV-2 spike protein interacts with ACE2 (blue), while TMPRSS2 (light blue) and NRP1 (green) facilitate viral attachment and membrane fusion. Furin (orange) cleaves the spike protein at the S1/S2 site, priming the virus for fusion and entry at the cell surface. (**b**) Endocytic entry pathway. Following binding to ACE2 (dark blue) and alternative receptors, including AXL (cyan), CD147 (light blue), ASGR1 (pink), and KREMEN1 (yellow), SARS-CoV-2 is internalized through endocytosis. Within endosomes, Cathepsin L (green) mediates proteolytic activation of the spike protein, enabling membrane fusion and viral genome release. Both entry routes ultimately lead to viral replication and production of new virions [90]. Reproduced with permission [89]. Copyright 2023 MDPI.

SARS-CoV-2 enters host cells in two distinct pathways, i.e., the cell surface pathway and the endosomal pathway, as shown in Figure 3. In the surface pathway, the viral spike (S) protein binds to the ACE2 receptor and is activated by the serine protease TMPRSS2, allowing direct fusion with the plasma membrane (Figure 3a) [4,5]. Alternatively, in the endosomal entry, the virus is internalized into endosomes, where Cathepsin L (CTSL), a lysosomal cysteine protease, cleaves and activates the spike protein, facilitating fusion with the endosomal membrane (Figure 3b) [87,88,89].

### 2.3. SARS-CoV2 Spike Protein ACE2: Structure and Function

**Figure 4:**
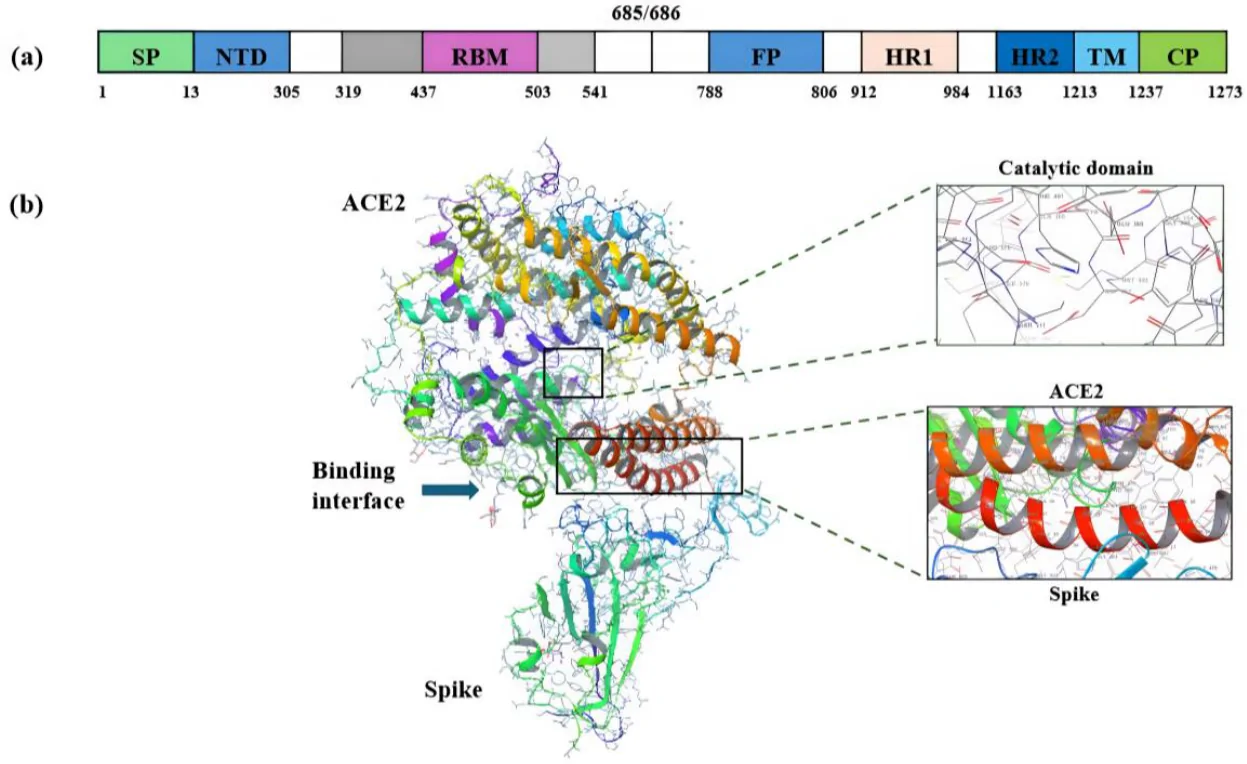
Structure of the SARS-CoV-2 S protein ACE2. (**a**) Domain organization of the SARS-CoV-2 spike protein (SP) showing the signal peptide (SP, green), N-terminal domain (NTD, blue), receptor-binding motif (RBM, magenta), fusion peptide (FP, blue), heptad repeats (HR1, peach and HR2, dark blue), transmembrane domain (TM, light blue), and cytoplasmic tail (CP, green), with the furin cleavage site at residues 685/686 (white). Reproduced with permission [91]. Copyright 2020 ACS. (**b**) ACE2 structure in a complex with the receptor-binding domain (RBD) of the SARS-CoV-2 spike protein (PDB ID: 6M0J). Reproduced with permission [95]. Copyright 2020 Springer Nature.

Spike protein is a glycoprotein made up of two subunits, i.e., the S1 subunit, which binds the host cell receptor, and the S2 subunit, responsible for the viral membrane fusion. The S1 subunit, which binds the host cell receptor, and the S2 subunit are responsible for the viral membrane fusion [91]. The S1 sub-unit contains the signal peptide (SP, 1–13 resi-dues), *N* terminal domain (NTD, 14–305 residues), and receptor binding domain (RBD, 319–541 residues) that bind to human angiotensin-converting enzyme-2 (hACE2); whereas the S2 subunit contains fusion peptide (FP, 788–806 residues), heptad repeats HR1 (912–984 residues) and HR2 (1163–1213 residues), trans-membrane domain (TM, 1213–1237 residues), and cytoplasmic domain (CP, 1237–1273 residues) as shown in Figure 4a [91]. The fusion peptide (FP) plays a crucial role in mediating membrane fusion by disrupting and connecting lipid bilayers of the host cell membrane [46]. SARS-CoV-2 initiates infection by binding to human angiotensin-converting enzyme-2 (hACE2) through the spike protein located at the SARS-CoV-2 virus’s envelope; see Figure 4b [91,92]. The hACE2 is a membrane-bound enzyme highly expressed in various cell types, including lungs, heart, liver, intestine, kidneys, and testis [30]. The spike protein has since been the main target of peptides, monoclonal antibodies, peptidomimetics, antibodies, and small molecules [46,93]. This resulted in a significant number of developed monoclonal antibodies against SARS-CoV-2.

The ACE2 receptor for SARS-CoV-2 targets mainly human organs, including the lungs, heart, liver, intestine, kidneys, and testis in the human body, and if the virus is not monitored, it can lead to cell damage [6]. These two are typical drug targets, especially the ACE2, as it attacks critical organs, resulting in long-COVID symptoms, which may result in permanent damage if untreated. Dysregulation of ACE2 after SARS-CoV-2 infection disrupts the angiotensin system, promoting persistent inflammation, endothelial dysfunction, and multi-organ symptoms [94].

### 2.4. Inhibitors Targeting the SARS-CoV-2 Spike Protein ACE2 Protein–Protein Interaction

**Figure 5:**
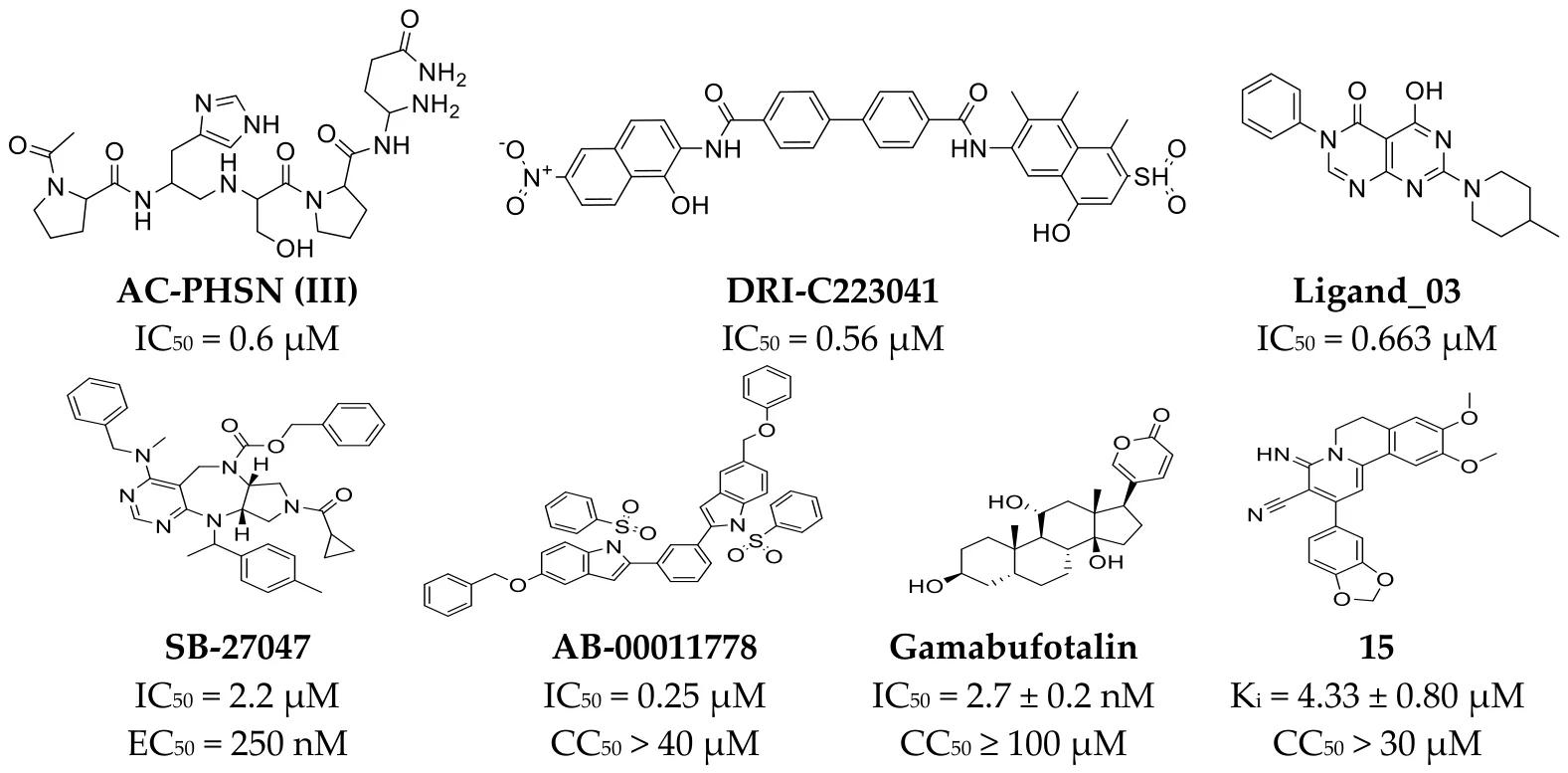
SARS-CoV-2 Spike protein ACE2 experimental inhibitors.

Several studies have used computational approaches to investigate inhibitors targeting the ACE2–SARS-CoV-2 interaction. Amin et al. demonstrated, using molecular modeling, that ACE2 binds more strongly to SARS-CoV-2 than to SARS-CoV, explaining its higher infectivity [96]. Munawar et al. optimized the ACE2 inhibitor ATN 161, yielding an IC_50_ of 3.16 µM, and developed a peptide analog, Ac-PHSPN-NH_2_ (Analog-III), which showed improved antiviral activity (IC_50_ = 0.6 µM), favorable cytotoxicity, and demonstrated the strongest binding affinities of −17.05 kcal/mol from molecular docking [97]. Bojadzic et al. identified potent spike-ACE2 inhibitors, with DRI C91005 exhibiting nanomolar activity (IC_50_ = 160 nM) [98]. Similarly, ELISA-based screening by Shin et al. identified small-molecule inhibitors of the ACE2–spike interaction, with SB27012 (cyclopropyl amide) showing an IC_50_ of 7.7 µM, while SB27016 (lacking R3 substituents) exhibited no activity, indicating structural dependence. Optimization of SB27012 produced SB27041, which demonstrated improved potency with IC_50_ values of 0.52–3.38 µM (spike–ACE2 binding) and a pseudovirus inhibition IC_50_ of 5.6 µM [99]. A CADD screening by Bastos et al. using the 6M0J crystal structure identified Ligand_003 with a docking score of −8.645 kcal/mol, outperforming Hydroxychloroquine (−7.595 kcal/mol). Subsequent optimization yielded Ligand_008, which showed improved binding affinity (−7.690 kcal/mol) and a predicted IC_50_ of 0.49 µM, compared to the experimental IC_50_ of 0.663 µM for Ligand_003. Lapaillerie et al. identified two SARS-CoV-2 entry inhibitors, AB 00011778 and AB 00047476, that blocked viral entry into human pulmonary cells, with IC_50_ values of 0.25–2.5 µM. However, despite good antiviral activity, AB 00011778 showed suboptimal cytotoxicity with a CC_50_ > 30 µM in A549 ACE2 TMPRSS2 cells [100]. Yang et al. reported a naturally occurring compound, Gamabufolatin (Figure 5), with the potential to inhibit SARS-CoV-2 ACE2, with an EC_50_ value in the nanomolar range; however, Gamabufolatin also showed poor cytotoxicity, with a CC_50_ value less than 40 µM [101]. Shahhamzehei et al. used molecular docking (PDB ID: 7BNN) to identify nitrile-containing isoquinoline derivatives 9, 14, and 15 as inhibitors of the spike RBD–ACE2 interaction. Among these, compound **15** showed promising activity with a K_i_ of 4.33 µM and CC_50_ > 100 µM, indicating good potency and favorable safety, and was proposed as a potential broad-spectrum coronavirus entry inhibitor [102].

Based on the three reported parameters of IC_50_, CC_50_, and K_i_ values, several compounds have shown strong potential as inhibitors of the ACE2–SARS-CoV-2 interaction. Among them, DRI-C91005 demonstrated the highest potency (IC_50_ = 160 nM), while Analog-III combined strong activity (IC_50_ = 0.6 µM) with low toxicity and high binding affinity, followed by compound **15** with a good K_i_ (4.33 µM) and excellent safety profile (CC_50_ > 100 µM). Compounds like SB27012, SB27041, and Gamabufolatin showed moderate to variable potency or poor cytotoxicity profiles. Consequently, although several compounds demonstrate promising in silico binding and moderate in vitro activity, discrepancies in reported IC_50_, CC_50_, and K_i_ values, together with incomplete toxicity data, make it difficult to draw definitive conclusions regarding their therapeutic potential. These limitations are further compounded by the reliance on molecular docking as a primary tool, which is constrained by simplified scoring functions, inadequate treatment of protein flexibility and solvent effects, and poor correlation with experimental binding affinities [103]. Therefore, integrated computational–experimental approaches are essential for more reliable validation of entry-target inhibitors.

### 2.5. SARS-CoV-2 TMPRSS 2: Structure and Function

**Figure 6:**
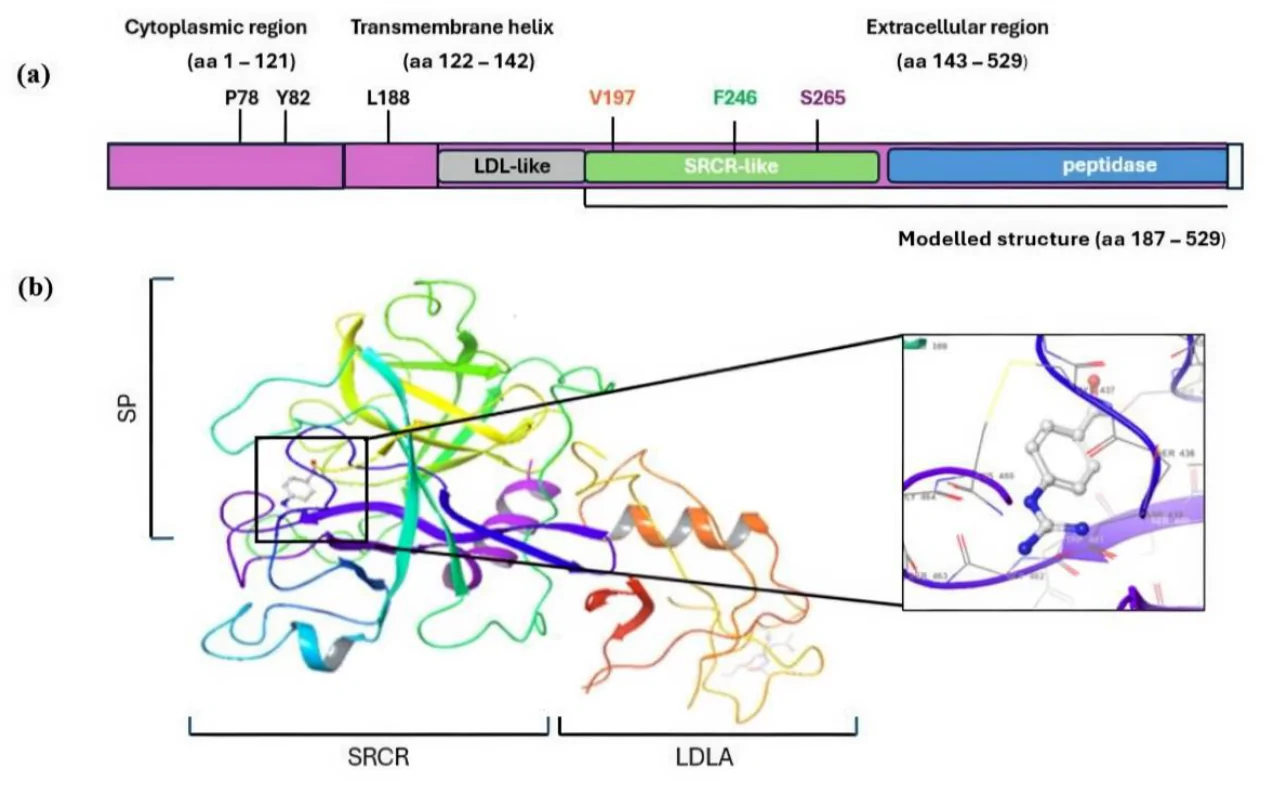
(**a**) Schematic representation of the amino acid sequence of SARS-CoV-2 TMPRSS2 showing the cytoplasmic region (purple), transmembrane helix (pink), low-density lipoprotein receptor class A-like (LDL-like; grey) domain, scavenger receptor cysteine-rich-like (SRCR-like; green) domain, and serine peptidase (blue) domain. Key residues (P78, Y82, L188, V197, F246, and S265) and the modeled region (aa 187–529) are indicated. Reproduced with permission [106]. Copyright 2021 MDPI. (**b**) A 3D structural presentation of SARS-CoV-2 S TMPRSS2 7MEQ, highlighting the serine protease (SP) domain, scavenger receptor cysteine-rich (SRCR) domain, and low-density lipoprotein receptor class A (LDLA) domain, and an extended view of the active site with ligand bound. Reproduced with permission [106]. Copyright 2023 ACS.

Transmembrane protease serine 2 (TMPRSS 2), a protease responsible for proteolytically processing the SARS-CoV-2 Spike protein, facilitating virus–host membrane fusion into human cells [4,93]. This protease comprises three subdomains, the LDLR-A, SRCR, and SP, each contributing to its function in viral entry and protein processing (Figure 6a,b). The LDLR-A domain is located around the region of amino acids 106 to 145 and is primarily involved in lipoprotein binding, which may facilitate interactions with cellular membranes. The SRCR domain, located around the region of amino acids 146 to 243, plays a key role in protein–protein interactions, and the serine protease (SP) domain spans the most critical region with amino acids 256 to 492. This domain accommodates the catalytic machinery of the protease enzyme and is responsible for its proteolytic activity (Figure 6a,b) [93]. The TMPRSS2 is particularly important for managing long-COVID symptoms as it is expressed in many epithelial cells, including those in the prostate, kidneys, bronchus, lungs, trachea, small intestine, pancreas, colon, salivary glands, and thymus, with the highest expression levels found in the lungs and airways [104]. The SARS-CoV-2 prefers the TMPRSS2 entry route compared to the alternative ‘endocytosis’ route, which is rather less efficient [105].

#### Inhibitors Targeting SARS-CoV-2 Transmembrane Protease Serine 2

**Figure 7:**
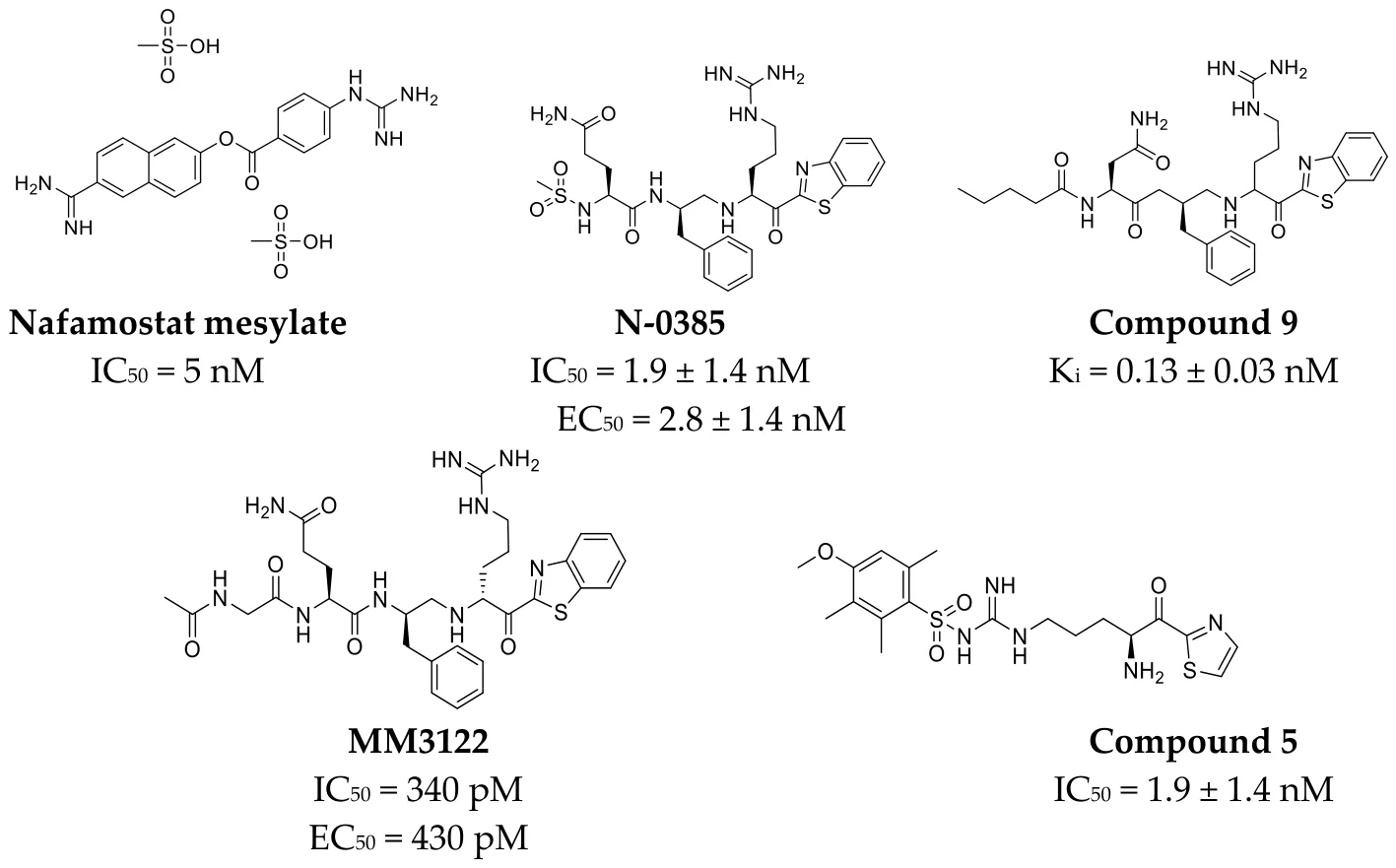
Experimental inhibitors targeting transmembrane serine 2 a viral entry target for SARS-CoV-2.

Camostat mesylate is one of the most prominent serine protease inhibitors active against TMPRSS2, with an IC_50_ of 87 nM; however, Hoffman and colleagues report a 17 times more potent inhibitor, Nafamostat mesylate, with a half-maximal inhibition concentration (IC_50_) of 5 nM [107]. A molecular modeling study by Shapira et al. using a crystal structure with PDB ID of 6N4T has led to the discovery of a TMPRSS2 inhibitor N-0385 with an EC_50_ of 2.8 nM against SARS-CoV-2. These compounds were assessed for antiviral efficacy against an ancestral SARS-CoV-2 strain (lineage B, VIDO) and four variants—Alpha (B.1.1.7), Beta (B.1.351), Gamma (P.1), and Delta (B.1.617.2)—in human lung cells, and provided significant therapeutic benefits [108]. Mahoney and colleagues employed rational structure-based drug design (SBDD) combined with substrate specificity screening to identify TMPRSS2 covalent inhibitors containing ketobenzo thiazole scaffolds. Among the identified compounds, MM3122 emerged as the lead candidate, exhibiting an IC_50_ of 340 pM and EC_50_ of 430 pM against recombinant full-length TMPRSS2 protein. These inhibitors demonstrated a significantly improved activity compared to existing drugs, such as Camostat and Nafamostat [109]. Wettstein et al. identified compound **2** and compound **5** as potent inhibitors of TMPRSS2, demonstrating a K_i_ value between 2.5 and 3.8 nM that has 3 times greater activity than the active metabolite FOY-251 with K_i_ = 9.7 nM. Both compounds effectively prevented cell-mediated proteolysis of a fluorogenic substrate, with IC_50_ of 12.7 nM for compound **5** and IC_50_ = 32 nM for compound **2** [110]. Ferková et al. reported a previously modified potential TMPRSS2 inhibitor, N-0385, to improve its pharmacodynamic and pharmacokinetic properties. In addressing some of these challenges, researchers developed a library of peptidomimetic compounds, introducing P3 site modifications to optimize selectivity and pharmacokinetics while maintaining sub-nanomolar potency, leading to the discovery of compound **9** with inhibitory potency of *K*_i_ = 0.13 ± 0.03 nM (Figure 7) [111]. Consequently, although several compounds demonstrate promising in silico binding and moderate in vitro activity, discrepancies in reported IC_50_, CC_50_, and K_i_ values, together with incomplete toxicity data, make it difficult to draw definitive conclusions regarding their therapeutic potential. These limitations are further compounded by the reliance on molecular docking as a primary tool, which is constrained by simplified scoring functions, inadequate treatment of protein flexibility and solvent effects, and poor correlation with experimental binding affinities as described in the previous section [103]. Furthermore, the accuracy of the docking score is system dependent and the energy terms in the scoring functions do not account for ligand and receptor’s strain energies.

### 2.6. Cathepsin L (CTSL): Structure and Function

**Figure 8:**
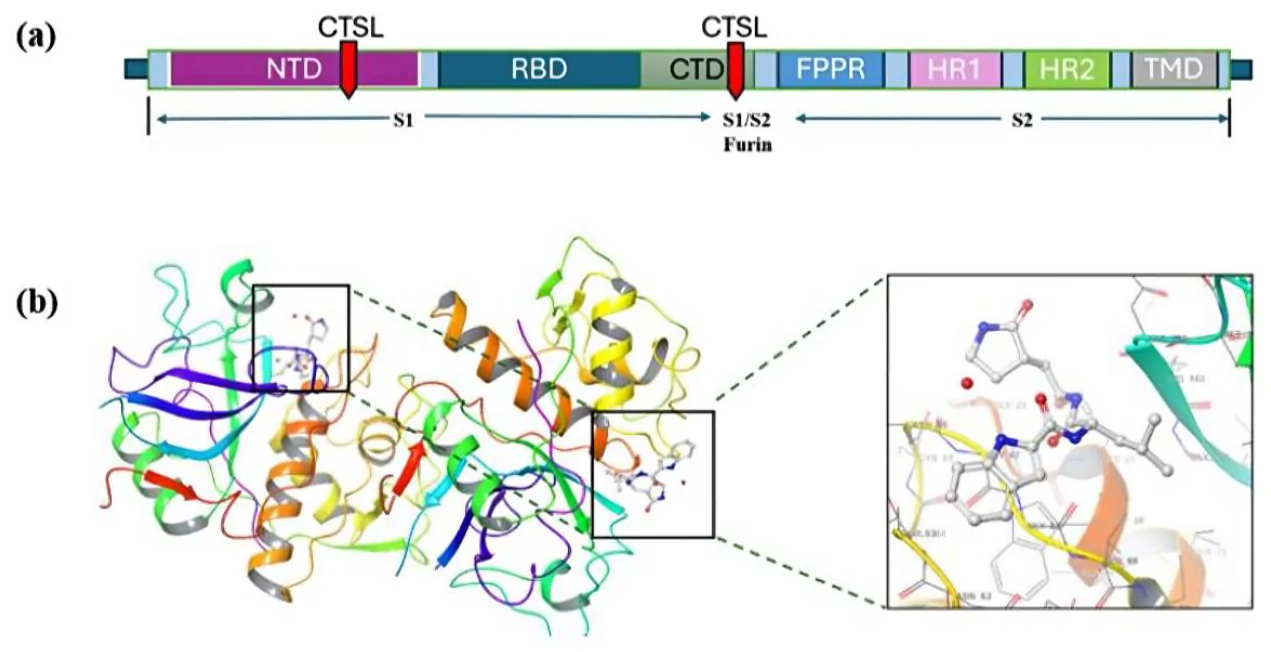
(**a**) A schematic representation of CTSL showing tthe N-terminal domain (NTD) in magenta, receptor-binding domain (RBD) in blue, C-terminal domain (CTD) in green, fusion peptide proximal region (FPPR) in light blue, heptad repeat 1 (HR1) in pink, heptad repeat 2 (HR2) in light green, and transmembrane domain (TMD) in grey, with Cathepsin L (CTSL) and furin cleavage sites indicated with red arrows. Reproduced with permission [114]. Copyright 2022 Springer Nature. (**b**) The fold of the two-chain form of native Cathepsin L (PDB entry: 8UAC) and an extended view of the active site with ligand bound in both monomers. Reproduced with permission [115]. Copyright 2024 Elsevier.

Cathepsin L (CTSL) is a three-dimensional structure that comprises an L domain of α-helix and an R domain of β-sheet. The L-domain comprises three α-helices, with the longest and the vertical helix also known as the central helix that further contains above 30 residues. The R-domain on the other side is made up of a β-barrel with the front strand(s) forming a coiled structure (Figure 8) [89]. These two catalytic residues form the thiolate–imidazolium ion pair, which is essential for the proteolytic activity of the enzymes [87,88,89]. This protease plays a significant role in the endosomal entry pathway of SARS-CoV-2 by facilitating viral entry into human cells through activation of the spike protein [87,88,89]. Cathepsin L protease is found in most human tissues, such as the kidney, bone marrow, respiratory system, sexual tissues, endocrine tissues, urinary bladder, gastrointestinal tract, and lymphoid tissues [89]. CTSL is more highly expressed in human lungs than the angiotensin-converting enzyme 2 (ACE2), transmembrane protease serine 2 (TMPRSS2), and furin [89]. Despite the limited literature on CTSL’s ability to prevent or treat SARS-CoV-2 virus infection, its overexpression in human cell lines boosts SARS-CoV-2 spike-mediated viral entry, making the CTSL one of the most promising targets to block the virus from entering the system [112]. It was believed that the Omicron variant entered the host cell through only Cathepsin L and not via transmembrane protease serine 2, and this claims Cathepsin L as the best target for viral entry; however, recent findings still highlight TMPRSS2 as the main entry pathway, even for Omicron [113].

#### Inhibitors Targeting SARS-CoV-2 Cathepsin L

**Figure 9:**
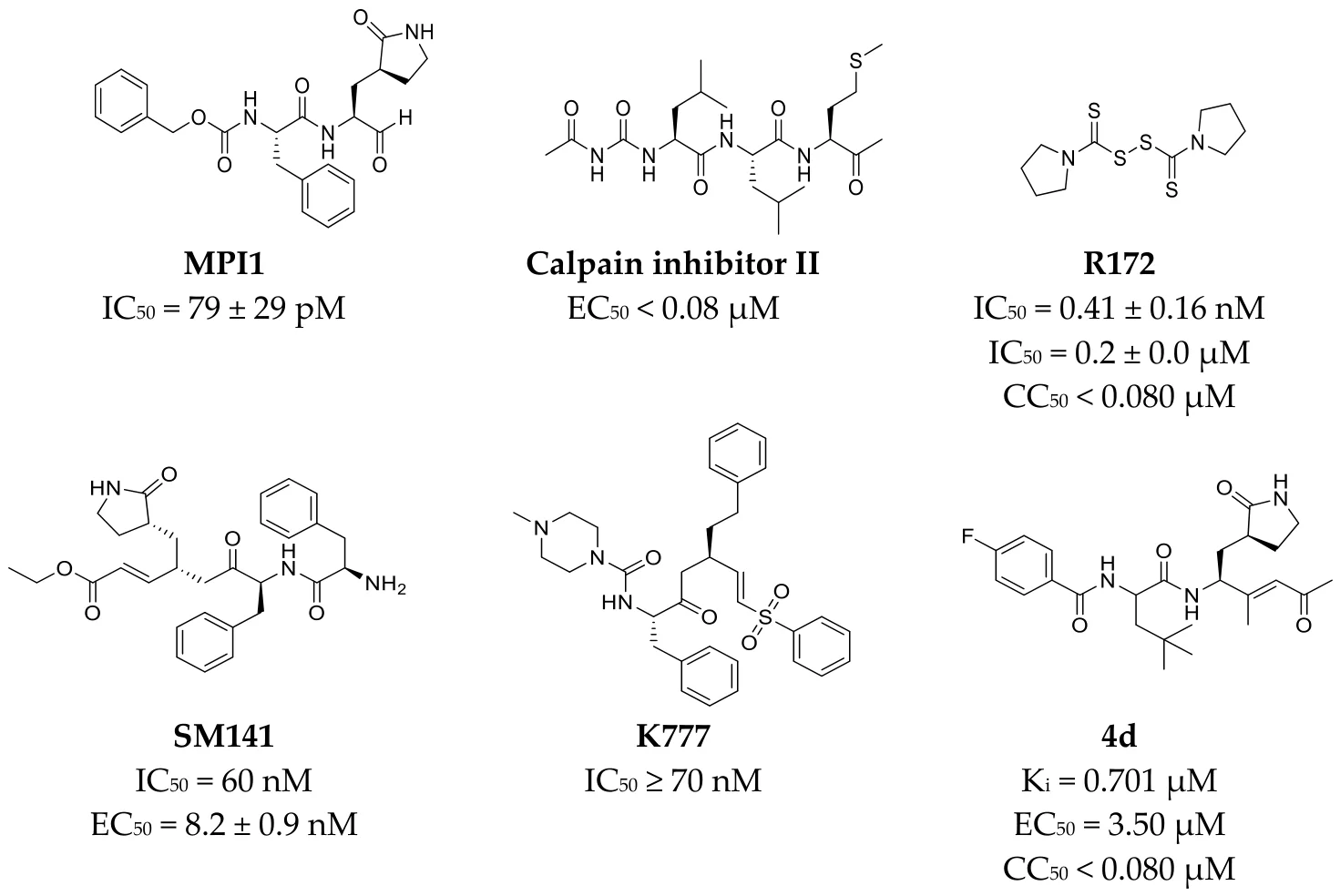
SARS-CoV-2 Cathepsin L experimental inhibitors.

Ma et al. designed a dual inhibitor, MPI1, through QSAR modeling with an IC_50_ of 79 pM against Cathepsin L and an IC_50_ of 105 nM against Mpro [88]. The SM141, a dual inhibitor for Mpro and Cathepsin L, has shown great potency against Cathepsin L with an antiviral activity in the nanomolar range [116,117]. The K777 is a covalent, irreversible inhibitor that is a candidate in Phase I clinical trials, demonstrating great potency against Cathepsin L with an antiviral activity of 74 nM in the Vero cell line [117]. Meewan et al. discovered triple inhibitors, with RI175, RI173, and RI172 being the most potent inhibitors in an enzymatic assay against Mpro, PLpro, and human Cathepsin L, with IC_50_ values of 300 nM, 200 nM, and 200 nM, respectively [118]. Sacco et al. investigated dual inhibitors, Calpain inhibitors II and XII, targeting both Mpro and Cathepsin L with inhibition concentrations of 0.41 nM, 1.62 nM for CTL, and 970 nM, 453 nM for Mpro, respectively [119]. Previt et al. conducted a similar study to Ma et al., reporting the use of dual inhibitors targeting both Mpro and human Cathepsin L through QSAR modeling. The most prominent compound **4**d, with K_i_ 0.70 µM and 5.54 µM for Cathepsin L and Mpro, respectively, has antiviral activity of EC_50_ = 3.50 μM, and CC_50_ values > 100 μM. This was achieved by varying aliphatic amino acids at the P2 site and fluorine position on the phenyl ring at the P3 site, which significantly influenced their binding affinity [120]. The ability of Mpro inhibitors to also target host proteases like Cathepsin L opens exciting possibilities for drug repurposing and lead optimization. Therefore, by modifying existing Mpro inhibitors, researchers can design dual or multi-target inhibitors, accelerating the discovery of broad-spectrum antivirals that combat SARS-CoV-2 and potentially other viral pathogens (Figure 9). Despite promising results, these dual inhibitors face challenges, including selectivity, toxicity, poor drug-likeness of peptidomimetics, and difficulties translating enzymatic inhibition into effective antiviral activity [121]. In addition, the underlying computational approaches, particularly QSAR and structure-based design, are limited by reliance on training data quality, simplified modeling assumptions, and limited predictive power for selectivity, pharmacokinetics, and efficacy [122].

### 2.7. SARS-CoV-2 Viral Replication Targets

**Figure 10:**
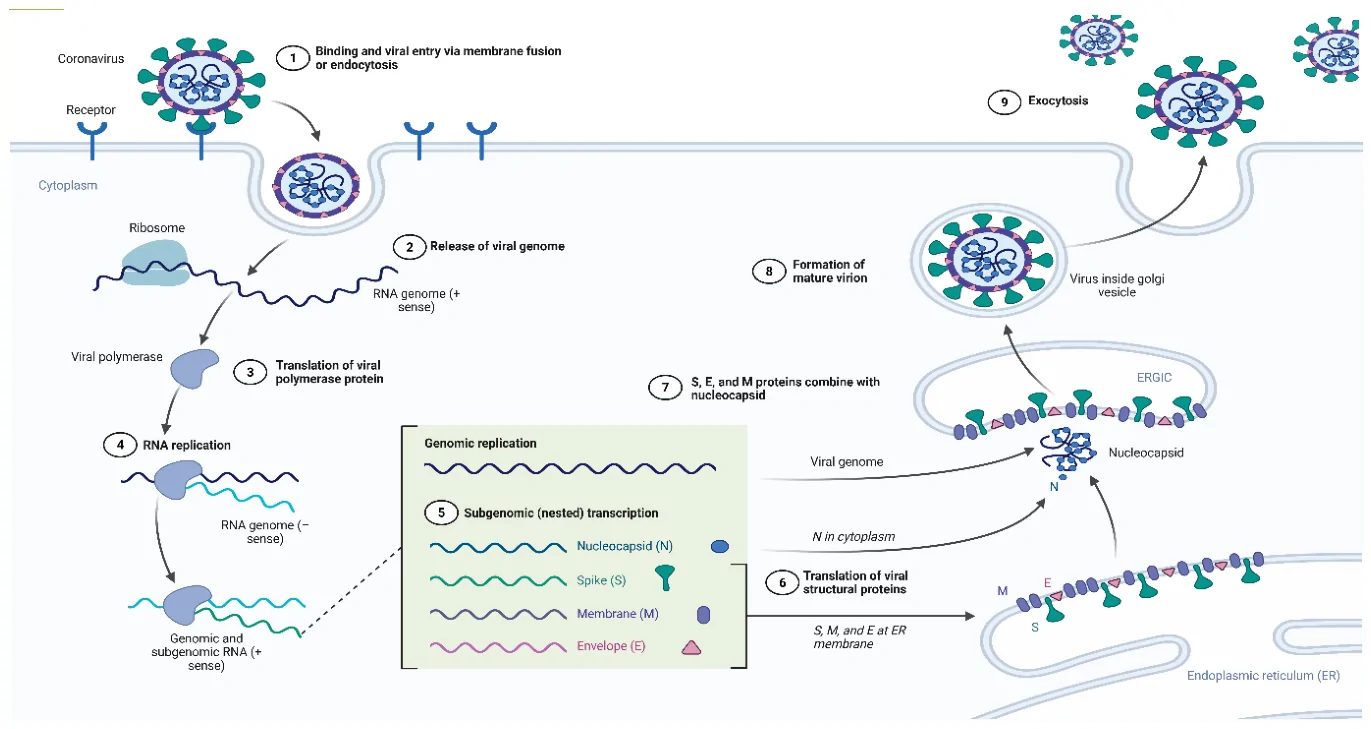
Key steps involved in SARS-CoV-2 viral replication. SARS-CoV-2 enters host cells through receptor-mediated attachment (blue), releases its RNA genome (dark blue) for replication and protein synthesis. Genomic and subgenomic RNAs are produced, giving rise to structural proteins including nucleocapsid (blue), spike (green), membrane (purple), and envelope (pink). New virions assemble in the ER–Golgi intermediate compartment (ERGIC) and are released by exocytosis. Image generated with Biorender.

Once the virus has entered the host, positive-sense ssRNA transcribes to produce non-structural proteins, including papain-like protease (PLpro) and main protease (Mpro) from large viral polyproteins Pp1a and Pp1ab (Figure 9) [31]. PLpro and Mpro play a massive role in viral replication and transcription as they proteolytically process proteins needed for viral replication [123]. On the other hand, structural proteins, including spike (S), membrane (M), and envelope (E), produced in the endoplasmic reticulum (ER), are transported to the ER–Golgi intermediate compartment (ERGIC) for virion assembly. The nucleocapsid (N) protein is a 419-amino acid, multi-domain RNA-binding protein with two conserved domains, the *N*-terminal domain (NTD) and the C-terminal domain (CTD), connected by an inherently disordered region called the central linking region (LKR). The N protein stabilizes and protects the viral genome by packaging it into the nucleocapsid, ensuring effective integration of virions during the replication cycle, assembly, and release phases [124,125]. The assembled virions in the ERGIC are then transported through smooth-wall vesicles to the cell membranes to release the mature virus particles (Figure 10) [124].

#### 2.7.1. SARS-CoV-2 Main Protease (Mpro): Structure and Function

**Figure 11:**
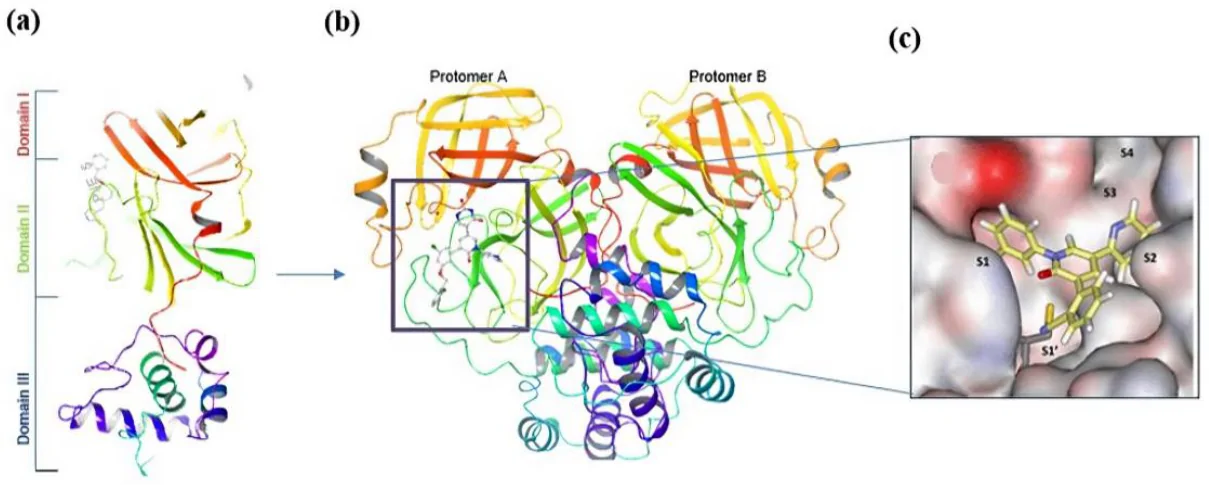
(**a**) SARS-CoV-2 Mpro monomer form (PDB: 6LU7) comprising Domain I (red–orange), Domain II (green), and Domain III (blue–purple). (**b**) Homodimeric structure of SARS-CoV-2 Mpro showing protomers A and B and the catalytic pocket (PDB: 7L13). (**c**) A close-up view of the catalytic binding site, made up of 4 subsites (S1′, S1, S2, S3, and S4) with a bound ligand. Reproduced with permission [129]. Copyright 2020 Springer Nature.

The main protease (Mpro), well known as 3 Chymotrypsin-like (3CL) or non-structural protein 5 (NSP5), is a three-domain homodimeric cysteine protease that plays a significant role in SARS-CoV-2 viral replication and transcription [45,123]. This protease comprises three domains (Figure 11a) classified as I (residues 8–101) and II (residues 102–184), each consisting of two protomers and six antiparallel β-barrels; and domain III (residues 201–303) consists of five α helices antiparallel globular cluster with active sites (S1′, S1, S2, and S3/S4) located between Domain I and Domain II, as shown in Figure 11b [126]. The S1′ region, located between Domains I and II, features a catalytic dyad, with Cys145 and His41. During peptide bond hydrolysis, His41 acts as a base and deprotonates the nucleophilic -SH group of Cys145. This activation permits the Cys145 residue to attack the peptide bond, leading to its cleavage, and the resulting intermediate is then stabilized by the “oxyanion hole”, formed by the backbones of Gly143 and Cys145. The S1 pocket comprises Phe140, His163, His164, Glu166, and His172, while S1′ has Cys145, S2 contains Met49, with Tyr54, Met165, Pro168, Val186, and, lastly, the S4 pocket contains Gln189, Ala191, Gln192, and Gly251. The Cys145-His41 catalytic dyad is responsible for the cleavage of the two large polyproteins produced by the virus during the invasion of the human host cell (Figure 11c) [49,51]. These sites interact with their respective fragments of inhibitors P1 (warhead containing amino acid), P1′, P2 (contains AA 2), and P3 (N-Cap, or a third AA) [51,127]. After the cell entry into the host, Mpro first auto-cleaves to become a mature enzyme and later cleaves all the 11 remaining downstream non-structural proteins of the polyproteins to polypeptides, which are required for the replication process of the virus [104,123]. The main protease inhibitors inhibit the catalytic cysteine residue (Cys145) through covalent and non-covalent interactions, and many developed inhibitors contain a warhead that reacts directly with Cys145 to form a covalent intermediate [88]. This is the most explored SARS-CoV-2 drug target, sharing 96.1% similarity with SARS-CoV Mpro [49,127,128].

#### 2.7.2. Inhibitors Targeting SARS-CoV-2 Main Protease

Mpro is the most explored target of all SARS-CoV-2 drug targets, with countless inhibitors in the pipeline under pre-clinical development, some in the clinical stage, and a few approved. Ahmad et al. performed molecular docking, molecular dynamics (MD) simulations, and binding free energy calculations through MM-PBSA for PF-07321332 (Nirmatrelvir), α-ketoamide, lopinavir, and Ritonavir on SARS-CoV-2 Mpro. This report revealed α-ketoamide as the most promising candidate with a binding energy of −102.002 kJ/mol after PF-07321332, with a binding energy of −74.065 kJ/mol. Ritonavir and lopinavir had the lowest binding energies of −43.525 kJ/mol and −33.497 kJ/mol, respectively, as determined by the MM-PBSA [130]. Another study by Hou et al. identified a noncovalent inhibitor, W04, which is active against the Mpro wild-type variant and omicron, with IC_50_ values of 72 nM and 53 nM, respectively, as determined by docking and free-energy perturbation [53]. An excellent review by Cannalire et al. summarized the results of Computer-Aided Drug Design (CADD) and biophysical and viral cell-based assays of Mpro inhibitors from the literature. The authors reported a ketoamide compound **3** that inhibited Mpro with an IC_50_ value of 0.67 µM using a biochemical assay with an EC_50_ of 4 µM in human lung cells (Calu3). The two most promising dipeptides, compounds **5** and **6**, were highlighted with IC_50_ of 53 and 40 nM, and EC_50_ of 0.53 and 0.72 µM, respectively [15]. Ahmed et al. screened 30 hits from FDA-approved antiviral drugs against SARS-CoV-2 in a cell-based viral replication assay. Only Atovaquone, Ouabain, Dronedarone, Mebendazole, and Entacapone exhibited some level of efficacy, with Atovaquone and Mebendazole being the only candidates with IC_50_ that fall within their therapeutic potential. They further performed an Mpro assay on the top hits, and only Dronedarone, with an IC_50_ of 18 μM, Mebendazole, with an IC_50_ of 19 μM, and entacapone, with an IC_50_ of 9 μM, exhibited significant Mpro inhibition [131]. Gosh et al. discovered a new Mpro inhibitor, 5e (GRL-190–21), with an IC_50_ of 44 pM by exploring P1 and P4 in modifying Nirmatrelvir. A high-resolution X-ray crystal structure also confirmed that the ligand-binding properties for 5e in the Mpro active site are comparable with those of Nirmatrelvir [50]. Another study by Dayan Elshan et al. explored SAR across all the SARS-CoV-2 Mpro regions based on knowledge of protease inhibitor designs from the literature, leading to the discovery of the clinical candidate CMX990 with approximately 5 times greater potency compared to Nirmatrelvir. This compound also maintained the correlation between in vitro and viral assay, with an IC_50_ of 23.4 nM and an EC_90_ of 10 nM [51]. Huang et al. report an α-ketoamide derivative with nanomolar activity confirmed by an IC_50_ value of 10.9 nM and an EC_50_ value of 43.6 nM against Mpro; this was a result of the structure–activity relationship (SAR) of the α-ketoamide compound and biological evaluation [132].

Majrashi and coworkers used pharmacophore-based virtual screening to screen 250 ZINC database chemical substances, which led to the discovery of four hits with acceptable docking scores within the range of −35.98 to −46.44 kcal/mol, respectively. Molecular dynamics (MD) and Molecular Mechanics Poisson–Boltzmann Surface Area (MM-PBSA) calculations have led to the discovery of W1 (ZINC000150656136), a potential Mpro inhibitor with a docking score of −11.1 kcal/mol and binding free energy of −324.7 ± 9.7 kJ/mol [133]. Chihab et al. reported indole derivatives as potential inhibitors for SARS-CoV-2 PLpro and Mpro using in silico ADMET prediction and molecular docking. Those compounds exhibited excellent docking scores with very low predicted inhibition constants (pK_i_) ranging between micromolar and nanomolar. Compounds **10** and **11** had pK_i_ values of 1.68 μM and 387.71 nM for Mpro, and 402.50 nM and 27.10 nM for PLpro [134]. Wang and colleagues developed a novel approach using DNA-encoded chemical library screening to identify noncovalent Mpro inhibitor 5, which had an IC_50_ value of 0.079 µM. They further utilized a macrocyclization strategy to stabilize the active conformation, resulting in lactone 12, which showed improved antiviral activity. Further optimization led to the potent lactam 26, with IC_50_ values of 0.006 µM against SARS-CoV-2, 0.033 µM against OC43, and 0.001–0.003 µM against Pan-CoV, demonstrating efficacy against Nirmatrelvir-resistant variants and various coronaviruses (Table 2 and Table S4) [135]. Carney and coworkers (2024) explored the structure–activity relationship (SAR) utilizing calculated thermodynamic maps of hydration sites, known as WaterMap, to enhance potency by displacing water from high-energy sites by designing thousands of diverse compounds to target these sites and prioritized them for synthesis using physics and structure-based free-energy perturbation (FEP) simulations, which accurately predicted their biochemical potencies leading to the discovery of a new Mpro inhibitor [136]. Compound **7**, with an EC_50_ of 7.1 μM and a ΔG of −3.5 kcal/mol, was selected for generating a co-crystal structure; however, it was not pursued as a lead due to its weak cellular activity. Compound **10** was selected as a lead for further optimization because it showed good potency against SARS-CoV-2 Mpro inhibition and broad-spectrum activity, with better safety than compound **9**, which had the highest potency (EC_50_ = 770 nM) but moderate cytotoxicity (CC_50_ = 26 µM), as determined in HeLa-ACE2 cells. The FEP+ model prediction showed a good correlation with the experimental values, all within a four-fold difference, highlighting its potential application in drug development.

Several computational approaches, including molecular docking, molecular dynamics (MD) simulations, free-energy perturbation (FEP), MM-GBSA/MM-PBSA, QSAR, and WaterMap analysis, have been widely applied in the design and evaluation of SARS-CoV-2 Mpro inhibitors. These methods have significantly contributed to understanding binding modes, ranking ligand affinities, and guiding lead optimization. However, each approach has inherent limitations. Docking relies on simplified scoring functions and often neglects protein flexibility and solvent effects, resulting in lower accuracy in predicting true binding affinities [137]. MD and MM-GBSA or MM-PBSA/FEP offer more rigorous insights into binding energetics but are computationally demanding and sensitive to force-field parameters and system preparation [138]. In addition, pharmacophore-based and virtual screening approaches may yield false positives or overlook novel scaffolds because they rely on predefined features. Furthermore, while methods such as WaterMap improve the consideration of hydration thermodynamics, they remain computationally intensive and depend on accurate sampling of water networks [137]. This, therefore, highlights the need for integrated, multi-method approaches that combine efficiency with improved physical realism and predictive accuracy in drug discovery, including AI-integrated approaches and machine learning [139,140].

**Table 2:** Pharmacological and physicochemical profiles of experimental inhibitors targeting SARS-CoV-2 Mpro.

Name ^1^	IC_50_	K_i_ (nM)	Type of Mpro Assay	EC_50_	Type of Viral Assay	CC_50_	CL_int_ (ml/min/mg)	Metabo-lite ID	LogD	Approval Status	Design Strategy	References
Ebselen	9.35 ± 0.18 µM	ND*	Enzymatic Assay	4.67 μM	Vero E6	ND*	ND*	ND*	ND*	Experimental	Repurposed	[129]
Mebendazole	19.94 µM	ND*	Enzymatic assay	1.30 µM	Vero E6	>100 µM	ND*	ND*	ND*	Experimental	Repurposed	[131]
Dronedarone	18.03 µM	ND*	Enzymatic assay	1.50 µM	Vero E6	22.70 µM	ND*	ND*	ND*	Experimental	Repurposed	[131]
Entacapone	9.04 µM	ND*	Enzymatic assay	ND*	Vero E6	>100 µM	ND*	ND*	ND*	Experimental	Repurposed	[131]
Nirmatrelvir, 1	ND*	3.11 nM	FRET assay	81.4 nM	Vero E6	ND*	0.025	CYP3A4	1.9	FDA approved	Retention of pep-tidomimetic chemotype	[49]
Nirmatrelvir	28.1 nM	ND*	Enzymatic fluorescence assay	148 nM	HeLa-ACE2	>40 µM	0.0328	CYP3A4.	1.5	FDA approved	Retention of pep-tidomimetic chemotype	[51]
Nirmatrelvir	2.57 nM	ND*	Mpro biochemical assay	ND*	CPE -Reduc-tion	ND*	ND*	CYP3A4	ND*	FDA approved	Retention of pep-tidomimetic chemotype	[141]
Nirmatrelvir	ND*	0.003 ± 0.04 µM	Enzymatic assay	<0.01 µM	Huh-7-ACE2	>100 µM	ND*	ND*	ND*	FDA approved	Retention of pep-tidomimetic chemotype	[120]
PF-07817883, 9	18 nM	2.48 nM	FRET assay	180 nM	Vero E6	ND*	0.0047	CYP3A4/5	0.9	Phase II	Retention of pep-tidomimetic chemotype	[49]
PF-07817883, (Ibuzatrelvir)	19	5 nM	FRET-based Mpro activity and inhibition assay	ND*	ND*	ND*	0.0047	ND*	ND*	Phase II	Retention of pep-tidomimetic chemotype	[142]
30	0.76 µM	ND*	Enzymatic fluorescence assay	4.88 µM	Vero	>300 µM	ND*	ND*	ND*	Experi-mental	Inhibitor Design Targeting Cys44 in the S2 Pocket	[143]
PF-00835231	101 nM	ND*	Enzymatic fluorescence assay	630 nM	HeLa-ACE2	ND*	0.0139	ND*	1.2	Lead	hydroxymethyl ketone warhead	[51]
CMX990	23.4 nM	ND*	Enzymatic fluorescence assay	9.6 nM	HeLa-ACE2	>40 µM	0.0075	No CYP450	2.3	Phase I	trifluoromethoxymethyl ketone warhead	[51]
Com-pound 1	ND*	ND*	Mpro biochemical assay	ND*	Vero E6	ND*	0.1858	ND*	1.65	Lead	Peptidomimetic with tetrafluoro-phenoxymethyl ketone warhead	[141]
MAT-POS-e194df51-1	0.037 µM	ND*	Mpro fluorescence assay	0.064 µM	Vero-E6 (Viral replication)	>50 µM	0.0000001 52	Cyp450 2C9/2D6/3 A4	2.5	Lead	AI/FEP & Crowdsourcing	[144]
119	15.7	ND*	Mpro FRET-based enzymatic assay	ND*	ND*	ND*	2.011	ND*	4	Lead	AI-generated Di-azepane Scaffold	[145]
Schaftoside	1.73 µM	ND*	Enzymatic assay	11.83 µM	Vero E6	200 µM	ND*	ND*	ND*	Experimental	plant based	[146]
GD-3	1.60 ± 0.2 µM	ND*	Enzymatic assay	183.0 ± 20.6 nM	Vero E6	>100 µM	ND*	ND*	ND*	Experimental	Nonpeptidic Piperazine Derivatives	[147]
Bepridil	72 ± 3 µM	ND*	FRET-based assay	0.86 µM	Vero E6	ND*	ND*	ND*	ND*	Experimental	Repurposed	[148]
Bardoxolone methyl	5.81 ± 0.79 µM	ND*	Enzymatic assay	0.29 µM	Vero	6.94 µM	Vero E6	ND*	ND*	Phase 2 (NCT0449 4646)	Compounds with electrophilic moieties	[149]
Bardoxolone	27.99 ± 2.34 µM	ND*	Enzymatic assay	0.43 µM	Vero	24.36 µM	Vero E6	ND*	ND*	Experimental	Compounds with electrophilic moieties	[149]
9a	1.66 ± 0.02 μM	ND*	Enzymatic assay	15.7 ± 8.3 μM	Vero E6	ND*	ND*	ND*	ND*	Experimental	α-ketoamides	[150]
Compound **18**	6.1 ± 0.5 µM	ND*	Enzymatic assay	2.4 ± 0.1 µM	DENV2 pro-HeLa	55.2 ± 8.5 µM	ND*	ND*	ND*	Experimental	Benzoxaborole-based inhibitors	[151]
JMX028 6	4.8 µM	ND*	FRET-based enzymatic assay	2.251 µM	A549-hACE2	3.53.1 µM	ND*	ND*	ND*	Experimental	Niclosamide derivatives	[152]
JMX0941	3.9 µM	ND*	FRET-based	1.7 µM	A549-hACE2	3.0 µM	ND*	ND*	ND*	Experimental	Niclosamide derivatives	[152]
JMX030 1	4.5 µM	ND*	FRET-based enzymatic assay	No dose response curve	A549-hACE2	342.4 µM	ND*	ND*	ND*	Experimental	Niclosamide derivatives	[152]
10b	0.374 ± 0.006 μM	ND*	Enzymatic assay	1.06	VeroE6	>100	ND*	ND*	ND*	Experimental	Tripeptidomimetic inhibitors	[153]
Oridonin	2.16 ± 0.22 µM	ND*	Fluorescence-based TSA	4.95 µM	Vero E6	24.94 µM	ND*	ND*	ND*	Experimental	Repurposed	[154]
MPI8	105 nM	ND*	Enzymatic assay	0.030 µM	Vero E6	109.2 µM	ND*	ND*	ND*	Experimental	Aldehyde-based tripeptidyl inhibitors	[155]
MPI43	45 ± 5 nM	ND*	Enzymatic assay	0.61 µM	Vero E6	34.2 µM	ND*	ND*	ND*	Experimental	Boceprevir-based main protease inhibitors	[101]
MPI44	59 ± 7 nM	ND*	Enzymatic assay	2.94 µM	Vero E6	143.7 µM	ND*	ND*	ND*	Experimental	Boceprevir-based main protease inhibitors	[101]
MPI46	120 ± 10 nM	ND*	Enzymatic assay	1.08 µM	Vero E6	163.4 µM	ND*	ND*	ND*	Experimental	Boceprevir-based main protease inhibitors	[101]
F8–B6	1.57 ± 0.08 µM	ND*	Enzymatic assay	ND*	ND*	ND*	ND*	ND*	ND*	Experimental	Analogs of F8–B1 to F8–B22	[156]
19	0.077 µM	ND*	Hydrolysis assay	0.11 ± 0.03 µM	Vero E6	>20 µM	ND*	ND*	ND*	Experimental	Merge compounds **8** and **9**	[157]
Y180	8.1 nM	ND*	Enzyme kinetics assay	11.4 nM	VeroE6-TMPRSS2	>81 µM	ND*	ND*	ND*	Experimental	α-ketoamide-con-taining	[158]
F01	54 µM	ND*	Enzymatic assay	150 µM	Vero-81	>400 µM	ND*	ND*	ND*	Experimental	Fragment-based	[159]
2a	0.18 ± 0.03 µM	ND*	Biochemical assay	0.035 ± 0.001 µM	Vero E6	>100 µM	ND*	ND*	ND*	Experimental	Aldehyde warhead or a latent aldehyde bisulfite adduct	[160]
3a	0.17 ± 0.02 µM	ND*	Biochemical assay	0.032 ± 0.001 µM	Vero E6	>100 µM	ND*	ND*	ND*	Experimental	Aldehyde warhead or a latent aldehyde bisulfite adduct	[160]
Z-VAD(Ome)-FMK	0.59 ± 0.44 µM	ND*	Hydrolytic activity	1.88 ± 0.52 µM	Vero E6	>300 µM	ND*	ND*	ND*	Experimental	Caspase inhibitors	[161]
26	0.170 ± 0.022 µM	ND*	FRET assay	2.0 ± 0.7 µM	MTT assay	>100 µM	ND*	ND*	ND*	Experimental	Cyanophenyl analogs	[104]
11a	53 ± 5 nM	ND*	FRET assay	0.53 ± 0.01 µM	Vero E6	>100 µM	ND*	ND*	ND*	Experimental	ND*	[162]
11b	40 ± 2 nM	ND*	FRET assay	0.72 ± 0.09 µM	Vero E6	>100 µM	ND*	ND*	ND*	Experimental	ND*	[162]
MI-12	19.0 ± 0.6 nM	ND*	FRET assay	0.53 ± 0.07 µM	Vero E6	>500	0.000003117	ND*	ND*	Experimental	Boceprevir or telaprevir-based	[163]
MI-30	17.2 ± 0.6 nM	ND*	FRET assay	0.54 ± 0.13 μM	Vero E6	>500 μM	0.000000171	ND*	ND*	Experimental	Boceprevir or telaprevir-based	[163]
41	0.068 ± 0.023 µM	ND*	FRET assay	0.497 ± 0.009 µM	Vero E6	ND*	ND*	ND*	ND*	Experimental	ML300 derivatives	[164]
14c	0.17 ± 0.07 µM	ND*	Enzymatic assay	ND*	ND*	ND*	ND*	ND*	ND*	Experimental	X77 derivatives	[165]
2j	0.75 ± 0.2 µM	ND*	FRET assay	ND*	ND*	ND*	ND*	ND*	ND*	Experimental	3,7-diazabicyclo[3.3.1] nonane (bispidine) derivatives	[166]
Boceprevir	3.1 µM	ND*	FRET assay	ND*	ND*	ND*	ND*	ND*	ND*	Hit	Repurposed	[167]
Calpeptin	ND*	ND*	ND*	0.072 µM	Vero E6	>100 µM	ND*	ND*	ND*	Experimental	Repurposed	[168]
Entrectinib	10.6 µM	ND*	Enzymatic assay	198 ± 116 nM	Vero E6	ND*	ND*	ND*	ND*	Experimental	Repurposed	[169]
Ethacridine	3.54 ± 0.66 µM	ND*	ND*	0.08 ± 0.01 µM	Vero E6	>40 µM	ND*	ND*	ND*	Experimental	Repurposed	[170]
CDD-1713	5.19 µM	24 ± 3 nM	Enzymatic assay	ND*	ND*	ND*	ND*	ND*	ND*	Hit	DNA-encoded chemistry technology	[171]
PUG	4.62 ± 0.27 µM	ND*	FRET assay	7.20 ± 1.08 µM	Vero E6	>100 µM	ND*	ND*	ND*	Experimental	Repurposed	[172]
Compound **4**	151 ± 15 nM	ND*	Enzymatic assay	2.883 ± 0.227 µM	Vero E6	ND*	ND*	ND*	ND*	Experimental	Peptidomimetic	[173]
N3	ND*	ND*	ND	16.77 ± 1.7 µM	Vero E6	>133 μM	ND*	ND*	ND*	Experimental	Repurposed	[129]
6e	0.17 ± 0.06 µM	ND*	FRET assay	0.15 ± 0.14 μM	Vero E6	63.3 ± 2.3 μM	ND*	ND*	ND*	Experimental	Broad-spectrum inhibitors	[174]
13b-K-13	0.12 ± 0.03 µM	ND*	FRET assay	1.3 µM	Vero E6	ND*	ND*	ND*	ND*	Experimental	Peptidomimetic	[175]
Compound **5**h	ND*	17.6 ± 3.2 nM	FRET assay	4.2 ± 0.7 µM	Vero E6	>100 µM	ND*	ND*	ND*	Experimental	Indole based	[176]
Shikonin	15.0 ± 30 µM	ND*	FRET assay	0.32 µM	Vero E6	>100 µM	ND*	ND*	ND*	Experimental	Repurposed	[177]
GC376	0.19 ± 0.04 µM	ND*	FRET assay	0.9 ± 0.2 µM	Vero E6	>200 µM	ND*	ND*	ND*	Experimental	Covalent inhibitor	[178]
MR6-31-2	0.824 ± 0.044 µM	ND*	FRET assay	1.78 ± 0.073 µM	Vero E6	ND*	ND*	ND*	ND*	Experimental	Ebselen derivatives	[179]
Mastinib	2.5 µM	ND*	Enzymatic assay	3.2 µM	A549	>10 µM	ND*	ND*	ND*	Experimental	Repurposed	[180]
FB2001	0.053 µM	ND*	FRET assay	0.39 ± 0.01 µM	Vero E6	274.4 µM	ND*	ND*	ND*	Experimental	Peptidomimetic	[181]
NBH-2	ND*	ND*	ND*	13.9 µM	HEK29 3-ACE2-TMPRSS2	>10 µM	ND*	ND*	ND*	Experimental	Narlaprevir- and boceprevir-derived hybrid inhibitors	[182]
19	0.044 ± 0.009 μM	ND*	Enzymatic assay	0.175 ± 0.05 μM	Vero E6	>80 μM	ND*	ND*	ND*	Experimental	Optimization of triarylpyridinone inhibitors	[183]
23R	0.20 ± 0.01 μM	ND*	Enzymatic assay	1.27 ± 0.09 μM	Vero E6	>100 µM	ND*	ND*	ND*	Experimental	Rational design of non-covalent inhibition	[184]
E24	2.77 ± 0.51 μM	ND*	FRET assay	0.8 ± 0.3 μM	Vero E6	ND*	ND*	ND*	3.18	Experimental	2-Phenyl-1,2-benzoselenazol-3-one analogs	[185]
32	0.5 ± 0.1 μM	ND*	Enzymatic assay	ND*	ND*	ND*	ND*	ND*	ND*	Hit	Penicillin V derivatives	[186]
C1	1.55 ±0.21 μM	6.09 μM	Enzymatic assay	ND*	ND*	ND*	ND*	ND*	ND*	Experimental	9,10-dihydrophe-nanthrene derivatives	[187]
Ouabain	ND*	ND*	ND*	0.03 ± 0.89 μM	Vero E6	>100 µM	ND*	ND*	ND*	Experimental	Repurposed	[131]
5	53 nM	ND*	Enzymatic assay	0.52 μM	Vero E6	>100 µM	ND*	ND*	ND*	Experimental	Targeting proteases and polymerase	[15]
A33	27.1 nM	ND*	FRET-based assay	ND*	ND*	ND*	ND*	ND*	5.84	Experimental	Pyrimidone derivatives	[188]
WU-04	72 ± 6 nM	ND*	FRET-based assay	12 ± 1 nM	A549-hACE2	>20	ND*	ND*	ND*	Experimental	DNA-encoded library	[53]
4d	ND*	5.54 ± 0.31 μM	Enzymatic Assay	3.50 ± 1.1 μM	Huh-7-ACE2	>100 µM	ND*	ND*	ND*	Experimental	Dual inhibitors	[120]
5e	ND*	0.04 nM	FRET-based assay	0.26 μM	Vero E6	ND*	ND*	ND*	ND*	Experimental	Exploring P1 and P4 of Nirmatrelvir	[50]
9g	35 ± 1 nM	ND*	FRET-based assay	ND*	ND*	ND*	ND*	ND*	ND*	Experimental	Thioamide-linked spiropyrrolidine derivatives	[54]
3 (S-217622)	0.013 μM	ND*	Enzymatic assay	0.37 μM	Vero E6	ND*	0.0000017	ND*	ND*	Phase 3 (NCT0530547)	Structure-based optimization	[189]
17 (S-892216)	0.655 nM	ND*	Enzymatic assay	2.48 nM	HEK 293T	ND*	0.00000679	ND*	ND*	Experimental	Reversible covalent warhead	[189]
Ol-gotrelvir	ND*	ND*	ND	1.38 μM	Vero E6	ND*	0.000011	ND*	ND*	Phase 1 (NCT0536 4840 & N CT05523739)	Covalent inhibitor	[115]
5	0.008 ± 0.001 μM	ND*	FRET-based assay	ND*	ND*	ND*	ND*	ND*	ND*	Experimental	Varying nirma-trelvir warheads	[190]
7	60 ± 10 nM	ND*	FRET-based assay	0.9 ± 0.1 μM	Vero E6	>200 µM	ND*	ND*	ND*	Experimental	Aldehyde and bisulfate derivatives	[191]
NK01-63	16 nM	ND*	qRT-PCR assay	6 nM	Huh-7ACE2	>100 µM	ND*	ND*	ND*	Experimental	GC376 analogs	[192]

^1^ Indicates the manuscript where Nirmatrelvir was first reported. ND* indicates the experimental data that was not determined.

**Figure 12:**
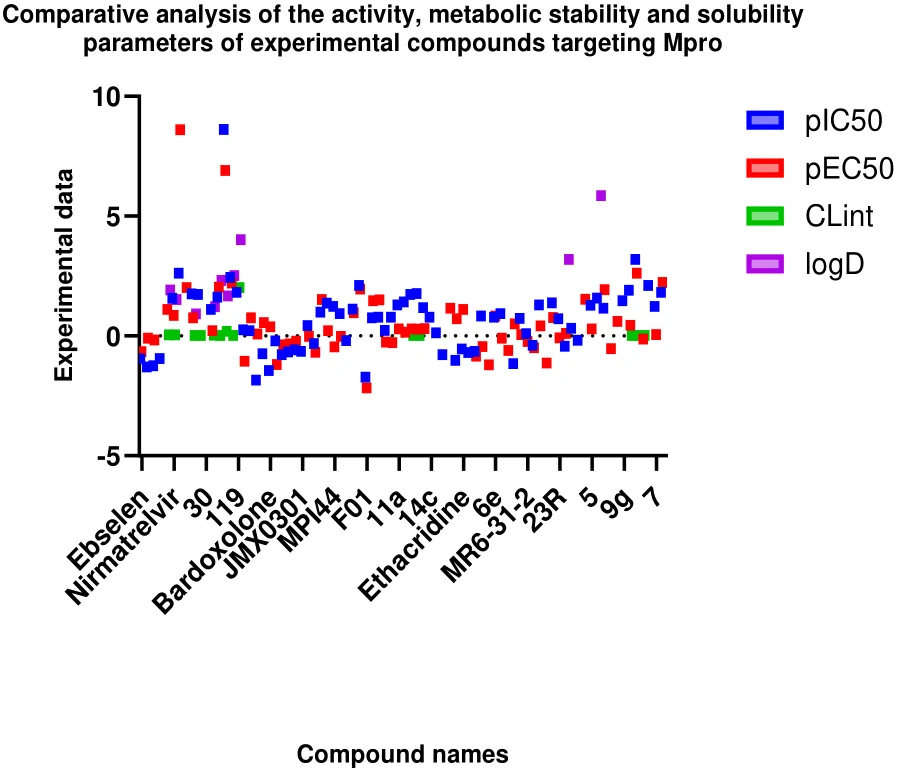
Comparative analysis of bioactivity, viral activity, metabolic stability, and solubility of compounds targeting Mpro reported in the literature.

Ordinarily, the design of compounds and their testing for the SARS-CoV-2 Mpro target needs to test the following parameters: pIC_50_, pEC_50_, metabolic stability, and solubility parameters. As such, we have analyzed the data, and a comparative analysis of all the parameters mentioned is illustrated in Figure 12. Some of the compounds lack data for certain metrics; most exhibit pIC_50_ and pEC_50_ values within the range of −2 to 2, with fewer outliers demonstrating higher activity with pIC_50_ ranging from 2 to 8.61 and pEC_50_ of up to 8.59. Compound **1**, listed in the foregoing Table 2, is the most active compound with the highest pIC_50_ of 8.61, the highest pEC_50_ of 6.98 in viral assay (Vero cell line), and a reasonable intrinsic clearance of 12.5 μL/min/106 cells in human hepatocytes [141]. Looking at Nirmatrelvir, it is more active in terms of viral activity, with a pEC_50_ of 8.59; however, in terms of pIC_50_, it is 3-fold lower, with a value of 2.6, and its intrinsic clearance is also low, as is its logD. However, compounds MAT-POS-e194df51-1, Y180, WU-04, 17(S892216), and NK01-63 have a correlative data of inhibitory activity and viral assay; they are active in terms of the experimental IC_50_ as well as the pEC_50_ data with values of 2.43 and 2.19; 2.09 and 1.94; 1.14 and 1.92; 3.18 and 2.61; and 1.8 and 2.22, respectively. Notably, the intrinsic clearance of compound MAT-POS-e194df51-1 and 17(S892216) is low; however, MAT-POS exhibits a reasonable logD value of 2.5. Compound **119** has a positive pIC_50_ value of 1.8, a good logD value of 4, and a moderate intrinsic clearance of 2011 µL/(min · mg). The compound that showed the most negative results was F01, with a pIC_50_ value of −1.73 and a pEC_50_ value of −2.18, which was an outlier. There is a direct correlation between pIC_50_ and pEC_50_ values, indicating that as the inhibitory activity of the compound in the cell-based assay increases, its activity in the viral assay also increases. The data in Figure 12 represent a similar trend to the data in Figure S1 of the supporting document, which includes all Mpro inhibitors. Among the covalent inhibitors reported to have high inhibitory activity, areas for improvement are highlighted, particularly in terms of metabolic stability and resistance to other variants. The COVID Moonshot organization reported a novel non-covalent inhibitor with a positive value greater than 1 for both pIC_50_ and pEC_50_, indicating high efficacy. Compound **1** is the most active inhibitor, with the most positive pIC_50_ and pEC_50_ values; however, it requires co-administration to improve its physicochemical properties, similar to Nirmatrelvir. This is a dual inhibitor as it also inhibits Cathepsin L with a pIC_50_ value of 7.54. This is a peptidomimetic inhibitor bearing a phenoxymethyl ketone warhead that covalently binds to Cys145 and has a logD of 1.65 [141]. Nirmatrelvir exhibits varying experimental values across different assay types, underscoring the need to validate an assay type for Mpro targets.

## 3. Covalent Inhibitors

**Figure 13:**
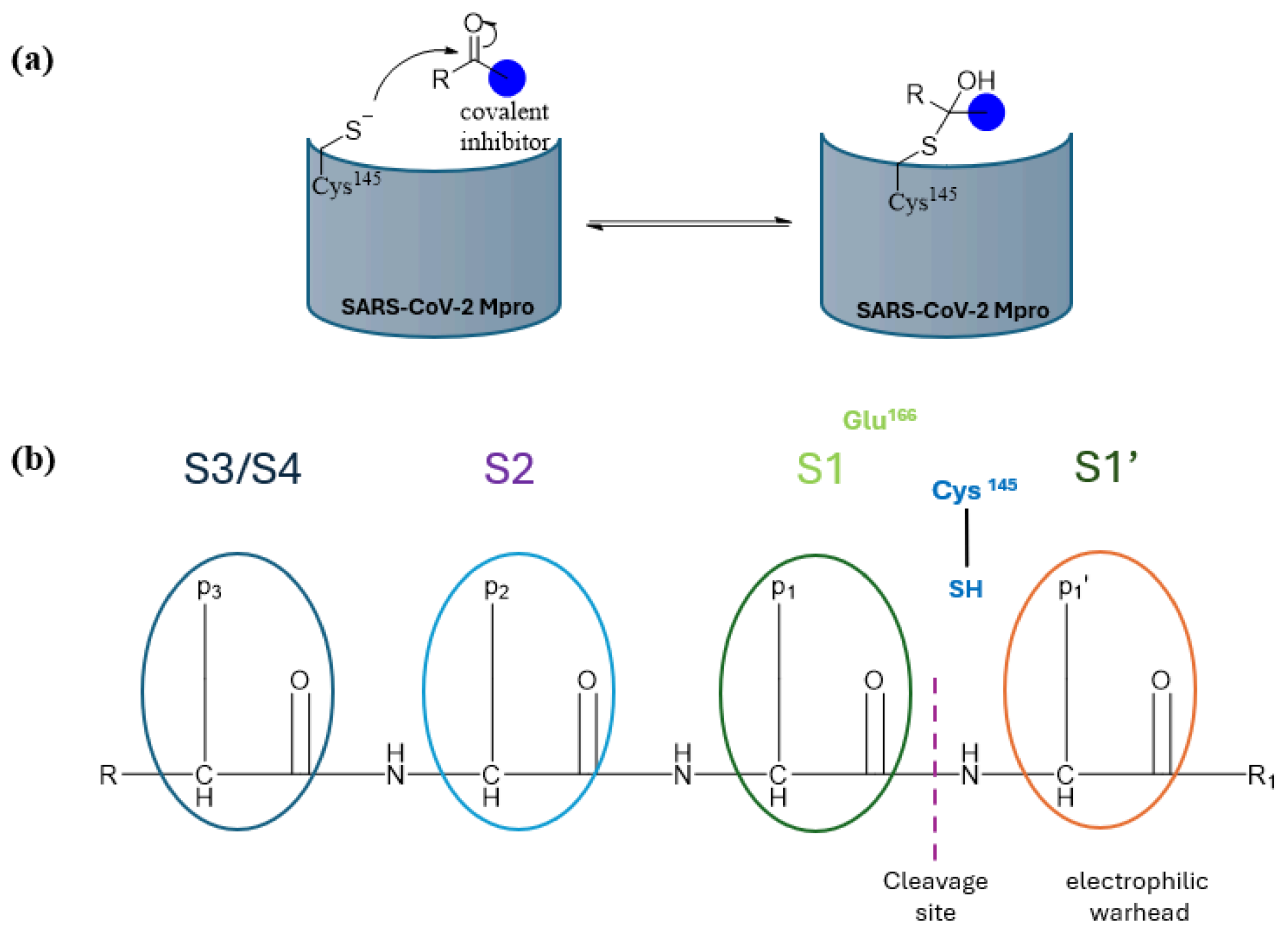
SARS-CoV-2 Mpro covalent inhibition and substrate-binding pockets. (**a**) Covalent bond formation between the catalytic residue Cys145 and an electrophilic warhead (blue). (**b**) Schematic representation of the Mpro substrate-binding pockets: S3/S4 (dark blue), S2 (light blue), S1 (green), and S1′ (orange), showing the positions of P3–P1′ substituents and the catalytic cleavage site (purple dashed line). Reproduced with permission [194]. Copyright 2022 ACS.

Covalent inhibitors have since been the most explored inhibitors for pan coronaviruses, including MERS and SARS-CoV-1, against different proteases, with remarkable results [193]. In addition, electrophilic warhead inhibitors show better efficacy and potency, better pharmacological action and overcome resistance. Mpro is the most conserved target between SARS-CoV and SARS-CoV-2, with about 96.1% similarity; hence, it is the most researched target. The design strategy for Mpro covalent inhibitors for SARS-CoV-2 is peptidomimetics with the electrophilic warhead that binds to Cys145, forming a covalent bond at the P1′ pocket (Figure 13a). The blockage of the viral replication is achieved by the cleavage of the glutamine amino acids and the peptide bond of the adjacent amino acid between the P1 and P1′ pocket (Figure 13b) [194]. Peptide aldehyde-based inhibitors can achieve excellent potency and selectivity by mimicking the interactions between the target protease and its substrate, while the C-terminal aldehyde forms a reversible covalent adduct with the nucleophilic active site cysteine. However, peptides typically exhibit low passive permeability through cellular membranes due to their polarity and excess number of hydrogen bond donors [136]. Covalent inhibitors are further classified into two groups: reversible covalent inhibitors that easily dissociate, unlike non-reversible covalent inhibitors [195,196]. Reversible covalent inhibitors are generally preferred, as their temporary binding allows their effects to wear off over time, reducing the risk of long-term toxicity [196]. Examples of reversible covalent inhibitors include Nirmatrelvir [49], CMX990 [51], Ibuzatrelvir [51,142], and many more. Covalent binding can enable targeting of especially challenging targets, such as the ValUb region in SARS-CoV-2 PLpro, for example, XR8-24 [197]. Irreversible inhibitors typically offer strong antiviral activity, long-lasting binding [195], improved selectivity [198], and targeting shallow binding sites [199]. However, selectivity remains the major concern, as unintended interactions can lead to adverse effects that are difficult to reverse [200]. Compound **17** is a typical example of an irreversible covalent inhibitor for SARS-CoV-2, with an enhanced potency (IC_50_ of 2.72 µM) compared to its parent compound, ML188, a reversible covalent inhibitor (IC_50_ of 3.14 mM). This was developed by Zaidman et al. using an automated computational pipeline to design irreversible covalent inhibitors targeting SARS-CoV-2 main protease (Mpro) based on non-covalent binders [201].

### 3.1. Quantitative Structure–Activity Relationship for Mpro Inhibitors

**Figure 14:**
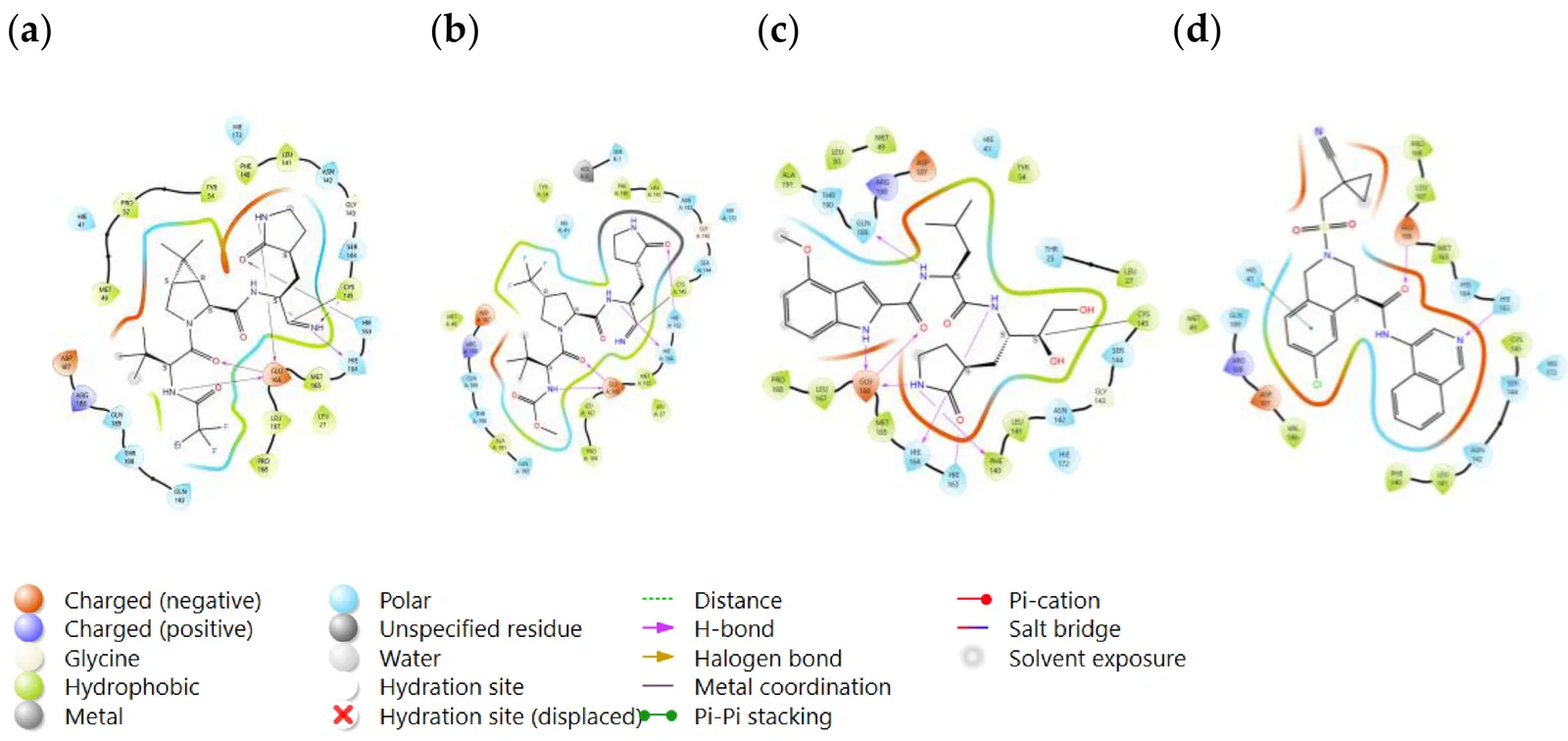
Ligand interaction diagram (LID) showing the amino acids involved in hydrogen bonding, π-π, and hydrophobic interactions between Mpro inhibitors with their respective Mpro receptors: (**a**) Nirmatrelvir (7U29), (**b**) Ibuzatrelvir (9BPF), (**c**) PF-00835231 (8J32), (**d**) MAT-POS e194df51-1 (7GAW).

**Figure 15:**
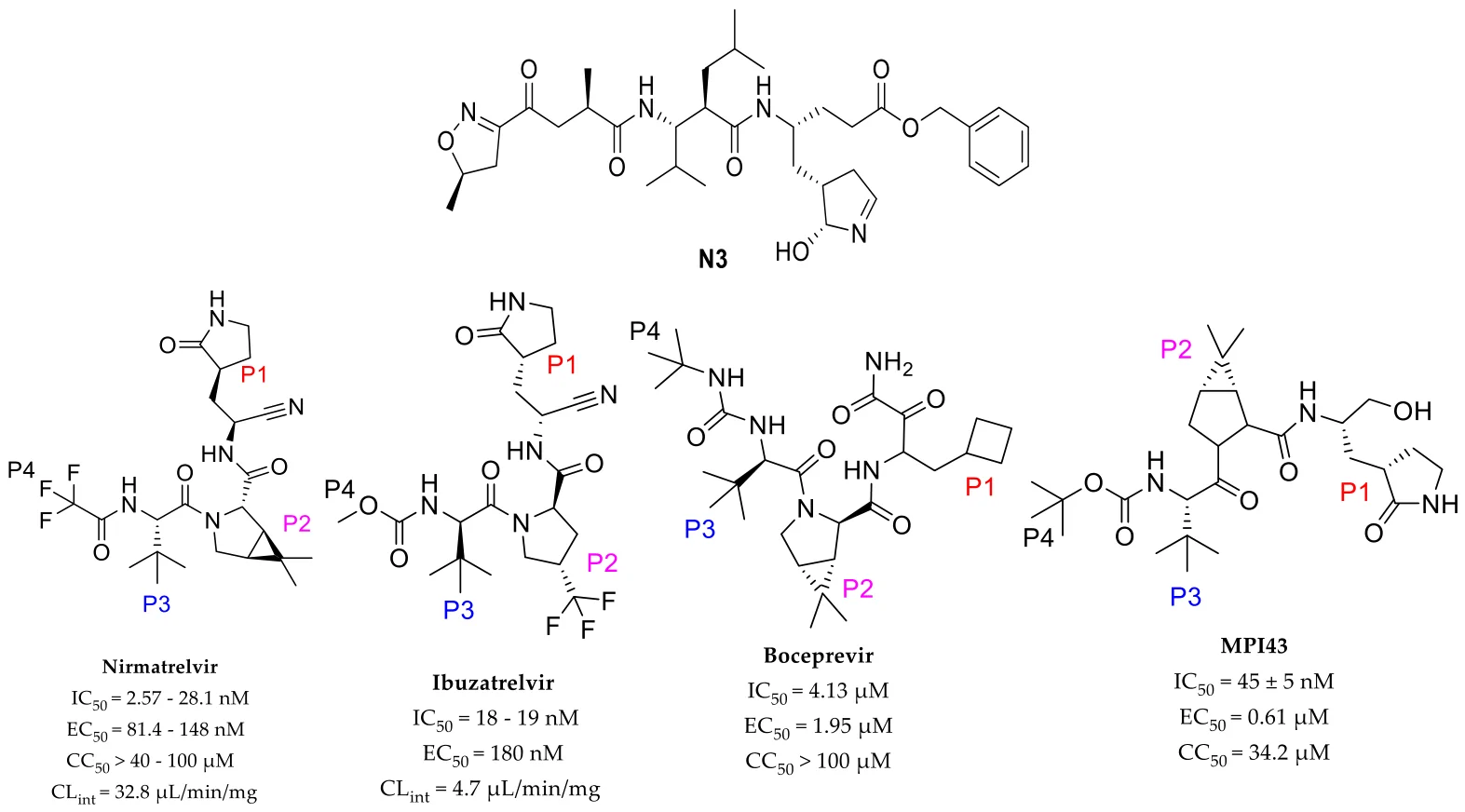
SARS-CoV-2 Mpro inhibitors share a similar mechanism of action to Nirmatrelvir.

In 2020, Jin et al. made a groundbreaking discovery with N3, the first publicly reported inhibitor targeting SARS-CoV-2 main protease using CADD. Interestingly, N3 binds to the substrate-binding pocket of Mpro, effectively blocking viral replication with an EC_50_ of 16.77 μM and CC_50_ > 133 μM, suggesting low cytotoxicity, making it a promising candidate with a favorable safety profile for further development [129]. Following this was the discovery of Nirmatrelvir, the active component of Paxlovid, which is a peptidomimetic inhibitor that binds covalently and reversibly to the catalytic cysteine (Cys145) of Mpro via its nitrile warhead. This forms strong hydrogen bonds with key residues such as Phe140, His163, Glu166, and Cys145, and engages in hydrophobic interactions with His41, contributing to its strong binding affinity and conformational stability, with a docking score of −8.3 kcal/mol [179]. However, as previously stated, Nirmatrelvir is co-administered with Ritonavir to boost its pharmacokinetics [142]. Chen et al. [142] and Allerton et al. [49] reported a second-generation phase III clinical candidate also developed by Pfizer (Ibuzatrelvir), sharing structural similarities with Nirmatrelvir but includes a trifluoromethyl proline at P2, which contributed to the reduction in metabolic stability with good intrinsic clearance (CL_int,app_), and a methyl carbamate at P4, which contributes positively inbinding affinity with a K_i_ value of 5 nM [49,142]. Ibizatrelvir (PF-07817883) inhibits Mpro through reversible covalent interaction like Nirmatrelvir and fits precisely into the S2 substrate pocket, forming a stable complex without requiring Ritonavir co-administration, but improved metabolic stability with a rate of 4.7 µL/min/mg using Human Liver Microsomes (HLM) [49,142]. Ibizatrelvir binds with MPro through a covalent inhibition of its nitrile warhead with Cys145, similarly to Nirmatrelvir. Resolved X-ray Crystal structures have revealed that Ibuzatrelvir maintains a strong hydrogen bond network with Glu166, Hie164, and Hie163, and hydrophobic contacts with amino acids such as Ala191, Leu16and Met165 in the active site cavity (Figure 14b). The experimental binding affinity was performed using a FRET-based assay and revealed an IC_50_ value as low as 19 nM, without any human off-target pharmacological activity in multiple in vitro assay panels (Figure 15) [142].

Boceprevir, originally developed for the hepatitis C virus, has a similar structure to Nirmatrelvir except for an α-ketoamide warhead forming a covalent adduct with Cys145, but is less stable than nitrile-based inhibitors; this is confirmed by a weaker binding affinity of −7.9 kcal/mol compared to Nirmatrelvir [202]. However, the presence of the β-cyclobu-tylalanyl moiety in P4, Dimethylcyclopropylproline (P2), and a *N*-terminal tert-butylcarbamide in P3 enhances cytotoxicity. Nonetheless, the repulsion with His4 reduces the overall potency [101,202]. Optimization of the P1 and warhead in boceprevir has led to enhanced potency, leading to the development of MPI43, an experimental compound featuring an aldehyde warhead. This structural refinement not only improved binding affinity to SARS-CoV-2 Mpro but also significantly reduced the CC_50_ value to 34.2 µM, indicating lower cytotoxicity and better therapeutic potential (Figure 15) [101].

**Figure 16:**
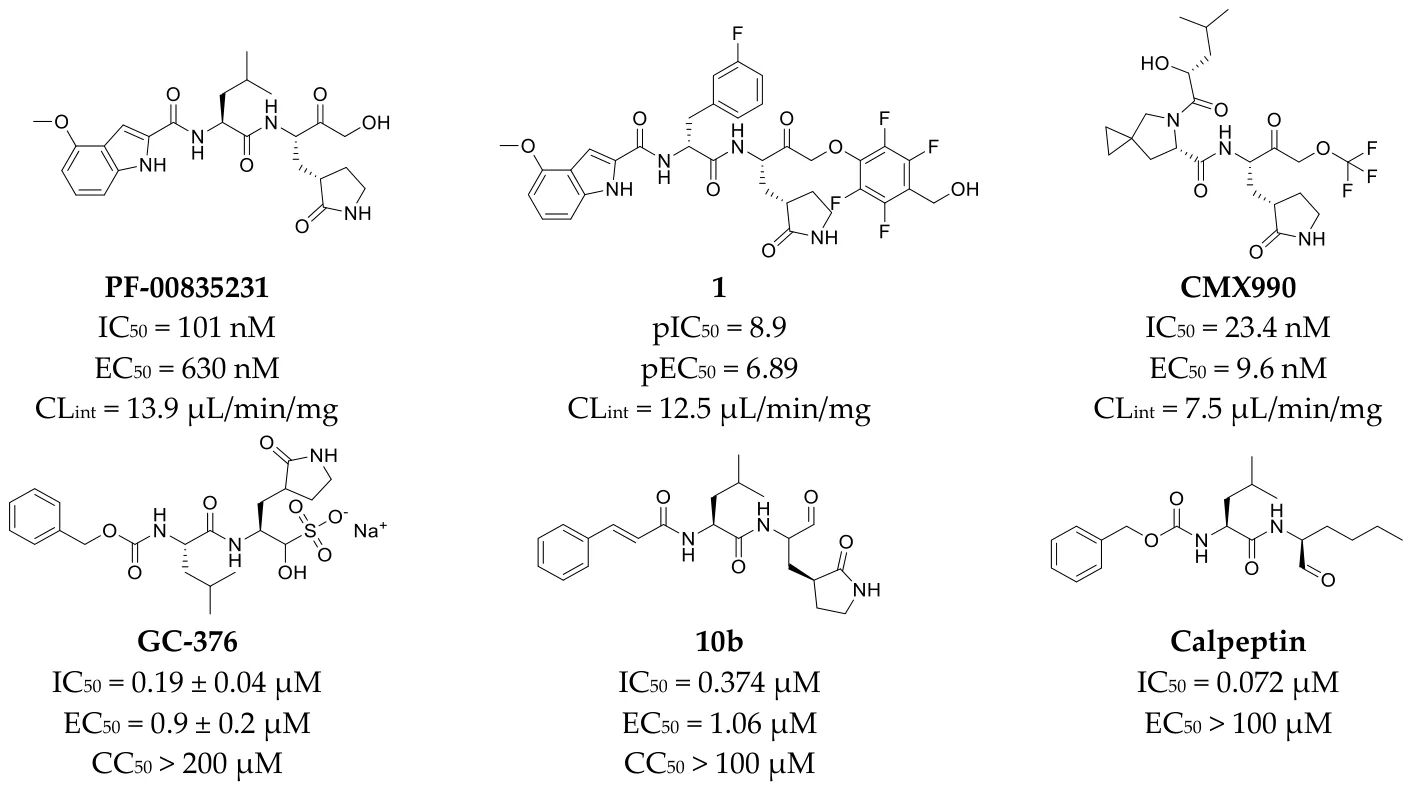
SARS-CoV-2 Mpro inhibitors share a similar mechanism of action as PF-00835231.

The PF-00835231 is an active form of Nirmatrelvir designed for the SARS-CoV outbreak as a covalent inhibitor targeting the SARS-CoV Mpro with a Hydroxymethylketone warhead [203]. Due to the high conservation of the SARS-CoV and SARS-CoV-2 proteases’ active sites, this compound was later adapted for the inhibition of SARS-CoV-2 [203]. This exhibited a very low intrinsic clearance in humans (HLM-CL_(int)_ = 13.9 μL/min/mg), with higher potency (EC_50_ = 81 nM, IC_50_ = 101 nM) against SARS-CoV-2 Mpro [51]. PF-00835231 offers a broad spectrum against numerous viruses including SARS-CoV-2 Mpro and its mutants such as G15S, Y54C, M49I, K90R, S46F, P132H, and V186F [204,205]. Compound 1, similar to PF-00835231 but bearing the tetrafluorophenoxymethyl ketone and a fluorobenzene instead of an isopropyl group, was found to be an unstable analog with moderate clearance CL_int_ = 12.5 μL/min/106 cells and a half-life (*t*1/2 = 55.5 min) in human hepatocytes, without co-administration with Ritonavir, and upon co-administration with Ritonavir, the intrinsic clearance improves to CL_int_ = 6.9 μL/min/106 cells and a half-life (*t*1/2 = 100.3 min); therefore, co-administration is advisable for this compound as Nirmatrelvir [141]. Replacement of the indole-substituted scaffold and restricted benzyl ring resulted in the loss of potency in compound **10**b, containing an aldehyde warhead and GC-376, a bisulfite adduct prodrug of GC-373; their warheads also contributed to their binding affinity (Figure 16).

The CMX990 is a phase III clinical candidate designed with the aim of improving both potency and human microsomal clearance of PF-00835231. CMX990, a trifluoromethoxymethyl ketone warhead, improved potency with an IC_50_ of 23.4 nM, EC_90_ of 10 nM, as the warhead plays a crucial role in the potency of covalent inhibitors interacting with Cys145. Further optimization of the P2 and P3 pockets also contributed to the improvement of efficacy, but mainly the intrinsic clearance of HLM-CL_(int)_ = 7.5 μL/min/mg, showing a very good correlation between the assays, with metabolic clearance five times better than that of Nirmatrelvir [51]. It is notable that the second-generation SARS-CoV-2 Mpro inhibitors not only offer improved metabolic stability but also offer less potential for drug–drug interaction and enhanced efficacy without the need for any pharmacokinetic booster. These compounds also contain a warhead for covalent interaction with the thiol of Mpro catalytic cysteine, as Nirmatrelvir, with some perturbation of certain molecules. From the selected experimental compounds inhibiting SARS-CoV-2 Mpro, we were able to conclude that Ibuzatrelvir, CMX990, compound **1**, and PF-00835231, covalent inhibitors binding reversibly to Cys 145 and a non-covalent inhibitor from COVID moonshot MAT-POS e194df51-1, were the most potent inhibitors. While acknowledging the drug-like nature of these inhibitors, metabolism remains the main challenge for experimental compounds, especially peptidomimetics.

Compounds that are considered peptidomimetics mimic natural proteins or peptides and retain the ability to interact with biological targets, producing the same biological interaction. Mpro inhibitors are dominated by peptidomimetics and even have an FDA-approved drug under this class of compounds (Nirmatrelvir). The synthetic procedure to prepare CMX990 is an example of the synthesis of peptidomimetic compounds. The method uses the commercially available reagents and forms peptide bonds that are essential to form these pharmacophores [206]. The synthesis for this clinical candidate began with the conversion of an ester to form the novel warhead under cryogenic conditions.

**Scheme 1:**
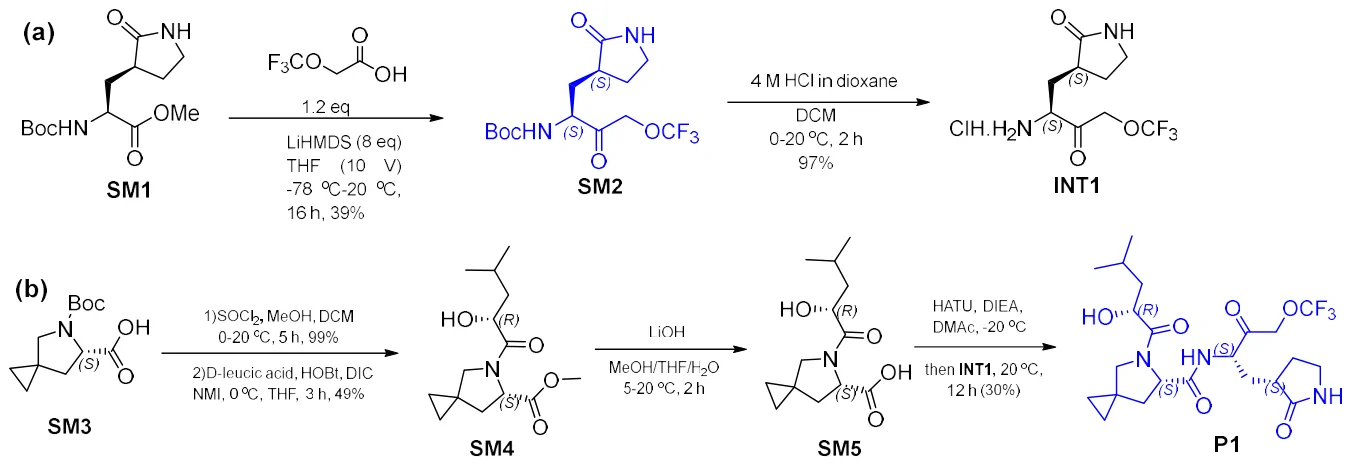
The synthetic procedure adopted for the synthesis of CMX990. (**a**) Trifluoroacetylation of Boc-protected intermediate SM1 to afford SM2, followed by Boc deprotection using HCl in dioxane to generate the corresponding amine intermediate. (**b**) SM3 was converted to carbonate intermediate SM4, which was subsequently reacted with (R)-2-methylbutylamine to yield carbamate SM5. Final HATU/DIPEA-mediated amide coupling between the two fragments afforded the target compound P1 [41].

This was followed by the deprotection of Boc with hydrochloric acid to afford the salt (Scheme 1a) [51]. The second linear four-step conversion started by manipulating the protecting group with a maximum yield of 99% and proceeded by coupling D-leucic acid with proline to give SM4 with moderate yield. The coupling product was subjected to basic conditions for the hydrolysis of the ester to carboxylic acid, affording an excellent yield of 93% of SM5. The intermediate obtained was formed through a conventional amide coupling with an amine salt, giving the product P1 as a solid (Scheme 1b) [51].

**Figure 17:**
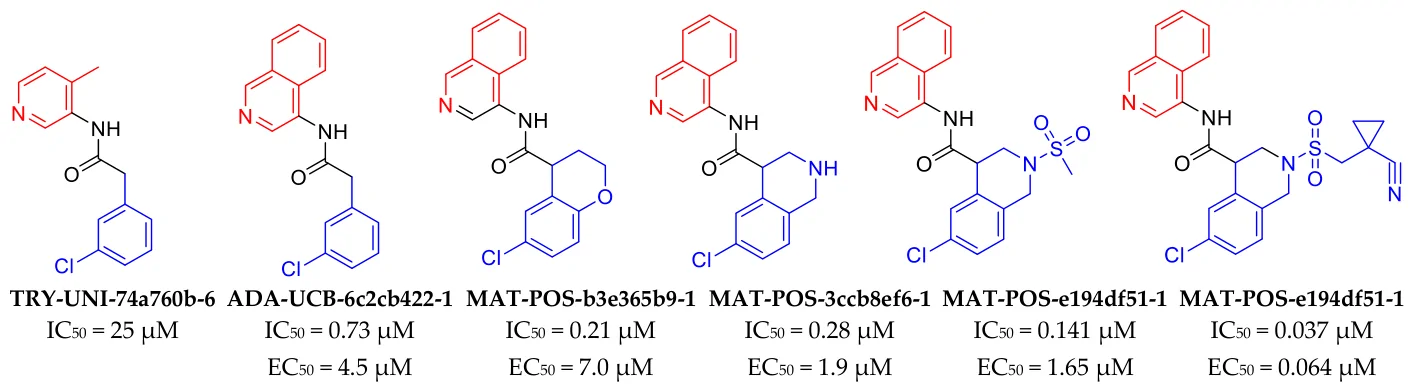
SAR analysis of non-covalent inhibitors targeting SARS-CoV-2 main protease (Mpro) [144].

COVID Moonshot, initiated in March 2020, represents a collaborative endeavor aimed at identifying novel molecules capable of inhibiting SARS-CoV-2 viral entry and replication. The primary focus of this initiative was the SARS-CoV-2 main protease (Mpro), utilizing crowdsourced molecular designs. This group employed computational approaches such as high-throughput screening, machine learning, and free-energy perturbation in accelerating the discovery of non-covalent Mpro inhibitors [144]. A resulting hit TRY-UNI-7149760b-6 is a 3-aminopyridine compound, demonstrating an IC_50_ of 25.0 µM. The exploitation of the P1 pocket through machine learning and free-energy perturbation to prioritize compounds for synthesis revealed a preference for scaffolds possessing an amino pyridine, which increased rigidity to facilitate hydrogen bond interactions. The substitution of pyridine from compound TRY-UNI-714a760b-6 with isoquinoline led to the development of ADA-UCB-6c2cb422-1, which exhibited a 34-fold improvement with an IC_50_ of 0.73 µM and additional hydrophobic interactions involving Asn142. The introduction of a tetrahydropyran ring into the P2 pocket, which is known to favor hydrophobic moieties, resulted in MAT-POS-b3e365b9-1, exhibiting an IC_50_ of 0.140 µM. However, this modification resulted in a reduction in antiviral activity in cellular assays, with the EC_50_ increasing from 4.5 µM to 7.0 µM. The subsequent incorporation of a tetrahydroisoquinoline moiety preserved the compound’s potency, and further substitution with a sulphonamide group enhanced both enzymatic inhibition and antiviral efficacy. This optimization process culminated in the identification of the lead compound MAT-POS e194df51-1, characterized by an IC_50_ of 0.037 µM and an EC_50_ of 0.064 µM, as depicted in Figure 17 [144].

**Scheme 2:**
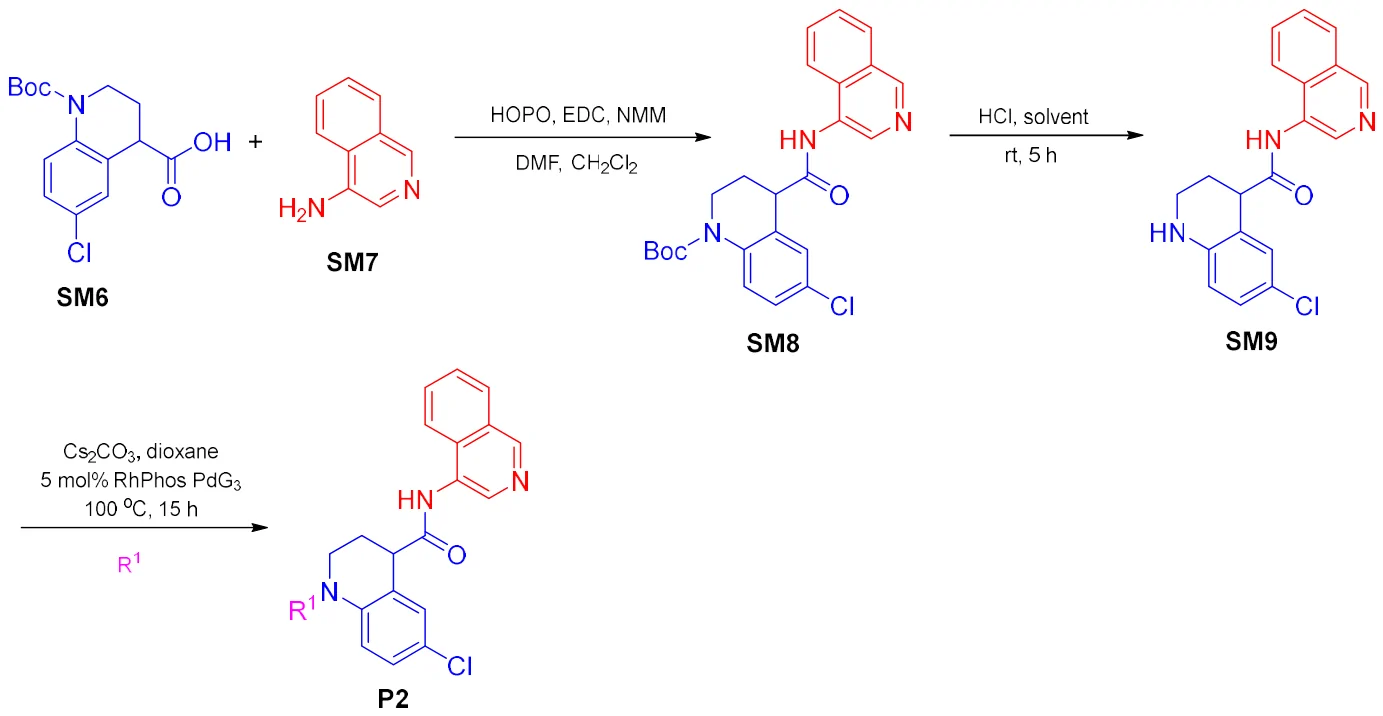
Synthesis of MAT-POS-e194df51-1 COVID moonshot non-covalent clinical candidate currently in Phase I clinical trials.

The synthetic Scheme 2 presented below shows the synthesis of the COVID Moonshot lead inhibitor targeting Mpro. The first step involves the coupling of a carboxylic acid with an amine to form an amide bond under mild reaction conditions. Followed by Boc deprotection under acidic conditions, allowing the secondary amine to functionalize via Buchwald reaction to afford a desired product (P2) (Scheme 2).

### 3.2. SARS-CoV-2 Papain-like Protease (PLpro): Structure and Function

**Figure 18:**
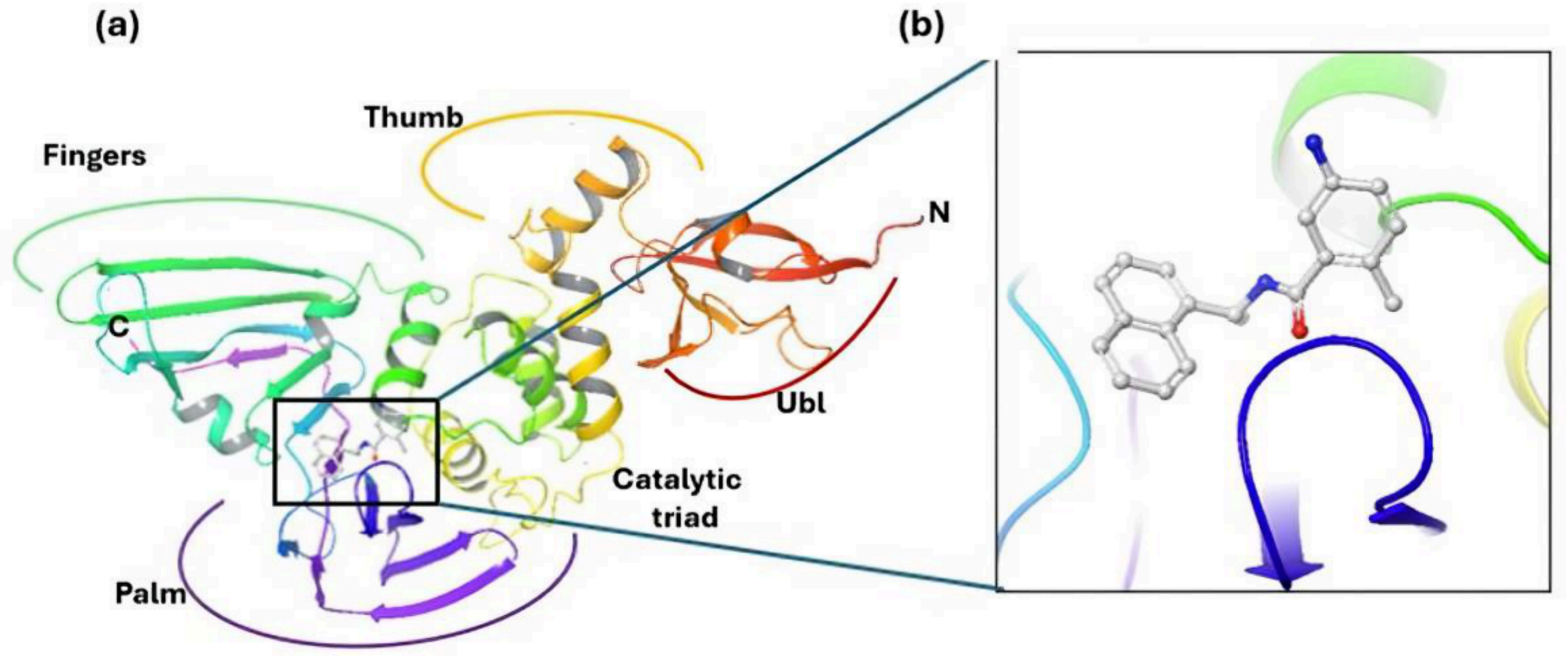
Structural organization of the SARS-CoV-2 papain-like protease (PLpro). (**a**) Ribbon representation of PLpro highlighting its subdomains: the ubiquitin-like (Ubl) domain (red/orange), thumb domain (yellow), fingers domain (green), and palm domain (magenta) with the catalytic triad located at the interface of the thumb and palm domains, and forms the enzyme’s active PDB ID: (7CMD). (**b**) A close-up view of the catalytic binding site with an inhibitor, GRL-0617, in the active site cavity. Reproduced with permission [208]. Copyright 2021 Springer Nature.

The papain-like protease (PLpro) is a non-structural protein (~212 kDa) containing 1945 residues responsible for viral replication and transcription; it cleaves the three peptide bonds to produce nonstructural proteins [207,208,209]. Its topological structure is divided (Figure 18) [207,210]. The finger sub-domain contains four conserved cysteines (Cys189 and Cys192 on the loop between b4e5, Cys 224 and Cys 226 on the loop between b6e7), forming a zinc finger which belongs to the “zinc ribbon” fold group [210]. The PLpro can suppress the viral spread and inflammation by deubiquitinating and deISG15ylating (interferon-induced gene 15) in the enzyme [13,210]. However, SARS-CoV PLpro prefers the ubiquitinated substrate, whereas SARS-CoV-2 PLpro prefers the ISGylated proteins [210]. PLpro’s dual functionality prevents virus replication and destroys its role in host immune response evasion, making it one of the most essential anti-SARS-CoV-2 drug targets [13,210]. This is regarded as the most challenging SARS-CoV-2 target, as the binding site contains Asp286, His272, and Cys111, which are located at the interface between the palm and thumb regions. In contrast, non-covalent inhibitors typically bind to nearby sites rather than the active site [211]. PLpro structural features are highly conserved in all coronaviruses, with approximately 82% sequence identity between SARS-CoV and SARS-CoV-2 papain-like proteases [210].

The GRL-0617 is a known SARS-CoV PLpro inhibitor with viral assay EC_50_ of 15 μM in Vero E6 cells, and an in vitro inhibition constant of K_i_ of 0.49 ± 0.08 µM, without any observed cytotoxicity effects [212]. Furthermore, GRL-0617 has been found to effectively inhibit SARS-CoV-2 PLpro, demonstrating its potential as a promising antiviral agent. Structural studies reveal that GRL-0617 binds to the active site of PLpro, interfering with the enzyme’s ability to cleave viral polyproteins and disrupt host immune responses [210], with an inhibition concentration of 1.39 µM [213] and relative binding free energy (ΔG) of −7.75 kcal/mol [13]. Despite its viral potency of 20 µM for viral replication, not sufficient for it to be a potential drug, GRL-0617, so far, remains the most optimized potential analog for the development of SARS-CoV-2 PLpro inhibitors with stronger binding affinity, selectivity, and better pharmacokinetic properties [13,33,88,214]. Several analogs of GRL-0617 address this issue by optimizing different regions and have managed to enhance the potency from μM to sub-μM and even nanomolar, inhibiting PLpro enzymatic activity (Table 3). Several analogs with moderate enzymatic and cellular activities have been reported. Notable examples with EC_50_ below 1 µM include CP7 [215], compound **19** [216], PF-07957472 [214], Jun12682 [217], Jun13296 [218], and GZNL-P36 [33]. A few other GRL-0617 analogs, such as XR8-24 [197], compound **2** [219], compound **39** [187], Jun11273 [217], and Jun9-75-4 [220], had EC_50_ values above 1 µM, hindering them from advancing to clinical trials; however, they are still below 10 µM, so they might serve as backbones for further optimization. Researchers are still exploring different sites of the PLpro, including the newly discovered site Valub; more discoveries are expected. Gumede, 2022 [13] designed GRL-0617 analogs using a Glide-based active learning approach followed by FEP+ to predict their relative binding affinities, with compound **45** being the best candidate, with a binding energy of ΔG = −7.28 ± 0.96 kcal/mol, comparable to that of GRL-0617 (ΔG = −7.75 ± 0.40 kcal/mol) [13]. Klem et al. [221] and Calleja et al. [27] also reported GRL-0617 analogs with IC_50_ values below 10 µM, rac5c with 0.81 µM, and compound **9** with 0.79 µM, respectively. Furthermore, Fu et al. reported a GRL-0617 analog, such as Compound **6**, with an IC_50_ exceeding 10 µM [211]. Stasiulewicz et al. screened a library of 15 million compounds using pharmacophore modeling, molecular docking, and MM-GB/SA to estimate their binding affinities. Compound **7** yielded the most active compound with 3 times better binding energy of ΔG = −24.54 kcal/mol than GRL-0617 and an acceptable IC_50_ value of 0.303 µM, also better than that of GRL-0617, making the compound a better backbone for PLpro inhibitors [13,222]. Sousa et al. reported a naphyride non-covalent inhibitor, Compound **85**, with an IC_50_ of 15.06 µM, with fivefold improvement in PLpro inhibition, and enhanced binding interactions compared to their parental compound **1** with an IC_50_ of 73.61 μM [223]. However, the optimization subsequently affected the metabolic stability; hence, further developments should focus on enhancing both the potency and maintaining or improving metabolic stability and solubility. Barder et al. reported the SAR results of WEHI-P8, a novel PLpro inhibitor aiming to alleviate long-COVID, with an IC_50_ of 12 nM and an EC_50_ of 0.36 µM [224]. Ma and co-workers further synthesized 12 analogs of Jun9-13-7 and Jun9-13-9 [88]. Biochemical assays revealed that these analogs inhibited SARS-CoV-2 PLpro at the submicromolar level. The most potent analogs in this series were Jun9-72-2 and Jun9-75-4, with an IC_50_ of 0.62 μM and 0.67 μM, and EC_50_ values of 6.62 μM and 7.88 μM, respectively [220]. Another study reported an analog of these compounds with an improved IC_50_ of 0.56 μM in the FRET assay [225]. While recognizing the important contributions of researchers exploring GRL-0617, it is essential to also highlight the role of drug repurposing, particularly in targeting such a complex system. Notable examples include Neobavasoflavone, which demonstrates a promising docking score of −4.59 kcal/mol against the PLpro target [206]. Other repurposed candidates, such as Tashinone IIA sulfonate, used for anti-inflammatory therapeutics and YM155, initially developed for cancer, exhibit acceptable inhibition concentration of 2.47 ± 0.46 µM and 1.65 ± 0.13 µM, respectively [213,226,227]. Most significantly, Acriflavine, used as an antiseptic or antibacterial agent, has the best nanomolar EC_50_ value of 0.64 nM and an IC_50_ of 1.46 µM against SARS-CoV-2 PLpro [228].

SARS-CoV-2 PLpro drug design has explored several computational approaches, including molecular docking, molecular dynamics (MD) simulations, MM-PBSA/MM-GBSA, free-energy perturbation (FEP), pharmacophore modeling, and WaterMap analysis, which have been extensively applied in the design and optimization of PLpro inhibitors such as GRL-0617 and its analogs. These methods have significantly contributed to understanding binding modes, estimating binding affinities, and guiding structure–activity relationship (SAR) optimization, as demonstrated by the identification of several potent submicromolar and nanomolar candidates. However, important limitations remain: docking relies on simplified scoring functions and often neglects protein flexibility and solvent effects [229], while MD and FEP, although more rigorous, are computationally expensive and sensitive to system setup and force-field parameters [138]; as such, FEP+ remains underreported. Additionally, methods such as pharmacophore modeling and virtual screening can introduce bias and may fail to identify novel scaffolds, while discrepancies between predicted binding affinity and experimental activity highlight challenges in accurately translating in silico findings into biological efficacy [229,230]. These limitations underscore the need for integrated, multi-method approaches that combine docking, MD simulations, free energy calculations [138], and data-driven methods such as machine learning [231,232] or AI-based docking [233] to balance computational efficiency with improved physical realism and predictive accuracy in PLpro inhibitor discovery.

**Table 3:** Pharmacological and physicochemical profiles of experimental inhibitors targeting SARS-CoV-2 PLpro.

No.	Name	IC50	Ki	Type of PLpro Assay	EC50	Type of Viral Assay	CC50	Metabolic Pro-Filing (CLint) (µl/min/mg)/µL/min/10 Cells	LogD	Approval Status	Design Strategy	References
1	GRL-0617	2.4 ± 0.2 µM	ND*	Enzymatic assay	27.6 ± 1.2 µM	Vero E6	>60 µM	ND*	ND*	Lead	Derivative of 7724772 with 1-naphthyl moiety and an amino group in the methylbenzene moiety	[234]
2	4(PF-07957472)	28 µM	2 nM	Enzymatic assay	147 nM	Vero E6	69.3 µM	8.84	2.5	Experimental	N-methyl pyrazole derivative	[214]
3	JUN12682	106.8 ± 5.0 nM	37.7 nM	FRET enzymatic assay	0.42 µM	Vero E6	61.3 ± 4.5	10.5	ND*	Experimental	Pyrazole-containing	[217]
4	10 (XR8-23)	0.39 µM	ND*	Enzymatic assay	2.5 ± 0.15 µM	A549-hACE 2	43 ± 1.8 µM	ND*	ND*	Experimental	Aminoazetidine-substituted phenyl-thiophenes	[235]
	XR8-23 (72)	0.39 µM	ND*	Enzymatic assays	1.4 ± 0.1 µM	A549-hACE2	21.6 ± 1.1 µM	5.89	ND*	Experimental	Optimization of methyl, naphthalene, and aniline groups from GRL-617	[197]
5	XR8-24 (73)	0.56 µM	ND*	Enzymatic assays	1.2 ± 0.2 µM	A549-hACE 2	>50 µM	9.60	ND*	Experimental	Optimization of methyl, naphthalene, and aniline groups from GRL-0617	[197]
6	F0213	7.4 µM	ND*	HTS fluo-rescence-based as-say	4.549 µM	Vero E6-TMPR SS2 cells	ND*	ND*	ND*	Experimental	HTS lead	[236]
7	Compound **6**	5 ± 1.9 µM	ND*	Enzymatic assay	21 ± 1.9 µM	Vero E6	>60 µM	ND*	ND*	Experimental	Lead containing 1-naphthyl and acetyl amino moieties	[234]
8	YM155	2.47 ± 0.46 µM	ND*	HTS fluorescence-based enzymatic assay	0.17 ± 0.22 µM	Vero E6	~400 µM	ND*	ND*	Experimental	HTS of a library containing diverse compounds	[213]
9	Rac5c	0.81 µM	ND*	HTS fluorescence-based enzymatic assay	ND*	ND*	ND*	ND*	ND*	Experimental	Repurposing of known drugs and clinical candidates	[237]
10	Tropifexor	5.11 µM	ND*	Enzymatic assay	4.03 ± 0.5 µM	Calu-3 cells	26.6 ± 1.4 µM	ND*	ND*	Experimental	HTS screening of a MedChemExpress bioactive compound library	[238]
11	Proantho-cyanidin	2.4 ± 0.2 µM	ND*	HTS fluorescence-based enzymatic Assay	2.5 ± 0.4 µM	Vero E6	56.4 ± 0.7 µM	ND*	ND*	Experimental	Screening of FDA-approved drugs	[239]
12	Compound **7**	6.0 µM	ND*	Fluorescence polarization assay	4.59 ± 1.22 µM	Vero E6	>800 µM	ND*	ND*	Experimental	Chloroxine derivative	[240]
13	7	12.7 ± 1.3 µM	ND*	Biochemical assay	5.2 ± 4.2 µM	Vero E6	ND*	ND*	ND*	Experimental	Amine-functionalized derivatives of GRL-0617	[208]
14	1d	236 ± 107 nM	ND*	Biochemical assay	ND*	ND*	ND*	ND*	ND*	Experimental	Optimization of Ebselen’s derivatives substituted on the phenyl ring	[241]
15	1e	256 ± 35 nM	ND*	Biochemical assay	ND*	ND*	ND*	ND*	ND*	Experimental	Optimization of Ebselen’s derivatives substituted on the phenyl ring	[241]
16	19	0.44 ± 0.05 µM	ND*	Enzymatic assay	ND*	hACE 2-HeLa	>10 µM	ND*	ND*	Experimental	HTS of a library containing diverse compounds	[216]
17	Jun9-72-2	0.67 ± 0.08 µM	ND*	FRET-based assay	6.62 ± 1.31 µM	Vero E6	>60 µM	ND*	ND*	Lead	Optimization of hits screened from an ENAMINE hit collection	[220]
18	Jun9-75-4	0.62 ± 0.06 µM	ND*	FRET-based assay	7.88 ± 1.44 µM	Vero E6	47.48 ± 14.63 µM	ND*	ND*	Lead	Optimization of hits screened from an ENAMINE hit collection	[220]
19	7	303 nM	ND*	Predicted	ND*	ND*	ND*	ND*	ND*	Experimental	Screening of ENAMINE real database using structure-based approaches	[222]
20	Acriflavine	1.66 µM	ND*	HTS FRET-based assay	ND*	ND*	ND*	ND*	ND*	Experimental	HTS of a library containing diverse clinical compounds	[228]
21	85	15.06 ± 2.19 µM	ND*	In vitro enzymatic assay	ND*	Vero	7.47 µM	ND*	ND*	Experimental	Retention of naphthyridine core from a HTS hit compound **1**	[223]
22	7	0.094 µM	ND*	Fluorogenic peptide assay	1.1 µM	ND*	ND*	38.9	ND*	Experimental	GRL-0617 designed by incorporating N,N’-acetylacetohydrazine linker instead of the methyl group of aminobenzene moiety	[215]
23	ZN-3-80 (65)	0.59 µM	ND*	Enzymatic assay	ND*	ND*	ND*	5.64	ND*	Experimental	Optimization of methyl, naphthalene, and aniline groups from GRL-0617	[197]
26	SIMR3030	0.0933 ± 0.1895 nM	ND*	Enzymatic assays	ND*	Vero E6	130.11 µM	ND*	ND*	Experimental	In vitro screening of an in-house library of drug-like molecules	[242]
27	Jun-22	0.67 ± 0.08 µM	ND*	ND*	ND*	ND*	23.69 ± 1.58 µM	778.8	ND*	Lead	Optimization of GRL-0617 by incorporating a quinoline moiety instead of naphthalene	[218]
28	Jun-51	0.66 ± 0.12 µM	ND*	ND*	4.11 ± 0.64 µM	Caco-2 cells	35.47 ± 1.52 µM	429.1	ND*	Lead	Optimization of GRL-0617 by incorporating a quinoline moiety instead of naphthalene	[218]
29	Jun11213	0.69 µM	ND*	ND*	5.30 ± 1.26 µM	Caco-2 cells	ND*	25.7	ND*	Lead	Optimization of GRL-0617 by incorporating a quinoline moiety instead of naphthalene	[218]
30	Jun11165	0.63 µM	ND*	ND*	6.04 ± 0.84 µM	Caco-2 cells	ND*	11.8	ND*	Lead	Optimization of GRL-0617 by incorporating a quinoline moiety instead of naphthalene	[218]
31	Jun11243	0.50 ± 0.10 µM	ND*	ND*	ND*	Caco-2 cells	15.20 ± 0.30 µM	<9.6	ND*	Lead	Optimization of GRL-0617 by incorporating a quinoline moiety instead of naphthalene	[218]
32	Jun10725	0.58 ± 0.08 µM	ND*	ND*	ND*	Vero E6	31.5 ± 2.70 µM	ND*	ND*	Lead	Optimization of GRL-0617 by incorporating a quinoline moiety instead of naphthalene	[218]
33	Jun-43	0.67 ± 0.08 µM	ND*	ND*	ND*	Vero E6 cell	24.69 ± 1.3 µM	ND*	ND*	Experimental	Optimization of GRL-0617 by incorporating a quinoline moiety instead of naphthalene	[218]
34	Jun11924	177.7 ± 10.0 nM	ND*	FRET-based enzymatic assay	ND*	ND*	36.4 ± 1.3 µM	ND*	ND*	Experimental	Rational design of biarylphenyl benzamide scaffolds	[217]
35	Jun12162	98.3 ± 7.0 nM	33.6 ± 3.0 nM	FRET-based enzymatic assay	0.23 µM	Vero E6	42.3 ± 2.3 µM	ND*	ND*	Hit	Rational design of biarylphenyl benzamide scaffolds	[217]
36	Jun12682	106.8 ± 5.0 nM	37.7 ± 3.0 nM	FRET-based enzymatic assay	0.42 µM	Vero E6	61.3 ± 4.5 µM	ND*	ND*	Experimental	Rational design of biarylphenyl benzamide scaffolds	[217]
37	39	0.078 ± 0.014 µM	ND*	Enzymatic assay	2.41 ± 0.12 µM	Cell-based assay (FlipG FP)	12.91 ± 3.55 µM	105 mL/min/gprot	ND*	Experimental	Optimization of HTS hit A0 using rational drug design	[187]
38	45	0.246 ± 0.044 µM	ND*	Enzymatic assay	3.13 ± 0.75 µM	Cell-based assay (FlipG FP)	>40 µM	2503 mL/min/gprot	ND*	Experimental	Optimization of HTS hit A0 using rational drug design	[187]
39	Jun9-75-5	0.56 µM	ND*	FRET assay	ND*	ND*	ND*	ND*	ND*	Experimental	Design of N-methyl-1-(naphthalen-1-yl)ethan-1-amine analogs using QSAR approaches	[225]
40	Compound **2**	0.39 µM	ND*	Enzymatic assay	3.61 ± 0.52 µM	FlipGF P as-say/C Ck8 assay	~179.9 µM	ND*	ND*	Experimental	Structure-based design of covalent peptidomimetics	[219]
41	Compound **6**	5.0 ± 1.9 µM	ND*	Enzymatic assay	21.0 ± 1.9 µM	Vero-E6	ND*	ND*	ND*	Experimental	Derivatives of original SARS-CoV-1 PLpro inhibitors	[234]
42	GNZL-P36	0.00645 µM	Vero E6	Enzymatic assay	111.0 ± 9.95	ND*	888.41 µM	ND*	ND*	Experimental	Rational drug design of GRL-0617 analogs by incorporating the cyclopropyl linker, and 1,2-dihydroace-naphthylene instead of naphthalene ring	[33]
43	WEHI-P8	12 nM	ND*	Enzymatic Assay	0.36 µM	FRET assay	ND*	ND*	ND*	Experimental	HTS screening of methoxynaphthalene-containing analogs	[27]
44	PR-619	1.39 µM	ND*	Enzymatic assay	ND*	ND*	ND*	ND*	ND*	Lead	Biochemical HTS screening of oxi-doreduction-sensitive compounds	[243]

ND*—Means not determined.

**Figure 19:**
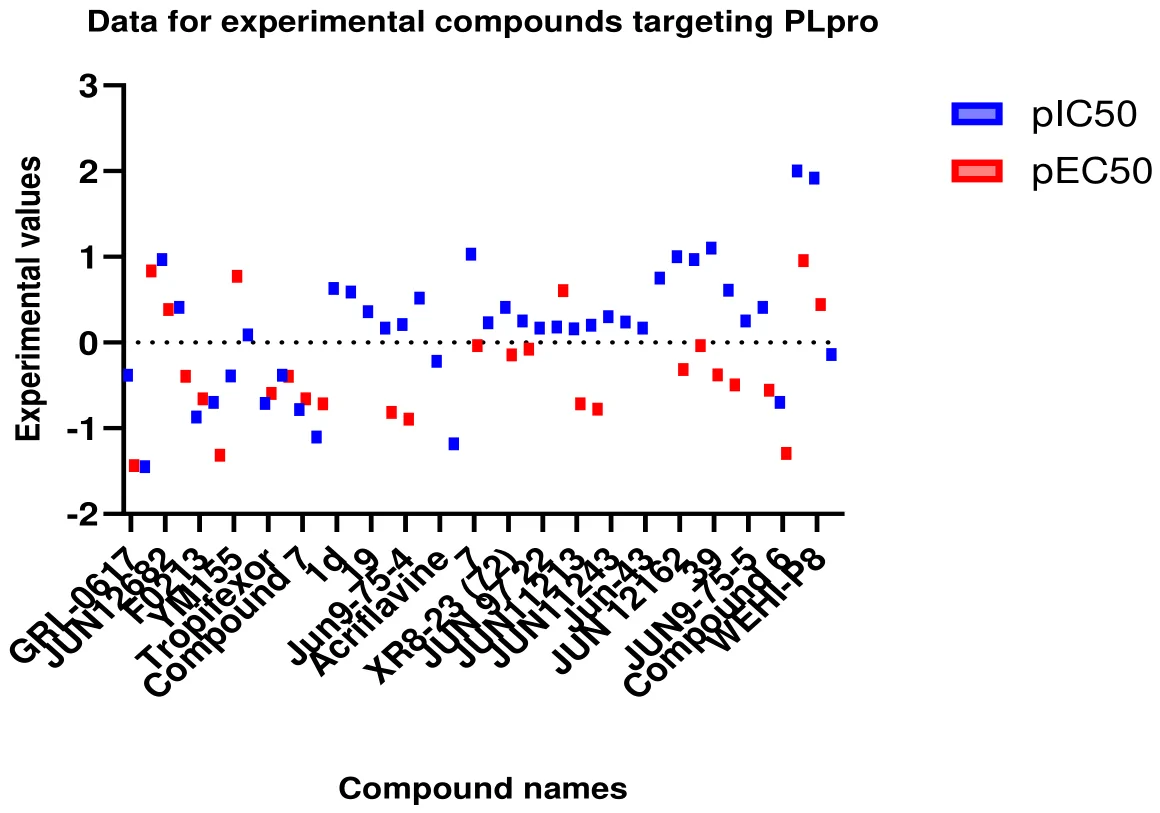
Comparative analysis of bioactivity, viral activity, metabolic stability, and solubility of compounds targeting PLpro reported in the literature.

Many GRL-0617 derivatives have improved inhibition activity, including JUN-12162 and JUN-12682, as shown in Figure 19, which follows, even though they demonstrated negative pEC_50_ values in the viral assay. As expected, there is no correlation between pIC_50_ and pEC_50_, which is a hallmark for compounds designed against PLpro. As such, most pIC_50_ values range between 1 and −1, whereas pE_C50_ values range between 0 and −1. However, compound WEHI-P8 is an outlier as it is most potent with a pIC_50_ value of 1.92 and a pEC_50_ value of 0.44. Notably, WEHI-P8 is a derivative of GNZL-P36, which is also among the most potent, with a pIC_50_ value of 2.0 and a pEC_50_ value of 0.95. On the other hand, some derivatives of GRL-0617, such as compound **4** (PF-07957472), compound **6**, and compound **85**, had their pIC_50_ values in the negative region. The scatter plot (Figure 19) comparing pEC_50_ and pIC_50_ values reveals a distinct difference in the potency profiles of the compounds tested. The pEC_50_ values are mainly clustered around zero or negative, indicating that most compounds showed poor efficacy in various assays. Only about six compounds (PF-07957472, YM155, JUN9851, GNZL-P36, WEHI-P8, and JUN12682) exhibited positive pEC_50_ values, suggesting that they had meaningful biological activity in specific assays. In contrast, the pIC_50_ values are generally more positive, with a wider distribution and a higher median, implying that many compounds demonstrated good inhibitory activity. The data in Figure 19 represent a similar trend to the data in Figure S2 of the supporting document, which includes all PLpro inhibitors. One of the main challenges in improving the effectiveness of PLpro inhibitors is the restricted accessibility of the S2 and S1 substrate-binding pockets, which strongly prefer a Gly–Gly motif, hence, the delays in the advancement of PLpro inhibitors [28].

### 3.3. Quantitative Structure–Activity Relationship (QSAR) of GRL-0617 Analogs

**Figure 20:**
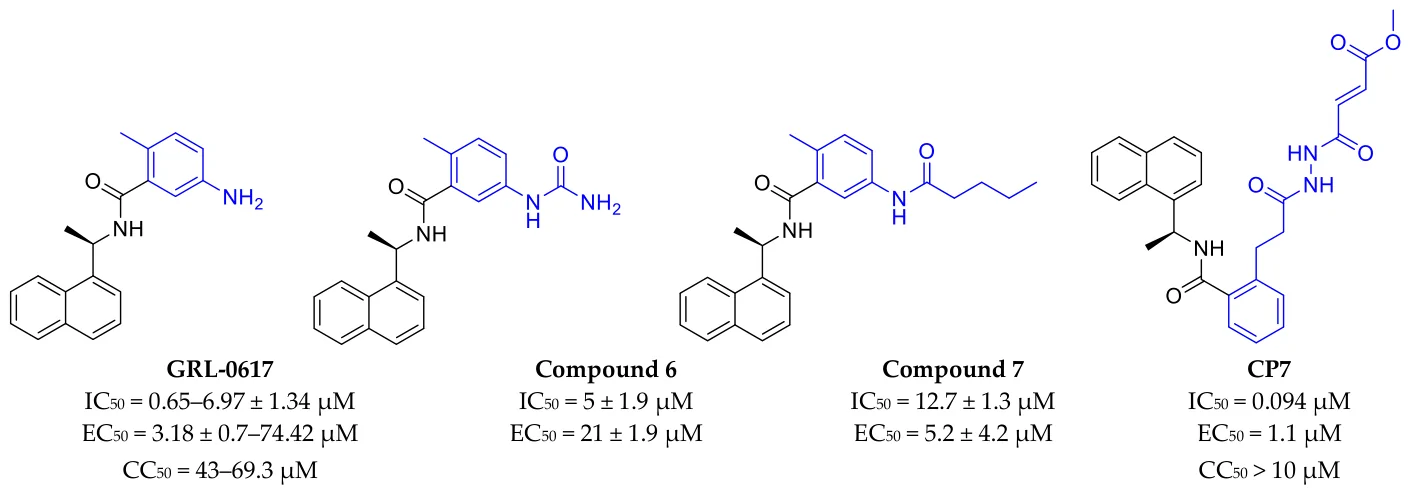
Chemical structures and biological activities of GRL-0617 and its selected naphthalene-containing analogs: compound **6**, compound **7**, and CP7.

While other PLpro inhibitors feature diverse scaffolds derived from various sources, GRL-0617 stands out as the most studied analog. GRL-0617 remains the most optimized analog for the development of SARS-CoV-2 PLpro inhibitors with stronger binding affinity, selectivity, and better pharmacokinetic properties [3,214]. GRL-0617 forms hydrophobic interactions with residues such as Tyr268, Pro247, and Leu162, forming π–π stacking interactions between the naphthalene ring of GRL-0617 stacks, with Tyr268, stabilizing the binding and hydrogen bonding between the NH group and the carbonyl group of GRL-0617 and the amino acids such as Gln269 and Asp164 for additional stabilization (Table 4) [28]. Retaining the naphthalene moiety and extending the amine side chain of GRL-0617 led to a remarkable improvement in potency, as shown in Figure 20. The inclusion of a withdrawing group within the linker, as seen in compound **6** and CP7, contributed positively to improving the potency; however, cytotoxicity is still a concern. It is important to highlight the importance of linker flexibility and polarity in enhancing biological activity.

**Figure 21:**
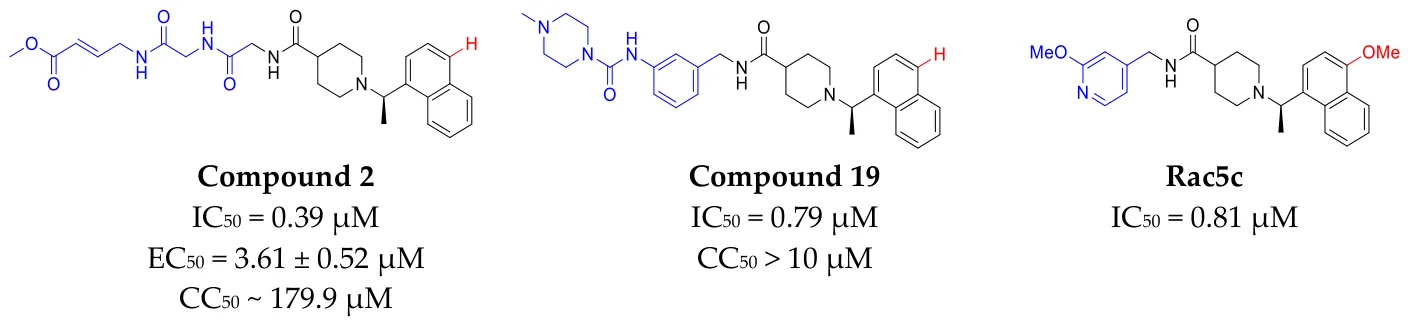
Chemical structures and biological activities of a piperidine-containing GRL-0617 analogs: compound **2**, compound **19**, and Rac5c.

Incorporation of a pyridine ring in place of the amine group near the naphthalene scaffold contributed positively to enhancing potency. Notably, covalent peptidomimetic inhibitor compound **2** showed beneficial effects by improving both potency and cytotoxicity. This forms a covalent binding to the catalytic residue Cys111 and promotes interaction with Try268, which accounts for the sub-µM inhibition [219]. Shan et al. reported another piperidine-containing pool of compounds with 19 as a lead. This contains a substituted piperazine group that interacts with the side chains of Tyr269, respectively, mainly through van der Waals forces [216]. The Rac5c compound also contains a bulkier moiety in R1, compound **19**, and has a lower IC_50_ compared to the peptidomimetic moiety inhibitor, compound **2**, accounting for the lack of accessibility of certain pockets [237] (Figure 21).

The PF-07957472, developed by Pfizer, is a machine learning-designed PLpro inhibitor with strong antiviral potency (EC_50_ of 147 nM) and oral bioavailability, showing promising results in pre-clinical COVID-19 models. This compound binds to the SARS-CoV-2 PLpro at the BL2 loop pocket, like GRL-0617, but features structural enhancements that improve its potency; its *N*-methyl pyrazole-quinoline moiety offers better hydrophobic coverage and stronger π-stacking interactions with Tyr268. The protonated piperazinyl group forms a salt bridge interaction with Glu167, distinct from GRL-0617’s distant amine. Additionally, the cyclopropyl group enables CH-π interactions with Tyr264 and contacts polar residues like Thr301 and Asp164, interactions absent in GRL-0617. These optimized scaffolds contributed positively to the superior inhibitory activity offered by this compound [214]. The literature highlights that the lack of potency in PLpro inhibitors is mainly due to the inaccessibility of the P1 and P2 sites recognizing glycine. A high-throughput screening (HTS) and QSAR by Shen et al. was conducted to overcome this challenge, leading to the discovery of XR8-24, a 2-phenylthiophene group substituted with an azetidine ring, enhancing electrostatic stabilization near Glu167, and the benzamide moiety that maintains hydrogen bond interaction with Gln269. A shift in the biaryl ring positions the thiophene into the new active site Valub, promotes van der Waals interaction, while the alicyclic tail forms an additional interaction near Pro248/Pro299 through a water-mediated hydrogen bond with Tyr264. This allows XR8-24 to make extensive contacts with the BL2 groove, accounting for the increased potency to IC_50_ of 0.56 μM and an EC_50_ of 1.2 μM [197]. Tan et al. reported a potent PLpro inhibitor Jun12682 from 85 noncovalent PLpro inhibitors binding to the recently explored ubiquitin binding site and the known BL2 groove with an enzymatic activity IC_50_ of 106.8 nM. This also showed good antiviral activity, with an EC_50_ ranging between 0.44 and 2.02 μM, against different SARS-CoV-2 variants [217]. Tan et al. achieved this by incorporating aryl substitutions at the third and fifth positions of the phenylethylamine ring to occupy hydrophobic interactions with residues in the two sites.

Amines on the meta-position of the benzoic acid were maintained to engage electrostatic interactions with Glu167, and the 1-phenylethyl benzamide core structure was kept, maintaining the hydrogen bonding and π-π interactions with the BL2 loop [217]. Lu et al. also substituted the naphthalene moiety by introducing a tricyclic (1,2-dihydroacenaphthylene) group, a bulkier substituent that promotes interaction between GZNL-P36 and residue Pro247. This modification shares similarity with the design of Jun12682, which extends the interaction in the BL2 groove with an aryl substitution. GZNL-P36 showed a low nanomolar IC_50_ of 6.45 nM for PLpro inhibition and broad-spectrum antiviral activity, with EC_50_ values ranging from 58.2 nM to 2.66 μM against SARS-CoV-2 variants and other human coronaviruses [220].

**Figure 22:**
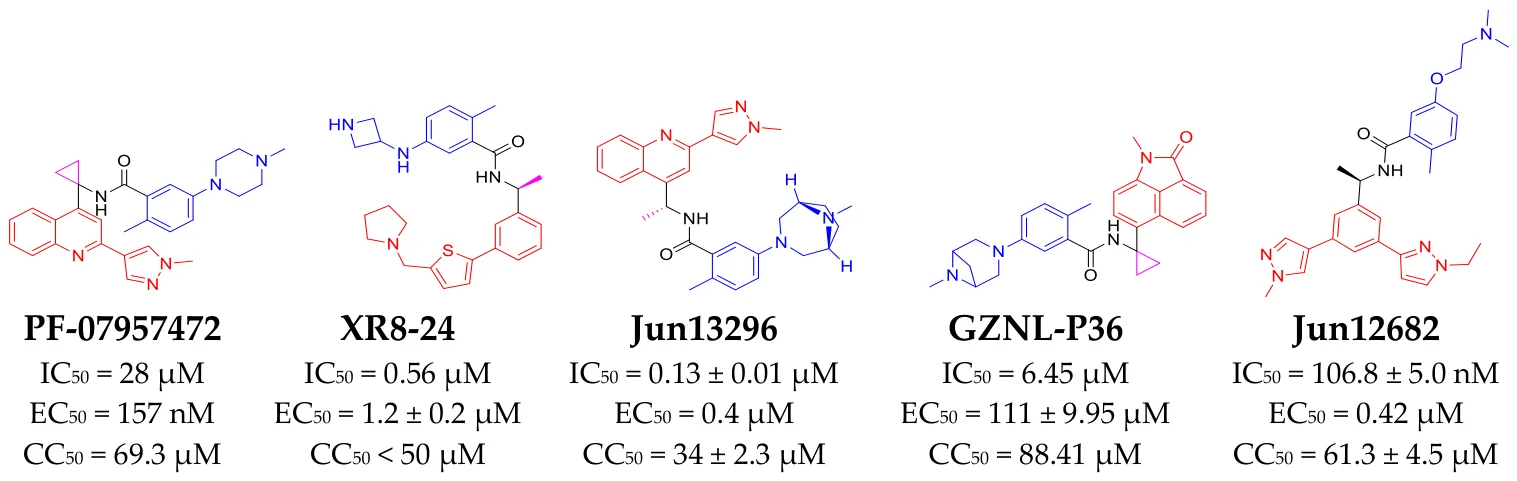
Chemical structures and biological activities of GRL-0617 analogs optimized at R^1^ (red), R^2^ (pink), and R^3^ (blue) positions.

Jadhav et al. reported quinoline-based inhibitors, including the lead compound Jun13296, similar to the recently reported clinical candidate PF-07957472 by Pfizer, which contains a quinoline scaffold in the R3 position, thereby enhancing binding affinity within specific binding pockets. Notably, the pyrazole substitution on the quinoline ring was tailored to interact with the Val70Ub site, mimicking the natural binding of ubiquitin, while the quinoline core caters for stable hydrogen bonding, contributing to strong and selective binding (Table 4). In addition to potent antiviral activity and efficacy against multiple SARS-CoV-2 variants, the Pfizer compound offers favorable pharmacokinetics, with CL_int(liver)_ = 16.9 mL/min/kg in humans (Figure 22) [218]. GZNL-P36 is one of the most potent compounds compared to PF-07957472; however, its CC_50_ value is unfavorable, as it is 13-fold higher than that of the Pfizer clinical candidate.

**Figure 23:**
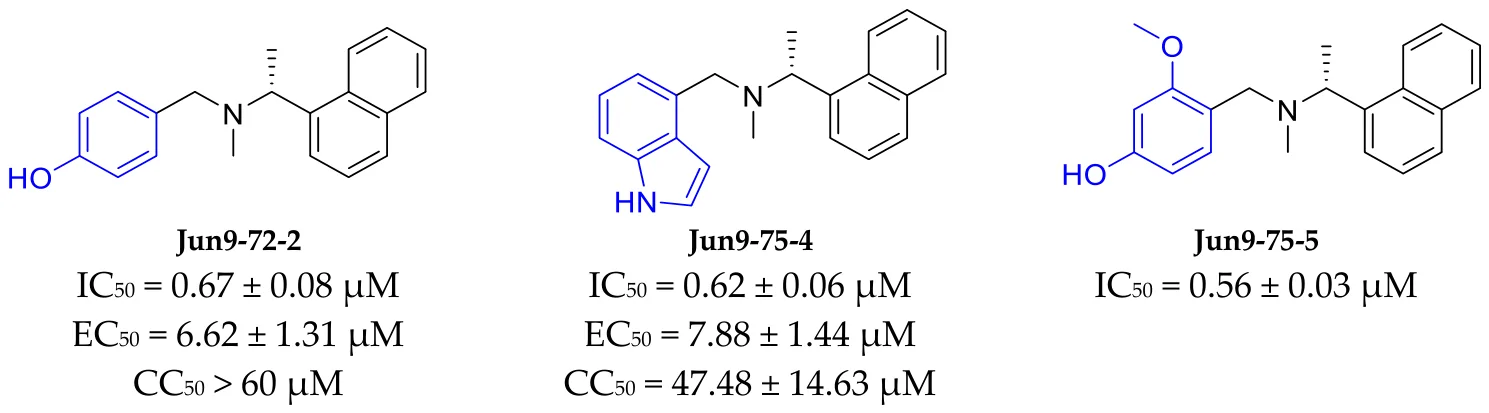
Trialkyl ammonium containing GRL-0617 analogs Jun9-72-2, Jun9-75-4 [219], and Jun9-75-5 [225].

Ma et al. [220] and Wang [225] did an optimization of GRL-0617 by replacing the carboxamide group with trialkyl ammonium for the design of new analogs such as Jun9-72-2, Jun9-75-4, and Jun9-75-5. The side chain of Asp164 formed a hydrogen bond with the N-H+ group, allowing it to occupy the receptor binding area [220]. Jun9-72-2 contains a benzophenol moiety, forming hydrogen bonding with Leu162. The replacement of benzophenol with an indole ring in Jun9-75-4 increased hydrogen bonding interactions with Gln269. A significant improvement was observed when a methoxy group was introduced into a benzophenol, with IC_50_ values of 0.67 μM, 0.62 μM, and 0.56 μM, respectively (Figure 23).

**Scheme 3:**
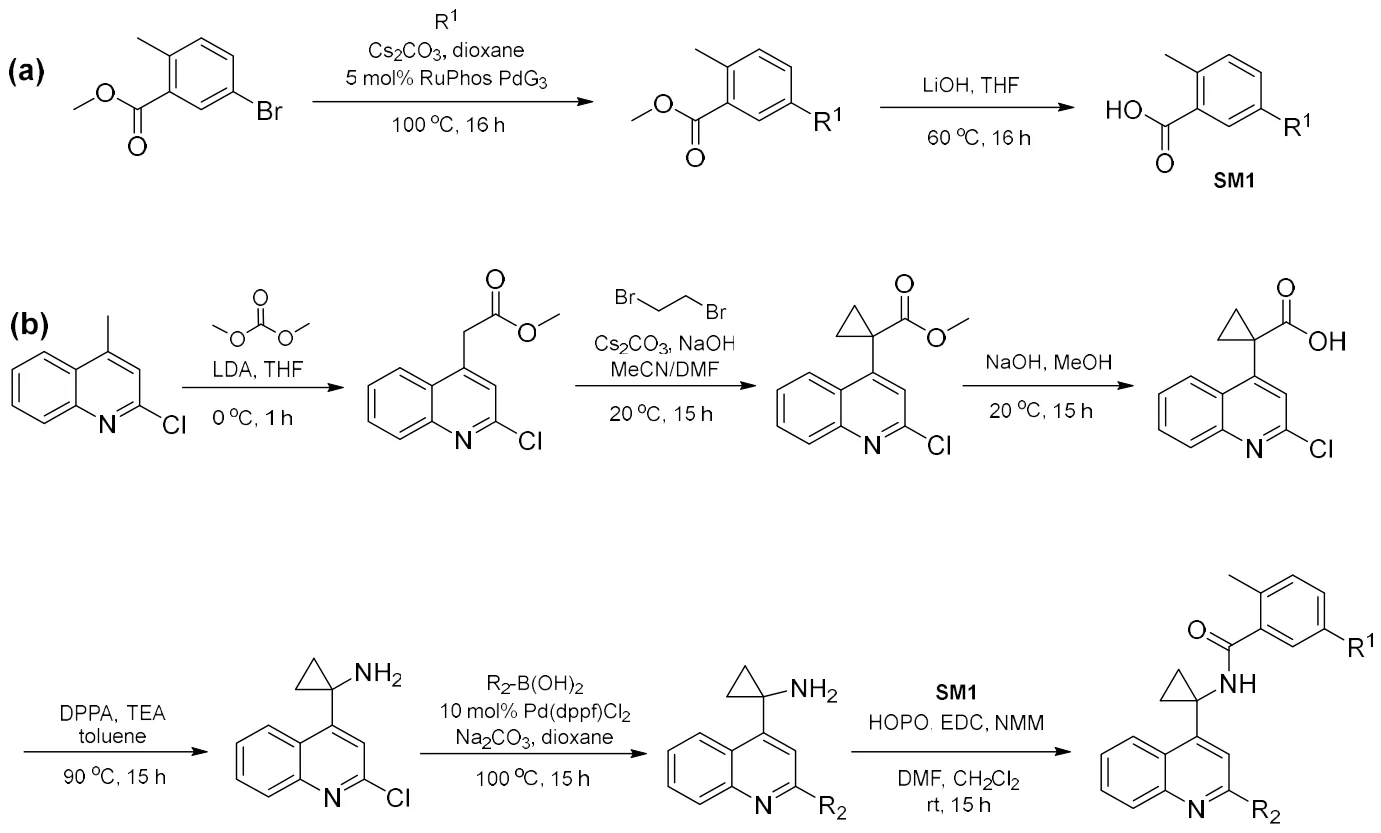
The synthetic scheme for the multistep synthesis of PF-07957472 and its derivatives, including their reaction conditions. (**a**) Buchwald-Hartwig amination of methyl 5-bromo-2-methylbenzoate with substituted amines (R^1^) using RuPhos Pd G3 as catalyst, followed by ester hydrolysis to afford the corresponding benzoic acid intermediate (SM1). (**b**) Multi-step synthesis of cyclopropyl-substituted quinoline intermediates involving esterification, cyclopropylation, Curtius rearrangement, Suzuki-Miyaura cross-coupling, and final amide coupling with SM1 to furnish the target compounds.

The illustrated synthetic Scheme 3 was reported by Garney et al. for the first time in 2024 [214]. Part A shows the Buchwald coupling from the commercially available methyl-5-bromo-2-methylbenzoate (SM10), facilitated by the palladium catalyst, followed by the hydrolysis of the ester to a carboxylic acid, which affords the first intermediate (INT1). Following part B of the procedure, the insertion of the ester into the ring was achieved under basic conditions, then the cyclization at the alpha carbon, followed by the hydrolysis of the ester under strong basic conditions, giving a carboxylic acid intermediate. An amine insertion, Suzuki coupling, and amide coupling were achieved to afford the desired product (Scheme 3).

## 4. Conclusions and Future Prospects

To design more lead compounds targeting Mpro and PLpro, a drug target for SARS-CoV-2, we propose the use of Ligand Designer, a molecular modeling tool that enables the enumeration of R-groups and bioisosteres within the core structure for Multiparameter lead optimization. The idea is to design new compounds with physicochemical properties that fall within the optimal range defined by Lipinski’s Rule of Five. These include a molecular weight < 500 Da, logP between 0 and 5.0, ≤5 HBD, and ≤10 HBA. Optimization strategies may involve structural modifications such as the replacement of sugar moieties, enumeration of R-groups, or alterations to the core scaffold using bioisostere replacement groups. These approaches would enhance drug-likeness while retaining or improving biological activity. Importantly, such modifications should preserve the integrity of the heterocyclic-containing core structure to maintain favorable physicochemical properties. This highlights the importance of Computational Chemistry in drug design, as molecular docking, MD simulations, MM-GB/SA, FEP+, reaction-based enumeration, and, more recently, Ligand Designer, play an essential role in the early stages of drug discovery by enabling the prediction of ligand–target interactions at the atomic level.

We also propose that compounds should be tested for their synergistic effects, as some compounds may offer dual inhibition against different targets. This can be achieved by testing the compounds against an in vitro assay for both viral replication targets and viral entry targets. For example, RI173 is a triple inhibitor for Cathepsin L, Mpro, and PLpro, with IC_50_ values of 1.7 µM, 0.6 µM, and 0.2 µM, respectively, while MPI8 is a dual inhibitor for Cathepsin L and Mpro, with IC_50_ values of 1.2 nM and 105 nM, respectively (Table S3). Despite the accuracy and advancement offered by Docking, WaterMap, MM-GB/SA, FEP+, and AI/ML in predicting binding affinities, these approaches have been underreported in SARS-CoV-2 drug design. We suggest that these complementary computer-based techniques should be considered, as they play a huge role in complementing the former approaches. While still acknowledging the advancement of CADD, which has made life much easier for our society, leading to faster, cheaper, and more effective drug development. It is also crucial to balance technological innovation with regulatory and ethical frameworks to ensure that these advancements benefit society at large while also ensuring the synthesizability of newly discovered active compounds by considering retrosynthesis as a guide for synthetic routes. Synthesizability of the enumerated scaffolds is very important, and the enumeration of functional groups with known chemistries is also important. SARS-CoV-2 drug discovery is moving toward fully AI-integrated, mutation-aware, and dynamic computational pipelines that integrate these tools into a single intelligent system to improve and accelerate the reliability of antiviral agent development targeting emerging SARS-CoV-2 variants, long-COVID, and future pandemics. Future progress in SARS-CoV-2 computational drug discovery will depend on integrating complementary methodologies to overcome the limitations of individual approaches. While virtual high-throughput screening (vHTS) and docking remain efficient, their simplified scoring functions limit accuracy, particularly in capturing protein flexibility and solvent effects. Incorporating more rigorous methods, such as absolute binding free energy (ABFE) and MD simulations, is expected to improve thermodynamic accuracy, especially for challenging targets [137,229]. In parallel, hybrid frameworks combining physics-based simulations with machine learning are emerging, aiming to balance the high accuracy of methods like FEP with the scalability of machine learning (ML), although challenges in generalization, data quality, and interpretability remain significant [138,140,231]. Although machine learning scoring functions demonstrate improved accuracy over traditional methods with high-quality crystal structures, their performance on docked poses remains inconsistent and insufficiently validated [139]. Advances in artificial intelligence (AI), including multimodal and physics-informed models, are expected to enhance prediction accuracy and enable efficient exploration of chemical space, particularly through ML-guided docking and active learning strategies [140,244]. Future approaches must also incorporate interaction-aware evaluation metrics (e.g., PLIFs) to ensure biological relevance and improve performance on novel targets [233]. Importantly, next-generation workflows will need to balance molecular novelty with target specificity and integrate computational predictions with automated experimental platforms, ultimately enabling more efficient, accurate, and scalable drug discovery pipelines [244,245,246].

## Figures and Tables

**Table 4:** Ligand interaction diagram (LID) showing the amino acids involved in hydrogen bonding, π-π, and hydrophobic interactions between PLpro inhibitors with the PLpro receptor.

Ligand	Protein-Ligand Interaction	PDB ID	Bond Radii	Reference
GRL-0617	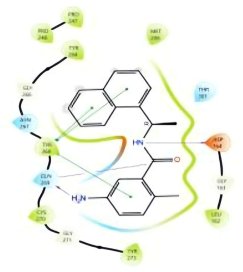	7CMD	2.59 Å	[210]
PF-07957472	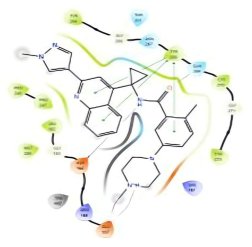	9CSY	2.595 Å	[214]
XR8-24	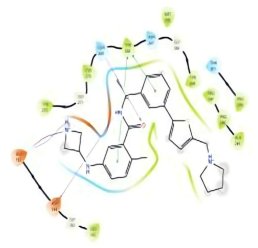	7LBS	2.80 Å	[235]
Jun12682	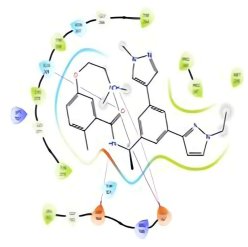	8UOB	2.52 Å	[217]
GZNL-P36	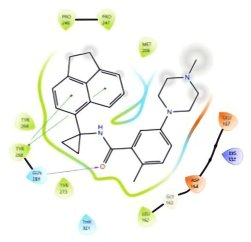	8YX2	2.31 Å	[33]
Jun13296	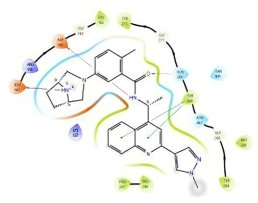	9DNU	2.3 Å	[218]
Jun9-72-2	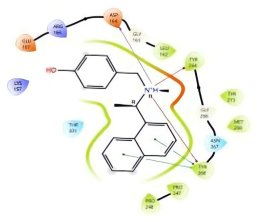	7SDR	2.72 Å	[208]
compound **2**	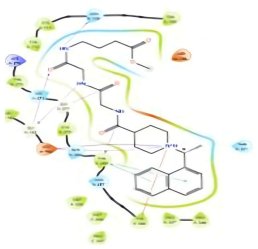	8IHO	2.5 Å	[219]
compound **19**	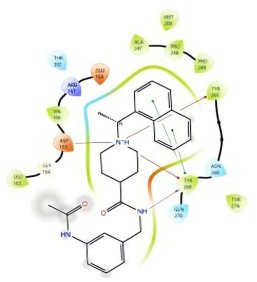	7E35	2.40 Å	[216]
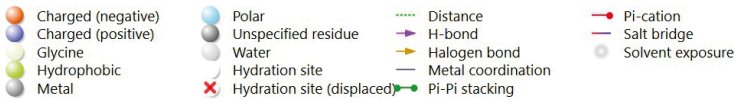				

## Data Availability

No new data were created or analyzed in this study. Data sharing is not applicable to this article.

## Supplementary Materials

The following supporting information can be downloaded at: https://www.mdpi.com/article/10.3390/molecules31162877/s1, Figure S1: Comparative analysis of the activity, metabolic stability and solubulity parameters of all reported compounds targeting Mpro; Figure S2. Comparative analysis of activity of all reported compounds targeting PLpro; Table S1: Pharmacological and physicochemical profiles for SARS-CoV-2 Spike ACE2 inhibitors; Table S2: Pharmacological and physicochemical profiles of inhibitors targeting SARS-CoV-2 TMPRSS2; Table S3: Pharmacological and physicochemical profiles of inhibitors targeting SARS-CoV-2 Cathepsin L; Table S4: Pharmacological and physicochemical profiles of inhibitors targeting SARS-CoV-2 Mpro; Table S5: Pharmacological and physicochemical profiles of inhibitors targeting SARS-CoV-2 PLpro.

## Abbreviations

The following abbreviations are used in this manuscript:

SARS-CoV-2	Severe Acute Respiratory Syndrome Coronavirus-2
SARS-CoV-1	Severe Acute Respiratory Syndrome Coronavirus-1
SARS-CoV	Severe Acute Respiratory Syndrome Coronavirus
FEP+	Free-Energy Perturbation Plus
QSAR	Quantitative Structure–Activity Relationship
ACE2	Angiotensin-Converting Enzyme 2
TMPRSS2	Transmembrane Protease Serine 2
PHEIC	Public Health Emergency of International Concern
EUA	Emergency Use Authorization
Mpro	Main Protease
PLpro	Papain-Like Protease
RdRp	RNA-Dependent RNA Polymerase
SAR	Structure–Activity Relationship
VOC	Variant of Concern
VOI	Variant of Interest
VBM	Variant Being Monitored
VOH	Variant of High Consequence
RBD	Receptor-Binding Domain
TR-PCR	Transcription Reverse–Polymerase Chain Reaction
RNA	Ribonucleic Acid
FDA	Food and Drug Administration
3CLpro	3-Chymotrypsin-Like Protease
US	United States
FRET	Förster Resonance Energy Transfer
CQ	Chloroquine
HCQ	Hydroxychloroquine
CNS	Central Nervous System
BARDA	Biomedical Advanced Research and Development Authority
MHRA	Medicines and Healthcare products Regulatory Agency
UK	United Kingdom
IC_50_	Half-Maximal Inhibitory Concentration
CC_50_	Half-Maximal Cytotoxic Concentration
EC_50_	Half-Maximal Effective Concentration
Ki	Inhibition Constant
ID	Identification
CLint	Intrinsic Clearance
ND*	Not Determined
PMDA	Pharmaceuticals and Medical Devices Agency
NMPA	National Medical Products Administration
CTSL	Cathepsin L
NTD	N-Terminal Domain
hACE2	Human Angiotensin-Converting Enzyme 2
HR	Heptad Repeat
TM	Transmembrane Domain
FP	Fusion Peptide
SP	Serine Protease
ACS	American Chemical Society
SBDD	Structure-Based Drug Design
S	Spike
M	Membrane
E	Envelope
N	Nucleocapsid
ER	Endoplasmic Reticulum
ERGIC	Endoplasmic Reticulum–Golgi Intermediate Compartment
CTD	C-Terminal Domain
LKR	Central Linking Region
NSP5	Non-Structural Protein 5
P	Pocket
S	Site
MD	Molecular Dynamics
MM-PBSA	Molecular Mechanics Poisson–Boltzmann Surface Area
CADD	Computer-Aided Drug Design
MM-GBSA	Molecular Mechanics Generalized Born Surface Area
ADMET	Absorption, Distribution, Metabolism, Excretion, and Toxicity
DNA	Deoxyribonucleic Acid
FEP	Free-Energy Perturbation
MERS	Middle East Respiratory Syndrome
HLM-CLint	Human Liver Microsome Intrinsic Clearance
SM	Starting Material
P1/P2	Products
INT	Intermediate
ΔG	Binding energy
HTS	High-Throughput Screening
LID	Ligand Interaction Diagram
HBD	Hydrogen Bond Donor
HBA	Hydrogen Bond Acceptor
NRF	National Research Foundation
CPRR	Competitive Program for Rated Researchers
vHTS	Virtual High Throughput Screening
ML	Machine Learning
AI	Artificial Intelligence
