# Supplementary material for: Recent Advances in the Design of Inhibitors Targeting the Viral Entry and Replication of the SARS-CoV-2 Virus, Driven by In Silico Approaches
Source: Molecules. 2026 Aug 18;31(16):2877. doi: 10.3390/molecules31162877 (PMC13515069; doi:10.3390/molecules31162877)
Supplement: Supplementary file 1 [file molecules-31-02877-s001.zip › molecules-4387489-supplementary.pdf]

Recent advances in the design of inhibitors targeting the viral entry and replication of the SARS-CoV-2 virus, driven by *in silico* approaches

*Phumelele Nodola, Patience S.S. Molefe, Potlaki Foster Tseki, Njabulo Joyfull Gumede**

Department of Chemical and Physical Sciences, Faculty of Natural Sciences, iYunivesithi Walter Sisulu (iWSU), Private Bag X01, Mthatha, 5099, Eastern Cape, South Africa.

Supporting Information

Table S1. Pharmacological and physicochemical profiles for SARS-CoV-2 Spike ACE2 inhibitors

No.	Name[1]	IC_{50}	EC50	CC50	Type of Assay	Metabolic profiling (CL_{int}) (μl/min/mg)	Approval status	Reference
1	ErB	0.43 µM	ND*	ND*	SARS-CoV-2-RBD-hACE2	ND*	Control (+)	[1]
2	SY (FD&C#6)	>500 µM	ND*	ND*	SARS-CoV-2-RBD-hACE2	ND*	Control (-)	[1]
3	NBIBk	>500 µM	ND*	ND*	SARS-CoV-2-RBD-hACE2	ND*	Hit	[1]
4	CgRd	0.99 µM	ND*	ND*	SARS-CoV-2-RBD-hACE2	ND*	Hit	[1]
5	DV1	1.47 µM	ND*	ND*	SARS-CoV-2-RBD-hACE2	ND*	Hit	[1]
6	EvBI	2.25 µM	ND*	ND*	SARS-CoV-2-RBD-hACE2	ND*	Hit	[1]
7	ChBk	2.57 µM	ND*	ND*	SARS-CoV-2-RBD-hACE2	ND*	Hit	[1]
8	CSc3B	4.25 µM	ND*	ND*	SARS-CoV-2-RBD-hACE2	ND*	Hit	[1]
9	MeBlu	3.26 µM	ND*	ND*	SARS-CoV-2-RBD-hACE2	ND*	Hit	[1]
10	Suramin	>500 µM	ND*	ND*	SARS-CoV-2-RBD-hACE2	ND*	Hit	[1]
11	Chloroquine	>500 µM	ND*	ND*	SARS-CoV-2-RBD-hACE2	ND*	Hit	[1]
12	DRI-C21041	1.81 µM	ND*	ND*	SARS-CoV-2-RBD-hACE2	ND*	Hit	[1]
13	DRI-C23041	0.52 µM	ND*	ND*	SARS-CoV-2-RBD-hACE2	ND*	Experimental	[1]
14	DRI-C24041	3.35 µM	ND*	ND*	SARS-CoV-2-RBD-hACE2	ND*	Hit	[1]
15	DRI-C61041	7.67 µM	ND*	ND*	SARS-CoV-2-RBD-hACE2	ND*	Hit	[1]
16	DRI-C71041	9.95 µM	ND*	ND*	SARS-CoV-2-RBD-hACE2	ND*	Hit	[1]
17	DRI-C2204745	4.04 µM	ND*	ND*	SARS-CoV-2-RBD-hACE2	ND*	Hit	[1]
18	DRI-C91005	0.16 µM	ND*	ND*	SARS-CoV-2-RBD-hACE2	ND*	Hit	[1]
19	DRI-C2105041	>500 µM	ND*	ND*	SARS-CoV-2-RBD-hACE2	ND*	Hit	[1]

No.	Name[1]	IC_{50}	EC50	CC50	Type of Assay	Metabolic profiling (CL_{int}) (μl/min/mg)	Approval status	Reference
20	DRI-C2105045	120.80 µM	ND*	ND*	SARS-CoV-2-RBD-hACE2	ND*	Hit	[1]
21	CgRd	3.92 µM	ND*	ND*	SARS-CoV-2-S1S2-hACE2	ND*	Hit	[1]
22	DV1	2.63 µM	ND*	ND*	SARS-CoV-2-S1S2-hACE2	ND*	Hit	[1]
23	EvBI	1.27 µM	ND*	ND*	SARS-CoV-2-S1S2-hACE2	ND*	Hit	[1]
24	CSc3B	9.94 µM	ND*	ND*	SARS-CoV-2-S1S2-hACE2	ND*	Hit	[1]
25	DRI-C23041	3.38 µM	ND*	ND*	SARS-CoV-2-S1S2-hACE2	ND*	Experimental	[1]
26	DRI-C61041	6.81 µM	ND*	ND*	SARS-CoV-2-S1S2-hACE2	ND*	Hit	[1]
27	DRI-C71041	8.60 µM	ND*	ND*	SARS-CoV-2-S1S2-hACE2	ND*	Hit	[1]
28	DRI-C2204745	4.42 µM	ND*	ND*	SARS-CoV-2-S1S2-hACE2	ND*	Hit	[1]
29	DRI-C91005	0.24 µM	ND*	ND*	SARS-CoV-2-S1S2-hACE2	ND*	Hit	[1]
30	Chloroqunine	>200 µM	ND*	ND*	SARS-CoV-2-S1 – hACE2	ND*	Hit	[1]
31	CgRd	0.31 µM	ND*	ND*	SARS-CoV-2-S1 – hACE2	ND*	Hit	[1]
32	DVI	0.31 µM	ND*	ND*	SARS-CoV-2-S1 – hACE2	ND*	Hit	[1]
33	EvBI	0.19 µM	ND*	ND*	SARS-CoV-2-S1 – hACE2	ND*	Hit	[1]
34	CSC3B	2.88 µM	ND*	ND*	SARS-CoV-2-S1 – hACE2	ND*	Hit	[1]
35	MeBlu	2.65 µM	ND*	ND*	SARS-CoV-2-S1 – hACE2	ND*	Hit	[1]
36	DRI-C23041	1.88 µM	ND*	ND*	SARS-CoV-2-S1 – hACE2	ND*	Experimental	[1]
37	DRI-C24041	3.16 µM	ND*	ND*	SARS-CoV-2-S1 – hACE2	ND*	Hit	[1]
38	DRI-C91005	0.18 µM	ND*	ND*	SARS-CoV-2-S1 – hACE2	ND*	Hit	[1]
39	Ligand_003	2.371 µM	ND*	ND*	Theoretical obtained, experimental (Spike-ACE2)	ND*	Experimental	[2]
40	Ligand_013	0.586 µM, 0.586 µM	ND*	ND*	Theoretical obtained, experimental (Spike-ACE2)	ND*	Hit	[2]
41	Ligand_033	0.663 µM	ND*	ND*	Theoretical obtained (Spike-ACE2)	ND*	Hit	[2]
42	Ligand_044	1.047 µM	ND*	ND*	Theoretical obtained (Spike-ACE2)	ND*	Hit	[2]
43	Ligand_080	0.459 µM	ND*	ND*	Theoretical obtained (Spike-ACE2)	ND*	Hit	[2]
44	Hydroxychloroquine	7.268 µM	ND*	ND*	Theoretical obtained (Spike-ACE2)	ND*	Hit	[2]

No.	Name[1]	IC_{50}	EC50	CC50	Type of Assay	Metabolic profiling (CL_{int}) (μl/min/mg)	Approval status	Reference
45	Heparin sodium	5.5 µg/mL	ND*	ND*	Plaque reduction assay (RBD-ACE2)	ND*	Hit	[3]
46	Aurintricarboxylic acid (ATA)	0.6 µg/mL	ND*	ND*	Plaque reduction assay (RBD-ACE2)	ND*	Experimental	[3]
47	Ellagic acid	2.5 µg/mL	ND*	ND*	Plaque reduction assay (RBD-ACE2)	ND*	Experimental	[3]
48	Compound 1	0.193 ± 0.01* µM	ND*	ND*	Cell-based assay (Vero E6) RBD	ND*	Hit	[4]
39	Compound 2	0.32 ± 0.08* µM	ND*	ND*	Cell-based assay (Vero E6) RBD	ND*	Hit	[4]
50	Compound 3	0.45 ± 0.005* µM	ND*	ND*	Cell-based assay (Vero E6) RBD	ND*	Hit	[4]
51	Compound 4	0.77 ± 0.01* µM	ND*	ND*	Cell-based assay (Vero E6) RBD	ND*	Hit	[4]
52	Compound 5	0.91 ± 0.27* µM	ND*	ND*	Cell-based assay (Vero E6) RBD	ND*	Hit	[4]
53	Compound 6	1.75 ± 0.39* µM	ND*	ND*	Cell-based assay (Vero E6) RBD	ND*	Hit	[4]
54	Compound 7	1.81 ± 0.23* µM	ND*	ND*	Cell-based assay (Vero E6) RBD	ND*	Hit	[4]
55	Compound 8	1.86 ± 0.008* µM	ND*	ND*	Cell-based assay (Vero E6) RBD	ND*	Hit	[4]
56	Compound 9	1.91 ± 0.04* µM	ND*	99.7 ± 1.5 µM	Cell-based assay (Vero E6) RBD	ND*	Experimental	[4]
57	Compound 10	2.35 ± 0.02* µM	ND*	ND*	Cell-based assay (Vero E6) RBD	ND*	Hit	[4]
58	Compound 11	2.82 ± 0.08* µM	ND*	ND*	Cell-based assay (Vero E6) RBD	ND*	Hit	[4]
59	Compound 12	3.3 ± 1.09* µM	ND*	ND*	Cell-based assay (Vero E6) RBD	ND*	Hit	[4]
60	Compound 13	3.31 ± 0.14* µM	ND*	ND*	Cell-based assay (Vero E6) RBD	ND*	Hit	[4]
61	Compound 14	4.17 ± 0.01* µM	ND*	96.8 ± 1.5 µM	Cell-based assay (Vero E6) RBD	ND*	Experimental	[4]
62	Compound 15	4.33 ± 0.80* µM	ND*	≥ 100 µM	Cell-based assay (Vero E6) RBD	ND*	Experimental	[4]
63	Compound 16	4.9 ± 0.24* µM	ND*	ND*	Cell-based assay (Vero E6) RBD	ND*	Hit	[4]
64	Compound 17	5.42 ± 1.53* µM	ND*	ND*	Cell-based assay (Vero E6) RBD	ND*	Hit	[4]
65	Compound 18	4.96 ± 1.45* µM	ND*	ND*	Cell-based assay (Vero E6) RBD	ND*	Hit	[4]
66	Etravirine (positive control)	4.15 ± 0.16* µM	ND*	ND*	Cell-based assay (Vero E6) RBD	ND*	Control	[4]

No.	Name[1]	IC_{50}	EC50	CC50	Type of Assay	Metabolic profiling (CL_{int}) (μl/min/mg)	Approval status	Reference
67	SB27012	3.7 69 µM	ND*	3.13 µM	ELISA-based RBD-ACE2 binding assay, enzymatic assay	ND*	Hit	[5]
68	SB27041	2.2 72 µM	ND*	ND*	ELISA-based RBD-ACE2 binding assay	ND*	Experimental	[5]
69	SB27047	2.6 69 µM	ND*	ND*	ELISA-based RBD-ACE2 binding assay	ND*	Experimental	[5]
		2.6 µM	ND*	ND*	Vero monkey kidney epithelial cells (ACE2)	ND*		
70	SB27051	8.1 53 µM	ND*	ND*	ELISA-based RBD-ACE2 binding assay	ND*	Hit	[5]
71	SB27052	7.0 49 µM	ND*	ND*	ELISA-based RBD-ACE2 binding assay	ND*	Hit	[5]
72	SB27053	7.8 48 µM	ND*	ND*	ELISA-based RBD-ACE2 binding assay	ND*	Hit	[5]
73	Analogue - I (11) Ac-PPSCN-NH2	ND*	ND*	ND*	Cell-based assay (Vero E6 cells, S-RBD/ACE2)	ND*	Hit	[6]
74	Analogue - II (12) Ac-PHPCN-NH2	ND*	ND*	ND*	Cell-based assay (Vero E6, Spike RBD/ACE2)	ND*	Hit	[6]
75	Analogue - III (13) Ac-PHSPN-NH2	0.6 µM	ND*	ND*	Cell-based assay (Vero E6, Spike RBD/ACE2)	ND*	Experimental	[6]
76	ATN-161 Ac-PHSCN-NH2	3.16 µM	ND*	ND*	Cell-based assay (Vero E6, Spike RBD/ACE2)	ND*	Hit	[6]
77	AB-00011778	0.25 µM	ND*	>30 µM	A549-ACE2	ND*	Experimental	[6]
		0.25 µM	ND*	>30 µM	A549-ACE2			185
78	AB-00047476	2.5 µM	ND*	>30 µM	A549-ACE2	ND*	Experimental	[6]
79	H69C2	85.75 µM	ND*	>250	Vero E6-spike	ND*	Experimental	[7]
80	Clofazimine	>100 µM	0.31 µM	ND*	Vero E6-spike RBD		Phase 2 (NCT044695)	[8]
81	ALD-R491	13.5 nM	0.036 µM	>10 µM	HEK-GFP-spike	ND*	Experimental	[9]
82	Sertraline	1.638 ± 0.622 µM	ND*	ND*	Cell-based assay (Vero E6-spike)	ND*	Experimental	[10]
83	Aminobenztropine	0.21 µM	ND*	>200 µM	293T-spike	ND*	Experimental	[11]
84	15f	ND*	1.45 µM	323 µM	Huh-7	ND*	Experimental	[12]
85	MU-UNMC-1	<0.21 µM	ND*	>100 µM	INCN1T (S-RBD)	ND*	Experimental	[13]
86	MU-UNMC-2	0.45 µM	ND*	>100 µM	INCN1T (S-RBD)	ND*	Experimental	[13]
87	MU-UNMC-7	>5 .0 µM	ND*	ND*	INCN1T (S-RBD)	ND*	Hit	[13]
88	MU-UNMC-6	1.91 µM	ND*	ND*	INCN1T (S-RBD)	ND*	Hit	[13]

No.	Name[1]	IC_{50}	EC50	CC50	Type of Assay	Metabolic profiling (CL_{int}) (μl/min/mg)	Approval status	Reference
89	MU-UNMC-8	>5.0 µM	ND*	ND*	INCN1T (S-RBD)	ND*	Hit	[13]
90	P2119	ND*	22 ± 5.6 ng/mL	ND*	spike	ND*	Experimental	[14]
91	P2165	ND*	37 ± 4.7 ng/mL	ND*	spike	ND*	Experimental	[14]
92	6-Thioguanine	ND*	0.55 µM	>40 µM	Calu-3 spike	ND*	Experimental	[15]
93	6-Thioguanosine	ND*	0.49 µM	>40 µM	Calu-3 spike	ND*	Hit	[15]
94	6-mercaptopurine	ND*	0.26 µM	>40 µM	Calu-3 spike	ND*	Hit	[15]
95	Glycyrrhetinic acid	ND*	3.17 µM	>100 µM	Vero-E6 spike-RBD	ND*	Experimental	[15]
96	HCC1	ND*	125.6 µM	391.5 µM	293T-ACE2 spike	ND*	Hit	[16]
97	HCC4	ND*	128.2 µM	515.7 µM	293T-ACE2 spike	ND*	Hit	[16]
98	HCC11	ND*	26.7 µM	>74.1 µM	293T-ACE2 spike	ND*	Hit	[16]
99	HCC1+4+11	ND*	13.3 µM	121.5 µM	293T-ACE2 spike	ND*	experimental	[16]
100	Dichlorcyclizine	ND*	2.9 ± 1.6 µM	>100 µM	Vero-E6-ACE2 spike	ND*	Experimental	[17]
101	Fluoxazolevir	ND*	3.9 ± 1.0 µM	>100 µM	Vero-E6-ACE2 spike	ND*	Experimental	[17]
102	(-)-hopeaphenol	0.11 µM	13.5 µM	>100 µM	Vero-E6-ACE2 spike	ND*	Experimental	[18]
103	Bifonazole	38.84 µM, 40.00 µM	ND*	ND*	VerohACE2-TMPRSS2 cells, Vero E6 ACE2	ND*	Hit	[19]
104	NMT5	ND*	5.28 µM	48.66 µM	HeLa-ACE2	ND*	Experimental	[20]
105	NMT1	ND*	53.88 µM	144.95 µM	HeLa-ACE2	ND*	Hit	[20]
106	NMT3	ND*	87.68 µM	>200 µM	HeLa-ACE2	ND*	Hit	[20]
107	NMT4	ND*	70.02 µM	>200 µM	HeLa-ACE2	ND*	Hit	[20]
108	NMT6	ND*	3.32 µM	17.65 µM	HeLa-ACE2	ND*	Hit	[20]
109	NMT8	ND*	3.75 µM	16.35 µM	HeLa-ACE2	ND*	Hit	[20]
110	Bruceine A	ND*	54 ± 5 nM	>40 µM	Vero E6, ACE2	ND*	Experimental	[21]
111	Gamabufotalin	ND*	2.7 ± 0.2 nM	>40 µM	Vero E6, ACE2	ND*	Experimental	[21]
112	Anisomycin	ND*	0.016 ± 0.001 µM	>40 µM	Vero E6, ACE2	ND*	Experimental	[21]
113	Homoharringtonine	ND*	0.054 ± 0.005 µM	>40 µM	Vero E6, ACE2	ND*	Hit	[21]
114	Bruceine D	ND*	0.22 ± 0.02 µM	>40 µM	Vero E,6 ACE2	ND*	Hit	[21]
115	Remdesivir	ND*	0.43 ± 0.05 µM	>40 µM	Vero E6, ACE2	ND*	Hit	[21]
116	Mitoxantrone	ND*	0.44 ± 0.05 µM	>40 µM	Vero E6, ACE2	ND*	Hit	[21]
117	Digoxin	ND*	0.16 ± 0.02 µM	>40 µM	Vero E6, ACE2	ND*	Hit	[21]
118	Cinobufotalin	ND*	0.14 ± 0.01 µM	>40 µM	Vero E6, ACE2	ND*	Hit	[21]
119	Brexpiprazole	ND*	0.67 ± 0.13 µM	24.5 ± 0.8 µM	Vero E6, ACE2	ND*	Hit	[21]

No.	Name[1]	IC_{50}	EC50	CC50	Type of Assay	Metabolic profiling (CL_{int}) (μl/min/mg)	Approval status	Referenc e
120	6048589 (CKP-25)	3.5 ± 0.5 μM	ND*	ND*	Vero E6, Spike RBD/ACE2	<8.6	Experimental	[21]
121	5979349 (CKP-22)	55 μM	ND*	ND*	Vero E6, Spike RBD/ACE2	ND*	Hit	[22]
122	5465527	9.6 μM	ND*	ND*	Vero E6, Spike RBD/ACE2	ND*	Hit	[22]
123	6052953	11 μM	ND*	ND*	Vero E6, Spike RBD/ACE2	ND*	Hit	[22]
124	6659989	8.4 μM	ND*	ND*	Vero E6, Spike RBD/ACE2	ND*	Hit	[22]
125	5315079	5.5 μM	ND*	ND*	Vero E6, Spike RBD/ACE2	ND*	Hit	[22]
126	5260662	62 μM	ND*	ND*	Vero E6, Spike RBD/ACE2	ND*	Hit	[22]

Table S2. Pharmacological and physicochemical profiles of inhibitors targeting SARS-CoV-2 TMPRSS2.

No.	Name[1]	IC_{50}	EC50	K_i (nM)	Type of TMPRSS Assay	Approval status	Design strategy	Refere nce
1	N-0130	3.1 ± 1.5 nM	30.1 ± 30.1 nM	0.13 ± 0.03	Vero E6	Hit	Retention of peptidomimetic chemotype	[23]
2	N-0438	5.2 ± 5.4 nM	35.7 ± 24.5 nM	1.11 ± 0.09	Vero E6	Hit	Retention of peptidomimetic chemotype	[23]
3	N-0386	3.9 ± 4.4 nM	2.3 ± 1.7 nM	0.51 ± 0.09	Vero E6	Hit	Retention of peptidomimetic chemotype	[23]
4	N-0385	1.9 ±1.4 nM	2.8 ± 1.4 nM	2.6 ± 0.4	Vero E6	Experimental	Retention of peptidomimetic chemotype	[23]
5	N-0385(OH)	>10000 nM	>1000 nM	ND*	Vero E6	Hit	Retention of peptidomimetic chemotype	[23]
6	Camostat mesylate	17.5 ± 18.8 nM	10.6 ± 8.4 nM	5.6 ± 1.0	Vero E6)	Hit	Retention of peptidomimetic chemotype	[23]
7	Camostat	1.5 nM	83 Nm	ND*	Calu-3 Cells	Hit	Repurposed	[24]
8	Nafamostat	0.14 nM	ND*	ND*	Enzymatic assay	Hit	Repurposed	[24]
9	Ac-SKLR-kbt-V (1)	74 nM	307 nM	ND*	Calu-3 Cells	Hit	Retention of peptidomimetic chemotype	[24]
10	Cyclo(DLK)R-kbt (2)	2.6 nM	104 nM	ND*	Calu-3 Cells	Hit	Retention of peptidomimetic chemotype	[24]

11	Cyclo(DMK)R-kbt (19)	19 nM	119 nM	ND*	Calu-3 Cells	Hit	Retention of peptidomimetic chemotype	[24]
12	Cyclo(aGLY)R-kbt (21)	197 nM	565 nM	ND*	Calu-3 Cells	Hit	Retention of peptidomimetic chemotype	[24]
13	Cyclo(DQK)R-kbt (20)	ND*	138 nM	ND*	Calu-3 Cells	Hit	Retention of peptidomimetic chemotype	[24]
14	Ac-WFR-kbt (8)	9.4 nM	ND*	ND*	Enzymatic assay	Hit	Retention of peptidomimetic chemotype	[24]
15	Ac-SKFR-kt (9)	7.9 nM	ND*	ND*	Enzymatic assay	Hit	Retention of peptidomimetic chemotype	[24]
16	Ac-KQFR-kt (10)	29 nM	ND*	ND*	Enzymatic assay	Hit	Retention of peptidomimetic chemotype	[24]
17	Ac-SQLR-kt (11)	16 nM	ND*	ND*	Enzymatic assay	Hit	Retention of peptidomimetic chemotype	[24]
18	Ac-LLR-kt (18)	54 nM	349 nM	ND*	Calu-3 Cells	Hit	Retention of peptidomimetic chemotype	[24]
19	Ac-SKLR-kbt (3)	39 nM	ND*	ND*	Enzymatic assay	Hit	Retention of peptidomimetic chemotype	[24]
20	Ac-FLFR-kbt (12)	3.0 nM	ND*	ND*	Enzymatic assay	Hit	Retention of peptidomimetic chemotype	[24]
21	Ac-dWFR-kbt (13)	1.1 nM	32 nM	ND*	Calu-3 Cells	Hit	Retention of peptidomimetic chemotype	[24]
22	dWFR-kbt (14)	39 nM	ND*	ND*	Enzymatic assay	Hit	Retention of peptidomimetic chemotype	[24]
23	dWFR-kbt-CO2H (15)	42 nM	ND*	ND*	Enzymatic assay	Hit	Retention of peptidomimetic chemotype	[24]
24	Ac-WLFR-kbt (16)	6.3 nM	150 nM	ND*	Calu-3 Cells	Hit	Retention of peptidomimetic chemotype	[24]
25	Ac-KQLR-kbt (17)	ND	78 nM	ND*	Calu-3 Cells	Hit	Retention of peptidomimetic chemotype	[24]
26	MM3122 (4)	340 pM	430 pM	ND*	Calu-3 Cells	Lead	Retention of peptidomimetic chemotype	[24]
27	MM3144 (6)	310 pM	530 pM	ND*	Calu-3 Cells	Experimental	Retention of peptidomimetic chemotype	[24]
28	MM3116 (7)	250 pM	3.6 nM	ND*	Calu-3 Cells	Experimental	Retention of peptidomimetic chemotype	[24]
29	MM3123 (5)	280 pM	860 pM	ND*	Calu-3 Cells	Experimental	Retention of peptidomimetic chemotype	[24]
30	N-0385	ND*	ND*	0.28 ± 0.03	Inhibition assay	Lead	Retention of peptidomimetic chemotype	[25]
31	1	ND*	ND*	0.33 ± 0.09	Inhibition assay	Hit	Retention of peptidomimetic chemotype	[25]
32	2	ND*	ND*	0.38 ± 0.11	Inhibition assay	Hit	Retention of peptidomimetic chemotype	[25]
33	3	ND*	ND*	0.41 ± 0.13	Inhibition assay	Hit	Retention of peptidomimetic chemotype	[25]
34	4	ND*	ND*	0.36 ± 0.10	Inhibition assay	Hit	Retention of peptidomimetic chemotype	[25]

35	5	ND*	ND*	0.60 ± 0.16	Inhibition assay	Hit	Retention of peptidomimetic chemotype	[25]
36	6	ND*	ND*	0.29 ± 0.06	Inhibition assay	Hit	Retention of peptidomimetic chemotype	[25]
37	7	ND*	ND*	0.37 ± 0.04	Inhibition assay	Hit	Retention of peptidomimetic chemotype	[25]
38	8	ND*	ND*	0.39 ± 0.05	Inhibition assay	Hit	Retention of peptidomimetic chemotype	[25]
39	9	ND*	ND*	0.13 ± 0.03	Inhibition assay	Experimental	Retention of peptidomimetic chemotype	[25]
40	10	ND*	ND*	0.14 ± 0.02	Inhibition assay	Hit	Retention of peptidomimetic chemotype	[25]
41	11	ND*	ND*	0.70 ± 0.07	Inhibition assay	Hit	Retention of peptidomimetic chemotype	[25]
42	12	ND*	ND*	0.39 ± 0.05	Inhibition assay	Hit	Retention of peptidomimetic chemotype	[25]
43	13	ND*	ND*	0.15 ± 0.00 03	Inhibition assay	Hit	Retention of peptidomimetic chemotype	[25]
44	14	ND*	ND*	0.16 ± 0.01	Inhibition assay	Hit	Retention of peptidomimetic chemotype	[25]
45	15	ND*	ND*	0.85 ± 0.05	Inhibition assay	Hit	Retention of peptidomimetic chemotype	[25]
46	16	ND*	ND*	0.53 ± 0.03	Inhibition assay	Hit	Retention of peptidomimetic chemotype	[25]
47	17	ND*	ND*	0.44 ± 0.12	Inhibition assay	Hit	Retention of peptidomimetic chemotype	[25]
48	18	ND*	ND*	1.55 ± 0.17	Inhibition assay	Hit	Retention of peptidomimetic chemotype	[25]
49	19	ND*	ND*	0.34 ± 0.07	Inhibition assay	Hit	Retention of peptidomimetic chemotype	[25]
50	Nafamostat mesylate	ND*	5 nM	ND*	Cell-based assay (Calu-3)	Experimental	Non-peptidomimetic	[26]
51		0.8 nM	ND*	ND*				
52	Camostat mesylate	ND*	87 nM	ND*	Cell-based assay (Calu-3)	Experimental	Non-peptidomimetic	[26]
53		6.7 nM	ND*	ND*				[27]
54	Gabeexate mesylate	ND*	1.2 M	ND*	Cell-based assay (Calu-3)	Hit	Non-peptidomimetic	[26]
55	1	ND*	ND*	86.7 nM	Cell-based assay (Caco-2)	Hit	Retention of peptidomimetic chemotype	[28]
56	2	32 nM	ND*	3.8 nM	Cell-based assay (Caco-2)	Experimental	Retention of peptidomimetic chemotype	[28]
57	3	ND*	ND*	9.1 nM	Cell-based assay (Caco-2)	Hit	Retention of peptidomimetic chemotype	[28]
58	4	ND*	ND*	8.5 nM	Cell-based assay (Caco-2)	Hit	Retention of peptidomimetic chemotype	[28]
59	5	12.7 nM	ND*	2.5 nM	Cell-based assay (Caco-2)	Experimental	Retention of peptidomimetic chemotype	[28]

60	6	ND*	ND*	71.5 nM	Cell-based assay (Caco-2)	Hit	Retention of peptidomimetic chemotype	[28]
61	7	ND*	ND*	57.7 nM	Cell-based assay (Caco-2)	Hit	Retention of peptidomimetic chemotype	[28]
62	8	ND*	ND*	215.9 nM	Cell-based assay (Caco-2)	Hit	Retention of peptidomimetic chemotype	[28]
63	CM	ND*	ND*	0.4 nM	Cell-based assay (Caco-2)	Hit	Retention of peptidomimetic chemotype	[28]
64	FOY-251	ND*	ND*	9.7 nM	Cell-based assay (Caco-2)	Hit	Retention of peptidomimetic chemotype	[28]
65	Halofugino ne	30 nM, 0.069 µM	ND*	CC50 ~5.54 µM	Caco-2 cells, TMPRSS2-HiBiT-BEAS-2B cells	Experimental	Repurposed	[29]
66	Homoharri ngtonine	30 nM, 0.061 µM	ND*	CC50>10 µM	Caco-2 cells, TMPRSS2-HiBiT-BEAS-2B cells	Experimental	Repurposed	[29]
67	Avoralstat	2.7 ± 0.19 nM	2.8 ± 0.7 µM	ND*	Calu-3 2B4 Cells	Experimental	Repurposed	**[30]**
68	SBTI	121 ± 4 nM	ND*	ND*	Calu-3 2B4 Cells	Hit	Repurposed	**[30]**
69	Antipain	748 ± 63 nM	ND*	ND*	Calu-3 2B4 Cells	Hit	Repurposed	**[30]**
70	PC1-2748	1.41 ± 0.04 µM	ND*	ND*	Calu-3 2B4 Cells	hit	Repurposed	**[30]**
71	Benzimidin e	120 ± 20 µM	ND*	ND*	Biochemica l assay	Hit	Repurposed	[27]
72	6-amidino-2-napthol	1.6 ± 0.5 µM	ND*	ND*	Biochemica l assay	Hit	Repurposed	[27]
73	Sunflowertr ypsin inhibitor-1	0.4 ± 0.2 µM	ND*	ND*	Biochemica l assay	Hit	cyclic peptide	[27]
74	Otamixaba n	18.7 µM	ND*	ND*	Calu-3	Hit	Repurposed	[31]
75	Compound 7	14.33 ± 0.23 µM	11.77 ± 0.04 µM	110.90 µM	Vero E6	Experimental	Repurposed	[32]
76	Tafenoquin e	31.8 µM	9.48 ± 0.48 µM	9.74 ± 1.31 µM	Vero E6	Hit	Repurposed	[32]
77	Compound 3	ND*	ND*	15.74 ± 3.84 µM	Vero E6	Hit	TFQ derivative	[32]
78	Compound 8	ND*	ND*	5.36 ± 0.61 µM	Vero E6	Hit	TFQ derivative	[32]
79	Compound 13	ND*	ND*	4.70 ± 0.65 µM	Vero E6	Hit	TFQ derivative	[32]
80	Compound 14	ND*	ND*	13.37 ± 1.70 µM	Vero E6	Hit	TFQ derivative	[32]

Table S3. Pharmacological and physicochemical profiles of inhibitors targeting SARS-CoV-2 Cathepsin L.

No.	Name[1]	IC_{50} (catsL)	IC_{50} (Mpro)	IC_{50} (PLpro)	EC_{50}	CC_{50}	Type of Assay	Approval status	Design strategy	Refere nce
1	MPI1	0.079 ± 0.029 nM	100 ± 23 nM	ND*	ND*	ND*	Inhibition assay	Hit	Peptidomim etic	[33]
2	MPI2	0.19 ± 0.03 nM	103 ± 14 nM	ND*	ND*	ND*	Inhibition assay	Hit	Peptidomim etic	[33]
3	MPI3	0.30 ± 0.07 nM	8.5 ± 1.5 nM	ND*	ND*	ND*	Inhibition assay	Hit	Peptidomim etic	[33]

4	MPI4	0.33 ± 0.04 nM	15 ± 5 nM	ND*	ND*	ND*	Inhibition assay	Hit	Peptidomimetic	[33]
5	MPI5	2.3 ± 1.9 nM	33 ± 2 nM	ND*	ND*	ND*	Inhibition assay	Hit	Peptidomimetic	[33]
6	MPI6	0.45 ± 0.08 nM	60 ± 4 nM	ND*	ND*	ND*	Inhibition assay	Hit	Peptidomimetic	[33]
7	MPI7	0.61 ± 0.15 nM	47 ± 3 nM	ND*	ND*	ND*	Inhibition assay	Hit	Peptidomimetic	[33]
8	MPI8	1.2 ± 1.0 nM	105 ± 22 nM	ND*	ND*	ND*	Inhibition assay	Experimental	Peptidomimetic	[33]
9	MPI9	0.56 ± 0.06 nM	56 ± 14 nM	ND*	ND*	ND*	Inhibition assay	Hit	Peptidomimetic	[33]
10	11a	0.14 ± 0.04 nM	31 ± 3 nM	ND*	ND*	ND*	Inhibition assay	Hit	Peptidomimetic	[33]
11	GC376	0.23 ± 0.05 nM	31 ± 4 nM	ND*	ND*	ND*	Inhibition assay	Hit	Peptidomimetic	[33]
12	Disulfiram	2.2 ± 1.4	1.5 ± 0.1 µM	3.8 ± 0.1 µM	0.5 ± 0.2 µM	0.9 ± 0.3 µM	Cell-based assay and enzymatic assay	Hit	Repurposed	[34]
13	RI173	1.7 ± 0.6	0.6 ± 0.1 µM	0.2 ± 0.1 µM	0.1 ± 0.00 µM	0.3 ± 0.1 µM	Cell-based assay and enzymatic assay	Experimental	Thiuram disulfide derivatives	[34]
14	RI175	1.9 ± 0.6	0.3 ± 0.1 µM	0.6 ± 0.1 µM	>60 µM	>60 µM	Cell-based assay and enzymatic assay	Hit	Thiuram disulfide derivatives	[34]
15	RI172	0.2 ± 0.0	0.6 ± 0.1 µM	0.7 ± 0.0 µM	<0.08 µM	<0.08 µM	Cell-based assay and enzymatic assay	Hit	Thiuram disulfide derivatives	[34]
16	RI174	16.9 ± 6.9	2.0 ± 0.3 µM	0.6 ± 0.0 µM	<0.08 µM	<0.08 µM	Cell-based assay and enzymatic assay	Hit	Thiuram disulfide derivatives	[34]
17	RI171	4.5 ± 0.7	3.2 ± 0.1 µM	1.9 ± 0.1 µM	1.9 ± 0.2 µM	1.7 ± 0.4 µM	Cell-based assay and enzymatic assay	Hit	Thiuram disulfide derivatives	[34]
18	RI177	>50	3.8 ± 0.6 µM	>50 µM	>60 µM	>60 µM	Cell-based assay and enzymatic assay	Hit	Thiuram disulfide derivatives	[34]
19	RI176	>50	>50 µM	>50 µM	ND*	ND*	Cell-based assay and enzymatic assay	Hit	Thiuram disulfide derivatives	[34]
20	Calpain inhibitor II	0.41 ± 0.16 nM	970 ± 268 nM	ND*	ND*	ND*	FRET-Based enzymatic assay	Experimental	analogs of GC-376	[35]
21	Calpain inhibitor I	ND*	8.60 ± 1.46 µM	ND*	ND*	ND*	FRET-Based enzymatic assay	Hit	analogs of GC-377	[35]
22	Calpain inhibitor XII	1.62 ± 0.33 nM	453 ± 71 nM	ND*	ND*	ND*	FRET-Based enzymatic assay	Experimental	analogs of GC-378	[35]
23	UAWJ257	ND*	64.5 ± 15.4 µM	ND*	ND*	ND*	FRET-Based enzymatic assay	Hit	analogs of GC-379	[35]
24	E-64d	ND*	ND*	ND*	0.27 µM	ND*	Vero E6	Experimental	ND*	[36]
25	Z-FY-CHO	5 µM	ND*	ND*	0.62 µM	ND*	Vero E6	Experimental	ND*	[36]
26	MOL-28170	ND*	ND*	ND*	1.56 µM	ND*	Vero E6	Experimental	ND*	[36]
27	Cathepsin inhibitor 1	ND*	ND*	ND*	0.14 µM	ND*	Vero E6	Experimental	ND*	[36]
28	Oxocarbate	ND*	ND*	ND*	10.89 µM	ND*	Vero E6	hit	ND*	[36]
29	SM141	60 ± 1 nM	900 ± 100 nM	ND*	8.2 ± 0.9 nM	ND*	A549-hACE2	Experimental	Peptidomimetic	[37]

30	SM142	145 ± 14 nM	1800 ± 100 nM	ND*	14.7 ± 2.2 nM	ND*	A549-hACE2	Experimental	Peptidomimetic	[37]
31	Obatoclax	<1 µM	ND*	ND*	ND*	>100 µM	Cau-3, Caco-2 cells	Experimental	Repurposed	[38]
32	K777	ND*	ND*	ND*	74 nM	ND*	Vero E6	Experimental	Irreversible covalent inhibitor	[39]
		ND*	ND*	ND*	0.035 µM	ND*	Vero E6	Experimental	Irreversible covalent inhibitor	[36]

Table S4. Pharmacological and physicochemical profiles of inhibitors targeting SARS-CoV-2 Mpro.

Name	IC_{50} (nM)	K_i (nM)	Type of Mpro Assay	EC_{50} µM	Type of Viral assay	CC_{50}	Metabolic profiling (CL_{int}) (µl/min/mg)	Metabolite ID	Log D	Approval status	Design strategy	References
Ebselen	9.35 ± 0.18 µM	ND*	Enzymatic Assay	4.67 µM	Vero E6	ND*	ND*	ND*	ND*	Experimental	Repurposed	[40]
Ebselen	0.67 ±0.09 µM	ND*	Enzymatic Assay	4.67 µM	ND*	ND*	ND*	ND*	ND*	Experimental	Repurposed	[40]
Disulfiram	1.55 ± 0.30 µM	ND*	Enzymatic Assay	ND*	ND*	ND*	ND*	ND*	ND*	Hit	Repurposed	[40]
Tideglusib	1.82 ± 0.06 µM	ND*	Enzymatic Assay	ND*	ND*	ND*	ND*	ND*	ND*	Hit	Repurposed	[40]
Carmofur	15.75 ± 8.22 µM	ND*	Enzymatic Assay	ND*	ND*	ND*	ND*	ND*	ND*	Hit	Repurposed	[40]
Shikonin	21.39 ± 7.06 µM	ND*	Enzymatic Assay	ND*	ND*	ND*	ND*	ND*	ND*	Hit	Repurposed	[40]
PX-12	ND*	ND*	Enzymatic Assay	ND*	ND*	ND*	ND*	ND*	ND*	Hit	Irreversible inhibitor	[40]
Atovaquone	ND*	ND*	Cell based and enzymatic assay	1.20 µM	Vero E6	>100	ND*	ND*	ND*	Hit	Repurposed	[41]
Mebendazole	19.94 µM	ND*	Cell based and enzymatic assay	1.30 µM	Vero E6	>100	ND*	ND*	ND*	Experimental	Repurposed	[41]
Dronedarone	18.03 µM	ND*	Cell based and enzymatic assay	1.50 µM	Vero E6	22.70	ND*	ND*	ND*	Experimental	Repurposed	[41]

Ouabain	ND*	ND*	Cell based and enzymatic assay	0.03 µM	Vero E6	>100	ND*	ND*	ND*	Hit	Repurposed	[41]
Digoxin	ND*	ND*	Cell based and enzymatic assay	0.07 µM	Vero E6	>100	ND*	ND*	ND*	Hit	Repurposed	[41]
Entacapone	9.04 µM	ND*	Cell based and enzymatic assay	ND*	Vero E6	>100	ND*	ND*	ND*	Experimental	Repurposed	[41]
Nirmatrelvir, 1	ND*	3.11 nM	FRET assay	81.4 nM	Vero E6	ND*	25	CYP3A4	1.9	FDA approved	Retention of peptidomimetic chemotype	[42]
Nirmatrelvir	28.1 nM	ND*	Enzymatic Fluorescence Assay	148 nM	HeLa-ACE2	>40 μM	32.8	CYP3A4	1.5	FDA approved	Retention of peptidomimetic chemotype	[43]
Nirmatrelvir	2.57 nM	ND*	Biochemical assay	pIC50 = 8.59	CPE – Reduction	pEC50 = 7.00	ND*	CYP3A4	ND*	FDA approved	Retention of peptidomimetic chemotype	[44]
Nirmatrelvir	ND*	0.003 ± 0.0004 µM	Enzymatic assay	<0.01 µM	Huh-7-ACE2	>100 µM	ND*	ND*	ND*	FDA approved	Retention of peptidomimetic chemotype	[45]
2	ND*	5.49 nM	FRET assay	2740 nM	Vero E6	ND*	24.3	ND*	0.8	Hit	Retention of peptidomimetic chemotype	[42]
3	ND*	1.12 nM	FRET assay	118 nM	Vero E6	ND*	8.1	ND*	1.2	Hit	Retention of peptidomimetic chemotype	[42]
4	ND*	0.71 nM	FRET assay	80.9 nM	Vero E6	ND*	9.42	ND*	1.7	Hit	Retention of peptidomimetic chemotype	[42]
5	ND*	0.63 nM	FRET assay	141 nM	Vero E6	ND*	7.12	ND*	1.0	Hit	Retention of peptidomimetic chemotype	[42]

6	ND*	2.83 nM	FRET assay	193 nM	Vero E6	ND*	7.12	ND*	1.2	Hit	Retention of peptidomimetic chemotype	[42]
7	ND*	3.88 nM	FRET assay	450 nM	Vero E6	ND*	7.12	ND*	0.6	Hit	Retention of peptidomimetic chemotype	[42]
8	ND*	4.46 nM	FRET assay	65.5 nM	Vero E6	ND*	13.0	ND*	1.4	Hit	Retention of peptidomimetic chemotype	[42]
PF-07817883, 9	18 nM	2.48 nM	FRET assay	180 nM	Vero E6	ND*	4.7	CYP3A4/5	0.9	Phase II	Retention of peptidomimetic chemotype	[42]
PF-07817883, Ibuzatrelvir	19	5 nM	FRET assay	ND*	ND*	ND*	4.7	ND*	ND*	Phase II	Retention of peptidomimetic chemotype	[46]
10	ND*	0.17 nM	FRET assay	46.2	Vero E6	ND*	9.5	ND*	1.2	Hit	Retention of peptidomimetic chemotype	[42]
11	ND*	13.27 nM	FRET assay	488	Vero E6	ND*	7.12	ND*	0.9	Hit	Retention of peptidomimetic chemotype	[42]
12	ND*	1.13 nM	FRET assay	78.9	Vero E6	ND*	7.12	ND*	0.7	Hit	Retention of peptidomimetic chemotype	[42]
1	0.74 μM	ND*	Enzymatic Fluorescence Assay	ND*	ND*	ND*	ND*	ND*	ND*	Hit	Inhibitor Design Targeting Cys44 in the S2 Pocket	[47]
5	20 μM	ND*	Enzymatic Fluorescence Assay	ND*	ND*	ND*	ND*	ND*	ND*	Hit	Inhibitor Design Targeting Cys44 in the S2 Pocket	[47]

8	20 μM	ND*	Enzymatic Fluorescence Assay	ND*	ND*	ND*	ND*	ND*	ND*	Hit	Inhibitor Design Targeting Cys44 in the S2 Pocket	[47]
13	0.6 μM	ND*	Enzymatic Fluorescence Assay	2.60 μM	Vero	12.1 μM	ND*	ND*	ND*	Hit	Inhibitor Design Targeting Cys44 in the S2 Pocket	[47]
14	0.95 μM	ND*	Enzymatic Fluorescence Assay	ND*	ND*	ND*	ND*	ND*	ND*	Hit	Inhibitor Design Targeting Cys44 in the S2 Pocket	[47]
15	3.2 μM	ND*	Enzymatic Fluorescence Assay	ND*	ND*	ND*	ND*	ND*	ND*	Hit	Inhibitor Design Targeting Cys44 in the S2 Pocket	[47]
16	3.6 μM	ND*	Enzymatic Fluorescence Assay	ND*	ND*	ND*	ND*	ND*	ND*	Hit	Inhibitor Design Targeting Cys44 in the S2 Pocket	[47]
17	3.6 μM	ND*	Enzymatic Fluorescence Assay	ND*	ND*	ND*	ND*	ND*	ND*	Hit	Inhibitor Design Targeting Cys44 in the S2 Pocket	[47]
18	3.6 μM	ND*	Enzymatic Fluorescence Assay	ND*	ND*	ND*	ND*	ND*	ND*	Hit	Inhibitor Design Targeting Cys44 in the S2 Pocket	[47]
19	2.2 μM	ND*	Enzymatic Fluorescence Assay	ND*	ND*	ND*	ND*	ND*	ND*	Hit	Inhibitor Design Targeting Cys44 in the S2 Pocket	[47]
22	1.61 μM	ND*	Enzymatic Fluoresce	ND*	ND*	ND*	ND*	ND*	ND*	Hit	Inhibitor Design	[47]

			nce Assay								Targeting Cys44 in the S2 Pocket	
24	2.4 μM	ND*	Enzymatic Fluorescence Assay	ND*	ND*	ND*	ND*	ND*	ND*	Hit	Inhibitor Design Targeting Cys44 in the S2 Pocket	[47]
25	6.6 μM	ND*	Enzymatic Fluorescence Assay	ND*	ND*	ND*	ND*	ND*	ND*	Hit	Inhibitor Design Targeting Cys44 in the S2 Pocket	[47]
26	0.49 μM	ND*	Enzymatic Fluorescence Assay	ND*	ND*	ND*	ND*	ND*	ND*	Hit	Inhibitor Design Targeting Cys44 in the S2 Pocket	[47]
30	0.76 μM	ND*	Enzymatic Fluorescence Assay	4.88 μM	Vero	> 300 µM	ND*	ND*	ND*	Experimental	Inhibitor Design Targeting Cys44 in the S2 Pocket	[47]
PF-00835231	101 nM	ND*	Enzymatic Florescence Assay	630 nM	HeLa-ACE2	ND*	13.9	ND*	1.2	Lead	hydroxymethyl ketone warhead	[43]
CMX990	23.4 nM	ND*	Enzymatic Florescence Assay	9.6 nM	HeLa-ACE2	>40 µM	7.5	No CYP450	2.3	Phase I	trifluoromethoxymethyl ketone warhead	[43]
1	ND*	ND*	Enzymatic Florescence Assay	72 nM	HeLa-ACE2	ND*	147.7	ND*	4.4	Hit	Interconversion of warheads	[43]
2	14.8 nM	ND*	Enzymatic Florescence Assay	54 nM	HeLa-ACE2	ND*	1109.4	ND*	3.3	Hit	Interconversion of warheads	[43]
3	76.3 nM	ND*	Enzymatic Florescence Assay	17 nM	HeLa-ACE2	ND*	29.6	ND*	2.8	Hit	Interconversion of warheads	[43]
4	16.1 nM	ND*	Enzymatic Florescence Assay	23 nM	HeLa-ACE2	ND*	53.7	ND*	2.4	Hit	Interconversion of warheads	[43]

5	81.1 nM	ND*	Enzymatic Florescence Assay	145 nM	HeLa-ACE2	ND*	45.3	ND*	1.6	Hit	Interconversion of warheads	[43]
6	28.8 nM	ND*	Enzymatic Florescence Assay	99 nM	HeLa-ACE2	ND*	34.2	ND*	2.0	Hit	Interconversion of warheads	[43]
7	24.0 nM	ND*	Enzymatic Florescence Assay	117 nM	HeLa-ACE2	ND*	62.2	ND*	2.6	Hit	Interconversion of warheads	[43]
8	34.7 nM	ND*	Enzymatic Florescence Assay	61 nM	HeLa-ACE2	ND*	26.2	ND*	3.0	Hit	Interconversion of warheads	[43]
9	36.5 nM	ND*	Enzymatic Florescence Assay	108 nM	HeLa-ACE2	ND*	16.3	ND*	2.0	Hit	Interconversion of warheads	[43]
10	35.4 nM	ND*	Enzymatic Florescence Assay	346 nM	HeLa-ACE2	ND*	12.8	ND*	1.3	Hit	Interconversion of warheads	[43]
11	34.5 nM	ND*	Enzymatic Florescence Assay	62 nM	HeLa-ACE2	ND*	8.4	ND*	2.0	Hit	Interconversion of warheads	[43]
12	33.7 nM	ND*	Enzymatic Florescence Assay	24 nM	HeLa-ACE2	ND*	17.4	ND*	1.0	Hit	Interconversion of warheads	[43]
13	34.8 nM	ND*	Enzymatic Florescence Assay	14 nM	HeLa-ACE2	ND*	19.2	ND*	3.6	Hit	Interconversion of warheads	[43]
14	60.1 nM	ND*	Enzymatic Florescence Assay	138 nM	HeLa-ACE2	ND*	12.2	ND*	2.0	Hit	Interconversion of warheads	[43]
15	160 nM	ND*	Enzymatic Florescence Assay	245 nM	HeLa-ACE2	ND*	13.0	ND*	0.3	Hit	Interconversion of warheads	[43]
16	22.0 nM	ND*	Enzymatic Florescence Assay	50 nM	HeLa-ACE2	ND*	14.9	ND*	2.6	Hit	Interconversion of warheads	[43]
1	2,45 E-09	ND*	SARS-CoV-2 Mpro biochemical assay	pEC50 = 6.89	Vero E6	ND*	185.8	ND*	1.65	Lead	Peptidomimetic with tetrafluorophenoxymethyl ketone warhead	[43]
2	4,27 E-06	ND*	SARS-CoV-2 Mpro biochemical assay	pEC50 = 4.66	Vero E6	ND*	ND*	ND*	ND*	Hit	Peptidomimetic with tetrafluorophe	[43]

											noxymethyl ketone warhead	
3	1,15 E-08	ND*	SARS-CoV-2 Mpro biochemical assay	pEC 50 = 7.15	Vero E6	ND*	206.3	ND*	2.23	Hit	Peptidomimetic with tetrafluorophenoxymethyl ketone warhead	[43]
4	3,80 E-06	ND*	SARS-CoV-2 Mpro biochemical assay	pEC 50 = 5.65	Vero E6	ND*	9.31	ND*	>3.5	Hit	Peptidomimetic with tetrafluorophenoxymethyl ketone warhead	[43]
5	4,17 E-08	ND*	SARS-CoV-2 Mpro biochemical assay	pEC 50 = 5.93	Vero E6	ND*	181.7	ND*	1.50	Hit	Peptidomimetic with tetrafluorophenoxymethyl ketone warhead	[43]
6	6,03 E-09	ND*	SARS-CoV-2 Mpro biochemical assay	pEC 50 = 5.72	Vero E6	ND*	155.5	ND*	3.08	Hit	Peptidomimetic with tetrafluorophenoxymethyl ketone warhead	[43]
7	2,75 E-09	ND*	SARS-CoV-2 Mpro biochemical assay	pEC 50 = 7.30	Vero E6	ND*	121.2	ND*	2.82	Hit	Peptidomimetic with tetrafluorophenoxymethyl ketone warhead	[43]
8	5,62 E-09	ND*	SARS-CoV-2 Mpro biochemical assay	pEC 50 = 6.80	Vero E6	ND*	64.5	ND*	3.26	Hit	Peptidomimetic with tetrafluorophenoxymethyl ketone warhead	[43]
9	5,50 E-09	ND*	SARS-CoV-2 Mpro biochemical assay	pEC 50 = 5.92	Vero E6	ND*	133.6	ND*	2.60	Hit	Peptidomimetic with tetrafluorophenoxymethyl ketone warhead	[43]

10	7,08 E-09	ND*	SARS-CoV-2 Mpro biochemical assay	pEC 50 = 6.92	Vero E6	ND*	47.6	ND*	3.15	Hit	Peptidomimetic with tetrafluorophenoxymethyl ketone warhead	[43]
11	6,76 E-09	ND*	SARS-CoV-2 Mpro biochemical assay	pEC 50 = 6.12	Vero E6	ND*	124.3	ND*	2.03	Hit	Peptidomimetic with tetrafluorophenoxymethyl ketone warhead	[43]
12	1,78 E-07	ND*	SARS-CoV-2 Mpro biochemical assay	pEC 50 = 6.19	Vero E6	ND*	76.8	ND*	2.88	Hit	Peptidomimetic with tetrafluorophenoxymethyl ketone warhead	[43]
13	2,95 E-07	ND*	SARS-CoV-2 Mpro biochemical assay	pEC 50 = 5.78	Vero E6	ND*	78.8	ND*	2.70	Hit	Peptidomimetic with tetrafluorophenoxymethyl ketone warhead	[43]
14	3,63 E-09	ND*	SARS-CoV-2 Mpro biochemical assay	pEC 50 = 7.40	Vero E6	ND*	112.1	ND*	2.52	Hit	Peptidomimetic with tetrafluorophenoxymethyl ketone warhead	[43]
15	5,89 E-09	ND*	SARS-CoV-2 Mpro biochemical assay	pEC 50 = 7.40	Vero E6	ND*	240.3	ND*	2.99	Hit	Peptidomimetic with tetrafluorophenoxymethyl ketone warhead	[43]
16	6,76 E-09	ND*	SARS-CoV-2 Mpro biochemical assay	pEC 50 = 7.40	Vero E6	ND*	153.7	ND*	2.95	Hit	Peptidomimetic with tetrafluorophenoxymethyl ketone warhead	[43]
1	4.1 μM	ND*	Mpro Fluorescence Assay	ND*	ND*	ND*	ND*	ND*	ND*	Hit	Algorithmic de novo design	[48]

2	16 μM	ND*	Mpro Fluorescence Assay	ND*	ND*	ND*	ND*	ND*	ND*	Hit	Algorithmic de novo design	[48]
3	58 μM	ND*	Mpro Fluorescence Assay	13 μM	Vero-E6 (Viral replication)	> 100 μM	ND*	ND*	ND*	Hit	Algorithmic de novo design	[48]
JOR-UNI-2fc98d0b-12	3.06 μM	ND*	Mpro Fluorescence Assay	ND*	ND*	ND*	ND*	ND*	ND*	Hit	Algorithmic de novo design	[48]
TRY-UNI-2eddb1ff-7	3.61 μM	ND*	Mpro Fluorescence Assay	ND*	ND*	ND*	ND*	ND*	ND*	Hit	Algorithmic de novo design	[48]
AGN-NEW-891393a6-1	6.68 μM	ND*	Mpro Fluorescence Assay	ND*	ND*	ND*	ND*	ND*	ND*	Hit	Algorithmic de novo design	[48]
TRY-UNI-7149760b-6	24.0 μM	ND*	Mpro Fluorescence Assay	ND*	ND*	ND*	ND*	ND*	ND*	Hit	AI/FEP & Crowd sourcing	[49]
MAT-POS-b3e365b9-1	140 nM	ND*	Mpro Fluorescence Assay	ND*	ND*	ND*	ND*	ND*	ND*	Hit	AI/FEP & Crowd sourcing	[49]
APL-POS-C59291d4-6	3.1 μM	ND*	Mpro Fluorescence Assay	ND*	ND*	ND*	ND*	ND*	ND*	Hit	AI/FEP & Crowd sourcing	[49]
MAT-POS-f24609ef-1	ND*	ND*	Mpro Fluorescence Assay	0.102 μM	ND*	ND*	ND*	ND*	ND*	Hit	AI/FEP & Crowd sourcing	[49]
ADA-UCB-6c2cb422-1	720 nM	ND*	Mpro Fluorescence Assay	4.2 μM	Vero-E6	>50 μM	ND*	ND*	ND*	Hit	AI/FEP & Crowd sourcing	[49]
VLA-UCB-1dbca3b4-15	360 nM	ND*	Mpro Fluorescence Assay	ND*	ND*	>50 μM	ND*	ND*	ND*	Hit	AI/FEP & Crowd sourcing	[50]
PET-UNK-29afea89-2	80 nM	ND*	Mpro Fluorescence Assay	550 nM	Vero-E6	>50 μM	ND*	ND*	ND*	Hit	AI/FEP & Crowd sourcing	[50]
MAT-POS-3ccb8ef6-1	0.28 μM	ND*	Mpro Fluorescence Assay	1.9 μM	Vero-E6	>50 μM	ND*	ND*	ND*	Hit	AI/FEP & Crowd sourcing	[49]
MAT-POS-e194df51-1	0.14 μM	ND*	Mpro Fluorescence Assay	1,65 μM	Vero-E6	>50 μM	ND*	ND*	ND*	Hit	AI/FEP & Crowd sourcing	[49]
MAT-POS-e194df51-1	0.037 μM	ND*	Mpro Fluorescence Assay	0.064 μM	Vero-E6	>50 μM	152	Cyp450 2C9/2	2.5	Lead	AI/FEP & Crowd	[49]

								D6/3A4			sourcing	
112	21.3 nM	ND*	Mpro FRET-based enzymatic assay	ND*	ND*	ND*	1100	ND*	4.1	Hit-Lead	AI-generated Diazepane Scaffold	[51]
119	15.7 nM	ND*	Mpro FRET-based enzymatic assay	ND*	ND*	ND*	2011	ND*	4.0	Lead	AI-generated Diazepane Scaffold	[51]
121	51.2 nM	ND*	Mpro FRET-based enzymatic assay	ND*	ND*	ND*	451	ND*	3.9	Hit-Lead	AI-generated Diazepane Scaffold	[51]
123	74.4 nM	ND*	Mpro FRET-based enzymatic assay	ND*	ND*	ND*	633	ND*	4.2	Hit-Lead	AI-generated Diazepane Scaffold	[51]
2c	ND*	2.95 μM	Mpro FRET-based enzymatic assay	10.3 μM	Huh-7-ACE2	>100 μM	ND*	ND*	ND*	Hit	γ-lactam glutamine derivatives harbouring a methyl vinyl ketone	[52]
2f	ND*	1.61 μM	Mpro FRET-based enzymatic assay	ND*	Huh-7-ACE2	12.6 μM	ND*	ND*	ND*	Hit	γ-lactam glutamine derivatives harbouring a methyl vinyl ketone	[52]
3f	ND*	3.66 μM	Mpro FRET-based enzymatic assay	ND*	Huh-7-ACE2	22.6 μM	ND*	ND*	ND*	Hit	γ-lactam glutamine derivatives harbouring a methyl vinyl ketone	[52]
4b	ND*	5.58 μM	Mpro FRET-based enzymatic assay	7.63 μM	Huh-7-ACE2	> 100 μM	ND*	ND*	ND*	Hit	γ-lactam glutamine derivatives harbouring a methyl vinyl ketone	[52]

4d	ND*	5.54 μM	Mpro FRET-based enzymatic assay	3.50 μM	Huh-7-ACE2	> 100 μM	ND*	ND*	ND*	Hit	γ-lactam glutamine derivatives harbouring a methyl vinyl ketone	[52]
4e	ND*	10.72 μM	Mpro FRET-based enzymatic assay	ND*	Huh-7-ACE2	> 100 μM	ND*	ND*	ND*	Hit	γ-lactam glutamine derivatives harbouring a methyl vinyl ketone	[52]
4f	ND*	3.14 μM	Mpro FRET-based enzymatic assay	ND*	Huh-7-ACE2	46.9 μM	ND*	ND*	ND*	Hit	γ-lactam glutamine derivatives harbouring a methyl vinyl ketone	[52]
SPR39	ND*	1.5 ± 0.3 μM	Enzymatic assay	ND*	Huh-7-ACE2 cells	100 μM	ND*	ND*	ND*	Experimental	Peptide-based vinyl methyl ketones	[45]
SPR35	ND*	1.77 ± 0.16 μM	Enzymatic assay	ND*	ND*	ND*	ND*	ND*	ND*	Hit	Peptide-based Michael acceptors	[45]
SPR36	ND*	33 ± 2% μM	Enzymatic assay	ND*	ND*	ND*	ND*	ND*	ND*	Hit	Peptide-based Michael acceptors	[45]
SPR37	ND*	0.386 ± 0.055 μM	Enzymatic assay	ND*	ND*	ND*	ND*	ND*	ND*	Hit	Peptide-based Michael acceptors	[45]
SPR38	ND*	0.260 ± 0.066	Enzymatic assay	18.5 ± 6.5 μM	Huh-7-ACE2	60.9 ± 11.5 μM	ND*	ND*	ND*	Hit	Peptide-based Michael acceptors	[45]
SPR39	ND*	0.252 ± 0.028 μM	Enzymatic assay	1.5 ± 0.3 μM	Huh-7-ACE3	100 μM	ND*	ND*	ND*	Hit	Peptide-based Michael acceptors	[45]
SPR40	ND*	0.416 ± 0.058 μM	Enzymatic assay	ND*	ND*	ND*	ND*	ND*	ND*	Hit	Peptide-based Michael acceptors	[45]

SPR41	ND*	0.184 ± 0.025	Enzymatic assay	1.8 ± 0.1 µM	Huh-7-ACE2	14.5 ± 3.4 µM	ND*	ND*	ND*	Hit	Peptide-based Michael acceptors	[45]
SPR42	ND*	29 ± 4% µM	Enzymatic assay	ND*	ND*	ND*	ND*	ND*	ND*	Hit	Peptide-based Michael acceptors	[45]
SPR43	ND*	50 ± 5% µM	Enzymatic assay	ND*	ND*	ND*	ND*	ND*	ND*	Hit	Peptide-based Michael acceptors	[45]
SPR44	ND*	47 ± 2% µM	Enzymatic assay	ND*	ND*	ND*	ND*	ND*	ND*	Hit	Peptide-based Michael acceptors	[45]
11a	ND*	0.008 ± 0.0009 µM	Enzymatic assay	ND*	ND*	ND*	ND*	ND*	ND*	ND*	Peptide-based vinyl methyl ketones	[45]
1	0.021 µM	ND*	Enzymatic assay	ND*	ND*	ND*	ND*	ND*	ND*	Hit	Novel dithiocarbamates	[53]
1A	35.000 µM	ND*	Enzymatic assay	ND*	ND*	ND*	ND*	ND*	ND*	Hit	Novel dithiocarbamates	[53]
1B	0.178 µM	ND*	Enzymatic assay	ND*	ND*	ND*	ND*	ND*	ND*	Hit	Novel dithiocarbamates	[53]
1C	0.023 µM	ND*	Enzymatic assay	ND*	ND*	ND*	ND*	ND*	ND*	Hit	Novel dithiocarbamates	[53]
1D	>100 µM	ND*	Enzymatic assay	ND*	ND*	ND*	ND*	ND*	ND*	Hit	Novel dithiocarbamates	[53]
1x	0.027 µM	ND*	Enzymatic assay	ND*	ND*	ND*	ND*	ND*	ND*	Hit	Novel dithiocarbamates	[53]
1	21.4 µM	ND*	Biochemical assay	ND*	ND*	ND*	ND*	ND*	ND*	Hit	Acrylamide fragment	[54]
2	10 µM	ND*	Biochemical assay	ND*	ND*	ND*	ND*	ND*	ND*	Experimental	Acrylamide fragment	[53]
3	56.6 µM	ND*	Biochemical assay	ND*	ND*	ND*	ND*	ND*	ND*	Hit	Acrylamide fragment	[53]
4	20.2 µM	ND*	Biochemical assay	ND*	ND*	ND*	ND*	ND*	ND*	Hit	Acrylamide fragment	[53]
5	17 µM	ND*	Biochemical assay	ND*	ND*	ND*	ND*	ND*	ND*	Experimental	Acrylamide fragment	[53]

6	ND*	ND*	ND*	ND*	ND*	ND*	ND*	ND*	ND*	Hit	Acrylamide fragment	[53]
7	ND*	ND*	ND*	ND*	ND*	ND*	ND*	ND*	ND*	Hit	Acrylamide fragment	[53]
Schaftoside	1.73 ± 0.22 µM	ND*	Enzymatic assay	11.83 ± 3.23 µM	Vero E6	>200 µM	ND*	ND*	ND*	experimental	Plant based	[55]
vicenin-2	1.43 ± 0.20 µM	ND*	Enzymatic assay	ND*	ND*	ND*	ND*	ND*	ND*	Hit	Plant based	[55]
licoisoflavanone	1.52 ± 0.14 µM	ND*	Enzymatic assay	20.26 ± 1.98 µM	Vero E6	84.45 ± 5.26 µM	ND*	ND*	ND*	Hit	Plant based	[55]
GC376	1.01 ± 0.41 µM	ND*	Enzymatic assay	ND*	ND*	ND*	ND*	ND*	ND*	Control	Clinical candidate	[55]
GD-3	1.60 ± 0.2 µM	ND*	Enzymatic assay	183.0 ± 20.6 nM	Vero E6	>100 µM	ND*	ND*	ND*	Experimental	Nonpeptidic Piperazine Derivatives	[56]
GD-8	0.18 ± 0.01 µM	ND*	Enzymatic assay	2.64 ± 0.62 µM	Vero E6	12.5 ± 2.1 µM	ND*	ND*	ND*	Hit	Nonpeptidic Piperazine Derivatives	[56]
GD-12	0.31 ± 0.02 µM	ND*	Enzymatic assay	4.24 ± 0.72 µM	Vero E6	6.50 ± 1.65 µM	ND*	ND*	ND*	Hit	Nonpeptidic Piperazine Derivatives	[56]
GD-24	2.8 ± 0.1 µM	ND*	Enzymatic assay	1.37 ± 0.43 µM	Vero E6	11.9 ± 2.8 µM	ND*	ND*	ND*	Hit	Nonpeptidic Piperazine Derivatives	[56]
Pimozide	42 ± 2 µM	ND*	FRET-based assay	ND*	ND*	ND*	ND*	ND*	ND*	Hit	Repurposed	[57]
Ebastine	57 ± 12 µM	ND*	FRET-based assay	ND*	ND*	ND*	ND*	ND*	ND*	Hit	Repurposed	[57]
Rupintrivir	68 ± 7 µM	ND*	FRET-based assay	ND*	ND*	ND*	ND*	ND*	ND*	Hit	Repurposed	[57]
Bepridil	72 ± 3 µM	ND*	FRET-based assay	0.86 µM	Vero E6	ND*	ND*	ND*	ND*	Experimental	Repurposed	[57]
Sertaconazole	76 ± 2 µM	ND*	FRET-based assay	ND*	ND*	ND*	ND*	ND*	ND*	Hit	Repurposed	[57]
Rimonabant	85 ± 3 µM	ND*	FRET-based assay	ND*	ND*	ND*	ND*	ND*	ND*	Hit	Repurposed	[57]
Oxiconazole	99 ± 6 µM	ND*	FRET-based assay	ND*	ND*	ND*	ND*	ND*	ND*	Hit	Repurposed	[57]
Itraconazole	111 ± 35 µM	ND*	FRET-based assay	ND*	ND*	ND*	ND*	ND*	ND*	Hit	Repurposed	[57]

Tipranavir	180 ± 20 µM	ND*	FRET-based assay	ND*	ND*	ND*	ND*	ND*	ND*	Hit	Repurposed	[57]
Nelfinavir	234 ± 15 µM	ND*	FRET-based assay	ND*	ND*	ND*	ND*	ND*	ND*	Hit	Repurposed	[57]
Zopiclone	349 ± 77 µM	ND*	FRET-based assay	ND*	ND*	ND*	ND*	ND*	ND*	Hit	Repurposed	[57]
Trihexyphenidyl	370 ± 53 µM	ND*	FRET-based assay	ND*	ND*	ND*	ND*	ND*	ND*	Hit	Repurposed	[57]
Saquinavir	411 ± 6 µM	ND*	FRET-based assay	ND*	ND*	ND*	ND*	ND*	ND*	Hit	Repurposed	[57]
Isavuconazole	438 ± 11 µM	ND*	FRET-based assay	ND*	ND*	ND*	ND*	ND*	ND*	Hit	Repurposed	[57]
Lopinavir	486 ± 2 µM	ND*	FRET-based assay	ND*	ND*	ND*	ND*	ND*	ND*	Hit	Repurposed	[57]
Clemastine	497 ± 148 µM	ND*	FRET-based assay	ND*	ND*	ND*	ND*	ND*	ND*	Hit	Repurposed	[57]
Metixene	635 ± 43 µM	ND*	FRET-based assay	ND*	ND*	ND*	ND*	ND*	ND*	Hit	Repurposed	[57]
Duloxetine	3,047 ± 634 µM	ND*	FRET-based assay	ND*	ND*	ND*	ND*	ND*	ND*	Hit	Repurposed	[57]
Boceprevir	5.24 ± 0.20 µM	ND*	Enzymatic assay	ND*	ND*	ND*	ND*	ND*	ND*	FDA-approved	Compounds with electrophilic moieties	[30]
Narlaprevir	2.30 ± 0.38 µM	ND*	Enzymatic assay	ND*	ND*	ND*	ND*	ND*	ND*	In clinical trials	Compounds with electrophilic moieties	[30]
Telaprevir	14.15 ± 1.94 µM	ND*	Enzymatic assay	ND*	ND*	ND*	ND*	ND*	ND*	FDA-approved	Compounds with electrophilic moieties	[30]
Bardoxolone methyl	5.81 ± 0.79 µM	ND*	Enzymatic assay	0.29 µM	Vero	6.94 µM	Vero E6	ND*	ND*	Phase 2 (NCT04494646)	Compounds with electrophilic moieties	[30]
Bardoxolone	27.99 ± 2.34 µM	ND*	Enzymatic assay	0.43 µM	Vero	24.36 µM	Vero E6	ND*	ND*	Experimental	Compounds with electrophilic moieties	[30]
Tideglusib	1.91 ± 0.16 µM	ND*	Enzymatic assay	ND*	ND*	ND*	ND*	ND*	ND*	In clinical trials	Compounds with electrophilic moieties	[30]

Domatinostat tosylate	7.31 ± 0.86	ND*	Enzymatic assay	ND*	ND*	ND*	ND*	ND*	ND*	In clinical trials	Compounds with electrophilic moieties	[30]
WP1066	16.61 ± 1.92	ND*	Enzymatic assay	ND*	ND*	ND*	ND*	ND*	ND*	Veterinary	Compounds with electrophilic moieties	[30]
Ceftiofur hydrochloride	29.55 ± 1.80	ND*	Enzymatic assay	ND*	ND*	ND*	ND*	ND*	ND*	Preclinical	Compounds with electrophilic moieties	[30]
Z-DEVD-FMK	0.31 ± 0.03	ND*	Enzymatic assay	ND*	ND*	ND*	ND*	ND*	ND*	Preclinical	Compounds with electrophilic moieties	[30]
MG132	1.64 ± 0.18	ND*	Enzymatic assay	ND*	ND*	ND*	ND*	ND*	ND*	Preclinical	Compounds with electrophilic moieties	[30]
Bay 11-7085	2.95 ± 0.10	ND*	Enzymatic assay	ND*	ND*	ND*	ND*	ND*	ND*	Preclinical	Compounds with electrophilic moieties	[30]
0600-5807	4.80 ± 0.12	ND*	Enzymatic assay	ND*	ND*	ND*	ND*	ND*	ND*	Preclinical	Compounds with electrophilic moieties	[30]
6H05	9.18 ± 0.52	ND*	Enzymatic assay	ND*	ND*	ND*	ND*	ND*	ND*	Preclinical	Compounds with electrophilic moieties	[30]
NSC129010	27.35 ± 1.71	ND*	Enzymatic assay	ND*	ND*	ND*	ND*	ND*	ND*	Preclinical	Compounds with electrophilic moieties	[30]
9a	1.66 ± 0.02 μM	ND*	Enzymatic assay	15.7 ± 8.3 μM	Vero E6	ND*	ND*	ND*	ND*	Experimental	α-ketoamides	[58]
9b	0.144 ± 0.02 μM	ND*	Enzymatic assay	>100 μM	Vero E6	ND*	ND*	ND*	ND*	Hit	α-ketoamides	[58]
9c	0.10 ± 0.002 μM	ND*	Enzymatic assay	>100 μM	Vero E6	ND*	ND*	ND*	ND*	Hit	α-ketoamides	[58]

9d	0.21 ± 0.02 μM	ND*	Enzymatic assay	>100 μM	Vero E6	ND*	ND*	ND*	ND*	Hit	α-ketoamides	[58]
9e	2.63 ± 0.11 μM	ND*	Enzymatic assay	>100 μM	Vero E6	ND*	ND*	ND*	ND*	Hit	α-ketoamides	[58]
9f	1.93 ± 0.16 μM	ND*	Enzymatic assay	>100 μM	Vero E6	ND*	ND*	ND*	ND*	Hit	α-ketoamides	[58]
9g	1.71 ± 0.14 μM	ND*	Enzymatic assay	>100 μM	Vero E6	ND*	ND*	ND*	ND*	Hit	α-ketoamides	[58]
9h	>30 μM	ND*	Enzymatic assay	>100 μM	Vero E6	ND*	ND*	ND*	ND*	Hit	α-ketoamides	[58]
9i	0.026 ± 0.002 μM	ND*	Enzymatic assay	>100 μM	Vero E6	ND*	ND*	ND*	ND*	Hit	α-ketoamides	[58]
9j	0.03 ± 0.003 μM	ND*	Enzymatic assay	>100 μM	Vero E6	ND*	ND*	ND*	ND*	Hit	α-ketoamides	[58]
GC376	0.0005 ± 0.00003 μM	ND*	Enzymatic assay	4.32 ± 1.33 μM	Vero E6	ND*	ND*	ND*	ND*	Control	Control	[58]
AG7404	47 ± 1.05 μM	ND*	Biochemical assay	6.8 μM	A549-ACE2	250 μM	ND*	ND*	ND*	Experimental	orally bioavailable enterovirus	[59]
Rupintrivir	101 ± 1.40 μM	ND*	Biochemical assay	8.46 μM	A549-ACE2	>250 μM	ND*	ND*	ND*	Hit	orally bioavailable enterovirus	[59]
Remdesivir	ND*	ND*	Biochemical assay	0.145 μM	A549-ACE2	>250 μM	ND*	ND*	ND*	FDA-approved	orally bioavailable enterovirus	[59]
Compound 1	ND*	ND*	ND*	ND*	DENV 2proHeLa	>100	ND*	ND*	ND*	Hit	benzoxaborole based inhibitors	[60]
Compound 2	ND*	ND*	ND*	ND*	DENV 2proHeLa	>100	ND*	ND*	ND*	Hit	benzoxaborole based inhibitors	[60]
Compound 3	ND*	ND*	ND*	ND*	DENV 2proHeLa	>100 μM	ND*	ND*	ND*	Hit	benzoxaborole based inhibitors	[60]
Compound 4	10.0 ± 0.5 μM	ND*	Enzymatic assay	ND*	DENV 2proHeLa	>100 μM	ND*	ND*	ND*	Hit	benzoxaborole based inhibitors	[60]
Compound 5	37.2 ± 12.4 μM	ND*	Enzymatic assay	19.2 ± 5.3 μM	DENV 2proHeLa	>200 μM	ND*	ND*	ND*	Hit	benzoxaborole based inhibitors	[60]
Compound 6	ND*	ND*	ND*	ND*	DENV 2proHeLa	>50 μM	ND*	ND*	ND*	Hit	benzoxaborole based inhibitors	[60]

Compound 7	ND*	ND*	ND*	ND*	DENV 2proHeLa	>200 µM	ND*	ND*	ND*	Hit	benzoxaborole based inhibitors	[60]
Compound 8	13.9 ± 0.7 µM	ND*	Enzymatic assay	ND*	DENV 2proHeLa	>200 µM	ND*	ND*	ND*	Hit	benzoxaborole based inhibitors	[60]
Compound 9	ND*	ND*	ND*	6.4 ± 1.8 µM	DENV 2proHeLa	>100 µM	ND*	ND*	ND*	Hit	benzoxaborole based inhibitors	[60]
Compound 10	ND*	ND*	ND*	ND*	DENV 2proHeLa	>100 µM	ND*	ND*	ND*	Hit	benzoxaborole based inhibitors	[60]
Compound 11	ND*	ND*	ND*	9.2 ± 2.0 µM	DENV 2proHeLa	>50 µM	ND*	ND*	ND*	Hit	benzoxaborole based inhibitors	[60]
Compound 12	ND*	ND*	ND*	>50	DENV 2proHeLa	>50 µM	ND*	ND*	ND*	Hit	benzoxaborole based inhibitors	[60]
Compound 13	ND*	ND*	ND*	22.5 ± 2.5 µM	DENV 2proHeLa	>200 µM	ND*	ND*	ND*	Hit	benzoxaborole based inhibitors	[60]
Compound 14	ND*	ND*	ND*	4.0 ± 0.7	DENV 2proHeLa	>50 µM	ND*	ND*	ND*	Hit	benzoxaborole based inhibitors	[60]
Compound 15	ND*	ND*	ND*	3.5 ± 0.4	DENV 2proHeLa	>50 µM	ND*	ND*	ND*	Hit	benzoxaborole based inhibitors	[60]
Compound 16	ND*	ND*	ND*	6.4 ± 1.5	DENV 2proHeLa	>50 µM	ND*	ND*	ND*	Hit	benzoxaborole based inhibitors	[60]
Compound 17	ND*	ND*	ND*	6.1 ± 1.7	DENV 2proHeLa	>50 µM	ND*	ND*	ND*	Hit	benzoxaborole based inhibitors	[60]
Compound 18	6.1 ± 0.5 µM	ND*	Enzymatic assay	2.4 ± 0.1 µM	DENV 2proHeLa	55.2 ± 8.5 µM	ND*	ND*	ND*	Experimental	benzoxaborole based inhibitors	[60]
Compound 19	ND*	ND*	ND*	8.8 ± 1.6 µM	DENV 2proHeLa	>50 µM	ND*	ND*	ND*	Hit	benzoxaborole based inhibitors	[60]
Compound 20	ND*	ND*	ND*	14.5 ± 3.1 µM	DENV 2proHeLa	>50 µM	ND*	ND*	ND*	Hit	benzoxaborole based inhibitors	[60]
Compound 21	ND*	ND*	ND*	3.1 ± 0.8 µM	DENV 2proHeLa	>50 µM	ND*	ND*	ND*	Hit	benzoxaborole based	[60]

											inhibitors	
Compound 22	56.9 ± 6.2 µM	ND*	Enzymatic assay	5.2 ± 0.4 µM	DENV2proHeLa	144.1 ± 5.0 µM	ND*	ND*	ND*	Hit	benzoxaborole based inhibitors	[60]
Compound 23	52.3 ± 4.6 µM	ND*	Enzymatic assay	13.1 ± 2.2 µM	DENV2proHeLa	>200 µM	ND*	ND*	ND*	Hit	benzoxaborole based inhibitors	[60]
Compound 24	ND*	ND*	ND*	17.1 ± 4.7 µM	DENV2proHeLa	>50 µM	ND*	ND*	ND*	Hit	benzoxaborole based inhibitors	[60]
Compound 25	ND*	ND*	ND*	21.6 ± 5.0 µM	DENV2proHeLa	>100 µM	ND*	ND*	ND*	Hit	benzoxaborole based inhibitors	[60]
Tavaborole	ND*	ND*	ND*	ND*	DENV2proHeLa	>50 µM	ND*	ND*	ND*	Hit	benzoxaborole based inhibitors	[60]
Compound 26	ND*	ND*	ND*	12.5 ± 1.9 µM	DENV2proHeLa	>50 µM	ND*	ND*	ND*	Hit	benzoxaborole based inhibitors	[60]
Compound 27	ND*	ND*	ND*	14.0 ± 2.9 µM	DENV2proHeLa	>50 µM	ND*	ND*	ND*	Hit	benzoxaborole based inhibitors	[60]
MB-53	ND*	ND*	ND*	19.6 ± 1.8 µM	DENV2proHeLa	>50 µM	ND*	ND*	ND*	Control	benzoxaborole based inhibitors	[60]
NK-189	ND*	ND*	ND*	0.6 ± 0.1 µM	DENV2proHeLa	>50 µM	ND*	ND*	ND*	Control	benzoxaborole based inhibitors	[60]
boceprevir	17.2 ± 1.5 µM	ND*	Enzymatic assay	ND*	DENV2proHeLa	>50 µM	ND*	ND*	ND*	Control	benzoxaborole based inhibitors	[60]
MAC-5576	1.7 ± 0.0 µM	ND*	Enzymatic assay	ND*	DENV2proHeLa	>50 µM	ND*	ND*	ND*	Control	benzoxaborole based inhibitors	[60]
JMX0207	ND*	ND*	ND*	1.222 µM	A549-hACE2	13.17 µM	ND*	ND*	ND*	Hit	Niclosamide derivatives	[61]
JMX0281	ND*	ND*	ND*	2.078 µM	A549-hACE2	8.32 µM	ND*	ND*	ND*	Hit	Niclosamide derivatives	[61]
JMX0285	ND*	ND*	ND*	3.031 µM	A549-hACE2	6.13 µM	ND*	ND*	ND*	Hit	Niclosamide derivatives	[61]
JMX0286	4.8 µM	ND*	FRET-based enzymatic assay	2.251 µM	A549-hACE2	3.53.1 µM	ND*	ND*	ND*	Experimental	Niclosamide derivatives	[61]

JMX0308	ND*	ND*	ND*	0.8065 μM	A549-hACE2	1.80 μM	ND*	ND*	ND*	Hit	Niclosamide derivatives	[61]
JMX0310	ND*	ND*	ND*	2.414 μM	A549-hACE2	10.06 μM	ND*	ND*	ND*	Hit	Niclosamide derivatives	[61]
JMX0312	ND*	ND*	ND*	1.129 μM	A549-hACE2	1.97 μM	ND*	ND*	ND*	Hit	Niclosamide derivatives	[61]
JMX0317	ND*	ND*	ND*	4.025 μM	A549-hACE2	11.70 μM	ND*	ND*	ND*	Hit	Niclosamide derivatives	[61]
JMX0321	ND*	ND*	ND*	1.186 μM	A549-hACE2	3.76 μM	ND*	ND*	ND*	Hit	Niclosamide derivatives	[61]
JMX0325	ND*	ND*	ND*	0.1228 μM	A549-hACE2	1.81 μM	ND*	ND*	ND*	Hit	Niclosamide derivatives	[61]
JMX0330	ND*	ND*	ND*	0.4981 μM	A549-hACE2	10.15 μM	ND*	ND*	ND*	Hit	Niclosamide derivatives	[61]
JMX0510–2	ND*	ND*	ND*	4.88 μM	A549-hACE2	11.50 μM	ND*	ND*	ND*	Hit	Niclosamide derivatives	[61]
JMX0670	ND*	ND*	ND*	3.202 μM	A549-hACE2	9.17 μM	ND*	ND*	ND*	Hit	Niclosamide derivatives	[61]
JMX0671	ND*	ND*	ND*	1.563 μM	A549-hACE2	8.03 μM	ND*	ND*	ND*	Hit	Niclosamide derivatives	[61]
JMX0672	ND*	ND*	ND*	1.921 μM	A549-hACE2	8.34 μM	ND*	ND*	ND*	Hit	Niclosamide derivatives	[61]
JMX0673	ND*	ND*	FRET-based enzymatic assay	1.743 μM	A549-hACE2	5.20 μM	ND*	ND*	ND*	Hit	Niclosamide derivatives	[61]
JMX0674	ND*	ND*	ND*	9.752 μM	A549-hACE2	>10 μM	ND*	ND*	ND*	Hit	Niclosamide derivatives	[61]
JMX0679	ND*	ND*	ND*	2.514 μM	A549-hACE2	7.14 μM	ND*	ND*	ND*	Hit	Niclosamide derivatives	[61]
JMX0680	ND*	ND*	ND*	4.566 μM	A549-hACE2	10.10 μM	ND*	ND*	ND*	Hit	Niclosamide derivatives	[61]
JMX0681	ND*	ND*	ND*	9.753 μM	A549-hACE2	>10 μM	ND*	ND*	ND*	Hit	Niclosamide derivatives	[61]
JMX0682	ND*	ND*	ND*	1.568 μM	A549-hACE2	6.48 μM	ND*	ND*	ND*	Hit	Niclosamide derivatives	[61]
JMX0887	ND*	ND*	ND*	0.9203 μM	A549-hACE2	4.18 μM	ND*	ND*	ND*	Hit	Niclosamide derivatives	[61]

JMX0895	ND*	ND*	ND*	0.665 μM	A549-hACE2	4.57 μM	ND*	ND*	ND*	Hit	Niclosamide derivatives	[61]
JMX0929	ND*	ND*	ND*	2.425 μM	A549-hACE2	5.06 μM	ND*	ND*	ND*	Hit	Niclosamide derivatives	[61]
JMX0936	ND*	ND*	ND*	2.214 μM	A549-hACE2	3.74 μM	ND*	ND*	ND*	Hit	Niclosamide derivatives	[61]
JMX0940	ND*	ND*	ND*	1.216 μM	A549-hACE2	7.72 μM	ND*	ND*	ND*	Hit	Niclosamide derivatives	[61]
JMX0941	3.9 μM	ND*	FRET-based enzymatic assay	1.7 μM	A549-hACE2	3.0 μM	ND*	ND*	ND*	Experimental	Niclosamide derivatives	[61]
JMX0942	ND*	ND*	ND*	2.891 μM	A549-hACE2	7.20 μM	ND*	ND*	ND*	Hit	Niclosamide derivatives	[61]
JMX0301	4.5 μM	ND*	FRET-based enzymatic assay	No dose response curve	A549-hACE2	342.4 μM	ND*	ND*	ND*	Experimental	Niclosamide derivatives	[61]
Boceprevir	4.9 μM	ND*	FRET-based enzymatic assay	15.6 μM	A549-hACE2	>200 μM	ND*	ND*	ND*	Control	Clinical trial	[61]
10a	3.889±0.051 μM	ND*	Enzymatic assay	>3.00 μM	VeroE6	>100	ND*	ND*	ND*	Hit	Tripeptidomimetic inhibitors	[61]
10b	0.374±0.006 μM	ND*	Enzymatic assay	1.06 μM	VeroE6	>100	ND*	ND*	ND*	Experimental	Tripeptidomimetic inhibitors	[61]
10c	0.373±0.011 μM	ND*	Enzymatic assay	1.11 μM	VeroE6	>100	ND*	ND*	ND*	Hit	Tripeptidomimetic inhibitors	[61]
10d	0.277±0.008 μM	ND*	Enzymatic assay	>3.00 μM	VeroE6	>100	ND*	ND*	ND*	Hit	Tripeptidomimetic inhibitors	[61]
10e	12.32±0.59 μM	ND*	Enzymatic assay	>3.00 μM	VeroE6	>100	ND*	ND*	ND*	Hit	Tripeptidomimetic inhibitors	[61]
10f	6.928±0.340 μM	ND*	Enzymatic assay	>3.00 μM	VeroE6	>100	ND*	ND*	ND*	Hit	Tripeptidomimetic inhibitors	[61]
10g	0.321±0.006 μM	ND*	Enzymatic assay	1.51 μM	VeroE6	>100	ND*	ND*	ND*	Hit	Tripeptidomimetic inhibitors	[61]
10h	3.436±0.034 μM	ND*	Enzymatic assay	>3.00 μM	VeroE6	>100	ND*	ND*	ND*	Hit	Tripeptidomimetic inhibitors	[61]

10i	0.298±0.014 µM	ND*	Enzymatic assay	1.13 µM	VeroE6	>100	ND*	ND*	ND*	Hit	Tripeptidomimetic inhibitors	[61]
10j	2.044±0.120 µM	ND*	Enzymatic assay	>3.00 µM	VeroE6	>100	ND*	ND*	ND*	Hit	Tripeptidomimetic inhibitors	[61]
10k	1.338±0.137 µM	ND*	Enzymatic assay	>3.00 µM	VeroE6	>100	ND*	ND*	ND*	Hit	Tripeptidomimetic inhibitors	[61]
10l	3.275±0.033 µM	ND*	Enzymatic assay	>3.00 µM	VeroE6	>100	ND*	ND*	ND*	Hit	Tripeptidomimetic inhibitors	[61]
Positive control	0.858±0.017 µM	ND*	Enzymatic assay	0.36 µM	VeroE6	>100	ND*	ND*	ND*	Control	Control	[61]
14a	0.543±0.008 µM	ND*	Enzymatic assay	>3.00 µM	VeroE6	>100	ND*	ND*	ND*	Hit	Tetrapeptidomimetic inhibitors	[61]
14b	0.357±0.021 µM	ND*	Enzymatic assay	>3.00 µM	VeroE6	>100	ND*	ND*	ND*	Hit	Tetrapeptidomimetic inhibitors	[61]
14c	0.281±0.033 µM	ND*	Enzymatic assay	>3.00 µM	VeroE6	>100	ND*	ND*	ND*	Hit	Tetrapeptidomimetic inhibitors	[61]
14d	1.497±0.101 µM	ND*	Enzymatic assay	1.67 µM	VeroE6	>100	ND*	ND*	ND*	Hit	Tetrapeptidomimetic inhibitors	[61]
17a	0.196±0.004 µM	ND*	Enzymatic assay	>3.00 µM	VeroE6	>100	ND*	ND*	ND*	Hit	Tetrapeptidomimetic inhibitors	[61]
17b	0.230±0.002 µM	ND*	Enzymatic assay	>3.00 µM	VeroE6	>100	ND*	ND*	ND*	Hit	Tetrapeptidomimetic inhibitors	[61]
17c	0.295±0.020 µM	ND*	Enzymatic assay	>3.00 µM	VeroE6	>100	ND*	ND*	ND*	Hit	Tetrapeptidomimetic inhibitors	[61]
17d	0.249±0.010 µM	ND*	Enzymatic assay	>3.00 µM	VeroE6	>100	ND*	ND*	ND*	Hit	Tetrapeptidomimetic inhibitors	[61]
17e	0.592±0.017 µM	ND*	Enzymatic assay	>3.00 µM	VeroE6	>100	ND*	ND*	ND*	Hit	Tetrapeptidomimetic inhibitors	[61]
17f	0.307±0.014 µM	ND*	Enzymatic assay	>3.00 µM	VeroE6	>100	ND*	ND*	ND*	Hit	Tetrapeptidomimetic inhibitors	[61]

17g	0.173±0.014 µM	ND*	Enzymatic assay	>3.00 µM	VeroE6	>100	ND*	ND*	ND*	Hit	Tetrapeptidomimetic inhibitors	[61]
17h	0.255±0.010 µM	ND*	Enzymatic assay	>3.00 µM	VeroE6	>100	ND*	ND*	ND*	Hit	Tetrapeptidomimetic inhibitors	[61]
17i	0.271±0.001 µM	ND*	Enzymatic assay	1.36 µM	VeroE6	>100	ND*	ND*	ND*	Hit	Tetrapeptidomimetic inhibitors	[61]
17j	0.295±0.012 µM	ND*	Enzymatic assay	>3.00 µM	VeroE6	>100	ND*	ND*	ND*	Hit	Tetrapeptidomimetic inhibitors	[61]
17k	0.221±0.001 µM	ND*	Enzymatic assay	>3.00 µM	VeroE6	>100	ND*	ND*	ND*	Hit	Tetrapeptidomimetic inhibitors	[61]
17l	0.199±0.001 µM	ND*	Enzymatic assay	>3.00 µM	VeroE6	>100	ND*	ND*	ND*	Hit	Tetrapeptidomimetic inhibitors	[61]
17m	0.257±0.024 µM	ND*	Enzymatic assay	>3.00 µM	VeroE6	>100	ND*	ND*	ND*	Hit	Tetrapeptidomimetic inhibitors	[61]
17n	0.404±0.003 µM	ND*	Enzymatic assay	>3.00 µM	VeroE6	>100	ND*	ND*	ND*	Hit	Tetrapeptidomimetic inhibitors	[61]
17o	1.409±0.124 µM	ND*	Enzymatic assay	>3.00 µM	VeroE6	>100	ND*	ND*	ND*	Hit	Tetrapeptidomimetic inhibitors	[61]
17p	0.234±0.007 µM	ND*	Enzymatic assay	>3.00 µM	VeroE6	>100	ND*	ND*	ND*	Hit	Tetrapeptidomimetic inhibitors	[61]
17q	0.173±0.013 µM	ND*	Enzymatic assay	>3.00 µM	VeroE6	>100	ND*	ND*	ND*	Hit	Tetrapeptidomimetic inhibitors	[61]
17r	0.172±0.018 µM	ND*	Enzymatic assay	>3.00 µM	VeroE6	>100	ND*	ND*	ND*	Hit	Tetrapeptidomimetic inhibitors	[61]
17s	0.153±0.010 µM	ND*	Enzymatic assay	>3.00 µM	VeroE6	>100	ND*	ND*	ND*	Hit	Tetrapeptidomimetic inhibitors	[61]
17t	0.640±0.009 µM	ND*	Enzymatic assay	>3.00 µM	VeroE6	>100	ND*	ND*	ND*	Hit	Tetrapeptidomimetic inhibitors	[61]
17u	0.565±0.006 µM	ND*	Enzymatic assay	>3.00 µM	VeroE6	>100	ND*	ND*	ND*	Hit	Tetrapeptidomimetic	[61]

											inhibitors	
17v	0.197±0.008 µM	ND*	Enzymatic assay	>3.00 µM	VeroE6	>100	ND*	ND*	ND*	Hit	Tetrapeptidomimetic inhibitors	[61]
17w	0.148±0.004 µM	ND*	Enzymatic assay	>3.00 µM	VeroE6	>100	ND*	ND*	ND*	Hit	Tetrapeptidomimetic inhibitors	[61]
17x	0.209±0.002 µM	ND*	Enzymatic assay	>3.00 µM	VeroE6	>100	ND*	ND*	ND*	Hit	Tetrapeptidomimetic inhibitors	[61]
17y	1.426±0.026 µM	ND*	Enzymatic assay	>3.00 µM	VeroE6	>100	ND*	ND*	ND*	Hit	Tetrapeptidomimetic inhibitors	[61]
17z	0.285±0.006 µM	ND*	Enzymatic assay	>3.00 µM	VeroE6	>100	ND*	ND*	ND*	Hit	Tetrapeptidomimetic inhibitors	[61]
Positive control	0.858±0.017 µM	ND*	Enzymatic assay	0.36 µM	VeroE6	>100	ND*	ND*	ND*	Control	Control	[61]
SAA	2.49 ± 0.57 µM	ND*	Fluorescence-Based TSA	ND*	ND*	ND*	ND*	ND*	ND*	Hit	Repurposed	[62]
EGCG	11.58 ± 1.62 µM	ND*	Fluorescence-Based TSA	ND*	ND*	ND*	ND*	ND*	ND*	Hit	Repurposed	[62]
Oridonin	2.16 ± 0.22 µM	ND*	Fluorescence-Based TSA	4.95 µM	Vero E6	24.94 µM	ND*	ND*	ND*	Experimental	Repurposed	[62]
GC376	ND*	ND*	ND*	1.87 µM	Vero E6	ND*	ND*	ND*	ND*	Control	Repurposed	[62]
MPI3	8.5 nM	ND*	Enzymatic assay	ND*	ND*	ND*	ND*	ND*	ND*	Hit	Aldehyde-based tripeptidyl inhibitors	[63]
MPI4	15 nM	ND*	Enzymatic assay	ND*	ND*	ND*	ND*	ND*	ND*	Hit	Aldehyde-based tripeptidyl inhibitors	[63]
MPI5	33 nM	ND*	Enzymatic assay	ND*	ND*	ND*	ND*	ND*	ND*	Hit	Aldehyde-based tripeptidyl inhibitors	[63]
MPI6	60 nM	ND*	Enzymatic assay	ND*	ND*	ND*	ND*	ND*	ND*	Hit	Aldehyde-based tripeptidyl inhibitors	[63]

MPI7	47 nM	ND*	Enzymatic assay	ND*	ND*	ND*	ND*	ND*	ND*	Hit	Aldehyde-based tripeptidyl inhibitors	[63]
MPI8	105 nM	ND*	Enzymatic assay	0.030 µM	Vero E6	109.2 µM	ND*	ND*	ND*	Experimental	Aldehyde-based tripeptidyl inhibitors	[63]
MPI11	19 nM	ND*	Enzymatic assay	ND*	ND*	ND*	ND*	ND*	ND*	Hit	Aldehyde-based tripeptidyl inhibitors	[63]
MPI12	11 nM	ND*	Enzymatic assay	ND*	ND*	ND*	ND*	ND*	ND*	Hit	Aldehyde-based tripeptidyl inhibitors	[63]
MPI13	4.8 nM	ND*	Enzymatic assay	ND*	ND*	ND*	ND*	ND*	ND*	Hit	Aldehyde-based tripeptidyl inhibitors	[63]
MPI14	5.2 nM	ND*	Enzymatic assay	ND*	ND*	ND*	ND*	ND*	ND*	Hit	Aldehyde-based tripeptidyl inhibitors	[63]
MPI15	ND*	ND*	Enzymatic assay	ND*	ND*	ND*	ND*	ND*	ND*	Hit	Aldehyde-based tripeptidyl inhibitors	[63]
MPI16	150 nM	ND*	Enzymatic assay	1.2 µM	Vero E6	>200 µM	ND*	ND*	ND*	Hit	Aldehyde-based tripeptidyl inhibitors	[63]
MPI17	60 nM	ND*	Enzymatic assay	1.2 µM	Vero E6	>200 µM	ND*	ND*	ND*	Hit	Aldehyde-based tripeptidyl inhibitors	[63]
MPI18	36 nM	ND*	Enzymatic assay	>5	Vero E6	>200 µM	ND*	ND*	ND*	Hit	Aldehyde-based tripeptidyl inhibitors	[63]
MPI19	13 nM	ND*	Enzymatic assay	ND*	ND*	ND*	ND*	ND*	ND*	Hit	Aldehyde-based tripeptidyl	[63]

											inhibitors	
MPI20	16 nM	ND*	Enzymatic assay	ND*	ND*	ND*	ND*	ND*	ND*	Hit	Aldehyde-based tripeptidyl inhibitors	[63]
MPI21	77 nM	ND*	Enzymatic assay	ND*	ND*	ND*	ND*	ND*	ND*	Hit	Aldehyde-based tripeptidyl inhibitors	[63]
MPI22	49 nM	ND*	Enzymatic assay	ND*	ND*	ND*	ND*	ND*	ND*	Hit	Aldehyde-based tripeptidyl inhibitors	[63]
MPI23	350 nM	ND*	Enzymatic assay	ND*	ND*	ND*	ND*	ND*	ND*	Hit	Aldehyde-based tripeptidyl inhibitors	[63]
MPI24	8.4 nM	ND*	Enzymatic assay	ND*	ND*	ND*	ND*	ND*	ND*	Hit	Aldehyde-based tripeptidyl inhibitors	[63]
MPI25	650 nM	ND*	Enzymatic assay	2.2 µM	Vero E6	130.0 µM	ND*	ND*	ND*	Hit	Aldehyde-based tripeptidyl inhibitors	[63]
MPI26	530 nM	ND*	Enzymatic assay	1.9 µM	Vero E6	182.9 µM	ND*	ND*	ND*	Hit	Aldehyde-based tripeptidyl inhibitors	[63]
MPI27	160 nM	ND*	Enzymatic assay	>5 µM	Vero E6	114.0 µM	ND*	ND*	ND*	Hit	Aldehyde-based tripeptidyl inhibitors	[63]
MPI28	52 nM	ND*	Enzymatic assay	ND*	ND*	ND*	ND*	ND*	ND*	Hit	Aldehyde-based tripeptidyl inhibitors	[63]
Nirmatrelvir	66 nM	ND*	Enzymatic assay	ND*	ND*	ND*	ND*	ND*	ND*	Control	Aldehyde-based tripeptidyl inhibitors	[63]
GC376	30 nM	ND*	Enzymatic assay	ND*	ND*	ND*	ND*	ND*	ND*	Control	Aldehyde-based	[63]

											tripeptidyl inhibitors	
Boceprevir	4200 ± 600 nM/800 ± 1500 nM	ND*	Enzymatic assay	ND*	ND*	ND*	ND*	ND*	ND*	Refference	Nirmatelvir-based main protease inhibitors	[64]
MPI29	9.3 ± 0.8 nM	ND*	Enzymatic assay	ND*	ND*	ND*	ND*	ND*	ND*	Hit	Boceprevir-based main protease inhibitors	[64]
MPI30	40 ± 4 nM	ND*	Enzymatic assay	ND*	ND*	ND*	ND*	ND*	ND*	Hit	Boceprevir-based main protease inhibitors	[64]
MPI31	360 ± 50 nM	ND*	Enzymatic assay	ND*	ND*	ND*	ND*	ND*	ND*	Hit	Boceprevir-based main protease inhibitors	[64]
MPI32	620 ± 170 nM	ND*	Enzymatic assay	ND*	ND*	ND*	ND*	ND*	ND*	Hit	Boceprevir-based main protease inhibitors	[64]
MPI33	75 ± 9 nM	ND*	Enzymatic assay	ND*	ND*	ND*	ND*	ND*	ND*	Hit	Boceprevir-based main protease inhibitors	[64]
MPI34	370 ± 30 nM	ND*	Enzymatic assay	ND*	ND*	ND*	ND*	ND*	ND*	Hit	Boceprevir-based main protease inhibitors	[64]
MPI35	720 ± 80 nM	ND*	Enzymatic assay	ND*	ND*	ND*	ND*	ND*	ND*	Hit	Boceprevir-based main protease inhibitors	[64]
MPI36	102 ± 2 nM	ND*	Enzymatic assay	ND*	ND*	ND*	ND*	ND*	ND*	Hit	Boceprevir-based main protease	[64]

											inhibitors	
MPI37	23 ± 1 nM	ND*	Enzymatic assay	ND*	ND*	ND*	ND*	ND*	ND*	Hit	Boceprevir-based main protease inhibitors	[64]
MPI38	17 ± 2 nM	ND*	Enzymatic assay	ND*	ND*	ND*	ND*	ND*	ND*	Hit	Boceprevir-based main protease inhibitors	[64]
MPI39	26 ± 1 nM	ND*	Enzymatic assay	ND*	ND*	ND*	ND*	ND*	ND*	Hit	Boceprevir-based main protease inhibitors	[64]
MPI40	180 ± 20 nM	ND*	Enzymatic assay	ND*	ND*	ND*	ND*	ND*	ND*	Hit	Boceprevir-based main protease inhibitors	[64]
MPI41	150 ± 20 nM	ND*	Enzymatic assay	ND*	ND*	ND*	ND*	ND*	ND*	Hit	Boceprevir-based main protease inhibitors	[64]
MPI42	22 ± 4 nM	ND*	Enzymatic assay	ND*	ND*	ND*	ND*	ND*	ND*	Hit	Boceprevir-based main protease inhibitors	[64]
MPI43	45 ± 5 nM	ND*	Enzymatic assay	0.61 µM	Vero E6	34.2 µM	ND*	ND*	ND*	Experimental	Boceprevir-based main protease inhibitors	[64]
MPI44	59 ± 7 nM	ND*	Enzymatic assay	2.94 µM	Vero E6	143.7 µM	ND*	ND*	ND*	Experimental	Boceprevir-based main protease inhibitors	[64]
MPI46	120 ± 10 nM	ND*	Enzymatic assay	1.08 µM	Vero E6	163.4 µM	ND*	ND*	ND*	Experimental	Boceprevir-based main protease inhibitors	[64]

MPI47	720 ± 90 nM	ND*	Enzymatic assay	ND*	ND*	ND*	ND*	ND*	ND*	Hit	Boceprevir-based main protease inhibitors	[64]
PF-07321332	66 ± 12 nM	ND*	Enzymatic assay	1.3 μM	Vero E6	ND*	ND*	ND*	ND*	Control	Boceprevir-based main protease inhibitors	[64]
Leupeptin	127.2 μM	ND*	Enzymatic assay	42.34 μM	Vero	ND*		ND*	ND*	Hit	Plant-based	[65]
Tideglusib	0.30 ± 0.02 μM	ND*	Enzymatic assay	ND*	ND*	ND*	ND*	ND*	ND*	Hit	Analogs of F8	[66]
F8	21.28 ± 0.89 μM	ND*	Enzymatic assay	ND*	ND*	ND*	ND*	ND*	ND*	Hit	Analogs of F8	[66]
F8–S22	25.02 ± 1.11 μM	ND*	Enzymatic assay	ND*	ND*	ND*	ND*	ND*	ND*	Hit	Analogs of F8	[66]
F8–S24	17.50 ± 1.65 μM	ND*	Enzymatic assay	ND*	ND*	ND*	ND*	ND*	ND*	Hit	Analogs of F8	[66]
F8–S40	10.88 ± 0.16 μM	ND*	Enzymatic assay	ND*	ND*	ND*	ND*	ND*	ND*	Hit	Analogs of F8	[66]
F8–S43	10.76 ± 0.48 μM	ND*	Enzymatic assay	ND*	ND*	ND*	ND*	ND*	ND*	Hit	Analogs of F8	[66]
F8–S43	10.76 ± 0.48 μM	ND*	Enzymatic assay	ND*	ND*	ND*	ND*	ND*	ND*	Hit	Analogs of F8–S43	[66]
F8–S43–S2	8.08 ± 0.38 μM	ND*	Enzymatic assay	ND*	ND*	ND*	ND*	ND*	ND*	Hit	Analogs of F8–S43	[66]
F8–S43–S3	9.69 ± 0.45 μM	ND*	Enzymatic assay	ND*	ND*	ND*	ND*	ND*	ND*	Hit	Analogs of F8–S43	[66]
F8-A1 (F8–S40)	10.88 ± 0.16 μM	ND*	Enzymatic assay	ND*	ND*	ND*	ND*	ND*	ND*	Hit	Analogues of F8-A1 to F8-A9	[66]
F8-A2	17.53 ± 3.98 μM	ND*	Enzymatic assay	ND*	ND*	ND*	ND*	ND*	ND*	Hit	Analogues of F8-A1 to F8-A9	[66]
F8-A2	17.53 ± 3.98 μM	ND*	Enzymatic assay	ND*	ND*	ND*	ND*	ND*	ND*	Hit	Analogues of F8–B1 to F8–B22	[66]
F8–B1	4.00 ± 0.25 μM	ND*	Enzymatic assay	ND*	ND*	ND*	ND*	ND*	ND*	Hit	Analogues of F8–B1	[66]

											to F8–B22	
F8–B2	5.39 ± 0.14 μM	ND*	Enzymatic assay	ND*	ND*	ND*	ND*	ND*	ND*	Hit	Analogues of F8–B1 to F8–B22	[66]
F8–B3	4.05 ± 0.26 μM	ND*	Enzymatic assay	ND*	ND*	ND*	ND*	ND*	ND*	Hit	Analogues of F8–B1 to F8–B22	[66]
F8–B4	8.14 ± 1.14 μM	ND*	Enzymatic assay	ND*	ND*	ND*	ND*	ND*	ND*	Hit	Analogues of F8–B1 to F8–B22	[66]
F8–B5	4.69 ± 0.34 μM	ND*	Enzymatic assay	ND*	ND*	ND*	ND*	ND*	ND*	Hit	Analogues of F8–B1 to F8–B22	[66]
F8–B6	1.57 ± 0.08 μM	ND*	Enzymatic assay	ND*	ND*	ND*	ND*	ND*	ND*	Experimental	Analogues of F8–B1 to F8–B22	[66]
F8–B7	32.53 ± 2.33 μM	ND*	Enzymatic assay	ND*	ND*	ND*	ND*	ND*	ND*	Hit	Analogues of F8–B1 to F8–B22	[66]
F8–B8	40.59 ± 1.06 μM	ND*	Enzymatic assay	ND*	ND*	ND*	ND*	ND*	ND*	Hit	Analogues of F8–B1 to F8–B22	[66]
F8–B9	25.39 ± 2.05 μM	ND*	Enzymatic assay	ND*	ND*	ND*	ND*	ND*	ND*	Hit	Analogues of F8–B1 to F8–B22	[66]
F8–B10	10.00 ± 0.50 μM	ND*	Enzymatic assay	ND*	ND*	ND*	ND*	ND*	ND*	Hit	Analogues of F8–B1 to F8–B22	[66]
F8–B12	9.09 ± 1.05 μM	ND*	Enzymatic assay	ND*	ND*	ND*	ND*	ND*	ND*	Hit	Analogues of F8–B1 to F8–B22	[66]
F8–B13	4.14 ± 0.16 μM	ND*	Enzymatic assay	ND*	ND*	ND*	ND*	ND*	ND*	Hit	Analogues of F8–B1 to F8–B22	[66]
F8–B14	5.81 ± 0.07 μM	ND*	Enzymatic assay	ND*	ND*	ND*	ND*	ND*	ND*	Hit	Analogues of F8–B1 to F8–B22	[66]
F8–B16	7.09 ± 0.51 μM	ND*	Enzymatic assay	ND*	ND*	ND*	ND*	ND*	ND*	Hit	Analogues of F8–B1 to F8–B22	[66]
F8–B17	8.76 ± 0.75 μM	ND*	Enzymatic assay	ND*	ND*	ND*	ND*	ND*	ND*	Hit	Analogues of F8–B1 to F8–B22	[66]
F8–B20	10.40 ± 2	ND*	Enzymatic assay	ND*	ND*	ND*	ND*	ND*	ND*	Hit	Analogues of	[66]

	.20 µM										F8–B1 to F8–B22	
F8–B21	10.30 ± 0.70 µM	ND*	Enzymatic assay	ND*	ND*	ND*	ND*	ND*	ND*	Hit	Analogues of F8–B1 to F8–B22	[66]
F8–B22	1.55 ± 0.08 µM	ND*	Enzymatic assay	ND*	ND*	ND*	ND*	ND*	ND*	Hit	Analogues of F8–B1 to F8–B22	[66]
F8-A2	17.53 ± 3.98 µM	ND*	Enzymatic assay	ND*	ND*	ND*	ND*	ND*	ND*	Hit	F8–C1 to F8–C4.	[66]
F8–C1	24.56 ± 0.70 µM	ND*	Enzymatic assay	ND*	ND*	ND*	ND*	ND*	ND*	Hit	F8–C1 to F8–C4.	[66]
F8–C2	5.78 ± 0.30 µM	ND*	Enzymatic assay	ND*	ND*	ND*	ND*	ND*	ND*	Hit	F8–C1 to F8–C4.	[66]
F8–C3	5.44 ± 0.16 µM	ND*	Enzymatic assay	ND*	ND*	ND*	ND*	ND*	ND*	Hit	F8–C1 to F8–C4.	[66]
X77(rac)	2.8 ± 0.6 µM	ND*	Hydrolysis assay	ND*	ND*	ND*	ND*	ND*	ND*	Control	Refference	[67]
MLI(rac)	6.7 ± 1.3	ND*	Hydrolysis assay	14.1 ± 0.5 µM	ND*	ND*	ND*	ND*	ND*	Control	Refference	[67]
1	26 ± 5 %	ND*	Hydrolysis assay	ND*	ND*	ND*	ND*	ND*	ND*	Hit	Enamine compounds	[67]
2	55 ± 2 %	ND*	Hydrolysis assay	ND*	ND*	ND*	ND*	ND*	ND*	Hit	Enamine compounds	[67]
3	63 ± 3 %	ND*	Hydrolysis assay	ND*	ND*	ND*	ND*	ND*	ND*	Hit	Enamine compounds	[67]
4	101 ± 1 ~~%~~	ND*	Hydrolysis assay	ND*	ND*	ND*	ND*	ND*	ND*	Hit	Enamine compounds	[67]
5	20 ± 1.4 µM	ND*	Hydrolysis assay	ND*	ND*	ND*	ND*	ND*	ND*	Hit	Enamine compounds	[67]
6	44 ± 4 %	ND*	Hydrolysis assay	ND*	ND*	ND*	ND*	ND*	ND*	Hit	Enamine compounds	[67]
7	54 ± 2 %	ND*	Hydrolysis assay	ND*	ND*	ND*	ND*	ND*	ND*	Hit	Enamine compounds	[67]
8	61 ± 3 %	ND*	Hydrolysis assay	ND*	ND*	ND*	ND*	ND*	ND*	Hit	Enamine compounds	[67]
9	67 ± 1 ~~%~~	ND*	Hydrolysis assay	ND*	ND*	ND*	ND*	ND*	ND*	Hit	Enamine compounds	[67]
10	18 µM	ND*	Hydrolysis assay	ND*	ND*	ND*	ND*	ND*	ND*	Hit	S1 optimization	[67]

11	7.5 µM	ND*	Hydrolysis assay	ND*	ND*	ND*	ND*	ND*	ND*	Hit	S1 optimization	[67]
12	1.3 µM	ND*	Hydrolysis assay	ND*	ND*	ND*	ND*	ND*	ND*	Hit	S1 optimization	[67]
16	0.46 µM	ND*	Hydrolysis assay	ND*	Vero E6	>20 µM	ND*	ND*	ND*	Hit	S1 optimization	[67]
16a	ND*	ND*	ND*	0.95 ± 0.10 µM	Vero E6	ND*	ND*	ND*	ND*	Hit	Derivative of compound 16	[67]
16b	ND*	ND*	ND*	16 ± 1 µM	Vero E6	ND*	ND*	ND*	ND*	Hit	Derivative of compound 17	[67]
13	79 µm	ND*	Hydrolysis assay	ND*	ND*	ND*	ND*	ND*	ND*	Hit	S2 optimization	[67]
14	8.3 µM	ND*	Hydrolysis assay	ND*	ND*	ND*	ND*	ND*	ND*	Hit	S2 optimization	[67]
15	6.9 µM	ND*	Hydrolysis assay	ND*	ND*	ND*	ND*	ND*	ND*	Hit	S2 optimization	[67]
17	0.33 µM	ND*	Hydrolysis assay	ND*	Vero E6	>20 µM	ND*	ND*	ND*	Hit	S2 optimization	[67]
17a	ND*	ND*	ND*	1.5 ± 0.1 µM	Huh7	ND*	ND*	ND*	ND*	Hit	Derivative of compound 17	[67]
17b	ND*	ND*	ND*	1.4 ± 0.1 µM	Huh7	ND*	ND*	ND*	ND*	Hit	Derivative of compound 18	[67]
21	2.1 µM	ND*	Hydrolysis assay	ND*	ND*	ND*	ND*	ND*	ND*	Hit	Merge (8 +5)	[67]
18	0.39 µM	ND*	Hydrolysis assay	ND*	ND*	ND*	ND*	ND*	ND*	Hit	Merge (8 +5)	[67]
19	0.077 µM	ND*	Hydrolysis assay	0.11 ± 0.03 µM	Vero E6	>20 µM	ND*	ND*	ND*	Experimental	Merge (8 +5)	[67]
GC376	ND*	ND*	ND*	4.4 ± 0.3 µM	Vero E6	ND*	ND*	ND*	ND*	Control	Clinical candidate	[67]
PF-07321332	ND*	ND*	ND*	0.08 ± 0.03 µM	Vero E6/Huh 7	>100 µM	ND*	ND*	ND*	Control	FDA approved	[67]
Y180	8.1 nM	ND*	Enzyme kinetics assay	11.4 nM	VeroE6-TMPRSS2	>81 µM	ND*	ND*	ND*	Experimental	α-ketoamide-containing	[68]
F01	54 µM	ND*	Enzymatic assay	150 µM	Vero-81	>400 µM	ND*	ND*	ND*	Experimental	Fragment - based	[69]
2a	0.18 ± 0.03 µM	ND*	Biochemical assay	0.035 ± 0.001 µM	Vero E6	> 100 µM	ND*	ND*	ND*	Experimental	Aldehyde warhead	[70]
3a	0.17 ± 0.02 µM	ND*	Biochemical assay	0.032 ± 0.001 µM	Vero E6	> 100 µM	ND*	ND*	ND*	Experimental	Aldehyde warhead	[70]
2b	0.29 ±	ND*	Biochemical assay	ND*	Vero E6	> 100 µM	ND*	ND*	ND*	Hit	Aldehyde	[70]

	0.05 µM										warhead	
3b	0.29 ± 0.01 µM	ND*	Biochemical assay	ND*	Vero E6	> 100 µM	ND*	ND*	ND*	Hit	Aldehyde warhead	[70]
2c	0.31 ± 0.06 µM	ND*	Biochemical assay	ND*	Vero E6	> 100 µM	ND*	ND*	ND*	Hit	Aldehyde warhead	[70]
3c	0.26 ± 0.05 µM	ND*	Biochemical assay	ND*	Vero E6	> 100 µM	ND*	ND*	ND*	Hit	Aldehyde warhead	[70]
2d	0.28 ± 0.08 µM	ND*	Biochemical assay	ND*	Vero E6	> 100 µM	ND*	ND*	ND*	Hit	Aldehyde warhead	
3d	0.30 ± 0.03 µM	ND*	Biochemical assay	ND*	Vero E6	> 100 µM	ND*	ND*	ND*	Hit	Aldehyde warhead	[70]
2e	0.26 ± 0.05 µM	ND*	Biochemical assay	ND*	Vero E6	> 100 µM	ND*	ND*	ND*	Hit	Aldehyde warhead	[70]
3e	0.28 ± 0.03 µM	ND*	Biochemical assay	ND*	Vero E6	> 100 µM	ND*	ND*	ND*	Hit	Aldehyde warhead	[70]
2f	0.14 ± 0.02 µM	ND*	Biochemical assay	ND*	Vero E6	> 100 µM	ND*	ND*	ND*	Hit	Aldehyde warhead	[70]
3f	0.10 ± 0.01 µM	ND*	Biochemical assay	ND*	Vero E6	> 100 µM	ND*	ND*	ND*	Hit	Aldehyde warhead	[70]
2g	1.90 ± 0.14 µM	ND*	Biochemical assay	ND*	Vero E6	> 100 µM	ND*	ND*	ND*	Hit	Aldehyde warhead	[70]
3g	1.71 ± 0.16 µM	ND*	Biochemical assay	ND*	Vero E6	> 100 µM	ND*	ND*	ND*	Hit	Aldehyde warhead	[70]
2h	0.39 ± 0.02 µM	ND*	Biochemical assay	ND*	Vero E6	> 100 µM	ND*	ND*	ND*	Hit	Aldehyde warhead	[70]
3h	0.37 ± 0.03 µM	ND*	Biochemical assay	ND*	Vero E6	> 100 µM	ND*	ND*	ND*	Hit	Aldehyde warhead	[70]
2i	0.27 ± 0.02 µM	ND*	Biochemical assay	ND*	Vero E6	21 ± 1 µM	ND*	ND*	ND*	Hit	Aldehyde warhead	[70]
3i	0.30 ± 0.02 µM	ND*	Biochemical assay	ND*	Vero E6	21 ± 3 µM	ND*	ND*	ND*	Hit	Aldehyde warhead	[70]
2j	0.31 ± 0.03 µM	ND*	Biochemical assay	ND*	Vero E6	20 ± 3 µM	ND*	ND*	ND*	Hit	Aldehyde warhead	[70]
3j	0.31 ± 0.06 µM	ND*	Biochemical assay	ND*	Vero E6	23 ± 1 µM	ND*	ND*	ND*	Hit	Aldehyde warhead	[70]
2k	0.18 ± 0.04 µM	ND*	Biochemical assay	ND*	Vero E6	> 100 µM	ND*	ND*	ND*	Hit	Aldehyde warhead	[70]

3k	0.15 ± 0.04 µM	ND*	Biochemical assay	ND*	Vero E6	> 100 µM	ND*	ND*	ND*	Hit	Aldehyde warhead	[70]
2l	0.29 ± 0.04 µM	ND*	Biochemical assay	ND*	Vero E6	> 100 µM	ND*	ND*	ND*	Hit	Aldehyde warhead	[70]
3l	0.27 ± 0.04 µM	ND*	Biochemical assay	ND*	Vero E6	> 100 µM	ND*	ND*	ND*	Hit	Aldehyde warhead	[70]
2m	0.27 ± 0.02 µM	ND*	Biochemical assay	ND*	Vero E6	> 100 µM	ND*	ND*	ND*	Hit	Aldehyde warhead	[70]
3m	0.25 ± 0.04 µM	ND*	Biochemical assay	ND*	Vero E6	> 100 µM	ND*	ND*	ND*	Hit	Aldehyde warhead	[70]
2n	0.82 ± 0.19 µM	ND*	Biochemical assay	ND*	Vero E6	> 100 µM	ND*	ND*	ND*	Hit	Aldehyde warhead	[70]
3n	1.03 ± 0.24 µM	ND*	Biochemical assay	ND*	Vero E6	> 100 µM	ND*	ND*	ND*	Hit	Aldehyde warhead	[70]
2o	0.74 ± 0.26 µM	ND*	Biochemical assay	ND*	Vero E6	> 100 µM	ND*	ND*	ND*	Hit	Aldehyde warhead	[70]
3o	0.78 ± 0.18 µM	ND*	Biochemical assay	ND*	Vero E6	> 100 µM	ND*	ND*	ND*	Hit	Aldehyde warhead	[70]
MG132	3.91 ± 1.12 µM	ND*	Hydrolytic activity	ND*	Vero E6	ND*	ND*	ND*	ND*	Hit	Proteasome inhibitor	[71]
Z-VAD(OMe)-FMK	0.59 ± 0.44 µM	ND*	Hydrolytic activity	1.88 ± 0.52 µM	Vero E6	>300 µM	ND*	ND*	ND*	Experimental	Caspase inhibitors	[71]
Z-DEVD-FMK	2.8 ± 0.32 µM	ND*	Hydrolytic activity	0.87 ± 0.10 µM	Vero E6	>300 µM	ND*	ND*	ND*	Hit	Caspase inhibitors	[71]
Z-IETD-FMK	1.61 ± 0.05 µM	ND*	Hydrolytic activity	0.64 ± 0.08 µM	Vero E6	>300 µM	ND*	ND*	ND*	Hit	Caspase inhibitors	[71]
1	100–250 µM	ND*	FRET assay	ND*	ND*	ND*	ND*	ND*	ND*	Hit	Cyanophenyl analogues	[72]
2	9.99 ± 2.50 µM	ND*	FRET assay	ND*	ND*	ND*	ND*	ND*	ND*	Hit	Cyanophenyl analogues	[72]
3	6.38 ± 1.21 µM	ND*	FRET assay	ND*	ND*	ND*	ND*	ND*	ND*	Hit	Cyanophenyl analogues	[72]
4	4.02 ± 1.36 µM	ND*	FRET assay	ND*	ND*	ND*	ND*	ND*	ND*	Hit	Cyanophenyl analogues	[72]
5	0.14 ± 0.02 µM	ND*	FRET assay	2.5 ± 0.7 µM	MTT assay	22 ± 7.2 µM	ND*	ND*	ND*	Lead	Cyanophenyl analogues	[72]

6	0.47 ± 0.02 µM	ND*	FRET assay	ND*	ND*	ND*	ND*	ND*	ND*	Hit	Cyanophenyl analogues	[72]
7	0.28 ± 0.05 µM	ND*	FRET assay	ND*	ND*	ND*	ND*	ND*	ND*	Hit	Cyanophenyl analogues	[72]
8	0.51 ± 0.02 µM	ND*	FRET assay	ND*	ND*	ND*	ND*	ND*	ND*	Hit	Cyanophenyl analogues	[72]
9	1 µM	ND*	FRET assay	ND*	ND*	ND*	ND*	ND*	ND*	Hit	Cyanophenyl analogues	[72]
10	1.20 ± 0.03 µM	ND*	FRET assay	ND*	ND*	ND*	ND*	ND*	ND*	Hit	Cyanophenyl analogues	[72]
11	0.120 ± 0.016 µM	ND*	FRET assay	ND*	ND*	ND*	ND*	ND*	ND*	Hit	Cyanophenyl analogues	[72]
12	0.25 ± 0.09 µM	ND*	FRET assay	ND*	ND*	ND*	ND*	ND*	ND*	Hit	Cyanophenyl analogues	[72]
13	0.19 ± 0.03 µM	ND*	FRET assay	ND*	ND*	ND*	ND*	ND*	ND*	Hit	Cyanophenyl analogues	[72]
14	0.128 ± 0.015 µM	ND*	FRET assay	ND*	ND*	12.3 ± 7.0 µM	ND*	ND*	ND*	Hit	Cyanophenyl analogues	[72]
15	0.110 ± 0.013 µM	ND*	FRET assay	ND*	ND*	ND*	ND*	ND*	ND*	Hit	Cyanophenyl analogues	[72]
16	0.100 ± 0.007 µM	ND*	FRET assay	ND*	ND*	ND*	ND*	ND*	ND*	Hit	Cyanophenyl analogues	[72]
17	0.110 ± 0.035 µM	ND*	FRET assay	ND*	ND*	ND*	ND*	ND*	ND*	Hit	Cyanophenyl analogues	[72]
18	0.024 ± 0.007 µM	ND*	FRET assay	ND*	ND*	ND*	ND*	ND*	ND*	Hit	Cyanophenyl analogues	[72]
19	0.037 ± 0.007 µM	ND*	FRET assay	ND*	ND*	ND*	ND*	ND*	ND*	Hit	Cyanophenyl analogues	[72]
20	0.036 ± 0.003 µM	ND*	FRET assay	ND*	ND*	ND*	ND*	ND*	ND*	Hit	Cyanophenyl analogues	[72]
21	0.018 ± 0.002 µM	ND*	FRET assay	ND*	ND*	1.7 ± 0.9 µM		ND*	ND*	Hit	Cyanophenyl analogues	[72]
22	0.036 ± 0.004 µM	ND*	FRET assay	ND*	ND*	ND*	ND*	ND*	ND*	Hit	Cyanophenyl analogues	[72]
23	0.020 ± 0.005 µM	ND*	FRET assay	ND*	ND*	1.15 ± 0.5 µM	ND*	ND*	ND*	Hit	Cyanophenyl analogues	[72]
24	0.037 ± 0	ND*	FRET assay	ND*	ND*	ND*	ND*	ND*	ND*	Hit	Cyanophenyl	[72]

	.004 µM										analogues	
25	0.025 ± 0.003 µM	ND*	FRET assay	ND*	ND*	ND*	ND*	ND*	ND*	Hit	Cyano phenyl analogues	[72]
26	0.170 ± 0.022 µM	ND*	FRET assay	2.0 ± 0.7 µM	MTT assay	>100 µM	ND*	ND*	ND*	Experimental	Cyano phenyl analogues	[72]
27	0.120 ± 0.006 µM	ND*	FRET assay	1.1 ±0.5 µM	MTT assay	22 ± 8 µM	ND*	ND*	ND*	Hit	Cyano phenyl analogues	[72]
Remdesivir	ND*	ND*	ND*	1.1 ± 0.2 µM	MTT assay	72 ± 28 µM	ND*	ND*	ND*	Control	FDA approved	[72]
Perampanel	100–250 µM	ND*	FRET assay	ND*	ND*	ND*	ND*	ND*	ND*	Lead		[73]
2	9.99 ± 2.50 µM	ND*	FRET assay	ND*	ND*	ND*	ND*	ND*	ND*	Hit	Perampanel analogs	[73]
3	6.38 ± 1.21 µM	ND*	FRET assay	ND*	ND*	ND*	ND*	ND*	ND*	Hit	Perampanel analogs	[73]
4	4.02 ± 1.36 µM	ND*	FRET assay	ND*	ND*	ND*	ND*	ND*	ND*	Hit	Perampanel analogs	[73]
5	0.14 ± 0.02 µM	ND*	FRET assay	ND*	ND*	ND*	ND*	ND*	ND*	Hit	Perampanel analogs	[73]
6	0.47 ± 0.02 µM	ND*	FRET assay	ND*	ND*	ND*	ND*	ND*	ND*	Hit	Perampanel analogs	[73]
11	0.120 ± 0.016 µM	ND*	FRET assay	ND*	ND*	ND*	ND*	ND*	ND*	Hit	Perampanel analogs	[73]
14	0.128 ± 0.015 µM	ND*	FRET assay	ND*	ND*	ND*	ND*	ND*	ND*	Hit	Perampanel analogs	[73]
15	0.110 ± 0.013 µM	ND*	FRET assay	ND*	ND*	ND*	ND*	ND*	ND*	Hit	Perampanel analogs	[73]
16	0.100 ± 0.035 µM	ND*	FRET assay	ND*	ND*	ND*	ND*	ND*	ND*	Hit	Perampanel analogs	[73]
19	0.037 ± 0.007 µM	ND*	FRET assay	ND*	ND*	ND*	ND*	ND*	ND*	Hit	Perampanel analogs	[73]
21	0.018 ± 0.002 µM	ND*	FRET assay	ND*	ND*	ND*	ND*	ND*	ND*	Hit	Perampanel analogs	[73]
23	0.020 ± 0.00	ND*	FRET assay	ND*	ND*	ND*	ND*	ND*	ND*	Hit	Perampanel analogs	[73]

	5 µM											
25	0.025 ± 0.003 µM	ND*	FRET assay	ND*	ND*	ND*	ND*	ND*	ND*	Hit	Perampanel analogs	[73]
26	0.170 ± 0.022 µM	ND*	FRET assay	ND*	ND*	ND*	ND*	ND*	ND*	Hit	Perampanel analogs	[73]
29	0.25 – 0.50 µM	ND*	FRET assay	ND*	ND*	ND*	ND*	ND*	ND*	Hit	Perampanel analogs	[73]
31	0.037 ± 0.004 µM	ND*	FRET assay	ND*	ND*	ND*	ND*	ND*	ND*	Hit	Perampanel analogs	[73]
50	0.25 – 0.50 µM	ND*	FRET assay	ND*	ND*	ND*	ND*	ND*	ND*	Hit	Perampanel analogs	[73]
11a	53 ± 5 nM	ND*	FRET assay	0.53 ± 0.01 µM	Vero E6	>100 µM	ND*	ND*	ND*	Experimental	ND*	[74]
11b	40 ± 2 nM	ND*	FRET assay	0.72 ± 0.09 µM	Vero E6	>100 µM	ND*	ND*	ND*	Experimental	ND*	[74]
MI-01	453.0 ± 3.2 nM	ND*	FRET assay	ND*	ND*	ND*	ND*	ND*	ND*	Hit	Boceprevir or telaprevir based	[75]
MI-02	52.1 ± 1.4 nM	ND*	FRET assay	ND*	ND*	ND*	ND*	ND*	ND*	Hit	Boceprevir or telaprevir based	[75]
MI-03	16.5 ± 1.4 nM	ND*	FRET assay	2.23 ± 0.62 µM	Vero E6	>500 µM	ND*	ND*	ND*	Hit	Boceprevir or telaprevir based	[75]
MI-04	18.5 ± 0.5 nM	ND*	FRET assay	1.64 ± 0.88 µM	Vero E6	>500 µM	ND*	ND*	ND*	Hit	Boceprevir or telaprevir based	[75]
MI-05	13.2 ± 0.4 nM	ND*	FRET assay	5.57 ± 1.00 µM	Vero E6	>500 µM	ND*	ND*	ND*	Hit	Boceprevir or telaprevir based	[75]
MI-06	14.5 ± 0.4 nM	ND*	FRET assay	1.73 ± 1.01 µM	Vero E6	>500 µM	ND*	ND*	ND*	Hit	Boceprevir or telaprevir based	[75]
MI-07	43.3 ± 0.8 nM	ND*	FRET assay	16.95 ± 0.90 µM	Vero E6	>500 µM	ND*	ND*	ND*	Hit	Boceprevir or telaprevir based	[75]
MI-08	37.2 ± 0.7 nM	ND*	FRET assay	4.68 ± 0.83 µM	Vero E6	>500 µM	ND*	ND*	ND*	Hit	Boceprevir or telaprevir based	[75]

MI-09	15.2 ± 0.4 nM	ND*	FRET assay	0.86 ± 0.07 µM	Vero E6	>500 µM	22.67 ± 2.97 mL/min/kg	ND*	ND*	Hit	Boceprevir or telaprevir based	[75]
MI-10	50.8 ± 0.5 nM	ND*	FRET assay	ND*	ND*	ND*	ND*	ND*	ND*	Hit	Boceprevir or telaprevir based	[75]
MI-11	13.3 ± 0.2 nM	ND*	FRET assay	1.18 ± 0.10 µM	Vero E6	>500 µM	ND*	ND*	ND*	Hit	Boceprevir or telaprevir based	[75]
MI-12	19.0 ± 0.6 nM	ND*	FRET assay	0.53 ± 0.07 µM	Vero E6	>500 µM	31.17 ± 4.65	ND*	ND*	Experimental	Boceprevir or telaprevir based	[75]
MI-13	12.4 ± 0.2 nM	ND*	FRET assay	2.08 ± 0.81 µM	Vero E6	>500 µM	ND*	ND*	ND*	Hit	Boceprevir or telaprevir based	[75]
MI-14	13.0 ± 0.3 nM	ND*	FRET assay	0.66 ± 0.06 µM	Vero E6	>500 µM	18.05 ± 2.57	ND*	ND*	Hit	Boceprevir or telaprevir based	[75]
MI-15	748.5 ± 2.3 nM	ND*	FRET assay	ND*	ND*	ND*	ND*	ND*	ND*	Hit	Boceprevir or telaprevir based	[75]
MI-16	153.1 ± 1.0 nM	ND*	FRET assay	ND*	ND*	ND*	ND*	ND*	ND*	Hit	Boceprevir or telaprevir based	[75]
11b	27.4 ± 0.5 nM	ND*	FRET assay	0.89 ± 0.04 µM	Vero E6	>500 µM	ND*	ND*	ND*	Hit	Boceprevir or telaprevir based	[75]
MI-17	298.8 ± 2.3 nM	ND*	FRET assay	ND*	ND*	ND*	ND*	ND*	ND*	Hit	Boceprevir or telaprevir based	[75]
MI-18	525.9 ± 8.3 nM	ND*	FRET assay	ND*	ND*	ND*	ND*	ND*	ND*	Hit	Boceprevir or telaprevir based	[75]
MI-19	195.6 ± 0.8 nM	ND*	FRET assay	ND*	ND*	ND*	ND*	ND*	ND*	Hit	Boceprevir or telaprevir based	[75]
MI-20	375.0 ± 1.0 nM	ND*	FRET assay	ND*	ND*	ND*	ND*	ND*	ND*	Hit	Boceprevir or telaprevir based	[75]
MI-21	7.6 ± 0.2 nM	ND*	FRET assay	2.97 ± 0.81 µM	Vero E6	>500 µM	ND*	ND*	ND*	Hit	Boceprevir or telaprevir based	[75]
MI-22	17.4 ± 0.4 nM	ND*	FRET assay	30.49 ± 1.24 µM	Vero E6	>500 µM	ND*	ND*	ND*	Hit	Boceprevir or telapre	[75]

											vir based	
MI-23	7.6 ± 0.1 nM	ND*	FRET assay	5.63 ± 1.09 µM	Vero E6	>500 µM	ND*	ND*	ND*	Hit	Boceprevir or telaprevir based	[75]
MI-24	378.2 ± 2.3 nM	ND*	FRET assay	24.72 ± 17.17 µM	Vero E6	>500 µM	ND*	ND*	ND*	Hit	Boceprevir or telaprevir based	[75]
MI-25	36.2 ± 0.7 nM	ND*	FRET assay	ND*	ND*	ND*	ND*	ND*	ND*	Hit	Boceprevir or telaprevir based	[75]
MI-26	69.1 ± 1.6 nM	ND*	FRET assay	ND*	ND*	ND*	ND*	ND*	ND*	Hit	Boceprevir or telaprevir based	[75]
MI-27	93.5 ± 1.2 nM	ND*	FRET assay	ND*	ND*	ND*	ND*	ND*	ND*	Hit	Boceprevir or telaprevir based	[75]
MI-28	9.2 ± 0.2 nM	ND*	FRET assay	0.67 ± 0.06 µM	Vero E6	>500 µM	14.25 ± 0.54	ND*	ND*	Hit	Boceprevir or telaprevir based	[75]
MI-29	34.7 ± 0.4 nM	ND*	FRET assay	1.65 ± 0.23 µM	Vero E6	>500 µM	ND*	ND*	ND*	Hit	Boceprevir or telaprevir based	[75]
MI-30	17.2 ± 0.6 nM	ND*	FRET assay	0.54 ± 0.13 µM	Vero E6	>500 µM	17.10 ± 1.12	ND*	ND*	Experimental	Boceprevir or telaprevir based	[75]
MI-31	30.0 ± 0.4 nM	ND*	FRET assay	0.83 ± 0.28 µM	Vero E6	>500 µM	8.94 ± 1.63	ND*	ND*	Hit	Boceprevir or telaprevir based	[75]
MI-32	19.7 ± 0.7 nM	ND*	FRET assay	1.50 ± 0.28 µM	Vero E6	>500 µM	ND*	ND*	ND*	Hit	Boceprevir or telaprevir based	[75]
GC376	37.4 ± 1.9 nM	ND*	FRET assay	1.46 ± 0.14 µM	Vero E6	>100 µM	ND*	ND*	ND*	Control		[75]
1(ML300)	4.99 ± 0.62 µm	ND*	FRET assay	19.90 ± 6.32 µM	Vero E6	ND*	ND*	ND*	ND*	Lead		[76]
7(17a)	31.8 ± 4.65 µM	ND*	FRET assay	ND*	ND*	ND*	ND*	ND*	ND*	Hit	ML300 derivatives	[76]
8(17b)	0.95 ± 0.06 µM	ND*	FRET assay	12.07 ± 0.31 µM	Vero E6	ND*	ND*	ND*	ND*	Hit	ML300 derivatives	[76]
9	18.87 ± 1.34 µM	ND*	FRET assay	ND*	ND*	ND*	ND*	ND*	ND*	Hit	ML300 derivatives	[76]

10	0.44 ± 0.06 µM	ND*	FRET assay	ND*	ND*	ND*	ND*	ND*	ND*	Hit	ML300 derivatives	[76]
11	15.94 ± 1.01 µM	ND*	FRET assay	ND*	ND*	ND*	ND*	ND*	ND*	Hit	ML300 derivatives	[76]
12	1.61 ± 0.44 µM	ND*	FRET assay	ND*	ND*	ND*	ND*	ND*	ND*	Hit	ML300 derivatives	[76]
13	8.77 ± 0.20 µM	ND*	FRET assay	ND*	ND*	ND*	ND*	ND*	ND*	Hit	ML300 derivatives	[76]
14	2.09 ± 0.27 µM	ND*	FRET assay	ND*	ND*	ND*	ND*	ND*	ND*	Hit	ML300 derivatives	[76]
15	1.40 ± 0.21 µM	ND*	FRET assay	ND*	ND*	ND*	ND*	ND*	ND*	Hit	ML300 derivatives	[76]
16	5.07 ± 0.60 µM	ND*	FRET assay	ND*	ND*	ND*	ND*	ND*	ND*	Hit	ML300 derivatives	[76]
17	0.92 ± 0.21 µM	ND*	FRET assay	ND*	ND*	ND*	ND*	ND*	ND*	Hit	ML300 derivatives	[76]
18	20.39 ± 0.42 µM	ND*	FRET assay	ND*	ND*	ND*	ND*	ND*	ND*	Hit	ML300 derivatives	[76]
19	0.106 ± 0.025 µM	ND*	FRET assay	5.76 ± 1.96 µM	Vero E6	ND*	ND*	ND*	ND*	Hit	ML300 derivatives	[76]
20	9.55 ± 0.99 µM	ND*	FRET assay	ND*	ND*	ND*	ND*	ND*	ND*	Hit	ML300 derivatives	[76]
21	0.063 ± 0.014 µM	ND*	FRET assay	1.74 ± 0.19 µM	Vero E6	ND*	ND*	ND*	ND*	Hit	ML300 derivatives	[76]
35	0.746 ± 0.098 µM	ND*	FRET assay	8.77 ± 1.14 µM	Vero E6	ND*	ND*	ND*	ND*	Hit	ML300 derivatives	[76]
36	0.270 ± 0.067 µM	ND*	FRET assay	1.98 ± 0.24 µM	Vero E6	ND*	ND*	ND*	ND*	Hit	ML300 derivatives	[76]
37	0.328 ± 0.027 µM	ND*	FRET assay	2.93 ± 0.28 µM	Vero E6	ND*	ND*	ND*	ND*	Hit	ML300 derivatives	[76]
38	0.197 ±0.050 µM	ND*	FRET assay	2.30 ± 0.33 µM	Vero E6	ND*	ND*	ND*	ND*	Hit	ML300 derivatives	[76]
39	0.333 ± 0.09	ND*	FRET assay	3.81 ± 0.18 µM	Vero E6	ND*	ND*	ND*	ND*	Hit	ML300 derivatives	[76]

	3 µM											
40	0.171 ±0.029 µM	ND*	FRET assay	1.91 ± 0.30 µM	Vero E6	ND*	ND*	ND*	ND*	Hit	ML300 derivatives	[76]
41	0.068 ± 0.023 µM	ND*	FRET assay	0.497 ± 0.009 µM	Vero E6	ND*	ND*	ND*	ND*	Experimental	ML300 derivatives	[76]
22	4.62 ± 0.44 µM	ND*	FRET assay	ND*	ND*	ND*	ND*	ND*	ND*	Hit	ML300 derivatives	[76]
23	2.51 ± 0.44 µM	ND*	FRET assay	ND*	ND*	ND*	ND*	ND*	ND*	Hit	ML300 derivatives	[76]
24	0.83 ± 0.03 µM	ND*	FRET assay	ND*	ND*	ND*	ND*	ND*	ND*	Hit	ML300 derivatives	[76]
25	1.16 ± 0.19 µM	ND*	FRET assay	ND*	ND*	ND*	ND*	ND*	ND*	Hit	ML300 derivatives	[76]
26	23.0 ± 1.38 µM	ND*	FRET assay	ND*	ND*	ND*	ND*	ND*	ND*	Hit	ML300 derivatives	[76]
27	1.59 ± 0.44 µM	ND*	FRET assay	ND*	ND*	ND*	ND*	ND*	ND*	Hit	ML300 derivatives	[76]
28	0.39 ± 0.11 µM	ND*	FRET assay	7.61 ± 1.43 µM	Vero E6	ND*	ND*	ND*	ND*	Hit	ML300 derivatives	[76]
29	8.53 ± 1.82 µM	ND*	FRET assay	ND*	ND*	ND*	ND*	ND*	ND*	Hit	ML300 derivatives	[76]
30	>200 µM	ND*	FRET assay	ND*	ND*	ND*	ND*	ND*	ND*	Hit	ML300 derivatives	[76]
31	0.119 ± 0.05 µM	ND*	FRET assay	ND*	ND*	ND*	ND*	ND*	ND*	Hit	ML300 derivatives	[76]
32	0.176 ± 0.025 µM	ND*	FRET assay	ND*	ND*	ND*	ND*	ND*	ND*	Hit	ML300 derivatives	[76]
33	0.312 ± 0.073 µM	ND*	FRET assay	ND*	ND*	ND*	ND*	ND*	ND*	Hit	ML300 derivatives	[76]
34	0.540 ± 0.070 µM	ND*	FRET assay	ND*	ND*	ND*	ND*	ND*	ND*	Hit	ML300 derivatives	[76]
42	1.27 µM	ND*	FRET assay	ND*	ND*	ND*	ND*	ND*	ND*	Hit	ML300 derivatives	[76]
43	3.44 µM	ND*	FRET assay	ND*	ND*	ND*	ND*	ND*	ND*	Hit	ML300 derivatives	[76]

Remdesivir	ND*	ND*	ND*	0.34 ± 0.01 µM	Vero E6	ND*	ND*	ND*	ND*	Control	FDA approved	[76]
GC376	0.11 ± 0.06 µM	ND*	Enzymatic assay	ND*	ND*	ND*	ND*	ND*	ND*	Control		[77]
X77	4.1 ± 1.2 µM	ND*	Enzymatic assay	ND*	ND*	ND*	ND*	ND*	ND*	Control		[77]
ML188	1.4 ± 0.4 µM	ND*	Enzymatic assay	ND*	ND*	ND*	ND*	ND*	ND*	Control		[77]
6a	11.1 ± 1.5 µM	ND*	Enzymatic assay	ND*	ND*	ND*	ND*	ND*	ND*	Hit	X77 derivatives	[77]
13a	5.3 ± 0.8 µM	ND*	Enzymatic assay	ND*	ND*	ND*	ND*	ND*	ND*	Hit	X77 derivatives	[77]
14a	0.42 ± 0.11 µM	ND*	Enzymatic assay	ND*	ND*	ND*	ND*	ND*	ND*	Hit	X77 derivatives	[77]
16a	0.41 ± 0.13 µM	ND*	Enzymatic assay	ND*	ND*	ND*	ND*	ND*	ND*	Hit	X77 derivatives	[77]
18a	5.2 ± 1.2 µM	ND*	Enzymatic assay	ND*	ND*	ND*	ND*	ND*	ND*	Hit	X77 derivatives	[77]
20a	7.0 ± 0.2 µM	ND*	Enzymatic assay	ND*	ND*	ND*	ND*	ND*	ND*	Hit	X77 derivatives	[77]
22a	12.4 ± 5.2 µM	ND*	Enzymatic assay	ND*	ND*	ND*	ND*	ND*	ND*	Hit	X77 derivatives	[77]
23a	0.85 ± 0.42 µM	ND*	Enzymatic assay	ND*	ND*	ND*	ND*	ND*	ND*	Hit	X77 derivatives	[77]
13b	15.0 ± 9.3 µM	ND*	Enzymatic assay	ND*	ND*	ND*	ND*	ND*	ND*	Hit	X77 derivatives	[77]
13c	9.7 ± 3.8 µM	ND*	Enzymatic assay	ND*	ND*	ND*	ND*	ND*	ND*	Hit	X77 derivatives	[77]
13d	>30 µM	ND*	Enzymatic assay	ND*	ND*	ND*	ND*	ND*	ND*	Hit	X77 derivatives	[77]
16b	0.38 ± 0.09 µM	ND*	Enzymatic assay	ND*	ND*	ND*	ND*	ND*	ND*	Hit	X77 derivatives	[77]
16c	0.92 ± 0.24 µM	ND*	Enzymatic assay	ND*	ND*	ND*	ND*	ND*	ND*	Hit	X77 derivatives	[77]
14b	0.28 ± 0.10 µM	ND*	Enzymatic assay	ND*	ND*	ND*	ND*	ND*	ND*	Hit	X77 derivatives	[77]
14c	0.17 ± 0.07 µM	ND*	Enzymatic assay	ND*	ND*	ND*	ND*	ND*	ND*	Experimental	X77 derivatives	[77]
14d	0.24 ± 0.15 µM	ND*	Enzymatic assay	ND*	ND*	ND*	ND*	ND*	ND*	Hit	X77 derivatives	[77]
14e	0.52 ± 0.16 µM	ND*	Enzymatic assay	ND*	ND*	ND*	ND*	ND*	ND*	Hit	X77 derivatives	[77]

14f	0.22 ± 0.08 µM	ND*	Enzymatic assay	ND*	ND*	ND*	ND*	ND*	ND*	Hit	X77 derivatives	[77]
14g (R,S)	0.32 ± 0.10 µM	ND*	Enzymatic assay	ND*	ND*	ND*	ND*	ND*	ND*	Hit	X77 derivatives	[77]
14h (S,S)	6.0 ± 2.7 µM	ND*	Enzymatic assay	ND*	ND*	ND*	ND*	ND*	ND*	Hit	X77 derivatives	[77]
16e	0.84 ± 0.30 µM	ND*	Enzymatic assay	ND*	ND*	ND*	ND*	ND*	ND*	Hit	X77 derivatives	[77]
16f	0.98 ± 0.35 µM	ND*	Enzymatic assay	ND*	ND*	ND*	ND*	ND*	ND*	Hit	X77 derivatives	[77]
13e	5.0 ± 2.3 µM	ND*	Enzymatic assay	ND*	ND*	ND*	ND*	ND*	ND*	Hit	X77 derivatives	[77]
13p	45.1 ± 18.3 µM	ND*	Enzymatic assay	ND*	ND*	ND*	ND*	ND*	ND*	Hit	X77 derivatives	[77]
Disulfiram	6.1 ± 0.6a µM	ND*	FRET assay	ND*	ND*	ND*	ND*	ND*	ND*	Control		[78]
Ebselen	1.7 ± 0.4a µM	ND*	FRET assay	ND*	ND*	ND*	ND*	ND*	ND*	Control		[78]
Ibuprofen	>500 µM	ND*	FRET assay	ND*	ND*	ND*	ND*	ND*	ND*	Control		[78]
1	2.6 ± 0.39 µM	ND*	FRET assay	ND*	ND*	ND*	ND*	ND*	ND*	Hit	3,7-diazabicyclo[3.3.1]nonane (bispidine) derivatives	[78]
2a	5.9 ± 2.2 µM	ND*	FRET assay	ND*	ND*	ND*	ND*	ND*	ND*	Hit	3,7-diazabicyclo[3.3.1]nonane (bispidine) derivatives	[78]
2b	18.5 ± 3.1 µM	ND*	FRET assay	ND*	ND*	ND*	ND*	ND*	ND*	Hit	3,7-diazabicyclo[3.3.1]nonane (bispidine) derivatives	[78]
2c	5.5 ± 0.7 µM	ND*	FRET assay	ND*	ND*	ND*	ND*	ND*	ND*	Hit	3,7-diazabicyclo[3.3.1]nonane (bispidine) derivatives	[78]
2d	3.3 ± 0.5 µM	ND*	FRET assay	ND*	ND*	ND*	ND*	ND*	ND*	Hit	3,7-diazabicyclo[3.3.1]no	[78]

											nane (bispidine) derivatives	
2e	4.8 ± 0.6 µM	ND*	FRET assay	ND*	ND*	ND*	ND*	ND*	ND*	Hit	3,7-diazabicyclo[3.3.1]nonane (bispidine) derivatives	[78]
2f	1.45 ± 0.2 µM	ND*	FRET assay	ND*	ND*	ND*	ND*	ND*	ND*	Hit	3,7-diazabicyclo[3.3.1]nonane (bispidine) derivatives	[78]
2g	4.99 ± 0.8 µM	ND*	FRET assay	ND*	ND*	ND*	ND*	ND*	ND*	Hit	3,7-diazabicyclo[3.3.1]nonane (bispidine) derivatives	[78]
2h	1.4 ± 0.2 µM	ND*	FRET assay	ND*	ND*	ND*	ND*	ND*	ND*	Hit	3,7-diazabicyclo[3.3.1]nonane (bispidine) derivatives	[78]
2i	13.0 ± 2.1 µM	ND*	FRET assay	ND*	ND*	ND*	ND*	ND*	ND*	Hit	3,7-diazabicyclo[3.3.1]nonane (bispidine) derivatives	[78]
2j	0.75 ± 0.2 µM	ND*	FRET assay	ND*	ND*	ND*	ND*	ND*	ND*	Experimental	3,7-diazabicyclo[3.3.1]nonane (bispidine) derivatives	[78]
3a	17.9 ± 3.1 µM	ND*	FRET assay	ND*	ND*	ND*	ND*	ND*	ND*	Hit	3,7-diazabicyclo[3.3.1]nonane (bispidine) derivatives	[78]
3e	46.6 ± 3.1 µM	ND*	FRET assay	ND*	ND*	ND*	ND*	ND*	ND*	Hit	3,7-diazabicyclo[3.3.1]nonane (bispidine)	[78]

											derivatives	
3k	99.6 ± 10 µM	ND*	FRET assay	ND*	ND*	ND*	ND*	ND*	ND*	Hit	3,7-diazabicyclo[3.3.1]nonane (bispidine) derivatives	[78]
4c	8.8 ± 2 µM	ND*	FRET assay	ND*	ND*	ND*	ND*	ND*	ND*	Hit	3,7-diazabicyclo[3.3.1]nonane (bispidine) derivatives	[78]
4d	0.9 ± 0.2 µM	ND*	FRET assay	ND*	ND*	ND*	ND*	ND*	ND*	Hit	3,7-diazabicyclo[3.3.1]nonane (bispidine) derivatives	[78]
4l	5.5 ± 1.2 µM	ND*	FRET assay	ND*	ND*	ND*	ND*	ND*	ND*	Hit	3,7-diazabicyclo[3.3.1]nonane (bispidine) derivatives	[78]
4m	7.1 ± 1.4 µM	ND*	FRET assay	ND*	ND*	ND*	ND*	ND*	ND*	Hit	3,7-diazabicyclo[3.3.1]nonane (bispidine) derivatives	[78]
4n	33.7 ± 5.2 µM	ND*	FRET assay	ND*	ND*	ND*	ND*	ND*	ND*	Hit	3,7-diazabicyclo[3.3.1]nonane (bispidine) derivatives	[78]
4o	5.2 ± 1.1 µM	ND*	FRET assay	ND*	ND*	ND*	ND*	ND*	ND*	Hit	3,7-diazabicyclo[3.3.1]nonane (bispidine) derivatives	[78]
4p	2.02 ± 0.41 µM	ND*	FRET assay	ND*	ND*	ND*	ND*	ND*	ND*	Hit	3,7-diazabicyclo[3.3.1]nonane (bispidine) derivatives	[78]

4q	0.83 ± 0.17 µM	ND*	FRET assay	ND*	ND*	ND*	ND*	ND*	ND*	Hit	3,7-diazabicyclo[3.3.1]nonane (bispidine) derivatives	[78]
4r	2.2 ± 0.5 µM	ND*	FRET assay	ND*	ND*	ND*	ND*	ND*	ND*	Hit	3,7-diazabicyclo[3.3.1]nonane (bispidine) derivatives	[78]
4s	16.3 ± 3.1 µM	ND*	FRET assay	ND*	ND*	ND*	ND*	ND*	ND*	Hit	3,7-diazabicyclo[3.3.1]nonane (bispidine) derivatives	[78]
4t	13.6 ± 1.4 µM	ND*	FRET assay	ND*	ND*	ND*	ND*	ND*	ND*	Hit	3,7-diazabicyclo[3.3.1]nonane (bispidine) derivatives	[78]
Leupeptin	92 µM	ND*	Enzymatic assay	ND*	ND*	ND*	ND*	ND*	ND*	Hit	Repurposed	[79]
Telaprevir	18 µM	ND*	Enzymatic assay	ND*	ND*	ND*	ND*	ND*	ND*	Hit	Repurposed	[79]
Narlaprevir	5.1 µM	ND*	Enzymatic assay	ND*	ND*	ND*	ND*	ND*	ND*	Hit	Repurposed	[79]
Boceprevir	3.1 µM	ND*	Enzymatic assay	ND*	ND*	ND*	ND*	ND*	ND*	Hit	Repurposed	[79]
AT7519	ND*	ND*	ND*	25.16 µM	Vero E6	>100 µM	ND*	ND*	ND*	ND*	Repurposed	[80]
Calpeptin	ND*	ND*	ND*	0.072 µM	Vero E6	>100 µM	ND*	ND*	ND*	ND*	Repurposed	[80]
HEAT	ND*	ND*	ND*	24.05 µM	Vero E6	55.42 µM	ND*	ND*	ND*	ND*	Repurposed	[80]
Ifenprodil	ND*	ND*	ND*	46.86 µM	Vero E6	>100 µM	ND*	ND*	ND*	ND*	Repurposed	[80]
Isofloxythepin	ND*	ND*	ND*	4.8 µM	Vero E6	17 µM	ND*	ND*	ND*	ND*	Repurposed	[80]
MUT056399	ND*	ND*	ND*	38.24 µM	Vero E6	>100 µM	ND*	ND*	ND*	ND*	Repurposed	[80]
Pelitinib	ND*	ND*	ND*	1.25 µM	Vero E6	13.96 µM	ND*	ND*	ND*	ND*	Repurposed	[80]
Quipazine meleate	ND*	ND*	ND*	31.64 µM	Vero E6	>100 µM	ND*	ND*	ND*	ND*	Repurposed	[80]
RS-102895	ND*	ND*	ND*	19.8 µM	Vero E6	54.98 µM	ND*	ND*	ND*	ND*	Repurposed	[80]
Tolperisone	ND*	ND*	ND*	19.17 µM	Vero E6	>100 µM	ND*	ND*	ND*	ND*	Repurposed	[80]

Triglycidyl isocyanurate	ND*	ND*	ND*	30.02 μM	Vero E6	>100 μM	ND*	ND*	ND*	ND*	Repurposed	[80]
GC376	13.8 μM	ND*	Enzymatic assay	74 ± 35 nM	Vero E6	ND*	ND*	ND*	ND*	Control		[80]
Entrectinib	10.6 μM	ND*	Enzymatic assay	198 ± 116 nM	Vero E6	ND*	ND*	ND*	ND*	Experimental	Repurposed	[80]
Simeprevir	2.46 ± 0.26 μM	High throuput screeniong	ND*	1.40 ± 0.09 μM	Vero E6	18.58 ± 0.70 μM	ND*	ND*	ND*	Hit	Repurposed	[80]
Cinacalcet	5.99 ± 1.32 μM	High throuput screeniong	ND*	2.93 ± 0.38 μM	Vero E6	21.23 ± 1.62 μM	ND*	ND*	ND*	Hit	Repurposed	[80]
Ethacridine	3.54 ± 0.66 μM	High throuput screeniong	ND*	0.08 ± 0.01 μM	Vero E6	>40 μM	ND*	ND*	ND*	Experimental	Repurposed	[80]
ABT-199 (veneotclax)	3.18 ± 0.55 μM	High throuput screeniong	ND*	1.18 ± 0.12 μM	Vero E6	20.56 ± 2.56 μM	ND*	ND*	ND*	Hit	Repurposed	[80]
Hydroxyprogesterone	2.47 ± 0.30 μM	High throuput screeniong	ND*	2.77 ± 0.49 μM	Vero E6	>40 μM	ND*	ND*	ND*	Hit	Repurposed	[80]
Butoconazole	5.07 ± 0.58 μM	High throuput screeniong	ND*	ND*	ND*	ND*	ND*	ND*	ND*	Hit	Repurposed	[80]
Remdesivir	ND*	ND*	ND*	0.52 ± 0.09 μM	Vero E6	>40 μM	ND*	ND*	ND*	Control	FDA approved	[80]
CDD-1777	NI	2400 ± 230 nM	Enzymatic assay	ND*	ND*	ND*	ND*	ND*	ND*	Hit	DNA-encoded chemistry technology	[81]
CDD-1713	5.19 μM	24 ± 3 nM	Enzymatic assay	ND*	ND*	ND*	ND*	ND*	ND*	Hit	DNA-encoded chemistry technology	[81]
CDD-1976	2.50 μM	42 ± 5 nM	Enzymatic assay	ND*	ND*	ND*	ND*	ND*	ND*	Hit	DNA-encoded chemistry technology	[81]
PUG	4.62 ±	ND*	FRET assay	7.20 ±	Vero E6	>100 μm	ND*	ND*	ND*	Experimental	Repurposed	[82]

	0.27 µM			1.08 µM								
CHLA	9.09 ± 0.87 µM	ND*	FRET assay	9.76 ± 0.42 µM	Vero E6	>100 µM	ND*	ND*	ND*	Hit	Repurposed	[82]
GC376	0.47 ± 0.03 µM	ND*	FRET assay	ND*	ND*	ND*	ND*	ND*	ND*	Control	Repurposed	[83]
Bismuth Gluconate	0.96 ± 0.02 µM	ND*	FRET assay	ND*	ND*	ND*	ND*	ND*	ND*	Hit	Repurposed	[83]
CBS	0.93 ± 0.04 µM	ND*	FRET assay	177.3 ± 32 µM	Vero E6	>200 µM	ND*	ND*	ND*	Experimental	Repurposed	[83]
GC376	160 ± 34 nM	ND*	Enzymatic assay	ND*	ND*	ND*	ND*	ND*	ND*	Control		[84]
Compound 4	151 ± 15 nM	ND*	Enzymatic assay	2.883 ± 0.227 µM	Vero E6	ND*	ND*	ND*	ND*	Experimental		[84]
MAC-5576	81 ± 12 nM	ND*	Enzymatic assay	ND*	ND*	ND*	ND*	ND*	ND*	Hit		[84]
Ebselen	0.67 ± 0.09 µM	ND*	Enzymatic assay	4.67 ± 0.80 µM	Vero E6	ND*	ND*	ND*	ND*	Hit	Repurposed	[40]
Disulfiram	9.35 ± 0.18 µM	ND*	Enzymatic assay	ND*	ND*	ND*	ND*	ND*	ND*	Hit	Repurposed	[40]
Tideglusib	1.55 ± 0.30 µM	ND*	Enzymatic assay	ND*	ND*	ND*	ND*	ND*	ND*	Hit	Repurposed	[40]
Carmofur	1.82 ± 0.06 µM	ND*	Enzymatic assay	ND*	ND*	ND*	ND*	ND*	ND*	Hit	Repurposed	[40]
Shikonin	15.75 ± 8.22 µM	ND*	Enzymatic assay	ND*	ND*	ND*	ND*	ND*	ND*	Hit	Repurposed	[40]
PX-12	21.39 ± 7.06 µM	ND*	Enzymatic assay	ND*	ND*	ND*	ND*	ND*	ND*	Hit	Repurposed	[40]
N3		ND*	ND	16.77 ± 1.7 µM	Vero E6	ND*	ND*	ND*	ND*	Experimental	Repurposed	[40]
6a	0.82 ± 0.1 µM	ND*	FRET enzyme assay	ND	Vero E6	>100 µM	ND*	ND*	ND*	Hit	Broad-spectrum inhibitors	[85]
7a	0.65 ± 0.06 µM	ND*	FRET enzyme assay	ND	Vero E6	>100 µM	ND*	ND*	ND*	Hit	Broad-spectrum inhibitors	[85]
6c	0.28 ± 0.05 µM	ND*	FRET enzyme assay	0.25 ± 0.15 µM	Vero E6	>100 µM	ND*	ND*	ND*	Hit	Broad-spectrum inhibitors	[85]

7c	0.23 ± 0.05 µM	ND*	FRET enzyme assay	ND	Vero E6	>100 µM	ND*	ND*	ND*	Hit	Broad-spectrum inhibitors	[85]
6e	0.17 ± 0.06 µM	ND*	FRET enzyme assay	0.15 ± 0.14 µM	Vero E6	63.3 ± 2.3 µM	ND*	ND*	ND*	Experimental	Broad-spectrum inhibitors	[85]
7e	0.20 ± 0.06 µM	ND*	FRET enzyme assay	ND	Vero E6	59.1 ± 3.5 µM	ND*	ND*	ND*	Hit	Broad-spectrum inhibitors	[85]
6h	0.43 ± 0.1 µM	ND*	FRET enzyme assay	0.9 ± 0.8 µM	Vero E6	>100 µM	ND*	ND*	ND*	Hit	Broad-spectrum inhibitors	[85]
7h	0.41 ± 0.1 µM	ND*	FRET enzyme assay	ND	Vero E6	>100 µM	ND*	ND*	ND*	Hit	Broad-spectrum inhibitors	[85]
6j	0.48 ± 0.08 µM	ND*	FRET enzyme assay	0.8 ± 0.7 µM	Vero E6	>100 µM	ND*	ND*	ND*	Hit	Broad-spectrum inhibitors	[85]
7j	0.45 ± 0.1 µM	ND*	FRET enzyme assay	ND	Vero E6	>100 µM	ND*	ND*	ND*	Hit	Broad-spectrum inhibitors	[85]
GC376	0.62 ± 0.08 µM	ND*	FRET enzyme assay	ND	Vero E6	>100 µM	ND*	ND*	ND*	Control		[85]
13B-H (12)	>5 µM	ND*	ND*	ND*	ND*	ND*	ND*	ND*	ND*	Hit		[86]
13b-K (13)	0.12 ± 0.03 µM	ND*	ND*	1.3 µM	Vero E6	ND*	ND*	ND*	ND*	Experimental		[86]
YH-53	0.124 µM	Enzymatic assay	ND*	ND*	ND*	ND*	ND*	ND*	ND*	Hit	benzothiazolyl ketone warhead	[87]
GRL-1720	0.32 ± 0.02 µM	2.15 ± 0.49 µM	FRET-based assay	15 ± 4 µM	Vero E6	>100 µM	ND*	ND*	ND*	Hit	Indole based	[88]
Compound 5h	ND*	17.6 ± 3.2 nM	FRET-based assay	4.2 ± 0.7 µM	Vero E6	>100 µM	ND*	ND*	ND*	Experimental	Indole based	[88]
GC376	0.052 ± 0.007 µm	ND*	FRET assay	>50 µM	Vero E6	10 ± 4.2 µM	ND*	ND*	ND*	Hit	Repurposed	[89]
Calpain Inhibitor II	8.98 ± 2.0 µM	ND*	FRET assay	>100 µM	Vero E6	27 ± 1.4 µM	ND*	ND*	ND*	Hit	Repurposed	[89]
Calpain Inhibitor XII	6.48 ± 3.4 µM	ND*	FRET assay	>50 µM	Vero E6	1.3 ± 0.57 µM	ND*	ND*	ND*	Hit	Repurposed	[89]
Ebselen	>100 µM	ND*	FRET assay	8.23 µM	Vero E6	>100 µM	ND*	ND*	ND*	Hit	Repurposed	[89]
Disulfiram	>100 µM	ND*	FRET assay	8.35 µM	Vero E6	>100 µM	ND*	ND*	ND*	Hit	Repurposed	[89]

Shikonin	15.0 ± 3.0 µM	ND*	FRET assay	0.32 µM	Vero E6	>100 µM	ND*	ND*	ND*	Experimental	Repurposed	[89]
Remdesivir	ND*	ND*	ND*	≥50 µM	Vero E6	ND*	ND*	ND*	ND*	Control	FDA approved	[89]
GS-441524	ND*	ND*	ND*	ND*	Vero E6	<0.8 µM	ND*	ND*	ND*	Control		[89]
GC376	0.19 ± 0.04 µM	ND*	FRET assay	0.9 ± 0.2 µM	Vero E6	>200 µM	ND*	ND*	ND*	Experimental		[90]
GC373	0.40 ± 0.05 µM	ND*	FRET assay	1.5 µM	Vero E6	>200 µM	ND*	ND*	ND*	Hit		[90]
Ebselen	0.67 ± 0.033 µM	ND*	FRET assay	4.67 ± 0.024 µM	Vero E6	ND*	ND*	ND*	ND*	Control	Repurposed	[91]
MR6-7-2	0.363 ± 0.044 µM	ND*	FRET assay	4.50 ± 0.086 µM	Vero E6	ND*	ND*	ND*	ND*	Hit	Ebselen derivatives	[91]
MR6-17-2	0.70 ± 0.032 µM	ND*	FRET assay	ND*	ND*	ND*	ND*	ND*	ND*	Hit	Ebselen derivatives	[91]
MR6-18-4	0.345 ± 0.034 µM	ND*	FRET assay	3.74 ± 0.066 µM	Vero E6	ND*	ND*	ND*	ND*	Hit	Ebselen derivatives	[91]
MR6-26-2	0.467 ± 0.038 µM	ND*	FRET assay	3.17 ± 0.112 µM	Vero E6	ND*	ND*	ND*	ND*	Hit	Ebselen derivatives	[91]
MR6-31-2	0.824 ± 0.044 µM	ND*	FRET assay	1.78 ± 0.073 µM	Vero E6	ND*	ND*	ND*	ND*	Experimental	Ebselen derivatives	[91]
PF-00835231 (4)	0.27 ± 0.1 nM	ND*	ND*	ND*	ND*	ND*	ND*	ND*	ND*	clinical candidate	Ketone-Based Covalent Inhibitors	[92]
Mastinib	2.5 µM	ND*	Enzymatic assay	3.2 µM	A549	>10 µM	ND*	ND*	ND*	Experimental	Repurposed	[93]
GRL-0496	3.8 µM	ND*	Enzymatic assay	ND*	ND*	ND*	ND*	ND*	ND*	Hit	Repurposed	[93]
FB2001	0.053 µM	ND*		0.39 ± 0.01 µM	Vero E6	274.4 µM	ND*	ND*	ND*	Experimental		[94]
Chloroquinine phosphate	ND*	ND*	ND*	4.17 ± 0.05 µM	Vero E6	>100 µM	ND*	ND*	ND*	Hit		[94]
PF-07304814					Vero E6					lead		[95]
PF-00835231	0.0069 µM	0.27 ± 0.1 µM		39.8 µM	Vero E6	>100 µM		P450	14 µL/min/mg	lead		[95]
PF-07321332	ND*	ND*	ND*	0.88 µM	TMPRSS2	>10 µM	ND*	ND*	ND*	Control	narlaprevir- and bocepr	[95]

											evir-derived hybrid inhibitors	
BBH-1	ND*	ND*	ND*	16.1 µM	HEK293-ACE2-TMPRSS2	>10 µM		ND*	ND*	Hit	narlaprevir- and boceprevir-derived hybrid inhibitors	[95]
BBH-2	ND*	ND*	ND*	15.4 µM	HEK293-ACE2-TMPRSS2	>10 µM		ND*	ND*	Hit	narlaprevir- and boceprevir-derived hybrid inhibitors	[95]
NBH-2	ND*	ND*	ND*	13.9 µM	HEK293-ACE2-TMPRSS2	>10 µM		ND*	ND*	Experimental	narlaprevir- and boceprevir-derived hybrid inhibitors	[95]
Compound 9	1.4 µM		Fluorescence 3CLpro Inhibition assay	ND*	ND*	ND*	ND*	ND*	ND*	Hit	Deep learning-based screening	[96]
Compound 13	4.9 µM		Fluorescence 3CLpro Inhibition assay	ND*	ND*	ND*	ND*	ND*	ND*	Hit	Deep learning-based screening	[96]
Compound 20	5.2 µM		Fluorescence 3CLpro Inhibition assay	ND*	ND*	ND*	ND*	ND*	ND*	Hit	Deep learning-based screening	[96]
Compound 21	10.7 µM		Fluorescence 3CLpro Inhibition assay	ND*	ND*	ND*	ND*	ND*	ND*	Hit	Deep learning-based screening	[96]
Compound 22	11.9 µM		Fluorescence 3CLpro Inhibition assay	ND*	ND*	ND*	ND*	ND*	ND*	Hit	Deep learning-based screening	[96]
Compound 27	5.5 µM		Fluorescence 3CLpro Inhibition assay	ND*	ND*	ND*	ND*	ND*	ND*	Hit	Deep learning-based screening	[96]
Tideglusib	0.3 ± 0.02 µM		Fluorescence 3CLpro Inhibition assay	ND*	ND*	ND*	ND*	ND*	ND*	Control		[96]

1	100–250a μM	ND*	Enzymatic assay	ND*	ND*	ND*	ND*	ND*	ND*	Hit	Optimization of Triaryl pyridinone Inhibitors	[97]
2	0.140 ± 0.020 μM	ND*	Enzymatic assay	1.51 ± 0.01 μM	Vero E6	22 ± 7.2 μM	ND*	ND*	ND*	Hit	Optimization of Triaryl pyridinone Inhibitors	[97]
3	0.170 ± 0.022 μM	ND*	Enzymatic assay	1.76 ± 0.31 μM	Vero E6	>100 μM	ND*	ND*	ND*	Hit	Optimization of Triaryl pyridinone Inhibitors	[97]
4	0.120 ± 0.006 μM	ND*	Enzymatic assay	1.20 ± 0.20 μM	Vero E6	22 ± 8 μM	ND*	ND*	ND*	Hit	Optimization of Triaryl pyridinone Inhibitors	[97]
5	0.120 ± 0.016 μM	ND*	Enzymatic assay	ND8	ND*	ND*	ND*	ND*	ND*	Hit	Optimization of Triaryl pyridinone Inhibitors	[97]
6	0.037 ± 0.004 μM	ND*	Enzymatic assay	ND*	ND*	ND*	ND*	ND*	ND*	Hit	Optimization of Triaryl pyridinone Inhibitors	[97]
7	0.025 ± 0.003 μM	ND*	Enzymatic assay	ND*	ND*	ND*	ND*	ND*	ND*	Hit	Optimization of Triaryl pyridinone Inhibitors	[97]
8	0.328 ± 0.061 μM	ND*	Enzymatic assay	ND*	ND*	ND*	ND*	ND*	ND*	Hit	Optimization of Triaryl pyridinone Inhibitors	[97]
9	0.083 ± 0.023 μM	ND*	Enzymatic assay	0.73 ± 0.26 μM	Vero E6	ND*	ND*	ND*	ND*	Hit	Optimization of Triaryl pyridinone Inhibitors	[97]
10	0.085 ± 0	ND*	Enzymatic assay	ND*	ND*	ND*	ND*	ND*	ND*	Hit	Optimization of	[97]

	.005 μM										Triarylpyridinone Inhibitors	
11	0.131 ± 0.009 μM	ND*	Enzymatic assay	ND*	ND*	ND*	ND*	ND*	ND*	Hit	Optimization of Triarylpyridinone Inhibitors	[97]
12	0.027 ± 0.004 μM	ND*	Enzymatic assay	ND*	ND*	ND*	ND*	ND*	ND*	Hit	Optimization of Triarylpyridinone Inhibitors	[97]
13	0.042 ± 0.015 μM	ND*	Enzymatic assay	>40 μM	Vero E6	ND*	ND*	ND*	ND*	Hit	Optimization of Triarylpyridinone Inhibitors	[97]
14	0.105 ± 0.013 μM	ND*	Enzymatic assay	NAd	Vero E6	>100 μM	ND*	ND*	ND*	Hit	Optimization of Triarylpyridinone Inhibitors	[97]
15	0.038 ± 0.015 μM	ND*	Enzymatic assay	NAd	Vero E6	>100 μM	ND*	ND*	ND*	Hit	Optimization of Triarylpyridinone Inhibitors	[97]
16	0.061 ± 0.007 μM	ND*	Enzymatic assay	0.53 ± 0.23 μM	Vero E6	>100 μM	ND*	ND*	ND*	Hit	Optimization of Triarylpyridinone Inhibitors	[97]
17	0.059 ± 0.014 μM	ND*	Enzymatic assay	0.82 ± 0.28 μM	Vero E6	82 μM	ND*	ND*	ND*	Hit	Optimization of Triarylpyridinone Inhibitors	[97]
18	0.028 ± 0.007 μM	ND*	Enzymatic assay	3.2 ± 1.0 μM	Vero E6	>100 μM	ND*	ND*	ND*	Hit	Optimization of Triarylpyridinone Inhibitors	[97]
19	0.044 ± 0.009 μM	ND*	Enzymatic assay	0.175 ± 0.005 μM	Vero E6	>80 μM	ND*	ND*	ND*	Experimental	Optimization of Triarylpyridinone	[97]

											Inhibitors	
20	0.130 ± 0.018 µM	ND*	Enzymatic assay			>32.5 µM	ND*	ND*	ND*	Hit	Optimization of Triarylpyridinone Inhibitors	[97]
21	0.061 ± 0.011 µM	ND*	Enzymatic assay	1.08 µM	Vero E6	>100 µM	ND*	ND*	ND*	Hit	Optimization of Triarylpyridinone Inhibitors	[97]
Remdesivir	ND*	ND*	ND*	0.59 ± 0.25 µM	Vero E6	72 ± 28 µM	ND*	ND*	ND*	Control		[97]
ML 188 (1)	10.96 ± 1.58 Mm	ND*	Enzymatic assay	>20 µM	Vero E6	>125 µM	ND*	ND*	ND*	Hit	Rational design of non-covalent inhibitors	[98]
GC376	0.033 ± 0.003 µM	ND*	Enzymatic assay	1.51 ± 0.27 µM	Vero E6	>125 µM	ND*	ND*	ND*	Hit	Aldehyde prodrug	[98]
20	0.94 ± 0.33 µM	ND*	Enzymatic assay	1.73 ± 0.19 µM	Vero E6	>200 µM	ND*	ND*	ND*	Hit	Rational design of non-covalent inhibitors	[98]
23	0.66 ± 0.07 µM	ND*	Enzymatic assay	1.61 ± 0.26 µM	Vero E6	>200 µM	ND*	ND*	ND*	Hit	Rational design of non-covalent inhibitors	[98]
24	0.96 ± 0.09 µM	ND*	Enzymatic assay	13.06 ± 2.30 µM	Vero E6	129.38 ± 25.12 µM	ND*	ND*	ND*	Hit	Rational design of non-covalent inhibitors	[98]
37	0.67 ± 0.15 µM	ND*	Enzymatic assay	4.54 ± 0.69 µM	Vero E6	147.8 ± 13.9 µM	ND*	ND*	ND*	Hit	Rational design of non-covalent inhibitors	[98]
38	0.28 ± 0.07 µM	ND*	Enzymatic assay	0.82 ± 0.65 µM	Vero E6	>200 µM	ND*	ND*	ND*	Hit	Rational design of non-covalent inhibitors	[98]

40	1.05 ± 0. 09 μM	ND*	Enzymati c assay	2.04 ± 1. 08 μM	Vero E6	>200 μM	ND*	ND*	ND*	Hit	Ration al design of non-covale nt inhibit ors	[98]
23R	0.20 ± 0. 01 μM	ND*	Enzymati c assay	1.27 ± 0. 09 μM	Vero E6	>100 μM	ND*	ND*	ND*	Experime ntal	Ration al design of non-covale nt inhibit ors	[98]
23S	>20 μM	ND*	Enzymati c assay	ND*	Vero E6	ND*	ND*	ND*	ND*	Hit	Ration al design of non-covale nt inhibit ors	[98]
E01	0.33 ± 0.03 Mm	ND*	FRET assay	>20 μM	Vero E6	ND*	ND*	ND*	ND*	Hit	2-Phenyl -1,2-benzos elenazo l-3-one analog ues	[99]
E02	2.78 ± 1.0 μM	ND*	FRET assay	16.7 ± 4.8	Vero E6	ND*	ND*	ND*	ND*	Hit	2-Phenyl -1,2-benzos elenazo l-3-one analog ues	[99]
E03	>10 μM	ND*	FRET assay	>20 μM	Vero E6	ND*	ND*	ND*	ND*	Hit	2-Phenyl -1,2-benzos elenazo l-3-one analog ues	[99]
E04	0.90 ± 0.17 μM	ND*	FRET assay	11.2 ± 1.3 μM	Vero E6	ND*	ND*	ND*	3.19	Hit	2-Phenyl -1,2-benzos elenazo l-3-one analog ues	[99]
E05	>10 μM	ND*	FRET assay	12.2 ± 1.7 μM	Vero E6	ND*	ND*	ND*	ND*	Hit	2-Phenyl -1,2-benzos elenazo l-3-one analog ues	[99]
E06	1.00 ± 0.29 μM	ND*	FRET assay	>20 μM	Vero E6	ND*	ND*	ND*	ND*	Hit	2-Phenyl -1,2-benzos elenazo l-3-one analog ues	[99]
E07	0.61 ±	ND*	FRET assay	26.5 ± 1.2 μM	Vero E6	ND*	ND*	ND*	0.85	Hit	2-Phenyl -1,2-	[99]

	0.44 μM										benzoselenazol-3-one analogues	
E08	>10 μM	ND*	FRET assay	>20 μM	Vero E6	ND*	ND*	ND*	ND*	Hit	2-Phenyl-1,2-benzoselenazol-3-one analogues	[99]
E09	>10 μM	ND*	FRET assay	11.2 ± 3.7 μM	Vero E6	ND*	ND*	ND*	ND*	Hit	2-Phenyl-1,2-benzoselenazol-3-one analogues	[99]
E10	>10 μM	ND*	FRET assay	>20 μM	Vero E6	ND*	ND*	ND*	ND*	Hit	2-Phenyl-1,2-benzoselenazol-3-one analogues	[99]
E11	2.09 ± 0.97 μM	ND*	FRET assay	>20 μM	Vero E6	ND*	ND*	ND*	ND*	Hit	2-Phenyl-1,2-benzoselenazol-3-one analogues	[99]
E12	>10 μM	ND*	FRET assay	>20 μM	Vero E6	ND*	ND*	ND*	ND*	Hit	2-Phenyl-1,2-benzoselenazol-3-one analogues	[99]
E13	>10 μM	ND*	FRET assay	>20 μM	Vero E6	ND*	ND*	ND*	ND*	Hit	2-Phenyl-1,2-benzoselenazol-3-one analogues	[99]
E14	>10 μM	ND*	FRET assay	>20 μM	Vero E6	ND*	ND*	ND*	ND*	Hit	2-Phenyl-1,2-benzoselenazol-3-one analogues	[99]
E15	6.40 ± 0.78 μM	ND*	FRET assay	11.2 ± 1.3 μM	Vero E6	ND*	ND*	ND*	ND*	Hit	2-Phenyl-1,2-benzoselenazol-3-one analogues	[99]
E16	3.30 ± 0.74 μM	ND*	FRET assay	>20 μM	Vero E6	ND*	ND*	ND*	ND*	Hit	2-Phenyl-1,2-benzoselenazol-3-one	[99]

											analogues	
E17	1.80 ± 0.88 µM	ND*	FRET assay	>20 µM	Vero E6	ND*	ND*	ND*	ND*	Hit	2-Phenyl-1,2-benzoselenazol-3-one analogues	[99]
E18	6.18 ± 1.74 µM	ND*	FRET assay	6.5 ± 2.0 µM	Vero E6	ND*	ND*	ND*	2.87	Hit	2-Phenyl-1,2-benzoselenazol-3-one analogues	[99]
E19	0.68 ± 0.28 µM	ND*	FRET assay	17.4 ± 3.5 µM	Vero E6	ND*	ND*	ND*	1.53	Hit	2-Phenyl-1,2-benzoselenazol-3-one analogues	[99]
E20	0.64 ± 0.10 µM	ND*	FRET assay	18.2 ± 3.6 µM	Vero E6	ND*	ND*	ND*	1.67	Hit	2-Phenyl-1,2-benzoselenazol-3-one analogues	[99]
E21	2.77 ± 0.67 µM	ND*	FRET assay	5.2 ± 1.8 µM	Vero E6	ND*	ND*	ND*	1.46	Hit	2-Phenyl-1,2-benzoselenazol-3-one analogues	[99]
E22	2.29 ± 0.95 µM	ND*	FRET assay	15.4 ± 1.5 µM	Vero E6	ND*	ND*	ND*	ND*	Hit	2-Phenyl-1,2-benzoselenazol-3-one analogues	[99]
E23	7.43 ± 0.77 µM	ND*	FRET assay	20.7 ± 2.6 µM	Vero E6	ND*	ND*	ND*	ND*	Hit	2-Phenyl-1,2-benzoselenazol-3-one analogues	[99]
E24	2.77 ± 0.51 µM	ND*	FRET assay	0.8 ± 0.3 µM	Vero E6	ND*	ND*	ND*	3.18	Experimental	2-Phenyl-1,2-benzoselenazol-3-one analogues	[99]
E25	0.38 ± 0.08 µM	ND*	FRET assay	2.0 ± 1.1 µM	Vero E6	ND*	ND*	ND*	1.45	Hit	2-Phenyl-1,2-benzoselenazol-3-one analogues	[99]

E26	>10 µM	ND*	FRET assay	15.1 ± 3.1 µM	Vero E6	ND*	ND*	ND*	ND*	Hit	2-Phenyl-1,2-benzoselenazol-3-one analogues	[99]
E27	1.25 ± 0.35 µM	ND*	FRET assay	1.7 ± 2.7 µM	Vero E6	ND*	ND*	ND*	ND*	Hit	2-Phenyl-1,2-benzoselenazol-3-one analogues	[99]
E28	5.56 ± 1.85 µM	ND*	FRET assay	17.3 ± 3.7 µM	Vero E6	ND*	ND*	ND*	ND*	Hit	2-Phenyl-1,2-benzoselenazol-3-one analogues	[99]
E29	2.69 ± 0.24 µM	ND*	FRET assay	>20 µM	Vero E6	ND*	ND*	ND*	ND*	Hit	2-Phenyl-1,2-benzoselenazol-3-one analogues	[99]
E30	>10 µM	ND*	FRET assay	>20 µM	Vero E6	ND*	ND*	ND*	ND*	Hit	2-Phenyl-1,2-benzoselenazol-3-one analogues	[99]
Ebselen	ND*	ND*	ND*	>20 µM	Vero E6	ND*	ND*	ND*	0.92	Lead	Repurposed	[99]
Remdesivir	ND*	ND*	ND*	1.8 µM	Vero E6	ND*	ND*	ND*	ND*	Control	FDA approved	[99]
10	6.6 ± 2.7 µM	ND*	Enzymatic assay	ND*	ND*	ND*	ND*	ND*	ND*	Hit	Penecilin V derivatives	[100]
20e	9.0 ± 1.3 µM	ND*	Enzymatic assay	ND*	ND*	ND*	ND*	ND*	ND*	Hit	Penecilin V derivatives	[100]
20f	7.9 ± 1.4 µM	ND*	Enzymatic assay	ND*	ND*	ND*	ND*	ND*	ND*	Hit	Penecilin V derivatives	[100]
20g	5.2 ± 0.6 µM	ND*	Enzymatic assay	ND*	ND*	ND*	ND*	ND*	ND*	Hit	Penecilin V derivatives	[100]
20h	3.0 ± 0.4 µM	ND*	Enzymatic assay	ND*	ND*	ND*	ND*	ND*	ND*	Hit	Penecilin V derivatives	[100]
20i	9.7 ± 3.3 µM	ND*	Enzymatic assay	ND*	ND*	ND*	ND*	ND*	ND*	Hit	Penecilin V derivatives	[100]
25h	5.3 ± 3.3 µM	ND*	Enzymatic assay	ND*	ND*	ND*	ND*	ND*	ND*	Hit	Penecilin V derivatives	[100]

25k	2.8 ± 1.2 µM	ND*	Enzymatic assay	ND*	ND*	ND*	ND*	ND*	ND*	Hit	Penecilin V derivatives	[100]
25l	2.3 ± 1.2	ND*	Enzymatic assay	ND*	ND*	ND*	ND*	ND*	ND*	Hit	Penecilin V derivatives	[100]
25m	4.1 ± 0.1 µM	ND*	Enzymatic assay	ND*	ND*	ND*	ND*	ND*	ND*	Hit	Penecilin V derivatives	[100]
28	0.7 ± 0.1 µM	ND*	Enzymatic assay	ND*	ND*	ND*	ND*	ND*	ND*	Hit	Penecilin V derivatives	[100]
31	0.6 ± 0.1 µM	ND*	Enzymatic assay	ND*	ND*	ND*	ND*	ND*	ND*	Hit	Penecilin V derivatives	[100]
32	0.5 ± 0.1 µM	ND*	Enzymatic assay	ND*	ND*	ND*	ND*	ND*	ND*	Hit	Penecilin V derivatives	[100]
Disulfiram	1.04 ± 0.03 µM	ND*	Enzymatic assay	ND*	ND*	ND*	ND*	ND*	ND*	Control	Repurposed	[101]
B1	8.54 ± 0.94 µM	ND*	Enzymatic assay	ND*	ND*	ND*	ND*	ND*	ND*	Hit	9,10-dihydrophenanthrene derivatives	[101]
B2	2.46 ± 0.14 µM	ND*	Enzymatic assay	ND*	ND*	ND*	ND*	ND*	ND*	Hit	9,10-dihydrophenanthrene derivatives	[101]
B3	9.82 ± 1.16 µM	ND*	Enzymatic assay	ND*	ND*	ND*	ND*	ND*	ND*	Hit	9,10-dihydrophenanthrene derivatives	[101]
B4	4.79 ± 0.22 µM	ND*	Enzymatic assay	ND*	ND*	ND*	ND*	ND*	ND*	Hit	9,10-dihydrophenanthrene derivatives	[101]
B5	10.26 ± 0.66 µM	ND*	Enzymatic assay	ND*	ND*	ND*	ND*	ND*	ND*	Hit	9,10-dihydrophenanthrene derivatives	[101]
B6	3.32 ± 0.25 µM	ND*	Enzymatic assay	ND*	ND*	ND*	ND*	ND*	ND*	Hit	9,10-dihydrophenanthrene derivatives	[101]
B7	1000 ± 0.47 µM	ND*	Enzymatic assay	ND*	ND*	ND*	ND*	ND*	ND*	Hit	9,10-dihydrophenanthrene derivatives	[101]
B8	11.23 ± 0.70 µM	ND*	Enzymatic assay	ND*	ND*	ND*	ND*	ND*	ND*	Hit	9,10-dihydrophenanthrene	[101]

											derivatives	
B9	7.39 ± 0.11 μM	ND*	Enzymatic assay	ND*	ND*	ND*	ND*	ND*	ND*	Hit	9,10-dihydrophenanthrene derivatives	[101]
B10	5.25 ± 0.24 μM	ND*	Enzymatic assay	ND*	ND*	ND*	ND*	ND*	ND*	Hit	9,10-dihydrophenanthrene derivatives	[101]
B11	4.29 ± 0.62 μM	ND*	Enzymatic assay	ND*	ND*	ND*	ND*	ND*	ND*	Hit	9,10-dihydrophenanthrene derivatives	[101]
B12	2.72 ± 0.23 μM	ND*	Enzymatic assay	ND*	ND*	ND*	ND*	ND*	ND*	Hit	9,10-dihydrophenanthrene derivatives	[101]
B13	6.73 ± 1.29 μM	ND*	Enzymatic assay	ND*	ND*	ND*	ND*	ND*	ND*	Hit	9,10-dihydrophenanthrene derivatives	[101]
B14	2.78 ± 0.18 μM	ND*	Enzymatic assay	ND*	ND*	ND*	ND*	ND*	ND*	Hit	9,10-dihydrophenanthrene derivatives	[101]
B15	8.89 ± 3.34 μM	ND*	Enzymatic assay	ND*	ND*	ND*	ND*	ND*	ND*	Hit	9,10-dihydrophenanthrene derivatives	[101]
B16	6.65 ± 0.26 μM	ND*	Enzymatic assay	ND*	ND*	ND*	ND*	ND*	ND*	Hit	9,10-dihydrophenanthrene derivatives	[101]
B17	5.83 ± 0.34 μM	ND*	Enzymatic assay	ND*	ND*	ND*	ND*	ND*	ND*	Hit	9,10-dihydrophenanthrene derivatives	[101]
B18	3.66 ± 0.17 μM	ND*	Enzymatic assay	ND*	ND*	ND*	ND*	ND*	ND*	Hit	9,10-dihydrophenanthrene derivatives	[101]
B19	8.28 ± 1.88 μM	ND*	Enzymatic assay	ND*	ND*	ND*	ND*	ND*	ND*	Hit	9,10-dihydrophenanthrene derivatives	[101]
B20	3.31 ± 0.36 μM	ND*	Enzymatic assay	ND*	ND*	ND*	ND*	ND*	ND*	Hit	9,10-dihydrophenanthrene derivatives	[101]

C1	1.55 ±0.21 μM	6.09 μM	Enzymatic assay	ND*	ND*	ND*	ND*	ND*	ND*	Experimental	9,10-dihydrophenanthrene derivatives	[101]
C2	1.81 ± 0.17 μM	7.64 μM	Enzymatic assay	ND*	ND*	ND*	ND*	ND*	ND*	Hit	9,10-dihydrophenanthrene derivatives	[101]
C3	10.23 ± 3.50 μM	ND*	Enzymatic assay	ND*	ND*	ND*	ND*	ND*	ND*	Hit	9,10-dihydrophenanthrene derivatives	[101]
C4	5.39 ± 0.65 μM	ND*	Enzymatic assay	ND*	ND*	ND*	ND*	ND*	ND*	Hit	9,10-dihydrophenanthrene derivatives	[101]
C5	4.71 ± 0.20 μM	ND*	Enzymatic assay	ND*	ND*	ND*	ND*	ND*	ND*	Hit	9,10-dihydrophenanthrene derivatives	[101]
C6	10.68 ± 0.46 μM	ND*	Enzymatic assay	ND*	ND*	ND*	ND*	ND*	ND*	Hit	9,10-dihydrophenanthrene derivatives	[101]
C7	5.50 ± 0.35 μM	ND*	Enzymatic assay	ND*	ND*	ND*	ND*	ND*	ND*	Hit	9,10-dihydrophenanthrene derivatives	[101]
Atovaquone	ND*	ND*	ND*	1.20 ± 0.84 μM	Vero E6	> 100 μM	ND*	ND*	ND*	Hit	Repurposed	[41]
Mebendazole	19.94 ± 1.06 μM	ND*	Enzymatic assay	1.30 ± 0.95 μM	Vero E6	> 100 μM	ND*	ND*	ND*	Hit	Repurposed	[41]
Dronedarone	18.03 ± 1.15 μM	ND*	Enzymatic assay	1.50 ± 0.86 μM	Vero E6	22.70 ± 0.91 μM	ND*	ND*	ND*	Hit	Repurposed	[41]
Ouabain	ND*	ND*	ND*	0.03 ± 0.89 μM	Vero E6	> 100 μM	ND*	ND*	ND*	Experimental	Repurposed	[41]
Digoxin	ND*	ND*	ND*	0.07 ± 0.91 μM	Vero E6	> 100 μM	ND*	ND*	ND*	Hit	Repurposed	[41]
Entacapone	9.04 ± 1.04 μM	ND*	Enzymatic assay	ND*	Vero E6	> 100 μM	ND*	ND*	ND*	Hit	Repurposed	[41]
1	ND*	ND*	ND*	16.8 μM	Vero E6	>133 μM	ND*	ND*	ND*	Hit	Targeting proteases and polymerase	[102]
2	0.18 μM	ND*	Enzymatic assay	ND*	ND*	ND*	ND*	ND*	ND*	Hit	Targeting	[102]

											proteases and polymerase	
3	0.67 μM	ND*	Enzymatic assay	4-5 μM	Calu3	ND*	ND*	ND*	ND*	Hit	Targeting proteases and polymerase	[102]
4	1.34 μM	ND*	Enzymatic assay	>40 μM	Calu3	ND*	ND*	ND*	ND*	Hit	Targeting proteases and polymerase	[102]
5	53 nM	ND*	Enzymatic assay	0.52 μM	Vero E6	>100 μM	ND*	ND*	ND*	Experimental	Targeting proteases and polymerase	[102]
6	40 nM	ND*	Enzymatic assay	0.72 μM	Vero E6	>100 μM	ND*	ND*	ND*	Hit	Targeting proteases and polymerase	[102]
7 (GC376)	30 nM	ND*	Enzymatic assay	3.37 μM	Vero E6	>100 μM	ND*	ND*	ND*	Hit	Targeting proteases and polymerase	[102]
Boceprevir	4.13 μM	1.18 μM	Enzymatic assay	1.95 a μM	Vero E6	>100 μM	ND*	ND*	ND*	Lead	Clinical candidate	[102]
Baicalein	0.94 μM	ND*	Enzymatic assay	1.69 μM	Vero E6	>200 μM	ND*	ND*	ND*	Hit	Repurposed	[102]
Baicalin	6.41 μM	ND*	Enzymatic assay	10.27 μM	Vero E6	>200 μM	ND*	ND*	ND*	Hit	Repurposed	[102]
A1	194.8 nM	ND*	FRET-based assay	ND*	ND*	ND*	ND*	ND*	3.34*	Hit	Pyrimidone derivatives	[103]
A2	20.9 nM	ND*	FRET-based assay	ND*	ND*	ND*	ND*	ND*	3.48*	Hit	Pyrimidone derivatives	[103]
A3	37.0 nM	ND*	FRET-based assay	ND*	ND*	ND*	ND*	ND*	3.63*	Hit	Pyrimidone derivatives	[103]
A4	18.8 nM	ND*	FRET-based assay	ND*	ND*	ND*	ND*	ND*	3.78*	Hit	Pyrimidone derivatives	[103]
A5	226.8 nM	ND*	FRET-based assay	ND*	ND*	ND*	ND*	ND*	3.93*	Hit	Pyrimidone derivatives	[103]
A6	38.3 nM	ND*	FRET-based assay	ND*	ND*	ND*	ND*	ND*	4.36*	Hit	Pyrimidone derivatives	[103]
A7	60.8 nM	ND*	FRET-based assay	ND*	ND*	ND*	ND*	ND*	3.19*	Hit	Pyrimidone derivatives	[103]
A8	185.6 nM	ND*	FRET-based assay	ND*	ND*	ND*	ND*	ND*	3.76*	Hit	Pyrimidone derivatives	[103]

A9	287.1 nM	ND*	FRET-based assay	ND*	ND*	ND*	ND*	ND*	5.09*	Hit	Pyrimidone derivatives	[103]
S-217622b	20.3 nM	ND*	FRET-based assay	ND*	ND*	ND*	ND*	ND*	4.11*	Control	Clinical candidate	[103]
A10	24.7 nM	ND*	FRET-based assay	ND*	ND*	ND*	ND*	ND*	3.78	Hit	Pyrimidone derivatives	[103]
A11	>1000 nM	ND*	FRET-based assay	ND*	ND*	ND*	ND*	ND*	3.96	Hit	Pyrimidone derivatives	[103]
A12	>1000 nM	ND*	FRET-based assay	ND*	ND*	ND*	ND*	ND*	3.63	Hit	Pyrimidone derivatives	[103]
A13	>1000 nM	ND*	FRET-based assay	ND*	ND*	ND*	ND*	ND*	4.19	Hit	Pyrimidone derivatives	[103]
A14	>1000 nM	ND*	FRET-based assay	ND*	ND*	ND*	ND*	ND*	4.31	Hit	Pyrimidone derivatives	[103]
A15	81.9 nM	ND*	FRET-based assay	ND*	ND*	ND*	ND*	ND*	4.91	Hit	Pyrimidone derivatives	[103]
A16	>1000 nM	ND*	FRET-based assay	ND*	ND*	ND*	ND*	ND*	3.35	Hit	Pyrimidone derivatives	[103]
A17	>1000 nM	ND*	FRET-based assay	ND*	ND*	ND*	ND*	ND*	4.87	Hit	Pyrimidone derivatives	[103]
A18	>1000 nM	ND*	FRET-based assay	ND*	ND*	ND*	ND*	ND*	0.70	Hit	Pyrimidone derivatives	[103]
A19	>1000 nM	ND*	FRET-based assay	ND*	ND*	ND*	ND*	ND*	2.69	Hit	Pyrimidone derivatives	[103]
A20	15.7 nM	ND*	FRET-based assay	ND*	ND*	ND*	ND*	ND*	3.74	Hit	Pyrimidone derivatives	[103]
A21	26.8 nM	ND*	FRET-based assay	ND*	ND*	ND*	ND*	ND*	4.35	Hit	Pyrimidone derivatives	[103]
A22	555.6 nM	ND*	FRET-based assay	ND*	ND*	ND*	ND*	ND*	3.21	Hit	Pyrimidone derivatives	[103]
A23	206.4 nM	ND*	FRET-based assay	ND*	ND*	ND*	ND*	ND*	4.54	Hit	Pyrimidone derivatives	[103]
A24	14.1 nM	ND*	FRET-based assay	ND*	ND*	ND*	ND*	ND*	5.88	Hit	Pyrimidone derivatives	[103]
A25	42.2 nM	ND*	FRET-based assay	ND*	ND*	ND*	ND*	ND*	5.46	Hit	Pyrimidone derivatives	[103]

A26	24.3 nM	ND*	FRET-based assay	ND*	ND*	ND*	ND*	ND*	5.04	Hit	Pyrimidone derivatives	[103]
A27	48.8 nM	ND*	FRET-based assay	ND*	ND*	ND*	ND*	ND*	4.62	Hit	Pyrimidone derivatives	[103]
A28	26.5 nM	ND*	FRET-based assay	ND*	ND*	ND*	ND*	ND*	4.13	Hit	Pyrimidone derivatives	[103]
A29	30.7 nM	ND*	FRET-based assay	ND*	ND*	ND*	ND*	ND*	4.93	Hit	Pyrimidone derivatives	[103]
A30	33.3 nM	ND*	FRET-based assay	ND*	ND*	ND*	ND*	ND*	5.14	Hit	Pyrimidone derivatives	[103]
A31	31.8 nM	ND*	FRET-based assay	ND*	ND*	ND*	ND*	ND*	3.66	Hit	Pyrimidone derivatives	[103]
A32	105.4 nM	ND*	FRET-based assay	ND*	ND*	ND*	ND*	ND*	7.34	Hit	Pyrimidone derivatives	[103]
A33	27.1 nM	ND*	FRET-based assay	ND*	ND*	ND*	ND*	ND*	5.84	Experimental	Pyrimidone derivatives	[103]
A34	11.4 nM	ND*	FRET-based assay	ND*	ND*	ND*	ND*	ND*	5.77	Hit	Pyrimidone derivatives	[103]
A35	55.6 nM	ND*	FRET-based assay	ND*	ND*	ND*	ND*	ND*	5.35	Hit	Pyrimidone derivatives	[103]
A36	51.3 nM	ND*	FRET-based assay	ND*	ND*	ND*	ND*	ND*	4.54	Hit	Pyrimidone derivatives	[103]
A37	29.2 nM	ND*	FRET-based assay	ND*	ND*	ND*	ND*	ND*	5.50	Hit	Pyrimidone derivatives	[103]
A38	55.2 nM	ND*	FRET-based assay	ND*	ND*	ND*	ND*	ND*	5.79	Hit	Pyrimidone derivatives	[103]
A39	51.9 nM	ND*	FRET-based assay	ND*	ND*	ND*	ND*	ND*	5.37	Hit	Pyrimidone derivatives	[103]
A40	40.5 nM	ND*	FRET-based assay	ND*	ND*	ND*	ND*	ND*	4.95	Hit	Pyrimidone derivatives	[103]
A41	27.4 nM	ND*	FRET-based assay	ND*	ND*	ND*	ND*	ND*	3.62	Hit	Pyrimidone derivatives	[103]
A42	17.7 nM	ND*	FRET-based assay	ND*	ND*	ND*	ND*	ND*	4.60	Hit	Pyrimidone derivatives	[103]
A43	29.6 nM	ND*	FRET-based assay	ND*	ND*	ND*	ND*	ND*	3.55	Hit	Pyrimidone derivatives	[103]

WU-01	648 ± 68 nM	ND*	FRET-based assay	ND*	ND*	ND*	ND*	ND*	ND*	Hit	DNA encoded library	[104]
WU-02	71 ± 4 nM	ND*	FRET-based assay	54 ± 10 nM	A549-hACE2	>20	ND*	ND*	ND*	Hit	DNA encoded library	[104]
WU-03	ND*	ND*	FRET-based assay	ND*	ND*	ND*	ND*	ND*	ND*	Hit	DNA encoded library	[104]
WU-04	72 ± 6 nM	ND*	FRET-based assay	12 ± 1 nM	A549-hACE2	>20	ND*	ND*	ND*	Experimental	DNA encoded library	[104]
WU-05	122 ± 16 nM	ND*	FRET-based assay	ND*	ND*	ND*	ND*	ND*	ND*	Hit	DNA encoded library	[104]
WU-06	ND*	ND*	FRET-based assay	ND*	ND*	ND*	ND*	ND*	ND*	Hit	Analogues of WU-04	[104]
WU-07	239 ± 16 nM	ND*	FRET-based assay	ND*	ND*	ND*	ND*	ND*	ND*	Hit	Analogues of WU-04	[104]
WU-08	467 ± 64 nM	ND*	FRET-based assay	ND*	ND*	ND*	ND*	ND*	ND*	Hit	Analogues of WU-04	[104]
WU-09	221 ± 35 nM	ND*	FRET-based assay	ND*	ND*	ND*	ND*	ND*	ND*	Hit	Analogues of WU-04	[104]
WU-10	135 ± 8 nM	ND*	FRET-based assay	ND*	ND*	ND*	ND*	ND*	ND*	Hit	Analogues of WU-04	[104]
WU-11	849 ± 161 nM	ND*	FRET-based assay	ND*	ND*	ND*	ND*	ND*	ND*	Hit	Analogues of WU-04	[104]
WU-12	6112 ± 2635 nM	ND*	FRET-based assay	ND*	ND*	ND*	ND*	ND*	ND*	Hit	Analogues of WU-04	[104]
PF-07321332	ND*	ND*	FRET-based assay	117 ± 9 nM	A549-hACE2	ND*	ND*	ND*	ND*	Control	FDA approved	[104]
2c	ND*	2.95 ± 0.29 µM	Enzymatic assay	10.3 ± 3.9 µM	Huh-7-ACE2	>100 µM	ND*	ND*	ND*	Hit	Dual inhibitors	[52]
2f	ND*	1.61 ± 0.21 µM	Enzymatic assay	ND*	Huh-7-ACE2	12.6 ± 7.7 µM	ND*	ND*	ND*	Hit	Dual inhibitors	[52]
3f	ND*	3.66 ± 0.23 µM	Enzymatic assay	ND*	Huh-7-ACE2	22.6 ± 2.3 µM	ND*	ND*	ND*	Hit	Dual inhibitors	[52]
4b	ND*	5.58 ± 0.32 µM	Enzymatic assay	7.63 ± 2.2 µM	Huh-7-ACE2	>100 µM	ND*	ND*	ND*	Hit	Dual inhibitors	[52]
4d	ND*	5.54 ± 0.31 µM	Enzymatic assay	3.50 ± 1.1 µM	Huh-7-ACE2	>100 µM	ND*	ND*	ND*	Experimental	Dual inhibitors	[52]
4e	ND*	10.72 ± 0.84 µM	Enzymatic assay	ND*	Huh-7-ACE2	>100 µM	ND*	ND*	ND*	Hit	Dual inhibitors	[52]
4f	ND*	3.14 ± 0.33 µM	Enzymatic assay	ND*	Huh-7-ACE2	46.9 ± 11.2 µM	ND*	ND*	ND*	Hit	Dual inhibitors	[52]
SPR3926	ND*	1.8 µM	Enzymatic assay	1.5 µM	Huh-7-ACE2	100 µM	ND*	ND*	ND*	Control	Dual inhibitors	[52]
Nirmatrelvir	ND*	0.003 µM	Enzymatic assay	ND*	ND*	ND*	ND*	ND*	ND*	FDA-approved	Peptidomimetic	[52]

											derivative	
1 (Nirmatrelvir)	ND*	0.26 nM	FRET-based assay	2.0 µM	Vero E6	ND*	ND*	ND*	ND*	FDA-approved	Exploring P1 and P4 of Nirmatrelvir	[105]
5a	ND*	0.44 nM	FRET-based assay	4.0 µM	Vero E6	ND*	ND*	ND*	ND*	Hit	Exploring P1 and P4 of Nirmatrelvir	[105]
5b	ND*	36.9 nM	FRET-based assay	17 µM	Vero E6	ND*	ND*	ND*	ND*	Hit	Exploring P1 and P4 of Nirmatrelvir	[105]
5c	ND*	22.1 nM	FRET-based assay	2.3 µM	Vero E6	ND*	ND*	ND*	ND*	Hit	Exploring P1 and P4 of Nirmatrelvir	[105]
5d	ND*	0.2 nM	FRET-based assay	1.6 µM	Vero E6	ND*	ND*	ND*	ND*	Hit	Exploring P1 and P4 of Nirmatrelvir	[105]
5e	ND*	0.04 nM	FRET-based assay	0.26 µM	Vero E6	ND*	ND*	ND*	ND*	Experimental	Exploring P1 and P4 of Nirmatrelvir	[105]
5f	ND*	1.1 nM	FRET-based assay	0.27 µM	Vero E6	ND*	ND*	ND*	ND*	Hit	Exploring P1 and P4 of Nirmatrelvir	[105]
5g	ND*	323 nM	FRET-based assay	100 µM	Vero E6	ND*	ND*	ND*	ND*	Hit	Exploring P1 and P4 of Nirmatrelvir	[105]
5h	ND*	7.7 nM	FRET-based assay	30.7 µM	Vero E6	ND*	ND*	ND*	ND*	Hit	Exploring P1 and P4 of Nirmatrelvir	[105]
5i	ND*	0.025 nM	FRET-based assay	0.29 µM	Vero E6	ND*	ND*	ND*	ND*	Hit	Exploring P1 and P4 of Nirmatrelvir	[105]
5j	ND*	32.2 nM	FRET-based assay	29.7 µM	Vero E6	ND*	ND*	ND*	ND*	Hit	Exploring P1 and P4 of Nirmatrelvir	[105]
5k	ND*	0.09 nM	FRET-based assay	0.035 µM	Vero E6	ND*	ND*	ND*	ND*	Hit	Exploring P1 and P4 of Nirmatrelvir	[105]

5l	ND*	0.49 nM	FRET-based assay	40.1 µM	Vero E6	ND*	ND*	ND*	ND*	Hit	Exploring P1 and P4 of Nirmatrelvir	[105]
5m	ND*	3.3 nM	FRET-based assay	100 µM	Vero E6	ND*	ND*	ND*	ND*	Hit	Exploring P1 and P4 of Nirmatrelvir	[105]
5n	ND*	0.094 nM	FRET-based assay	3.4 µM	Vero E6	ND*	ND*	ND*	ND*	Hit	Exploring P1 and P4 of Nirmatrelvir	[105]
5o	nD*	7.8 nM	FRET-based assay	27.1 µM	Vero E6	ND*	ND*	ND*	ND*	Hit	Exploring P1 and P4 of Nirmatrelvir	[105]
Compound 9	5.7 µM	ND*	FRET assay	ND*	ND*	ND*	ND*	ND*	ND*	Hit	Structure-Based optimization of S2 binding	[51]
Compound 14	4.4 µM	ND*	FRET assay	ND*	ND*	ND*	ND*	ND*	ND*	Hit	Structure-Based optimization of S2 binding	[51]
Compound 18	1.9 µM	ND*	FRET assay	ND*	ND*	ND*	ND*	ND*	ND*	Hit	Structure-Based optimization of S2 binding	[51]
Compound 19	0.7 µM	ND*	FRET assay	ND*	ND*	ND*	ND*	ND*	ND*	Hit	Structure-Based optimization of S2 binding	[51]
Compound 20	1.2 µM	ND*	FRET assay	ND*	ND*	ND*	ND*	ND*	ND*	Hit	Structure-Based optimization of S2 binding	[51]
Compound 21	10 µM	ND*	FRET assay	ND*	ND*	ND*	ND*	ND*	ND*	Hit	Structure-Based optimization of S2 binding	[51]
Compound 23	1.9 µM	ND*	FRET assay	ND*	ND*	ND*	ND*	ND*	ND*	Hit	Structure-Based optimization of S2 binding	[51]
Compound 25	6.6 µM	ND*	FRET assay	ND*	ND*	ND*	ND*	ND*	ND*	Hit	Structure-	[51]

											Based optimiz ation of S2 binding	
Compou nd 26	1.5 µM	ND*	FRET assay	ND*	ND*	ND*	ND*	ND*	ND*	Hit	Structu re-Based optimiz ation of S2 binding	[51]
Compou nd 27	3.5 µM	ND*	FRET assay	ND*	ND*	ND*	ND*	ND*	ND*	Hit	Structu re-Based optimiz ation of S2 binding	[51]
Compou nd 28	0.9 µM	ND*	FRET assay	ND*	ND*	ND*	ND*	ND*	ND*	Hit	Structu re-Based optimiz ation of S2 binding	[51]
Compou nd 29	2.7 µM	ND*	FRET assay	ND*	ND*	ND*	ND*	ND*	ND*	Hit	Structu re-Based optimiz ation of S2 binding	[51]
Compou nd 33	7.6 µM	ND*	FRET assay	ND*	ND*	ND*	ND*	ND*	ND*	Hit	Structu re-Based optimiz ation of S2 binding	[51]
Compou nd 36	8.8 µM	ND*	FRET assay	ND*	ND*	ND*	ND*	ND*	ND*	Hit	Tri-Substit uted Diazep ane Analog ues	[51]
Compou nd 37	0.66 µM	ND*	FRET assay	ND*	ND*	ND*	ND*	ND*	ND*	Hit	Tri-Substit uted Diazep ane Analog ues	[51]
Compou nd 37a	0.33 µM	ND*	FRET assay	ND*	ND*	ND*	ND*	ND*	ND*	Hit	Tri-Substit uted Diazep ane Analog ues	[51]
Compou nd 38	0.28 µM	ND*	FRET assay	ND*	ND*	ND*	ND*	ND*	ND*	Hit	Tri-Substit uted Diazep ane Analog ues	[51]
Compou nd 38a	0.16 µM	ND*	FRET assay	ND*	ND*	ND*	ND*	ND*	ND*	Hit	Tri-Substit uted Diazep ane Analog ues	[51]

Compound 39	2.8 μM	ND*	FRET assay	ND*	ND*	ND*	ND*	ND*	ND*	Hit	Tri-Substituted Diazepane Analogues	[51]
Compound 40	1.9 μM	ND*	FRET assay	ND*	ND*	ND*	ND*	ND*	ND*	Hit	Tri-Substituted Diazepane Analogues	[51]
Compound 61	0.19 μM	ND*	FRET assay	ND*	ND*	ND*	ND*	ND*	ND*	Hit	Tri-Substituted Diazepane Analogues	[51]
Compound 62	0.21 μM	ND*	FRET assay	ND*	ND*	ND*	ND*	ND*	ND*	Hit	Tri-Substituted Diazepane Analogues	[51]
Compound 63	0.92 μM	ND*	FRET assay	ND*	ND*	ND*	ND*	ND*	ND*	Hit	Tri-Substituted Diazepane Analogues	[51]
Compound 64	0.27 μM	ND*	FRET assay	ND*	ND*	ND*	ND*	ND*	ND*	Hit	Tri-Substituted Diazepane Analogues	[51]
Compound 65	1.3 μM	ND*	FRET assay	ND*	ND*	ND*	ND*	ND*	ND*	Hit	Tri-Substituted Diazepane Analogues	[51]
Compound 66	0.63 μM	ND*	FRET assay	ND*	ND*	ND*	ND*	ND*	ND*	Hit	Tri-Substituted Diazepane Analogues	[51]
Compound 67	1.0 μM	ND*	FRET assay	ND*	ND*	ND*	ND*	ND*	ND*	Hit	Tri-Substituted Diazepane Analogues	[51]
Compound 68	0.25 μM	ND*	FRET assay	ND*	ND*	ND*	ND*	ND*	ND*	Hit	Tri-Substituted Diazepane Analogues	[51]
Compound 69	0.52 μM	ND*	FRET assay	ND*	ND*	ND*	ND*	ND*	ND*	Hit	Tri-Substituted Diazepane	[51]

											Analogues	
Compound 70	10 µM	ND*	FRET assay	ND*	ND*	ND*	ND*	ND*	ND*	Hit	Tri-Substituted Diazepane Analogues	[51]
Compound 71	0.62 µM	ND*	FRET assay	ND*	ND*	ND*	ND*	ND*	ND*	Hit	Tri-Substituted Diazepane Analogues	[51]
Compound 76	0.2 µM	ND*	FRET assay	ND*	ND*	ND*	ND*	ND*	ND*	Hit	Tri-Substituted Diazepane Analogues	[51]
Compound 77	5.6 µM	ND*	FRET assay	ND*	ND*	ND*	ND*	ND*	ND*	Hit	Tri-Substituted Diazepane Analogues	[51]
Compound 78	0.39 µM	ND*	FRET assay	ND*	ND*	ND*	ND*	ND*	ND*	Hit	Tri-Substituted Diazepane Analogues	[51]
Compound 79	4.7 µM	ND*	FRET assay	ND*	ND*	ND*	ND*	ND*	ND*	Hit	Tri-Substituted Diazepane Analogues	[51]
Compound 80	2.2 µM	ND*	FRET assay	ND*	ND*	ND*	ND*	ND*	ND*	Hit	Tri-Substituted Diazepane Analogues	[51]
Compound 81	1.9 µM	ND*	FRET assay	ND*	ND*	ND*	ND*	ND*	ND*	Hit	Tri-Substituted Diazepane Analogues	[51]
Compound 82	7.2 µM	ND*	FRET assay	ND*	ND*	ND*	ND*	ND*	ND*	Hit	Tri-Substituted Diazepane Analogues	[51]
Compound 85	8.1 µM	ND*	FRET assay	ND*	ND*	ND*	ND*	ND*	ND*	Hit	Tri-Substituted Diazepane Analogues	[51]
Compound 87	3.7 µM	ND*	FRET assay	ND*	ND*	ND*	ND*	ND*	ND*	Hit	Tri-Substituted	[51]

											Diazepane Analogues	
Compound 90	9.2 µM	ND*	FRET assay	ND*	ND*	ND*	ND*	ND*	ND*	Hit	Tri-Substituted Diazepane Analogues	[51]
Compound 91	8.9 µM	ND*	FRET assay	ND*	ND*	ND*	ND*	ND*	ND*	Hit	Tri-Substituted Diazepane Analogues	[51]
Compound 92	9.9 µM	ND*	FRET assay	ND*	ND*	ND*	ND*	ND*	ND*	Hit	Tri-Substituted Diazepane Analogues	[51]
Compound 94	7.2 µM	ND*	FRET assay	ND*	ND*	ND*	ND*	ND*	ND*	Hit	Tri-Substituted Diazepane Analogues	[51]
Compound 96	1.5 µM	ND*	FRET assay	ND*	ND*	ND*	ND*	ND*	ND*	Hit	Tri-Substituted Diazepane Analogues	[51]
Compound 97	9.2 µM	ND*	FRET assay	ND*	ND*	ND*	ND*	ND*	ND*	Hit	Tri-Substituted Diazepane Analogues	[51]
Compound 98	0.23 µM	ND*	FRET assay	ND*	ND*	ND*	ND*	ND*	ND*	Hit	Tri-Substituted Diazepane Analogues	[51]
Compound 99	0.26 µM	ND*	FRET assay	ND*	ND*	ND*	ND*	ND*	ND*	Hit	Tri-Substituted Diazepane Analogues	[51]
Compound 100	0.15 µM	ND*	FRET assay	ND*	ND*	ND*	ND*	ND*	ND*	Hit	Tri-Substituted Diazepane Analogues	[51]
Compound 101	0.54 µM	ND*	FRET assay	ND*	ND*	ND*	ND*	ND*	ND*	Hit	Tri-Substituted Diazepane Analogues	[51]

Compound 102	0.12 µM	ND*	FRET assay	ND*	ND*	ND*	ND*	ND*	ND*	Hit	Tri-Substituted Diazepane Analogues	[51]
Compound 103	0.31 µM	ND*	FRET assay	ND*	ND*	ND*	ND*	ND*	ND*	Hit	Tri-Substituted Diazepane Analogues	[51]
Compound 104	0.27 µM	ND*	FRET assay	ND*	ND*	ND*	ND*	ND*	ND*	Hit	Tri-Substituted Diazepane Analogues	[51]
Compound 105	0.04 µM	ND*	FRET assay	ND*	ND*	ND*	ND*	ND*	ND*	Hit	Tri-Substituted Diazepane Analogues	[51]
Compound 106	0.06 µM	ND*	FRET assay	ND*	ND*	ND*	ND*	ND*	ND*	Hit	Tri-Substituted Diazepane Analogues	[51]
Compound 107	5.1 µM	ND*	FRET assay	ND*	ND*	ND*	ND*	ND*	ND*	Hit	Tri-Substituted Diazepane Analogues	[51]
Compound 108	0.39 µM	ND*	FRET assay	ND*	ND*	ND*	ND*	ND*	ND*	Hit	Tri-Substituted Diazepane Analogues	[51]
Compound 109	0.36 µM	ND*	FRET assay	ND*	ND*	ND*	ND*	ND*	ND*	Hit	Tri-Substituted Diazepane Analogues	[51]
Compound 110	0.24 µM	ND*	FRET assay	ND*	ND*	ND*	ND*	ND*	ND*	Hit	Tri-Substituted Diazepane Analogues	[51]
Compound 111	2.3 µM	ND*	FRET assay	ND*	ND*	ND*	ND*	ND*	ND*	Hit	Tri-Substituted Diazepane Analogues	[51]
Compound 119	15.7 nM	ND*	FRET assay	ND*	ND*	ND*	2011	ND*	ND*	Experimental	Tri-Substituted Diazepane	[51]

											Analogues	
Compound 112	21.3 nM	ND*	FRET assay	ND*	ND*	ND*	1100	ND*	ND*	Hit	Tri-Substituted Diazepane Analogues	[51]
Compound 121	51.2 nM	ND*	FRET assay	ND*	ND*	ND*	451	ND*	ND*	Hit	Tri-Substituted Diazepane Analogues	[51]
Compound 123	74.4 nM	ND*	FRET assay	ND*	ND*	ND*	633	ND*	ND*	Hit	Tri-Substituted Diazepane Analogues	[51]
9a	138 ± 13 nM	ND*	FRET assay	ND*	ND*	ND*	ND*	ND*	ND*	Hit	Thioamide-linked spiropyrrolidine derivatives	[106]
9d	115 ± 6 nM	ND*	FRET assay	ND*	ND*	ND*	ND*	ND*	ND*	Hit	Thioamide-linked spiropyrrolidine derivatives	[106]
9f	681 ± 47 nM	ND*	FRET assay	ND*	ND*	ND*	ND*	ND*	ND*	Hit	Thioamide-linked spiropyrrolidine derivatives	[106]
9g	35 ± 1 nM	ND*	FRET assay	ND*	ND*	ND*	ND*	ND*	ND*	Experimental	Thioamide-linked spiropyrrolidine derivatives	[106]
SD-9bb	25 ± 1 nM	ND*	FRET assay	ND*	ND*	ND*	ND*	ND*	ND*	Lead compound	Thioamide-linked spiropyrrolidine derivatives	[106]
Nirmatrelvir	54 ± 3 nM	ND*	FRET assay	ND*	ND*	ND*	ND*	ND*	ND*	Control	FDA approved	[106]
1	8.6 µM	ND*	Enzymatic assay	ND*	ND*	ND*	ND*	7.3 mL/min/kg	ND*	Hit	HTP screening	https://doi.org/10.1021/acs.jmedchem.2c00117

2	0.096 µM	ND*	Enzymatic assay	12.5 µM	Vero E6	ND*	ND*	4.9 mL/min/kg	ND*	Hit	Structure-based optimization	
3 (S-217622)	0.013 µM	ND*	Enzymatic assay	0.37 µM	Vero E6	ND*	ND*	1.7 mL/min/kg	ND*	Phase 3 (NCT0530547)	Structure-based optimization	
1	143 nM	ND*	Enzymatic assay	264 nM	HEK 293T	ND*	ND*	ND*	ND*	Hit	Reversible covalent warhead	[107]
2	335 nM	ND*	Enzymatic assay	1490 nM	HEK 293T	ND*	ND*	ND*	ND*	Hit	Reversible covalent warhead	[107]
3	179 nM	ND*	Enzymatic assay	191 nM	HEK 293T	ND*	ND*	ND*	ND*	Hit	Reversible covalent warhead	[107]
4	0.760 nM	ND*	Enzymatic assay	4.94 nM	HEK 293T	ND*	ND*	ND*	ND*	Hit	Reversible covalent warhead	[107]
5	11.8 nM	ND*	Enzymatic assay	20.4 nM	HEK 293T	ND*	ND*	ND*	ND*	Hit	Reversible covalent warhead	[107]
6	1.51 nM	ND*	Enzymatic assay	9.41 nM	HEK 293T	ND*	ND*	ND*	ND*	Hit	Reversible covalent warhead	[107]
7	14.0 nM	ND*	Enzymatic assay	41.9 nM	HEK 293T	ND*	ND*	ND*	ND*	Hit	Reversible covalent warhead	[107]
8	5.63 nM	ND*	Enzymatic assay	14.4 nM	HEK 293T	ND*	ND*	ND*	ND*	Hit	Reversible covalent warhead	[107]
9	8.37 nM	ND*	Enzymatic assay	18.4 nM	HEK 293T	ND*	ND*	ND*	ND*	Hit	Reversible covalent warhead	[107]
10	8.27 nM	ND*	Enzymatic assay	25.3 nM	HEK 293T	ND*	ND*	ND*	ND*	Hit	Reversible covalent warhead	[107]
11	19.6 nM	ND*	Enzymatic assay	41.8 nM	HEK 293T	ND*	ND*	ND*	ND*	Hit	Reversible covalent nt	[107]

											warhead	
12	99.6 nM	ND*	Enzymatic assay	117 nM	HEK 293T	ND*	ND*	ND*	ND*	Hit	Reversible covalent warhead	[107]
13	7.16 nM	ND*	Enzymatic assay	23.1 nM	HEK 293T	ND*	ND*	ND*	ND*	Hit	Reversible covalent warhead	[107]
14	0.403 nM	ND*	Enzymatic assay	1.57 nM	HEK 293T	ND*	ND*	ND*	ND*	Hit	Reversible covalent warhead	[107]
15	0.493 nM	ND*	Enzymatic assay	4.48 nM	HEK 293T	ND*	ND*	ND*	ND*	Hit	Reversible covalent warhead	[107]
16	2.36 nM	ND*	Enzymatic assay	7.79 nM	HEK 293T	ND*	ND*	ND*	ND*	Hit	Reversible covalent warhead	[107]
17 (S-892216)	0.655 nM	ND*	Enzymatic assay	2.48 nM	HEK 293T	ND*	ND*	6.79/2.52/11.4 mL/min/kg	ND*	Experimental	Reversible covalent warhead	[107]
18	0.391 nM	ND*	Enzymatic assay	3.82 nM	HEK 293T	ND*	ND*	6.69/4.59/19.7 mL/min/kg	ND*	Hit	Reversible covalent warhead	[107]
AC1115	2.44 nM	ND*		0.96 µM		ND*	ND*	ND*	ND*			[108]
Nirmatrelvir	5.38 nM	ND*		2.23 µM		ND*	ND*	41.2 mL/mL/kg	ND*	FDA-approved		[108]
Olgotrelvir	ND*	ND*		1.38 µM		ND*	ND*	11.0 mL/min/kg	ND*	Experimental		[108]
Nirmatrelvir	0.031 ± 0.001 µM	ND*	FRET assay	ND*	ND*	ND*	ND*	ND*	ND*	Lead compound	Varying nirmatrelvir warheads	[109]
2	0.010 ± 0.001 µM	ND*	FRET assay	ND*	ND*	ND*	ND*	ND*	ND*	Hit	Varying nirmatrelvir warheads	[109]
5	0.008 ± 0.001 µM	ND*	FRET assay	ND*	ND*	ND*	ND*	ND*	ND*	Experimental	Varying nirmatrelvir warheads	[109]
7	0.044 ± 0.	ND*	FRET assay	ND*	ND*	ND*	ND*	ND*	ND*	Hit	Varying nirmatrelvir	[109]

	002 μM										warheads	
8	0.019 ± 0.001 μM	ND*	FRET assay	ND*	ND*	ND*	ND*	ND*	ND*	Hit	Varying nirmatrelvir warheads	[109]
9	0.027 ± 0.001 μM	ND*	FRET assay	ND*	ND*	ND*	ND*	ND*	ND*	Hit	Varying nirmatrelvir warheads	[109]
10	0.027 ± 0.001 μM	ND*	FRET assay	ND*	ND*	ND*	ND*	ND*	ND*	Hit	Varying nirmatrelvir warheads	[109]
4 (GC-3760	190 ± 40 nM	ND*	FRET assay	0.9 ± 0.2 μM	Vero E6	>200 μM	ND*	ND*	ND*	Control	Aldehyde prodrug	[110]
7	60 ± 10 nM	ND*	FRET assay	0.9 ± 0.1 μM	Vero E6	>200 μM	ND*	ND*	ND*	Experimental	Aldehyde and bisulfate derivatives	[110]
17a	40 ± 8 nM	ND*	FRET assay	>5 μM	Vero E6	>200 μM	ND*	ND*	ND*	Hit	Nitrile warhead	[110]
18a	13 ± 3 nM	ND*	FRET assay	2.7 ± 0.7	Vero E6	>200 μM	ND*	ND*	ND*	Hit	Nitrile warhead	[110]
18b	9.1 ± 2.5 nM	ND*	FRET assay	2.2 ± 0.5 μM	Vero E6	>200 μM	ND*	ND*	ND*	Hit	Nitrile warhead	[110]
19b	14 ± 2 nM	ND*	FRET assay	1.5 ± 0.4 μM	Vero E6	>200 μM	ND*	ND*	ND*	Hit	Nitrile warhead	[110]
18c	24 ± 4 nM	ND*	FRET assay	4.8 ± 0.5 μM	Vero E6	>200 μM	ND*	ND*	ND*	Hit	Nitrile warhead	[110]
18d	13.7 ± 1.5 nM	ND*	FRET assay	2.6 ± 0.3 μM	Vero E6	>100 μM	ND*	ND*	ND*	Hit	Nitrile warhead	[110]
18e	49 ± 10 nM	ND*	FRET assay	>10 μM	Vero E6	>200 μM	ND*	ND*	ND*	Hit	Nitrile warhead	[110]
GC376	35 nM	ND*	qRT-PCR assay	51 nM	Huh-7ACE2	ND*	ND*	ND*	ND*	Control		[111]
EB46	23 nM	ND*	qRT-PCR assay	14 nM	Huh-7ACE3	>100 μM	ND*	ND*	ND*	Hit	GC376 analogs	[111]
EB48	200 nM	ND*	qRT-PCR assay	16 μM	Vero E6	ND*	ND*	ND*	ND*	Hit	GC376 analogs	[111]
EB54	29 nM	ND*	qRT-PCR assay	5 nM	Huh-7ACE2	>100 μM	ND*	ND*	ND*	Hit	GC376 analogs	[111]
EB55	72 nM	ND*	qRT-PCR assay	11 nM	Huh-7ACE2	ND*	ND*	ND*	ND*	Hit	GC376 analogs	[111]
EB56	50 nM	ND*	qRT-PCR assay	28 nM	Huh-7ACE2	ND*	ND*	ND*	ND*	Hit	GC376 analogs	[111]
SL-4-241	75 nM	ND*	qRT-PCR assay	17 μM	Vero E6	ND*	ND*	ND*	ND*	Hit	GC376 analogs	[111]

NK01-14	22 nM	ND*	qRT-PCR assay	26 nM	Huh-7ACE2	ND*	ND*	ND*	ND*	Hit	GC376 analogs	[111]
NK01-48	92 nM	ND*	qRT-PCR assay	15 nM	Huh-7ACE2	ND*	ND*	ND*	ND*	Hit	GC376 analogs	[111]
NK01-57	13 nM	ND*	qRT-PCR assay	92 nM	Huh-7ACE2	ND*	ND*	ND*	ND*	Hit	GC376 analogs	[111]
NK01-63	16 nM	ND*	qRT-PCR assay	6 nM	Huh-7ACE2	>100 µM	ND*	ND*	ND*	Experimental	GC376 analogs	[111]
NK01-69	24 nM	ND*	qRT-PCR assay	35 nM	Huh-7ACE2	ND*	ND*	ND*	ND*	Hit	GC376 analogs	[111]
GC376	31 ± 4 nM	ND*	fluorescent peptide assay	ND*	ND*		ND*	ND*	ND*	Control	Opal-based dipeptidyl and tripeptidyl inhibitors	[112]
MPI1	100 ± 23 nM	ND*	fluorescent peptide assay	ND*	ND*		ND*	ND*	ND*	Hit	Opal-based dipeptidyl and tripeptidyl inhibitors	[112]
MPI2	103 ± 14 nM	ND*	fluorescent peptide assay	ND*	ND*		ND*	ND*	ND*	Hit	Opal-based dipeptidyl and tripeptidyl inhibitors	[112]
MPI3	8.5 ± 1.5 nM	ND*	fluorescent peptide assay	ND*	Vero E6		ND*	ND*	ND*	Hit	Opal-based dipeptidyl and tripeptidyl inhibitors	[112]
MPI4	15.5 ± 5 nM	ND*	fluorescent peptide assay	ND*	ND*		ND*	ND*	ND*	Hit	Opal-based dipeptidyl and tripeptidyl inhibitors	[112]
MPI5	33 ± 2 nM	ND*	fluorescent peptide assay	ND*	Vero E6		ND*	ND*	ND*	Hit	Opal-based dipeptidyl and tripeptidyl inhibitors	[112]
MPI6	60 ± 4 nM	ND*	fluorescent peptide assay	ND*	ND*		ND*	ND*	ND*	Hit	Opal-based dipeptidyl and tripeptidyl inhibitors	[112]
MPI7	47 ± 3 nM	ND*	fluorescent peptide assay	ND*	ND*		ND*	ND*	ND*	Hit	Opal-based dipeptidyl and	[112]

											tripeptidyl inhibitors	
MPI8	105 ± 22 nM	ND*	fluorescent peptide assay	ND*	Vero E6		ND*	ND*	ND*	Hit	Opal-based dipeptidyl and tripeptidyl inhibitors	[112]
MPI9	56 ± 14 nM	ND*	fluorescent peptide assay	ND*	ND*		ND*	ND*	ND*	Hit	Opal-based dipeptidyl and tripeptidyl inhibitors	[112]
11a	31 ± 3 nM	ND*	fluorescent peptide assay	ND*	ND*	ND*	ND*	ND*	ND*	Experimental	Opal-based dipeptidyl and tripeptidyl inhibitors	[112]

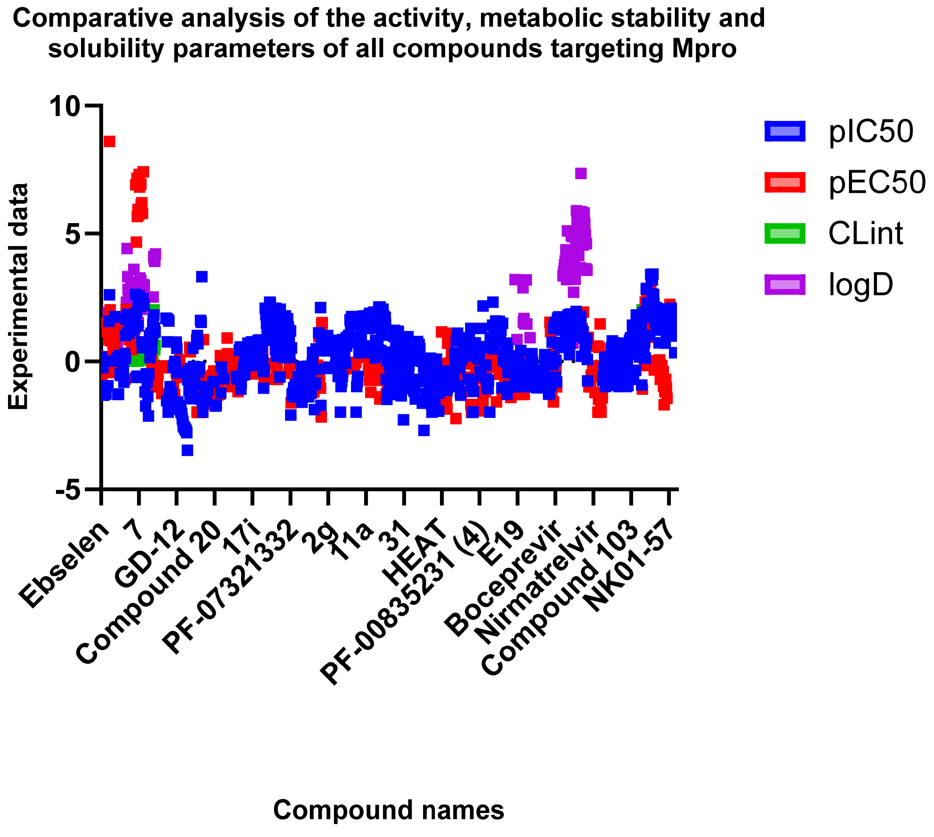

Figure S1. Comparative analysis of the activity, metabolic stability and solubulity parameters of all reported compounds targeting Mpro.

Table S5. Pharmacological and physicochemical profiles of inhibitors targeting SARS-CoV-2 PLpro

No.	Name	IC_{50}	K_i	Type of PLpro assay	EC_{50}	Type of viral assay	CC_{50}	Metabolic profiling (CL_{int}) (μl/min/mg)/μL/min/10 cells	Metabolite ID	Log D	Approval status	Design strategy	References
1	7724772	23.5 μM	ND*	SARS-CoV-2 PLpro Inhibition assay	ND*	ND*	ND*	ND*	ND*	ND*	Hit	Original HTS hit containing 2-naphtalene moiety	[113]
2	GRL-0617	2.4 ± 0.2 μM	ND*	Enzymatic assay	27.6 ± 1.2 μM	Vero E6	>60 μM	ND*	ND*	ND*	Lead	7724772 derivative with 1-naphthyl and amino-methylbenzene groups	[113]
2a	GRL-0617	2.1 ± 0.2 μM	ND*	Enzymatic assays	21.2 μM	Vero E6 cell	ND*	ND*	ND*	ND*	Lead	7724772 derivative with 1-naphthyl and amino-methylbenzene groups	[114]
2b	GRL0617	ND*	1800 nM	Enzymatic assay	68200 nM	Vero E6	ND*	20.9	ND*	ND*	Lead	7724772 derivative with 1-naphthyl and amino-methylbenzene groups	[115]
2c	GRL-0617	ND*	ND*	ND*	35.43 ± 15.02 μM	Vero E6	48.28± 20. 74 μM	ND*	ND*	ND*	Lead	7724772 derivative with 1-naphthyl and amino-methylbenzene groups	[116]
2d	GRL-0617	1.66 ± 0.36 μM	ND*	Enzymatic assay	33.9 ± 6.5 μM	Calu-3 cells	>40 μM	ND*	ND*	ND*	Lead	7724772 derivative with 1-naphthyl and amino-methylbenzene groups	[117]
2e	GRL-0617	1.39 ± 0.26 μM	ND*	High-throughput screening	3.18 ± 0.71	Vero E6	~500 μM	ND*	ND*	ND*	Lead	7724772 derivative with 1-naphthyl and amino-methylbenzene groups	[118]
2f	GRL-0617	0.82 μM	ND*	Enzymatic assay	ND*	ND*	ND*	ND*	ND*	ND*	Lead	7724772 derivative with 1-naphthyl and amino-methylbenzene groups	[119]
2g	GRL-0617	6.97 ± 1.34 μM	ND*	Enzymatic assay	ND*	ND*	ND*	ND*	ND*	ND*	Lead	7724772 derivative with 1-naphthyl and amino-methylbenzene groups	[120]

2h	GRL-0617	1.60 ± 0.13	ND*	FRET-based assay	23.64 ± 4.72 μM	Vero E6	>60 μM	ND*	ND*	ND*	Lead	7724772 derivative with 1-naphthyl and amino-methylbenzene groups	[121]
2i	GRL-0617	1.2 μM	ND*	Fluorogenic peptide assay	ND*	ND*	ND*	ND*	ND*	ND*	Lead	7724772 derivative with 1-naphthyl and amino-methylbenzene groups	[122]
2j	GRL-0167	1.61 μM	ND*	Enzymatic assays	>20 μM	ND*	>80 μM	13.13	ND*	ND*	Lead	7724772 derivative with 1-naphthyl and amino-methylbenzene groups	[123]
2k	GRL-0617	1.113 ± 0.175 ng/μL	ND*	Enzymatic assays	ND*	ND*	ND*	ND*	ND*	ND*	Lead	7724772 derivative with 1-naphthyl and amino-methylbenzene groups	[124]
2l	GRL-0617	0.65 μM	ND*	High-throughput screening	ND*	ND*	ND*	ND*	ND*	ND*	Lead	7724772 derivative with 1-naphthyl and amino-methylbenzene groups	[125]
2m	GRL-0617	ND*	ND*	ND*	25.47 μM	Caco-2	ND*	67.4	ND*	ND*	Lead	7724772 derivative with 1-naphthyl and amino-methylbenzene groups	[126]
2n	GRL-0617	1.43 ± 0.26 μM	ND*	Enzymatic assay	15.68 ± 1.97 μM	Cell-based assay (FlipGFP)	>40 μM	ND*	ND*	ND*	Lead	7724772 derivative with 1-naphthyl and amino-methylbenzene groups	[127]
2o	GRL-0617	1.88 μM	ND*	FRET assay	74.42 μM	ND*	ND*	ND*	ND*	ND*	Lead	7724772 derivative with 1-naphthyl and amino-methylbenzene groups	[128]
2p	GRL-0617	2.4 ± 1.2 μM	ND*	Enzymatic assay	17.6 ± 1.2 μM	Vero-E6	ND*	ND*	ND*	ND*	Lead	7724772 derivative with 1-naphthyl and amino-methylbenzene groups	[113]
2q	GRL-0617	ND*	ND*	ND*	19.56 ± 1.75 μM	FlipGFP assay/CCk8 assay	>200 μM	ND*	ND*	ND*	Lead	7724772 derivative with 1-naphthyl and amino-methylbenzene groups	[129]
2r	GRL-0617	4.82 μM	ND*	Enzymatic assay	ND*	ND*	ND*	ND*	ND*	ND*	Lead	7724772 derivative with 1-naphthyl	[130]

												and amino-methylbenzene groups	
2s	GRL-0617	2.12 µM	ND*	Enzymatic activity	ND*	ND*	ND*	ND*	ND*	ND*	Lead	7724772 derivative with 1-naphthyl and amino-methylbenzene groups	[131]
2t	GRL-0617	1918.0 ±150.0 nM	1374 ± 58 nM	FRET enzymatic assay	>20 µM	Vero E6	107.3 ±10.9 µM	ND*	ND*	ND*	Lead	7724772 derivative with 1-naphthyl and amino-methylbenzene groups	[132]
3	2	ND*	116 nM	Enzymatic assay	1850 nM	Vero E6	ND*	13.9	CYP 3A4	ND*	hit	N-methyl pyrazole derivative	[115]
4	3	ND*	9 nM	Enzymatic assay	652 nM	Vero E6	ND*	12.8	CYP 3A5	ND*	hit	N-methyl pyrazole derivative	[115]
5	4(PF-07957472)	28 µM	2 nM	Enzymatic assay	147 nM	Vero E6	69.3 µM	8.84	CYP 3A4	2.5	Experimental	N-methyl pyrazole derivative	[115]
6	JUN12129	90.9±4.6 nM	75.5± 2.0 nM	FRET enzymatic assay	ND*	Vero E6	8.3 ± 1.2 µM	ND*	CYP 450	ND*	Hit	Thiophene-containing	[132]
7	JUN11875	66.2 ±2 nM	13.2 ±2 nM	FRET enzymatic assay	ND*	Vero E6	4.5 ± 1.6 µM	ND*	CYP 450	ND*	Hit	Thiophene-containing	[132]
8	JUN12303	121.5 ± 4.0 nM	88.2 ± 6.0 nM	FRET enzymatic assay	0.32 µM	Vero E6	56.8 ± 7.4 µM	ND*	CYP 450	ND*	Hit	Pyrazole-containing	[132]
9	JUN12162	98.3 ± 7.0 nM	33.6 ± 3.0 nM	FRET enzymatic assay	0.23 µM	Vero E6	42.3 ± 2.3 µM	ND*	CYP 450	ND*	Hit	Pyrazole-containing	[132]
10	JUN12682	106.8±5.0 nM	37.7 nM	FRET enzymatic assay	0.42 µM	Vero E6	61.3 ± 4.5 µM	10.5	CYP 450	ND*	Experimental	Pyrazole-containing	[132]
11	JUN11941	151.4 ± 4.0 nM	34.3 ± 3.0 nM	FRET enzymatic assay	0.31 µM	Vero E6	54.8 ± 8.3 µM	ND*	CYP 450	ND*	Hit	Pyrazole-containing	[132]
12	JUN12199	108.8 ± 10.2 nM	47.6 ± 3.0 nM	FRET enzymatic assay	0.45 µM	Vero E6	63.2 ± 11.3 µM	ND*	CYP 450	ND*	Hit	Pyrazole-containing	[132]
13	JUN12197	102.7 ± 10.2 nM	33.2 ± 3.0 nM	FRET enzymatic assay	0.35 µM	Vero E6	43.7 ± 5.1 µM	ND*	CYP 450	ND*	Hit	Pyrazole-containing	[132]
14	JUN12603	112.2 ± 6.0 nM	39.8 ± 4.0 nM	FRET enzymatic assay	0.50 µM	Vero E6	61.0 ± 19.3 µM	ND*	CYP 450	ND*	Hit	Pyrazole-containing	[132]
15	JUN12351	98.8 ± 5.2 nM	29.3 ± 3.0	FRET enzym	0.59 µM	Vero E6	>125 µM	ND*	CYP 450	ND*	Hit	Pyrazole-containing	[132]

			n M	atic assay									
16	JUN12145	108.5 ± 6.2 nM	35.2 ± 2.0 nM	FRET enzymatic assay	0.58 μM	Vero E6	31.3 ± 2.0 μM	ND*	CYP 450	ND*	Hit	Pyrazole-containing	[132]
17	JUN12682	106.8 ± 5.0 nM	37.7 ± 3.0 nM	FRET enzymatic assay	0.42 μM	Vero E6	61.3 ± 4.5 μM	ND*	CYP 450	ND*	Hit	Pyrazole-containing	[132]
18	JUN12763	164.6 ± 23.0 nM	37.2 ± 4.0 nM	FRET enzymatic assay	0.96 μM	Vero E6	>125 μM	ND*	CYP 450	ND*	Hit	Pyrazole-containing	[132]
19	JUN12395	121.9 ± 8.0 nM	47.1 ± 4.0 nM	FRET enzymatic assay	1.15 μM	Vero E6	109.4 ± 16.2 μM	ND*	CYP 450	ND*	Hit	Pyrazole-containing	[132]
20	JUN12713	86.7 ± 6.0 nM	34.0 ± 3.0 nM	FRET enzymatic assay	0.86 μM	Vero E6	107.2 ± 6.2 μM	ND*	CYP 450	ND*	Hit	Pyrazole-containing	[132]
21	JUN12602	116.2 ± 9.2 nM	46.9 ± 3.0 nM	FRET enzymatic assay	0.80 μM	Vero E6	>125 μM	ND*	CYP 450	ND*	Hit	Pyrazole-containing	[132]
22	2 (XR8-24)	0.58 μM	ND*	Enzymatic assay	ND*	ND*	ND*	ND*	ND*	ND*	Lead	Aminoazetidine-substituted phenylthiophenes	[133]
23	10 (XR8-23)	0.39 μM	ND*	Enzymatic assay	2.5 ± 0.15 μM	A549-hACE2	43 ± 1.8 μM	ND*	ND*	ND*	Experimental	Aminoazetidine-substituted phenylthiophenes	[133]
24	11 (XR8-89)	0.11 μM	ND*	Enzymatic assay	ND*	ND*	ND*	ND*	ND*	ND*	Hit	Aminoazetidine-substituted phenylthiophenes	[133]
25	12 (XR8-65)	0.33 μM	ND*	Enzymatic assay	ND*	ND*	ND*	ND*	ND*	ND*	Hit	Aminoazetidine-substituted phenylthiophenes	[133]
26	13 (XR8-69)	0.37 μM	ND*	Enzymatic assay	ND*	ND*	ND*	ND*	ND*	ND*	Hit	Aminoazetidine-substituted phenylthiophenes	[133]
27	14 (XR8-83)	0.21 μM	ND*	Enzymatic assay	ND*	ND*	ND*	ND*	ND*	ND*	Hit	Aminoazetidine-substituted phenylthiophenes	[133]
28	15	0.92 μM	ND*	Enzymatic assay	ND*	ND*	ND*	ND*	ND*	ND*	Hit	Aminoazetidine-substituted phenylthiophenes	[133]
29	16	0.65 μM	ND*	Enzymatic assay	2.3 ± 0.22 μM	A549-Hace2	27 ± 1.4 μM	ND*	ND*	ND*	Hit	Aminoazetidine-substituted phenylthiophenes	[133]

30	17	0.33 µM	ND*	Enzymatic assay	ND*	ND*	ND*	ND*	ND*	ND*	Hit	Aminoazetidine-substituted phenylthiophenes	[133]
31	18	1.48 µM	ND*	Enzymatic assay	ND*	ND*	ND*	ND*	ND*	ND*	Hit	Aminoazetidine-substituted phenylthiophenes	[133]
32	19	1.96 µM	ND*	Enzymatic assay	ND*	ND*	ND*	ND*	ND*	ND*	Hit	Aminoazetidine-substituted phenylthiophenes	[133]
33	20	3.17 µM	ND*	Enzymatic assay	ND*	ND*	ND*	ND*	ND*	ND*	Hit	Aminoazetidine-substituted phenylthiophenes	[133]
34	21	1.56 µM	ND*	Enzymatic assay	ND*	ND*	ND*	ND*	ND*	ND*	Hit	Aminoazetidine-substituted phenylthiophenes	[133]
35	22	1.13 µM	ND*	Enzymatic assay	ND*	ND*	ND*	ND*	ND*	ND*	Hit	Aminoazetidine-substituted phenylthiophenes	[133]
36	23	5.53 µM	ND*	Enzymatic assay	ND*	ND*	ND*	ND*	ND*	ND*	Hit	Aminoazetidine-substituted phenylthiophenes	[133]
37	24	1.93 µM	ND*	Enzymatic assay	ND*	ND*	ND*	ND*	ND*	ND*	Hit	Aminoazetidine-substituted phenylthiophenes	[133]
38	25	1.19 µM	ND*	Enzymatic assay	ND*	ND*	ND*	ND*	ND*	ND*	Hit	Aminoazetidine-substituted phenylthiophenes	[133]
39	26	4.75 µM	ND*	Enzymatic assay	ND*	ND*	ND*	ND*	ND*	ND*	Hit	Aminoazetidine-substituted phenylthiophenes	[133]
40	27	4.43 µM	ND*	Enzymatic assay	ND*	ND*	ND*	ND*	ND*	ND*	Hit	Aminoazetidine-substituted phenylthiophenes	[133]
41	28	6.12 µM	ND*	Enzymatic assay	ND*	ND*	ND*	ND*	ND*	ND*	Hit	Aminoazetidine-substituted phenylthiophenes	[133]
42	29	1.08 µM	ND*	Enzymatic assay	ND*	ND*	ND*	ND*	ND*	ND*	Hit	Aminoazetidine-substituted phenylthiophenes	[133]
43	30	0.82 µM	ND*	Enzymatic assay	ND*	ND*	ND*	ND*	ND*	ND*	Hit	Aminoazetidine-substituted phenylthiophenes	[133]
44	31	0.79 µM	ND*	Enzymatic assay	ND*	ND*	ND*	ND*	ND*	ND*	Hit	Aminoazetidine-substituted	[133]

												phenylthiophenes	
45	32	0.38 µM	ND*	Enzymatic assay	ND*	ND*	ND*	ND*	ND*	ND*	Hit	Aminoazetidine-substituted phenylthiophenes	[133]
46	33	0.82 µM	ND*	Enzymatic assay	ND*	ND*	ND*	ND*	ND*	ND*	Hit	Aminoazetidine-substituted phenylthiophenes	[133]
47	34	0.69 µM	ND*	Enzymatic assay	ND*	ND*	ND*	ND*	ND*	ND*	Hit	Aminoazetidine-substituted phenylthiophenes	[133]
48	35	2.40 µM	ND*	Enzymatic assay	ND*	ND*	ND*	ND*	ND*	ND*	Hit	Aminoazetidine-substituted phenylthiophenes	[133]
49	36	1.14 µM	ND*	Enzymatic assay	ND*	ND*	ND*	ND*	ND*	ND*	Hit	Aminoazetidine-substituted phenylthiophenes	[133]
50	37	0.96 µM	ND*	Enzymatic assay	ND*	ND*	ND*	ND*	ND*	ND*	Hit	Aminoazetidine-substituted phenylthiophenes	[133]
51	38	0.55 µM	ND*	Enzymatic assay	ND*	ND*	ND*	ND*	ND*	ND*	Hit	Aminoazetidine-substituted phenylthiophenes	[133]
52	39	0.32 µM	ND*	Enzymatic assay	ND*	ND*	ND*	ND*	ND*	ND*	Hit	Aminoazetidine-substituted phenylthiophenes	[133]
53	40	0.49 µM	ND*	Enzymatic assay	ND*	ND*	ND*	ND*	ND*	ND*	Hit	Aminoazetidine-substituted phenylthiophenes	[133]
54	41	0.96 µM	ND*	Enzymatic assay	ND*	ND*	ND*	ND*	ND*	ND*	Hit	Aminoazetidine-substituted phenylthiophenes	[133]
55	42	0.81 µM	ND*	Enzymatic assay	ND*	ND*	ND*	ND*	ND*	ND*	Hit	Aminoazetidine-substituted phenylthiophenes	[133]
56	43	0.43 µM	ND*	Enzymatic assay	ND*	ND*	ND*	ND*	ND*	ND*	Hit	Aminoazetidine-substituted phenylthiophenes	[133]
57	44	0.75 µM	ND*	Enzymatic assay	787 ± 68 nM	A549-Hace2	22 ± 0.9 µM	ND*	ND*	ND*	Hit	Aminoazetidine-substituted phenylthiophenes	[133]
58	45	1.6 µM	ND*	Enzymatic assay	ND*	ND*	ND*	ND*	ND*	ND*	Hit	Aminoazetidine-substituted phenylthiophenes	[133]

59	46	0.36 µM	ND*	Enzymatic assay	ND*	ND*	ND*	ND*	ND*	ND*	Hit	Aminoazetidine-substituted phenylthiophenes	[133]
60	47	0.26 µM	ND*	Enzymatic assay	ND*	ND*	ND*	ND*	ND*	ND*	Hit	Aminoazetidine-substituted phenylthiophenes	[133]
61	48	0.57 µM	ND*	Enzymatic assay	ND*	ND*	ND*	ND*	ND*	ND*	Hit	Aminoazetidine-substituted phenylthiophenes	[133]
62	49	1.2 µM	ND*	Enzymatic assay	ND*	ND*	ND*	ND*	ND*	ND*	Hit	Aminoazetidine-substituted phenylthiophenes	[133]
63	50	1.79 µM	ND*	Enzymatic assay	ND*	ND*	ND*	ND*	ND*	ND*	Hit	Aminoazetidine-substituted phenylthiophenes	[133]
64	51	2.42 µM	ND*	Enzymatic assay	ND*	ND*	ND*	ND*	ND*	ND*	Hit	Aminoazetidine-substituted phenylthiophenes	[133]
65	52	1.52 µM	ND*	Enzymatic assay	ND*	ND*	ND*	ND*	ND*	ND*	Hit	Aminoazetidine-substituted phenylthiophenes	[133]
66	53	1.39 µM	ND*	Enzymatic assay	ND*	ND*	ND*	ND*	ND*	ND*	Hit	Aminoazetidine-substituted phenylthiophenes	[133]
67	54	1.69 µM	ND*	Enzymatic assay	ND*	ND*	ND*	ND*	ND*	ND*	Hit	Aminoazetidine-substituted phenylthiophenes	[133]
68	55	0.60 µM	ND*	Enzymatic assay	ND*	ND*	ND*	ND*	ND*	ND*	Hit	Aminoazetidine-substituted phenylthiophenes	[133]
69	56	2.82 µM	ND*	Enzymatic assay	ND*	ND*	ND*	ND*	ND*	ND*	Hit	Aminoazetidine-substituted phenylthiophenes	[133]
70	57	1.39 µM	ND*	Enzymatic assay	ND*	ND*	ND*	ND*	ND*	ND*	Hit	Aminoazetidine-substituted phenylthiophenes	[133]
71	58	1.56 µM	ND*	Enzymatic assay	ND*	ND*	ND*	ND*	ND*	ND*	Hit	Aminoazetidine-substituted phenylthiophenes	[133]
72	59	9.78 µM	ND*	Enzymatic assay	ND*	ND*	ND*	ND*	ND*	ND*	Hit	Aminoazetidine-substituted phenylthiophenes	[133]
73	60	0.56 µM	ND*	Enzymatic assay	ND*	ND*	ND*	ND*	ND*	ND*	Hit	Aminoazetidine-substituted	[133]

												phenylthiophenes	
74	61	0.50 µM	ND*	Enzymatic assay	ND*	ND*	ND*	ND*	ND*	ND*	Hit	Aminoazetidine-substituted phenylthiophenes	[133]
75	62	0.60 µM	ND*	Enzymatic assay	ND*	ND*	ND*	ND*	ND*	ND*	Hit	Aminoazetidine-substituted phenylthiophenes	[133]
76	63	0.47 µM	ND*	Enzymatic assay	ND*	ND*	ND*	ND*	ND*	ND*	Hit	Aminoazetidine-substituted phenylthiophenes	[133]
77	64	0.62 µM	ND*	Enzymatic assay	ND*	ND*	ND*	ND*	ND*	ND*	Hit	Aminoazetidine-substituted phenylthiophenes	[133]
78	F0213	7.4 µM	ND*	HTS fluorescence-based Plpro Cleavage assay	4.549 µM	Vero E6-TMPRSS2 cells	ND*	ND*	ND*	ND*	Experimental	HTS lead	[134]
79	F0326	8.2 µM	ND*	HTS fluorescence-based Plpro Cleavage assay	7.62 µM	Vero E6-TMPRSS2 cells	ND*	ND*	ND*	ND*	Hit	HTS hit	[134]
80	F0393	ND*	ND*	HTS fluorescence-based Plpro Cleavage assay	15.59 µM	Vero E6-TMPRSS2 cells	ND*	ND*	ND*	ND*	Hit	HTS hit	[134]
81	Compound 6	5 ± 1.9 µM	ND*	Enzymatic assay	21 ± 1.9 µM	Vero E6	>60 µM	ND*	ND*	ND*	Experimental	Lead containing 1-naphthyl and acetyl amino moieties	[113]
82	7724772	23.5 ± 1.7 µM	ND*	Enzymatic assay	ND*	ND*	ND*	ND*	ND*	ND*	Hit	Hit containing 1-naphthyl and acetyl amino moieties	[113]
83	6577871	100.7 ± 4.76 µM	ND*	Enzymatic assay	ND*	ND*	ND*	ND*	ND*	ND*	Hit	Insertion of a 4-piperidinecarboxa-mide oxalate moiety	[113]
84	9247873	>200 µM	ND*	Enzymatic assay	ND*	ND*	ND*	ND*	ND*	ND*	Hit	Insertion of a 4-piperidinecarboxa-mide oxalate moiety	[113]

88	1c	2.64 ± 0.34 µM	ND*	HEK293T	ND*	ND*	ND*	ND*	ND*	ND*	Lead	peptide–drug conjugate	[135]
89	Ac-EM-C	7.4 ± 0.37 µM	ND*	HEK293T	ND*	ND*	ND*	ND*	ND*	ND*	Experimental	peptide–drug conjugate	[135]
90	Ac-ELRGG	15.38 ± 1.63 µM	ND*	HEK293T	ND*	ND*	ND*	ND*	ND*	ND*	Hit	peptide–drug conjugate	[135]
91	Ac-EC-M	8.63 ± 0.55 µM	ND*	HEK293T	ND*	ND*	ND*	ND*	ND*	ND*	Experimental	peptide–drug conjugate	[135]
92	Cryptotanshinone	5.63 ± 1.45 µM	ND*	HTS fluorescence-based enzymatic assay	0.70 ± 0.09 µM	Vero E6	>300 µM	ND*	ND*	ND*	Hit	HTS of a library containing diverse compounds	[135]
93	Tanshinone I	2.1 ± 0.1 µM	ND*	HTS fluorescence-based enzymatic assay	2.26 ± 0.11 µM	Vero E6	>300 µM	ND*	ND*	ND*	Hit	HTS of a library containing diverse compounds	[135]
94	YM155	2.47 ± 0.46 µM	ND*	HTS fluorescence-based enzymatic assay	0.17 ± 0.22 µM	Vero E6	~400 µM	ND*	ND*	ND*	Experimental	HTS of a library containing diverse compounds	[135]
95	Rac5c	0.81 µM	ND*	HTS fluorescence-based enzymatic assay	ND*	ND*	ND*	ND*	ND*	ND*	Experimental	Repurposing of known drugs and clinical candidates	[136]
96	Rac3j	1.4 µM	ND*	HTS fluorescence-based enzymatic assay	ND*	ND*	ND*	ND*	ND*	ND*	Hit	Repurposing of known drugs and clinical candidates	[136]
97	Rac3k	1.15 µM	ND*	HTS fluorescence-based enzymatic assay	ND*	ND*	ND*	ND*	ND*	ND*	Hit	Repurposing of known drugs and clinical candidates	[136]
98	EACC	8.28 ± 1.11 µM	ND*	Enzymatic assay	>40 µM	Calu-3 cells	41.9 ± 7.1 µM	ND*	ND*	ND*	Hit	HTS Screening of a MedChemExpress bioactive compound library	[117]
99	KY226	3.39 ± 0.59 µM	ND*	Enzymatic assay	28.4 ± 2.3 µM	Calu-3 cells	49.3 ± 13.5 µM	ND*	ND*	ND*	Hit	HTS Screening of a MedChemExpress bioactive compound library	[117]
100	Tropifexor	5.11 µM	ND*	Enzymatic assay	4.03 ± 0.5 µM	Calu-3 cells	26.6 ± 1.4 µM	ND*	ND*	ND*	Experimental	HTS Screening of a MedChemExpress	[117]

												bioactive compound library	
101	Proanthocyanidin	2.4 ± 0.3 µM	ND*	HTS fluorescence-based enzymatic assay	2.5 ± 0.4 µM	Vero E6	56.4 ± 0.7 µM	ND*	ND*	ND*	Experimental	Screening of FDA approved drugs	[137]
102	Manidipine-2HCl	14.2 ± 1.7 µM	ND*	HTS fluorescence-based enzymatic assay	14.5 ± 0.8 µM	Vero E6	>100 µM	ND*	ND*	ND*	Hit	Screening of FDA approved drugs	[137]
103	Loperamide	33.5 ± 5.8 µM	ND*	HTS fluorescence-based enzymatic assay	11.4 ± 1.6 µM	Vero E6	>100 µM	ND*	ND*	ND*	Hit	Screening of FDA approved drugs	[137]
104	Levothyroxine	15.3 ± 4.2 µM	ND*	HTS fluorescence-based enzymatic assay	7.0 ± 2.2 µM	Vero E6	>100 µM	ND*	ND*	ND*	Hit	Screening of FDA approved drugs	[137]
105	Maprotiline	9.7 ± 0.3 µM	ND*	HTS fluorescence-based enzymatic assay	9.3 ± 0.1 µM	Vero E6	31.8 ± 0.4 µM	ND*	ND*	ND*	Hit	Screening of FDA approved drugs	[137]
106	Reserpine	5.7 ± 0.7 µM	ND*	HTS fluorescence-based enzymatic assay	6.6 ± 1.5 µM	Vero E6	>100 µM	ND*	ND*	ND*	Hit	Screening of FDA approved drugs	[137]
209	Chloroxine	7.24 ± 0.68 µM	ND*	PLpro protease-activity-based assay	ND*	ND*	ND*	ND*	ND*	ND*	Hit	HTS of a library of clinically approved drugs	[138]
210	Tashinone IIA sulfonate sodium	1.65±0.13 µM	ND*	PLpro protease-activity-based assay	ND*	ND*	ND*	ND*	ND*	ND*	Hit	HTS of a library of clinically approved drugs	[138]
107	Compound 1	93.7 µM	ND*	Enzymatic assay	ND*	ND*	ND*	ND*	ND*	ND*	Hit	Tanshinone IIA sulfonate sodium analogues	[139]
108	Compound 2	>100 µM	ND*	Enzymatic assay	ND*	ND*	ND*	ND*	ND*	ND*	Hit	Tanshinone IIA sulfonate sodium analogues	[139]

111	Compound 5	>100 µM	ND*	Enzymatic assay	ND*	ND*	ND*	ND*	ND*	ND*	Hit	Tanshinone IIA sulfonate sodium analogues	[139]
112	Compound 6	68.3 µM	ND*	Enzymatic assay	ND*	ND*	ND*	ND*	ND*	ND*	Hit	Tanshinone IIA sulfonate sodium analogues	[139]
113	Compound 7	6.0 µM	ND*	Fluorescence Polarization Assay	4.59 ± 1.22 µM	Vero E6	>800 µM	ND*	ND*	ND*	Experimental	Chloroxine derivative	[139]
115	Compound 9	8.9 µM	ND*	Fluorescence Polarization Assay	ND*	ND*	ND*	ND*	ND*	ND*	Hit	Chloroxine derivative	[139]
116	Compound 10	9.7 µM	ND*	Fluorescence Polarization Assay	ND*	ND*	ND*	ND*	ND*	ND*	Hit	Chloroxine derivative	[139]
117	Compound 11	7.6 µM	ND*	Fluorescence Polarization Assay	ND*	ND*	ND*	ND*	ND*	ND*	Hit	Chloroxine derivative	[139]
118	Compound 12	9.5 µM	ND*	Fluorescence Polarization Assay	ND*	ND*	ND*	ND*	ND*	ND*	Hit	Chloroxine derivative	[139]
119	4	43.2 ± 9.4 µM	ND*	Biochemical assay	1.7 ± 4.4 µM	Vero E6	ND*	ND*	ND*	ND*	Hit	Derivative of GRL-0617	[140]
120	7	12.7 ± 1.3 µM	ND*	Biochemical assay	5.2 ± 4.2 µM	Vero E6	ND*	ND*	ND*	ND*	Experimental	Amine-functionalized derivatives of GRL-0617	[140]
121	6	7.0 ± 0.6 µM	ND*	Biochemical assay	ND*	ND*	ND*	ND*	ND*	ND*	Hit	Amine-functionalized derivatives of GRL-0617	[140]
122	5	16.8 ± 2.9 µM	ND*	Biochemical assay	ND*	ND*	ND*	ND*	ND*	ND*	Hit	Amine-functionalized derivatives of GRL-0617	[140]
123	3	6.4 ± 4.2 µM	ND*	Biochemical assay	ND*	ND*	ND*	ND*	ND*	ND*	Hit	Amine-functionalized derivatives of GRL-0617	[140]
124	2	5.1 ± 0.7 µM	ND*	Biochemical assay	ND*	ND*	ND*	ND*	ND*	ND*	Hit	Amine-functionalized derivatives of GRL-0617	[140]
125	1	2.3 ± 0.2 µM	ND*	Biochemical assay	ND*	ND*	ND*	ND*	ND*	ND*	Hit	Derivative of GRL-0617	[140]

126	HE9	0.2 μM	ND*	Enzymatic assay	ND*	ND*	ND*	ND*	ND*	ND*	Hit	Natural phenolic compounds	[119]
127	HBA	1 μM	ND*	Enzymatic assay	ND*	ND*	ND*	ND*	ND*	ND*	Hit	Natural phenolic compounds	[119]
128	YRL	1.3 μM	ND*	Enzymatic assay	ND*	ND*	ND*	ND*	ND*	ND*	Hit	Natural phenolic compounds	[119]
129	1d	236 ± 107 nM	ND*	Biochemical assay	ND*	ND*	ND*	ND*	ND*	ND*	Experimental	Optimization of Ebselen's derivatives substituted on the phenyl ring	[141]
130	1e	256 ± 35 nM	ND*	Biochemical assay	ND*	ND*	ND*	ND*	ND*	ND*	Experimental	Optimization of Ebselen's derivatives substituted on the phenyl ring	[141]
131	2d	339 ± 109 nM	ND*	Biochemical assay	ND*	ND*	ND*	ND*	ND*	ND*	Hit	Optimization of Ebselen's derivatives substituted on the phenyl ring	[141]
132	2e	263 ± 121 nM	ND*	Biochemical assay	ND*	ND*	ND*	ND*	ND*	ND*	Hit	Optimization of Ebselen's derivatives substituted on the phenyl ring	[141]
133	11	2.64 ± 0.29 μM	ND*	Enzymatic assay	ND*	ND*	ND*	ND*	ND*	ND*	Hit	HTS of a library containing diverse compounds	[120]
134	12	2.69 ± 0.34 μM	ND*	Enzymatic assay	ND*	ND*	ND*	ND*	ND*	ND*	Hit	HTS of a library containing diverse compounds	[120]
135	13	4.32 ± 0.20 μM	ND*	Enzymatic assay	ND*	ND*	ND*	ND*	ND*	ND*	Hit	HTS of a library containing diverse compounds	[120]
136	14	1.76 ± 0.06 μM	ND*	Enzymatic assay	ND*	ND*	ND*	ND*	ND*	ND*	Hit	HTS of a library containing diverse compounds	[120]
137	15	3.75 ± 0.82 μM	ND*	Enzymatic assay	ND*	ND*	ND*	ND*	ND*	ND*	Hit	HTS of a library containing diverse compounds	[120]
138	16	1.37 ± 0.40 μM	ND*	Enzymatic assay	ND*	ND*	ND*	ND*	ND*	ND*	Hit	HTS of a library containing diverse compounds	[120]
139	17	0.82 ± 0.16 μM	ND*	Enzymatic assay	ND*	hACE2-HeLa	>10 μM	ND*	ND*	ND*	Hit	HTS of a library containing diverse compounds	[120]

140	18	0.80 ± 0.24 µM	ND*	Enzymatic assay	ND*	hACE2-HeLa	~10 µM	ND*	ND*	ND*	Lead	HTS of a library containing diverse compounds	[120]
141	19	0.44 ± 0.05 µM	ND*	Enzymatic assay	ND*	hACE2-HeLa	>10 µM	ND*	ND*	ND*	Experimental	HTS of a library containing diverse compounds	[120]
142	Jun9-10-1	16.7 ± 2.48 µM	ND*	FRET-based enzymatic assay	ND*	ND*	ND*	ND*	ND*	ND*	Hit	Hit Optimization from Enamine Collection	[121]
143	Jun9-10-2	98.8 ± 13.2 µM	ND*	FRET-based enzymatic assay	ND*	ND*	ND*	ND*	ND*	ND*	Hit	Hit Optimization from Enamine Collection	[121]
144	Jun9-10-3	19.6 ± 1.6 µM	ND*	FRET-based enzymatic assay	ND*	ND*	ND*	ND*	ND*	ND*	Hit	Hit Optimization from Enamine Collection	[121]
145	Jun9-10-4	16.2 ± 5.9 µM	ND*	FRET-based enzymatic assay	ND*	ND*	ND*	ND*	ND*	ND*	Hit	Hit Optimization from Enamine Collection	[121]
146	Jun9-10-5	12.8 ± 2.3 µM	ND*	FRET-based enzymatic assay	ND*	ND*	ND*	ND*	ND*	ND*	Hit	Hit Optimization from Enamine Collection	[121]
147	Jun9-10-6	23.7 ± 10.1 µM	ND*	FRET-based enzymatic assay	ND*	ND*	ND*	ND*	ND*	ND*	Hit	Hit Optimization from Enamine Collection	[121]
148	Jun9-10-7	17.4 ± 2.2 µM	ND*	FRET-based enzymatic assay	ND*	ND*	ND*	ND*	ND*	ND*	Hit	Hit Optimization from Enamine Collection	[121]
149	Jun9-10-8	20.5 ± 5.1 µM	ND*	FRET-based enzymatic assay	ND*	ND*	ND*	ND*	ND*	ND*	Hit	Hit Optimization from Enamine Collection	[121]
150	Jun9-10-9	38.7 ± 2.9 µM	ND*	FRET-based enzymatic assay	ND*	ND*	ND*	ND*	ND*	ND*	Hit	Hit Optimization from Enamine Collection	[121]
151	Jun9-13-1	78.1 ± 28.1 µM	ND*	FRET-based enzymatic assay	ND*	ND*	ND*	ND*	ND*	ND*	Hit	Hit Optimization from Enamine Collection	[121]
152	Jun9-13-2	>200 µM	ND*	FRET-based enzymatic assay	ND*	ND*	ND*	ND*	ND*	ND*	Hit	Hit Optimization from Enamine Collection	[121]
153	Jun9-13-7	7.29 ± 1.03 µM	ND*	FRET-based enzymatic assay	ND*	ND*	ND*	ND*	ND*	ND*	Hit	Hit Optimization from Enamine Collection	[121]
154	Jun9-13-9	6.67 ± 0.55 µM	ND*	FRET-based enzym	ND*	ND*	ND*	ND*	ND*	ND*	Hit	Hit Optimization from	[121]

				atic assay								Enamine Collection	
155	GC376	ND*	ND*	FRET-based assay	>60 µM	Vero E6	>60 µM	ND*	ND*	ND*	Hit	Hit Optimization from Enamine Collection	[121]
156	Jun9-53-2	0.89 ± 0.12 µM	ND*	FRET-based assay	25.19 ± 9.32 µM	Vero E6	>60 µM	ND*	ND*	ND*	Hit	Hit Optimization from Enamine Collection	[121]
157	Jun9-72-2	0.67 ± 0.08 µM	ND*	FRET-based assay	6.62 ± 1.31 µM	Vero E6	>60 µM	ND*	ND*	ND*	Lead	Hit Optimization from Enamine Collection	[121]
158	Jun9-75-4	0.62 ± 0.06 µM	ND*	FRET-based assay	7.88 ± 1.44 µM	Vero E6	47.48 ±14.63 µM	ND*	ND*	ND*	Lead	Hit Optimization from Enamine Collection	[121]
159	Jun9-75-5	0.56 ± 0.03 µM	ND*	FRET-based assay	>60 µM	FRET-based assay	>60 µM	ND*	ND*	ND*	Hit	Hit Optimization from Enamine Collection	[121]
160	Jun9-84-3	ND*	ND*	FRET-based assay	8.31 ± 2.68 µM	Vero E6	>60 µM	ND*	ND*	ND*	Hit	Hit Optimization from Enamine Collection	[121]
161	Jun9-85-1	0.66 ± 0.03 µM	ND*	FRET-based assay	7.81 ± 2.55 µM	Vero E6	>60 µM	ND*	ND*	ND*	Hit	Hit Optimization from Enamine Collection	[121]
162	Jun9-87-1	ND*	ND*	FRET-based assay	10.14 ± 3.52 µM	Vero E6	>60 µM	ND*	ND*	ND*	Hit	Hit Optimization from Enamine Collection	[121]
163	Jun9-87-2	0.90 ± 0.24 µM	ND*	FRET-based assay	55.07 ± 9.77 µM	FRET-based assay	>60 µM	ND*	ND*	ND*	Hit	Hit Optimization from Enamine Collection	[121]
164	Jun9-87-3	0.80 ± 0.10 µM	ND*	FRET-based assay	22.34 ± 9.75 µM	Vero E6	49.52 ± 17.28 µM	ND*	ND*	ND*	Hit	Hit Optimization from Enamine Collection	[121]
165	Jun9-13-7	7.29 ± 1.03 µM	ND*	FRET-based assay	ND*	ND*	ND*	ND*	ND*	ND*	Hit	Hit Optimization from Enamine Collection	[121]
166	Jun9-13-9	6.67 ± 0.55 µM	ND*	FRET-based assay	ND*	ND*	ND*	ND*	ND*	ND*	Hit	Hit Optimization from Enamine Collection	[121]
167	Jun9-81-1	19.23 ± 1.70 µM	ND*	FRET-based assay	ND*	ND*	ND*	ND*	ND*	ND*	Hit	Hit Optimization from Enamine Collection	[121]

168	Jun9-67-2	7.10 ± 2.47 µM	ND*	FRET-based assay	ND*	ND*	ND*	ND*	ND*	ND*	Hit	Hit Optimization from Enamine Collection	[121]
169	Jun9-68-1	11.71 ± 2.05 µM	ND*	FRET-based assay	ND*	ND*	ND*	ND*	ND*	ND*	Hit	Hit Optimization from Enamine Collection	[121]
170	Jun9-80-4	1.58 ± 0.26 µM	ND*	FRET-based assay	ND*	ND*	ND*	ND*	ND*	ND*	Hit	Hit Optimization from Enamine Collection	[121]
171	Jun9-68-3	5.13 ± 0.74 µM	ND*	FRET-based assay	ND*	ND*	ND*	ND*	ND*	ND*	Hit	Hit Optimization from Enamine Collection	[121]
172	Jun9-68-2	1.30 ± 0.14 µM	ND*	FRET-based assay	ND*	ND*	ND*	ND*	ND*	ND*	Hit	Hit Optimization from Enamine Collection	[121]
173	Jun9-75-2	1.54 ± 0.30 µM	ND*	FRET-based assay	ND*	ND*	ND*	ND*	ND*	ND*	Hit	Hit Optimization from Enamine Collection	[121]
174	Jun9-84-2	2.47 ± 0.44 µM	ND*	FRET-based assay	ND*	ND*	ND*	ND*	ND*	ND*	Hit	Hit Optimization from Enamine Collection	[121]
175	Jun9-86-2	6.49 ± 1.14 µM	ND*	FRET-based assay	ND*	ND*	ND*	ND*	ND*	ND*	Hit	Hit Optimization from Enamine Collection	[121]
176	Jun9-87-3	0.80 ± 0.10 µM	ND*	FRET-based assay	ND*	ND*	ND*	ND*	ND*	ND*	Ht	Hit Optimization from Enamine Collection	[121]
177	Jun9-75-3	8.89 ± 2.23 µM	ND*	FRET-based assay	ND*	ND*	ND*	ND*	ND*	ND*	Hit	Hit Optimization from Enamine Collection	[121]
178	Jun9-67-1	2.72 ± 0.28 µM	ND*	FRET-based assay	ND*	ND*	ND*	ND*	ND*	ND*	Hit	Hit Optimization from Enamine Collection	[121]
179	Jun9-86-8	5.50 ± 0.27 µM	ND*	FRET-based assay	ND*	ND*	ND*	ND*	ND*	ND*	Hit	Hit Optimization from Enamine Collection	[121]
180	Jun9-86-5	6.12 ± 0.51 µM	ND*	FRET-based assay	ND*	ND*	ND*	ND*	ND*	ND*	Hit	Hit Optimization from Enamine Collection	[121]
181	Jun9-81-3	1.85 ± 0.15 µM	ND*	FRET-based assay	ND*	ND*	ND*	ND*	ND*	ND*	Hit	Hit Optimization from Enamine Collection	[121]
182	Jun9-85-5	16.59 ± 2.60 µM	ND*	FRET-based assay	ND*	ND*	ND*	ND*	ND*	ND*	Hit	Hit Optimization from	[121]

												Enamine Collection	
183	Jun9-85-6	4.42 ± 1.22 µM	ND*	FRET-based assay	ND*	ND*	ND*	ND*	ND*	ND*	Hit	Hit Optimization from Enamine Collection	[121]
184	Jun9-81-2	1.44 ± 0.22 µM	ND*	FRET-based assay	ND*	ND*	ND*	ND*	ND*	ND*	Hit	Hit Optimization from Enamine Collection	[121]
185	Jun9-86-4	9.22 ± 3.31 µM	ND*	FRET-based assay	ND*	ND*	ND*	ND*	ND*	ND*	Hit	Hit Optimization from Enamine Collection	[121]
189	1	159 nM	ND*	Predicted	ND*	ND*	ND*	ND*	ND*	ND*	Hit	Structure-Based Screening of ENAMINE Real Database	[142]
190	2	226 nM	ND*	Predicted	ND*	ND*	ND*	ND*	ND*	ND*	Hit	Structure-Based Screening of ENAMINE Real Database	[142]
191	3	227 nM	ND*	Predicted	ND*	ND*	ND*	ND*	ND*	ND*	Hit	Structure-Based Screening of ENAMINE Real Database	[142]
192	4	248 nM	ND*	Predicted	ND*	ND*	ND*	ND*	ND*	ND*	Hit	Structure-Based Screening of ENAMINE Real Database	[142]
193	5	270 nM	ND*	Predicted	ND*	ND*	ND*	ND*	ND*	ND*	Hit	Structure-Based Screening of ENAMINE Real Database	[142]
194	6	287 nM	ND*	Predicted	ND*	ND*	ND*	ND*	ND*	ND*	Hit	Structure-Based Screening of ENAMINE Real Database	[142]
195	7	303 nM	ND*	Predicted	ND*	ND*	ND*	ND*	ND*	ND*	Experimental	Structure-Based Screening of ENAMINE Real Database	[142]
196	8	324 nM	ND*	Predicted	ND*	ND*	ND*	ND*	ND*	ND*	Hit	Structure-Based Screening of ENAMINE	[142]

												Real Database	
197	9	359 nM	ND*	Predicted	ND*	ND*	ND*	ND*	ND*	ND*	Hit	Structure-Based Screening of ENAMINE Real Database	[142]
198	10	370 nM	ND*	Predicted	ND*	ND*	ND*	ND*	ND*	ND*	Hit	Structure-Based Screening of ENAMINE Real Database	[142]
199	11	383 nM	ND*	Predicted	ND*	ND*	ND*	ND*	ND*	ND*	Hit	Structure-Based Screening of ENAMINE Real Database	[142]
200	12	385 nM	ND*	Predicted	ND*	ND*	ND*	ND*	ND*	ND*	Hit	Structure-Based Screening of ENAMINE Real Database	[142]
201	13	420 nM	ND*	Predicted	ND*	ND*	ND*	ND*	ND*	ND*	Hit	Structure-Based Screening of ENAMINE Real Database	[142]
202	14	432 nM	ND*	Predicted	ND*	ND*	ND*	ND*	ND*	ND*	Hit	Structure-Based Screening of ENAMINE Real Database	[142]
203	15	433 nM	ND*	Predicted	ND*	ND*	ND*	ND*	ND*	ND*	Hit	Structure-Based Screening of ENAMINE Real Database	[142]
204	16	446 nM	ND*	Predicted	ND*	ND*	ND*	ND*	ND*	ND*	Hit	Structure-Based Screening of ENAMINE Real Database	[142]
205	17	476 nM	ND*	Predicted	ND*	ND*	ND*	ND*	ND*	ND*	Hit	Structure-Based Screening of ENAMINE Real Database	[142]
206	18	483 nM	ND*	Predicted	ND*	ND*	ND*	ND*	ND*	ND*	Hit	Structure-Based Screening of ENAMINE	[142]

												Real Database	
207	19	498 nM	N D*	Predict ed	ND*	ND*	ND*	ND*	ND*	ND*	Hit	Structure-Based Screening of ENAMINE Real Database	[142]
208	20	505 nM	N D*	Predict ed	ND*	ND*	ND*	ND*	ND*	ND*	Hit	Structure-Based Screening of ENAMINE Real Database	[142]
211	Acrifl avine	1.66 µM	N D*	HTS FRET-based assay	ND*	ND*	ND*	ND*	ND*	ND*	Experi mental	HTS of a library containing diverse clinical compounds	[125]
214	Profla vine Hemis ulfate	2.77 µM	N D*	HTS FRET-based assay	ND*	ND*	ND*	ND*	ND*	ND*	Hit	HTS of a library containing diverse clinical compounds	[125]
215	PHA-66575 2	3.92 µM	N D*	HTS FRET-based assay	ND*	ND*	ND*	ND*	ND*	ND*	Hit	HTS of a library containing diverse clinical compounds	[125]
216	Chlor ophyll in	>100 µM	N D*	HTS FRET-based assay	ND*	ND*	ND*	ND*	ND*	ND*	Hit	HTS of a library containing diverse clinical compounds	[125]
217	Clofa zimin e	>100 µM	N D*	HTS FRET-based assay	ND*	ND*	ND*	ND*	ND*	ND*	Hit	HTS of a library containing diverse clinical compounds	[125]
218	Dithra nol	24.83 µM	N D*	HTS FRET-based assay	ND*	ND*	ND*	ND*	ND*	ND*	Hit	HTS of a library containing diverse clinical compounds	[125]
219	Proto porph yrinix	3.335 µM	N D*	HTS FRET-based assay	ND*	ND*	ND*	ND*	ND*	ND*	Hit	HTS of a library containing diverse clinical compounds	[125]
220	Tanni c acid	6.039 µM	N D*	HTS FRET-based assay	ND*	ND*	ND*	ND*	ND*	ND*	Hit	HTS of a library containing diverse clinical compounds	[125]
221	Evans Blue	5.88 µM	N D*	HTS FRET-based assay	ND*	ND*	ND*	ND*	ND*	ND*	Hit	HTS of a library containing diverse clinical compounds	[125]

222	Hpericin	7.02 μM	ND*	HTS FRET-based assay	ND*	ND*	ND*	ND*	ND*	ND*	Hit	HTS of a library containing diverse clinical compounds	[125]
223	Verteporfin	4.201 μM	ND*	HTS FRET-based assay	ND*	ND*	ND*	ND*	ND*	ND*	Hit	HTS of a library containing diverse clinical compounds	[125]
224	Acridine orange base	15.10 μM	ND*	HTS FRET-based assay	ND*	ND*	ND*	ND*	ND*	ND*	Hit	Acriflavine analogue	[125]
225	Acridine-3,6-diamine sulfate	3.728 μM	ND*	HTS FRET-based assay	ND*	ND*	ND*	ND*	ND*	ND*	Hit	Acriflavine analogue	[125]
226	1	73.60 ± 11.94 μM	ND*	In vitro enzymatic assay	ND*	Vero	23.77 μM	ND*	ND*	ND*	Hit	Retention of naphthyridine core from a vHTS hit compound 1	[143]
227	82	79.77 ± 20.37 μM	ND*	In vitro enzymatic assay	ND*	Vero	>50 μM	ND*	ND*	ND*	Hit	Retention of naphthyridine core from a vHTS hit compound 1	[143]
228	83	54.8 ± 20.68 μM	ND*	In vitro enzymatic assay	ND*	Vero	43.58 μM	ND*	ND*	ND*	Hit	Retention of naphthyridine core from a vHTS hit compound 1	[143]
229	84	39.5 ± 4.62 μM	ND*	In vitro enzymatic assay	ND*	Vero	47.32 μM	ND*	ND*	ND*	Hit	Retention of naphthyridine core from a vHTS hit compound 1	[143]
230	85	15.06 ± 2.19 μM	ND*	In vitro enzymatic assay	ND*	Vero	7.47 μM	ND*	ND*	ND*	Experimental	Retention of naphthyridine core from a vHTS hit compound 1	[143]
231	Compound 6	11±3 μM	ND*	Enzymatic assays	ND*	ND*	ND*	ND*	ND*	ND*	Hit	Acetamide derivative of GRL0617	

232	3	>100 μM	N D*	Fluoro genic peptide assay	ND*	ND*	ND*	ND*	ND*	ND*	Hit	GRL-0617 Redesign with N,N'-Acetylaceto hydrazine Linker	
234	5	24 μM	N D*	Fluoro genic peptide assay	ND*	ND*	ND*	ND*	ND*	ND*	Hit	GRL-0617 Redesign with N,N'-Acetylaceto hydrazine Linker	
236	7	0.094 μM	N D*	Fluoro genic peptide assay	1.1 μM	ND*	ND*	38.9	ND*	ND*	Experi mental	GRL-0617 Redesign with N,N'-Acetylaceto hydrazine Linker	
237	8	0.23 μM	N D*	Fluoro genic peptide assay	ND*	ND*	ND*	ND*	ND*	ND*	Hit	GRL-0617 Redesign with N,N'-Acetylaceto hydrazine Linker	
238	9	5.4 μM	N D*	Fluoro genic peptide assay	34 μM	ND*	ND*	689.4	ND*	ND*	Hit	GRL-0617 Redesign with N,N'-Acetylaceto hydrazine Linker	
239	10	4.4 μM	N D*	Fluoro genic peptide assay	ND*	ND*	ND*	ND*	ND*	ND*	Hit	GRL-0617 Redesign with N,N'-Acetylaceto hydrazine Linker	
240	11	8 μM	N D*	Fluoro genic peptide assay	ND*	ND*	ND*	ND*	ND*	ND*	Hit	GRL-0617 Redesign with N,N'-Acetylaceto hydrazine Linker	
241	12	0.098 μM		Fluoro genic peptide assay	ND*	ND*	1.3 μM	ND*	ND*	ND*	Hit	GRL-0617 Redesign with N,N'-Acetylaceto hydrazine Linker	

242	13	>200 µM		Fluorogenic peptide assay	ND*	ND*	5.0 µM	ND*	ND*	ND*	Hit	GRL-0617 Redesign with N,N'-Acetylacetohydrazine Linker	
243	14	100 µM	ND*	Fluorogenic peptide assay	ND*	ND*	ND*	<38.5	ND*	ND*	Hit	GRL-0617 Redesign with N,N'-Acetylacetohydrazine Linker	
244	15	6.2 µM	ND*	Fluorogenic peptide assay	ND*	ND*	ND*	ND*	ND*	ND*	Hit	GRL-0617 Redesign with N,N'-Acetylacetohydrazine Linker	
246	ZN-2-180 (101)	6.08 µM	ND*	Enzymatic assays	ND*	ND*	ND*	ND*	ND*	ND*	Hit	Extention of the amine group of GRL-0617	[123]
247	ZN-2-181 (2)	1.1 µM	ND*	Enzymatic assays	ND*	ND*	ND*	ND*	ND*	ND*	Hit	Extention of the amine group of GRL-0617	[123]
248	ZN-2-182 (3)	5.5 µM	ND*	Enzymatic assays	ND*	ND*	ND*	ND*	ND*	ND*	Hit	Extention of the amine group of GRL-0617	[123]
249	ZN-2-183 (4)	6.0 µM	ND*	Enzymatic assays	ND*	ND*	ND*	ND*	ND*	ND*	Hit	Extention of the amine group of GRL-0617	[123]
250	ZN-2-184 (5)	1.01 µM	ND*	Enzymatic assays	ND*	ND*	ND*	ND*	ND*	ND*	Hit	Extention of the amine group of GRL-0617	[123]
251	ZN-2-185 (6)	0.6 µM	ND*	Enzymatic assays	ND*	ND*	ND*	ND*	ND*	ND*	Hit	Extention of the amine group of GRL-0617	[123]
252	ZN-2-186 (7)	1.2 µM	ND*	Enzymatic assays	ND*	ND*	ND*	ND*	ND*	ND*	Hit	Extention of the amine group of GRL-0617	[123]
253	ZN-2-187 (8)	0.8 µM	ND*	Enzymatic assays	ND*	ND*	ND*	ND*	ND*	ND*	Hit	Extention of the amine group of GRL-0617	[123]
254	ZN-2-189 (9)	0.7 µM	ND*	Enzymatic assays	ND*	ND*	ND*	ND*	ND*	ND*	Hit	Extention of the amine group of GRL-0617	[123]

255	ZN-2-188-1 (10)	1.6 µM	N D*	Enzymatic assays	ND*	ND*	ND*	ND*	ND*	ND*	Hit	Extention of the amine group of GRL-0617	[123]
256	ZN-2-188-2 (11)	4.3 µM	N D*	Enzymatic assays	ND*	ND*	ND*	ND*	ND*	ND*	Hit	Extention of the amine group of GRL-0617	[123]
257	ZN-2-197 (12)	2.4 µM	N D*	Enzymatic assays	ND*	ND*	ND*	ND*	ND*	ND*	Hit	Extention of the amine group of GRL-0617	[123]
258	ZN-3-56 (13)	3.9 µM	N D*	Enzymatic assays	ND*	ND*	ND*	ND*	ND*	ND*	Hit	Extention of the amine group of GRL-0617	[123]
259	DY2-144 (14)	1.3 µM	N D*	Enzymatic assays	ND*	ND*	ND*	ND*	ND*	ND*	Hit	Extention of the amine group of GRL-0617	[123]
260	DY2-137 (15)	3.3 µM	N D*	Enzymatic assays	ND*	ND*	ND*	ND*	ND*	ND*	Hit	Extention of the amine group of GRL-0617	[123]
261	DY2-138-2 (16)	6.1 µM	N D*	Enzymatic assays	ND*	ND*	ND*	ND*	ND*	ND*	Hit	Extention of the amine group of GRL-0617	[123]
265	ZN-2-192 (20)	4.8 µM	N D*	Enzymatic assays	ND*	ND*	ND*	ND*	ND*	ND*	Hit	Extention of the amine group of GRL-0617	[123]
269	DY2-115 (24)	7 µM	N D*	Enzymatic assays	ND*	ND*	ND*	ND*	ND*	ND*	Hit	Extention of the amine group of GRL-0617	[123]
272	DY-3-15 (27)	0.8 µM	N D*	Enzymatic assays	ND*	ND*	ND*	ND*	ND*	ND*	Hit	Extention of the amine group of GRL-0617	[123]
273	DY-3-65 (28)	6.3 µM	N D*	Enzymatic assays	ND*	ND*	ND*	ND*	ND*	ND*	Hit	Extention of the amine group of GRL-0617	[123]
274	DY-3-70 (29)	6.4 µM	N D*	Enzymatic assays	ND*	ND*	ND*	ND*	ND*	ND*	Hit	Extention of the amine group of GRL-0617	[123]
276	ZN-3-55 (31)	7.4 µM	N D*	Enzymatic assays	ND*	ND*	ND*	ND*	ND*	ND*	Hit	Extention of the amine group of GRL-0617	[123]
278	ZN-3-66 (33)	4.1 µM	N D*	Enzymatic assays	ND*	ND*	ND*	ND*	ND*	ND*	Hit	Extention of the amine group of GRL-0617	[123]
279	ZN-3-70 (34)	10.7 µM	N D*	Enzymatic assays	ND*	ND*	ND*	ND*	ND*	ND*	Hit	Extention of the amine	[123]

												group of GRL-0617	
290	XDY 2-62 (45)	3.3 µM	N D*	Enzym atic assays	ND*	ND*	ND*	ND*	ND*	ND*	Hit	Optimizatio n of GRL-0617 (methyl, naphthalene , and aniline groups)	[123]
293	YF4-136 (48)	4.7 µM	N D*	Enzym atic assays	ND*	ND*	ND*	ND*	ND*	ND*	Hit	Optimizatio n of GRL-0617 (methyl, naphthalene , and aniline groups)	[123]
297	ZN-3-45 (52)	5.7 µM	N D*	Enzym atic assays	ND*	ND*	ND*	ND*	ND*	ND*	Hit	Optimizatio n of GRL-0617 (methyl, naphthalene , and aniline groups)	[123]
298	DY-3-59 (53)	6.7 µM	N D*	Enzym atic assays	ND*	ND*	ND*	ND*	ND*	ND*	Hit	Optimizatio n of GRL-0617 (methyl, naphthalene , and aniline groups)	[123]
299	DY-3-66 (54)	3.3 µM	N D*	Enzym atic assays	ND*	ND*	ND*	ND*	ND*	ND*	Hit	Optimizatio n of GRL-0617 (methyl, naphthalene , and aniline groups)	[123]
300	ZN-3-79 (55)	1.9 µM	N D*	Enzym atic assays	ND*	ND*	ND*	ND*	ND*	ND*	Hit	Optimizatio n of GRL-0617 (methyl, naphthalene , and aniline groups)	[123]
301	ZN-3-67 (56)	8.5 µM	N D*	Enzym atic assays	ND*	ND*	ND*	ND*	ND*	ND*	Hit	Optimizatio n of GRL-0617 (methyl, naphthalene , and aniline groups)	[123]
302	ZN-3-71 (57)	10.9 µM	N D*	Enzym atic assays	ND*	ND*	ND*	ND*	ND*	ND*	Hit	Optimizatio n of GRL-0617 (methyl, naphthalene , and aniline groups)	[123]
303	DY2-149 (58)	1.6 µM	N D*	Enzym atic assays	ND*	ND*	ND*	ND*	ND*	ND*	Hit	Optimizatio n of GRL-0617 (methyl, naphthalene , and	[123]

												aniline groups)	
304	ZN-3-79 (59)	1.9 µM	ND*	Enzymatic assays	ND*	ND*	ND*	ND*	ND*	ND*	Hit	Optimization of GRL-0617 (methyl, naphthalene, and aniline groups)	[123]
305	DY-2-153 (60)	1.8 µM	ND*	Enzymatic assays	ND*	ND*	ND*	ND*	ND*	ND*	Hit	Optimization of GRL-0617 (methyl, naphthalene, and aniline groups)	[123]
309	ZN-3-74 (64)	2.8 µM	ND*	Enzymatic assays	ND*	ND*	ND*	ND*	ND*	ND*	Hit	Optimization of GRL-0617 (methyl, naphthalene, and aniline groups)	[123]
310	ZN-3-80 (65)	0.59 µM	ND*	Enzymatic assays	ND*	ND*	ND*	5.64	ND*	ND*	Experimental	Optimization of GRL-0617 (methyl, naphthalene, and aniline groups)	[123]
311	XR8-8 (66)	1.3 µM	ND*	Enzymatic assays	ND*	ND*	ND*	ND*	ND*	ND*	Hit	Optimization of GRL-0617 (methyl, naphthalene, and aniline groups)	[123]
312	XR8-9 (67)	1.8 µM	ND*	Enzymatic assays	ND*	ND*	ND*	ND*	ND*	ND*	Hit	Optimization of GRL-0617 (methyl, naphthalene, and aniline groups)	[123]
313	XR8-14 (68)	1.2 µM	ND*	Enzymatic assays	ND*	ND*	ND*	ND*	ND*	ND*	Hit	Optimization of GRL-0617 (methyl, naphthalene, and aniline groups)	[123]
314	XR8-15 (69)	0.9 µM	ND*	Enzymatic assays	ND*	ND*	ND*	ND*	ND*	ND*	Hit	Optimization of GRL-0617 (methyl, naphthalene, and aniline groups)	[123]
315	XR8-16 (70)	1.6 µM	ND*	Enzymatic assays	ND*	ND*	ND*	ND*	ND*	ND*	Hit	Optimization of GRL-0617 (methyl, naphthalene, and	[123]

												aniline groups)	
316	XR8-17 (71)	2.7 µM	ND*	Enzymatic assays	ND*	ND*	ND*	ND*	ND*	ND*	Hit	Optimization of GRL-0617 (methyl, naphthalene, and aniline groups)	[123]
317	XR8-23 (72)	0.39 µM	ND*	Enzymatic assays	1.4 ± 0.1 µM	A549-hACE2	21.6 ± 1.1 µM	5.89	ND*	ND*	Experimental	Optimization of GRL-0617 (methyl, naphthalene, and aniline groups)	[123]
318	XR8-24 (73)	0.56 µM	ND*	Enzymatic assays	1.2 ± 0.2 µM	A549-hACE2	>50 µM	9.60	ND*	ND*	Experimental	Optimization of GRL-0617 (methyl, naphthalene, and aniline groups)	[123]
319	XR8-30 (74)	0.75 µM	ND*	Enzymatic assays	ND*	ND*	ND*	ND*	ND*	ND*	Hit	Optimization of GRL-0617 (methyl, naphthalene, and aniline groups)	[123]
320	XR8-32-1 (75)	0.97 µM	ND*	Enzymatic assays	ND*	ND*	ND*	ND*	ND*	ND*	Hit	Optimization of GRL-0617 (methyl, naphthalene, and aniline groups)	[123]
321	XR8-32-2 (76)	0.81 µM	ND*	Enzymatic assays	ND*	ND*	ND*	ND*	ND*	ND*	Hit	Optimization of GRL-0617 (methyl, naphthalene, and aniline groups)	[123]
322	XR8-35 (77)	1.79 µM	ND*	Enzymatic assays	ND*	ND*	ND*	ND*	ND*	ND*	Hit	Optimization of GRL-0617 (methyl, naphthalene, and aniline groups)	[123]
323	XR8-38 (78)	0.76 µM	ND*	Enzymatic assays	ND*	ND*	ND*	ND*	ND*	ND*	Hit	Optimization of GRL-0617 (methyl, naphthalene, and aniline groups)	[123]
324	XR8-39 (79)	1.1 µM	ND*	Enzymatic assays	ND*	ND*	ND*	ND*	ND*	ND*	Hit	Optimization of GRL-0617 (methyl, naphthalene, and	[123]

												aniline groups)	
325	XR8-40 (80)	0.82 µM	ND*	Enzymatic assays	ND*	ND*	ND*	ND*	ND*	ND*	Hit	Optimization of GRL-0617 (methyl, naphthalene, and aniline groups)	[123]
326	XR8-49 (81)	0.64 µM	ND*	Enzymatic assays	ND*	ND*	ND*	ND*	ND*	ND*	Hit	Optimization of GRL-0617 (methyl, naphthalene, and aniline groups)	[123]
327	XR8-51 (82)	1.1 µM	ND*	Enzymatic assays	ND*	ND*	ND*	ND*	ND*	ND*	Hit	Optimization of GRL-0617 (methyl, naphthalene, and aniline groups)	[123]
328	XR8-56 (83)	2.2 µM	ND*	Enzymatic assays	ND*	ND*	ND*	ND*	ND*	ND*	Hit	Optimization of GRL-0617 (methyl, naphthalene, and aniline groups)	[123]
329	XR8-57 (84)	0.70 µM	ND*	Enzymatic assays	ND*	ND*	ND*	0.27	ND*	ND*	Hit	Optimization of GRL-0617 (methyl, naphthalene, and aniline groups)	[123]
330	XR8-61 (85)	6.5 µM	ND*	Enzymatic assays	ND*	ND*	ND*	ND*	ND*	ND*	Hit	Optimization of GRL-0617 (methyl, naphthalene, and aniline groups)	[123]
331	XR8-65 (86)	0.33 µM	ND*	Enzymatic assays	ND*	ND*	ND*	ND*	ND*	ND*	Hit	Optimization of GRL-0617 (methyl, naphthalene, and aniline groups)	[123]
332	XR8-66 (87)	0.62 µM	ND*	Enzymatic assays	ND*	ND*	ND*	ND*	ND*	ND*	Hit	Optimization of GRL-0617 (methyl, naphthalene, and aniline groups)	[123]
333	XR8-67 (88)	0.17 µM	ND*	Enzymatic assays	ND*	ND*	ND*	ND*	ND*	ND*	Hit	Optimization of GRL-0617 (methyl, naphthalene, and	[123]

												aniline groups)	
334	XR8-69 (89)	0.37 µM	ND*	Enzymatic assays	ND*	ND*	ND*	ND*	ND*	ND*	Hit	Optimization of GRL-0617 (methyl, naphthalene, and aniline groups)	[123]
335	XR8-77 (90)	0.64 µM	ND*	Enzymatic assays	ND*	ND*	ND*	ND*	ND*	ND*	Hit	Optimization of GRL-0617 (methyl, naphthalene, and aniline groups)	[123]
336	XR8-79 (91)	0.41 µM	ND*	Enzymatic assays	ND*	ND*	ND*	ND*	ND*	ND*	Hit	Optimization of GRL-0617 (methyl, naphthalene, and aniline groups)	[123]
337	XR8-83 (92)	0.21 µM	ND*	Enzymatic assays	ND*	ND*	ND*	ND*	ND*	ND*	Hit	Optimization of GRL-0617 (methyl, naphthalene, and aniline groups)	[123]
338	XR8-84 (93)	0.43 µM	ND*	Enzymatic assays	ND*	ND*	ND*	ND*	ND*	ND*	Hit	Optimization of GRL-0617 (methyl, naphthalene, and aniline groups)	[123]
339	XR8-89 (94)	0.113 µM	ND*	Enzymatic assays	ND*	ND*	ND*	ND*	ND*	ND*	Hit	Optimization of GRL-0617 (methyl, naphthalene, and aniline groups)	[123]
340	XR8-96 (95)	0.25 µM	ND*	Enzymatic assays	ND*	ND*	ND*	ND*	ND*	ND*	Hit	Optimization of GRL-0617 (methyl, naphthalene, and aniline groups)	[123]
341	XR8-98 (96)	0.81 µM	ND*	Enzymatic assays	ND*	ND*	ND*	ND*	ND*	ND*	Hit	Optimization of GRL-0617 (methyl, naphthalene, and aniline groups)	[123]
342	XR8-101 (97)	1.8 µM	ND*	Enzymatic assays	ND*	ND*	ND*	ND*	ND*	ND*	Hit	Optimization of GRL-0617 (methyl, naphthalene, and	[123]

												aniline groups)	
343	XR8-103 (98)	1.1 µM	ND*	Enzymatic assays	ND*	ND*	ND*	ND*	ND*	ND*	Hit	Optimization of GRL-0617 (methyl, naphthalene, and aniline groups)	[123]
344	XR8-104 (99)	2.3 µM	ND*	Enzymatic assays	ND*	ND*	ND*	ND*	ND*	ND*	Hit	Optimization of GRL-0617 (methyl, naphthalene, and aniline groups)	[123]
345	XR8-106 (100)	1.4 µM	ND*	Enzymatic assays	ND*	ND*	ND*	ND*	ND*	ND*	Hit	Optimization of GRL-0617 (methyl, naphthalene, and aniline groups)	[123]
347	SIMR 1440	4.117 + 1.410 ng/µL	ND*	Enzymatic assays	ND*	ND*	ND*	ND*	ND*	ND*	Hit	Exploiting shallow pockets	[124]
349	SIMR 1489	33.224 + 5.78 ng/µL	ND*	Enzymatic assays	ND*	ND*	ND*	ND*	ND*	ND*	Hit	Exploiting shallow pockets	[124]
350	SIMR 1493	0.0659 ± 0.0071 ng/µL	ND*	Enzymatic assays	ND*	Vero E6	43.12 µg/mL	ND*	ND*	ND*	Hit	Exploiting shallow pockets	[124]
351	SIMR 1783	27.577 ± 2.27 ng/µL	ND*	Enzymatic assays	ND*	ND*	ND*	ND*	ND*	ND*	Hit	Exploiting shallow pockets	[124]
352	SIMR 1888	4.094 ± 2.40 ng/µL	ND*	Enzymatic assays	ND*	ND*	ND*	ND*	ND*	ND*	Hit	Exploiting shallow pockets	[124]
355	SIMR 2593	0.852 ± 0.89 ng/µL	ND*	Enzymatic assays	ND*	ND*	ND*	ND*	ND*	ND*	Hit	Exploiting shallow pockets	[124]
356	SIMR 3030	0.0399 ± 0.081 ng/µL	ND*	Enzymatic assays	ND*	Vero E6	55.63 µg/mL	ND*	ND*	ND*	Experimental	Exploiting shallow pockets	[124]
357	SIMR 7088	6.0726 ± 0.65 ng/µL	ND*	Enzymatic assays	ND*	ND*	ND*	ND*	ND*	ND*	Hit	Exploiting shallow pockets	[124]
358	SIMR 7109	12.8315 ± 2.057 ng/µL	ND*	Enzymatic assays	ND*	ND*	ND*	ND*	ND*	ND*	Hit	Exploiting shallow pockets	[124]
359	SIMR 7111	13.947 ± 0.69 ng/µL	ND*	Enzymatic assays	ND*	ND*	ND*	ND*	ND*	ND*	Hit	Exploiting shallow pockets	[124]
360	Jun9722	0.67 ± 0.08 µM	ND*	ND*	ND*	ND*	23.69 ± 1.58 µM	778.8	ND*	ND*	Lead	Quinoline-based optimization of GRL-0617	[126]
361	Jun9871	0.87 ± 0.12 µM	ND*	ND*	ND*	ND*	14.43 ± 0.77 µM	2485.8	ND*	ND*	Hit	Quinoline-based optimization of GRL-0617	[126]

362	Jun9851	0.66 ± 0.12 µM	ND*	ND*	4.11 ± 0.64 µM	Caco-2 cells	35.47 ± 1.52 µM	429.1	ND*	ND*	Lead	Quinoline-based optimization of GRL-0617	[126]
366	Jun11213	0.69 µM	ND*	ND*	5.30 ± 1.26 µM	Caco-2 cells	ND*	25.7	ND*	ND*	Lead	Quinoline-based optimization of GRL-0617	[126]
367	Jun11165	0.63 µM	ND*	ND*	6.04 ± 0.84 µM	Caco-2 cells	ND*	11.8	ND*	ND*	Lead	Quinoline-based optimization of GRL-0617	[126]
368	Jun11243	0.50 ± 0.10	ND*	ND*	ND*	Caco-2 cells	15.20 ± 0.30	<9.6	ND*	ND*	Lead	Quinoline-based optimization of GRL-0617	[126]
369	Jun10714	2.81 ± 0.48 µM	ND*	ND*	ND*	ND*	ND*	ND*	ND*	ND*	Hit	Quinoline-based optimization of GRL-0617	[126]
370	Jun10725	0.58 ± 0.08 µM	ND*	ND*	ND*	Vero E6	31.5 ± 2.70 µM	ND*	ND*	ND*	Lead	Quinoline-based optimization of GRL-0617	[126]
371	Jun10132	4.97 ± 1.30 µM	ND*	ND*	ND*	ND*	ND*	ND*	ND*	ND*	Hit	Quinoline-based optimization of GRL-0617	[126]
372	Jun10827	1.23 ± 0.24 µM	ND*	ND*	ND*	ND*	ND*	ND*	ND*	ND*	Hit	Quinoline-based optimization of GRL-0617	[126]
373	Jun10828	2.36 ± 0.15 µM	ND*	ND*	ND*	ND*	ND*	ND*	ND*	ND*	Hit	Quinoline-based optimization of GRL-0617	[126]
374	Jun10713	>20 µM	ND*	ND*	ND*	ND*	ND*	ND*	ND*	ND*	Hit	Quinoline-based optimization of GRL-0617	[126]
375	Jun10836	>20 µM	ND*	ND*	ND*	ND*	ND*	ND*	ND*	ND*	Hit	Quinoline-based optimization of GRL-0617	[126]
376	Jun10872	>20 µM	ND*	ND*	ND*	ND*	ND*	ND*	ND*	ND*	Hit	Quinoline-based optimization of GRL-0617	[126]

377	Jun10 873	>20 µM	N D*	ND*	ND*	ND*	ND*	ND*	ND*	ND*	Hit	Quinoline-based optimization of GRL-0617	[126]
378	Jun10 837	0.48 ± 0.04 µM	N D*	ND*	ND*	Vero E6 cell	>100 µM	ND*	ND*	ND*	Hit	Quinoline-based optimization of GRL-0617	[126]
379	Jun-43	0.67 ± 0.14 µM	N D*	ND*	ND*	Vero E6 cell	24.69 ± 1.3 µM	ND*	ND*	ND*	Experimental	Quinoline-based optimization of GRL-0617	[126]
380	Jun10 711	0.60 ± 0.08 µM	N D*	ND*	ND*	Vero E6	31.80 ± 1.22 µM	ND*	ND*	ND*	Hit	Quinoline-based optimization of GRL-0617	[126]
381	Jun10 712	0.53 ± 0.09 µM	N D*	ND*	ND*	Vero E6	32.5 ± 1.52 µM	ND*	ND*	ND*	Hit	Quinoline-based optimization of GRL-0617	[126]
382	Jun10 715	6.45 ± 1.94 µM	N D*	ND*	ND*	ND*	ND*	ND*	ND*	ND*	Hit	Quinoline-based optimization of GRL-0617	[126]
383	Jun10 716	5.17 ± 1.24 µM	N D*	ND*	ND*	ND*	ND*	ND*	ND*	ND*	Hit	Quinoline-based optimization of GRL-0617	[126]
384	Jun10 722	0.63 ± 0.10 µM	N D*	ND*	ND*	Vero E6	28.30 ± 1.50 µM	ND*	ND*	ND*	Hit	Quinoline-based optimization of GRL-0617	[126]
385	Jun-21	0.96 ± 0.11 µM	N D*	ND*	ND*	Vero E6	13.67 ± 3.60 µM	ND*	ND*	ND*	Hit	Retention of naphthalene ring and extended the amino group	[126]
386	Jun10 888	2.50 ± 0.37 µM	N D*	ND*	ND*	ND*	ND*	ND*	ND*	ND*	Hit	Retention of naphthalene ring and extended the amino group	[126]
387	Jun11 76	1.66 ± 0.19 µM	N D*	ND*	ND*	ND*	ND*	ND*	ND*	ND*	Hit	Retention of naphthalene ring and extended the amino group	[126]

388	Jun11 114	1.27 ± 0.09 µM	N D*	ND*	ND*	ND*	ND*	ND*	ND*	ND*	Hit	Retention of naphthalene ring and extended the amino group	[126]
389	Jun10 889	>20 µM	N D*	ND*	ND*	ND*	ND*	ND*	ND*	ND*	Hit	Retention of naphthalene ring and extended the amino group	[126]
390	Jun10 823	2.45 ± 0.14 µM	N D*	ND*	ND*	ND*	ND*	ND*	ND*	ND*	Hit	Retention of naphthalene ring and extended the amino group	[126]
391	Jun-95	2.03 ± 0.33 µM	N D*	ND*	ND*	ND*	ND*	ND*	ND*	ND*	Hit	Retention of naphthalene ring and extended the amino group	[126]
392	Jun10 824	1.76 ± 0.33 µM	N D*	ND*	ND*	ND*	ND*	ND*	ND*	ND*	Hit	Retention of naphthalene ring and extended the amino group	[126]
393	Jun-91	5.89 ± 0.44 µM	N D*	ND*	ND*	ND*	ND*	ND*	ND*	ND*	Hit	Retention of naphthalene ring and extended the amino group	[126]
394	Jun10 821	2.66 ± 0.35 µM	N D*	ND*	ND*	ND*	ND*	ND*	ND*	ND*	Hit	Retention of naphthalene ring and extended the amino group	[126]
395	Jun10 822	2.34 ± 0.22 µM	N D*	ND*	ND*	ND*	ND*	ND*	ND*	ND*	Hit	Retention of naphthalene ring and extended the amino group	[126]
396	Jun10 825	5.03 ± 0.52 µM	N D*	ND*	ND*	ND*	ND*	ND*	ND*	ND*	Hit	Retention of naphthalene ring and extended the amino group	[126]
397	Jun10 826	2.36 ± 0.27 µM	N D*	ND*	ND*	ND*	ND*	ND*	ND*	ND*	Hit	Retention of naphthalene ring and extended the amino group	[126]
398	Jun-92	3.00 ± 0.52 µM	N D*	ND*	ND*	ND*	ND*	ND*	ND*	ND*	Hit	Retention of naphthalene ring and extended	[126]

												the amino group	
399	Jun-93	7.09 ± 1.50 µM	ND*	ND*	ND*	ND*	ND*	ND*	ND*	ND*	Hit	Retention of naphthalene ring and extended the amino group	[126]
400	Jun-94	3.39 ± 0.34 µM	ND*	ND*	ND*	ND*	ND*	ND*	ND*	ND*	Hit	Retention of naphthalene ring and extended the amino group	[126]
401	Jun1183	0.69 ± 0.08 µM	ND*	ND*	ND*	ND*	ND*	ND*	ND*	ND*	Hit	Derivatives of Jun9722 and XR8-23	[126]
402	Jun11115	0.89 ± 0.11 µM	ND*	ND*	ND*	ND*	ND*	ND*	ND*	ND*	Hit	Derivatives of Jun9722 and XR8-23	[126]
403	Jun11217	0.99 ± 0.10 µM	ND*	ND*	ND*	ND*	ND*	ND*	ND*	ND*	Hit	Derivatives of Jun9722 and XR8-23	[126]
404	Jun11168	1.41 ± 0.54 µM	ND*	ND*	ND*	ND*	ND*	ND*	ND*	ND*	Hit	Derivatives of Jun9722 and XR8-23	[126]
405	Jun11278	2.82 ± 0.36 µM	ND*	ND*	ND*	ND*	ND*	ND*	ND*	ND*	Hit	Derivatives of Jun9722 and XR8-23	[126]
406	Jun11164	>20 µM	ND*	ND*	ND*	ND*	ND*	ND*	ND*	ND*	Hit	Derivatives of Jun9722 and XR8-23	[126]
407	Jun11166	1.22 ± 0.10 µM	ND*	ND*	ND*	ND*	ND*	ND*	ND*	ND*	Hit	Derivatives of Jun9722 and XR8-23	[126]
408	Jun11184	1.54 ± 0.18 µM	ND*	ND*	ND*	ND*	ND*	ND*	ND*	ND*	Hit	Derivatives of Jun9722 and XR8-23	[126]
409	Jun11273	0.69 ± 0.17 µM	ND*	ND*	ND*	Vero E6	45.2 ± 2.40 µM	ND*	ND*	ND*	Hit	Derivatives of Jun9722 and XR8-23	[126]
410	Jun11287	2.25 ± 0.54 µM	ND*	ND*	ND*	ND*	ND*	ND*	ND*	ND*	Hit	Derivatives of Jun9722 and XR8-23	[126]
411	Jun11185	0.89 ± 0.05 µM	ND*	ND*	ND*	ND*	ND*	ND*	ND*	ND*	Hit	Derivatives of Jun9722 and XR8-23	[126]
412	Jun11167	>20 µM	ND*	ND*	ND*	ND*	ND*	ND*	ND*	ND*	Hit	Derivatives of Jun9722 and XR8-23	[126]
413	Jun11251	1.08 ± 0.08 µM	ND*	ND*	ND*	ND*	ND*	ND*	ND*	ND*	Hit	Derivatives of Jun9722 and XR8-23	[126]
414	Jun11253	0.97 ± 0.07 µM	ND*	ND*	ND*	ND*	ND*	ND*	ND*	ND*	Hit	Derivatives of Jun9722 and XR8-23	[126]

415	Jun11 165	0.53 ± 0.18 µM	N D*	ND*	ND*	Vero E6	>30	ND*	ND*	ND*	Hit	Derivatives of Jun9722 and XR8-23	[126]
416	Jun11 846	>10 µM	N D*	ND*	ND*	ND*	ND*	ND*	ND*	ND*	Hit	Derivatives of Jun9722 and XR8-23	[126]
417	Jun11 813	1.30 ± 0.24 µM	N D*	ND*	ND*	ND*	ND*	ND*	ND*	ND*	Hit	Derivatives of Jun9722 and XR8-23	[126]
418	Jun11 814	3.07 ± 0.23 µM	N D*	ND*	ND*	ND*	ND*	ND*	ND*	ND*	Hit	Derivatives of Jun9722 and XR8-23	[126]
419	Jun11 275	0.59 ± 0.06 µM	N D*	ND*	ND*	Vero E6	15.00 ± 0.80 µM	ND*	ND*	ND*	Hit	Derivatives of Jun9722 and XR8-23	[126]
420	Jun11 276	0.94 ± 0.09 µM	N D*	ND*	ND*	ND*	ND*	ND*	ND*	ND*	Hit	Derivatives of Jun9722 and XR8-23	[126]
421	Jun11 213	0.63 ± 0.14 µM	N D*	ND*	ND*	Vero E6	103.40 ± 5.60 µM	ND*	ND*	ND*	Hit	Derivatives of Jun9722 and XR8-23	[126]
422	Jun11 244	0.96 ± 0.08 µM	N D*	ND*	ND*	ND*	ND*	ND*	ND*	ND*	Hit	Derivatives of Jun9722 and XR8-23	[126]
423	Jun11 288	1.14 ± 0.06 µM	N D*	ND*	ND*	ND*	ND*	ND*	ND*	ND*	Hit	Derivatives of Jun9722 and XR8-23	[126]
424	Jun11 289	1.51 ± 0.15 µM	N D*	ND*	ND*	ND*	ND*	ND*	ND*	ND*	Hit	Derivatives of Jun9722 and XR8-23	[126]
425	Jun11 246	1.48 ± 0.32 µM	N D*	ND*	ND*	ND*	ND*	ND*	ND*	ND*	Hit	Derivatives of Jun9722 and XR8-23	[126]
426	Jun11 247	1.16 ± 0.06 µM	N D*	ND*	ND*	ND*	ND*	ND*	ND*	ND*	Hit	Derivatives of Jun9722 and XR8-23	[126]
427	Jun11 277	3.35 ± 0.51 µM	N D*	ND*	ND*	ND*	ND*	ND*	ND*	ND*	Hit	Derivatives of Jun9722 and XR8-23	[126]
428	Jun11 248	1.49 ± 0.18 µM	N D*	ND*	ND*	ND*	ND*	ND*	ND*	ND*	Hit	Derivatives of Jun9722 and XR8-23	[126]
429	Jun11 249	1.03 ± 0.05 µM	N D*	ND*	ND*	ND*	ND*	ND*	ND*	ND*	Hit	Derivatives of Jun9722 and XR8-23	[126]
430	Jun11 252	1.96 ± 0.61 µM	N D*	ND*	ND*	ND*	ND*	ND*	ND*	ND*	Hit	Derivatives of Jun9722 and XR8-23	[126]
431	Jun11 274	2.78 ± 0.38 µM	N D*	ND*	ND*	ND*	ND*	ND*	ND*	ND*	Hit	Derivatives of Jun9722 and XR8-23	[126]
432	Jun11 924	177.7 ± 10.0 nM	N D*	FRET-based enzymatic assay	ND*	ND*	36.4 ± 1.3 µM	ND*	ND*	ND*	Experimental	Biarylphenyl benzamide scaffolds	[132]

433	Jun11 922	171.2 ± 14.0 nM	N D*	FRET-based enzymatic assay	ND*	ND*	11.0 ± 0.4 µM	ND*	ND*	ND*	Hit	Biarylphenyl benzamide scaffolds	[132]
434	Jun12 38	339.8 ± 29.0 nM	N D*	FRET-based enzymatic assay	ND*	ND*	5.9 ± 0.7 µM	ND*	ND*	ND*	Hit	Biarylphenyl benzamide scaffolds	[132]
435	Jun12 112	184.1 ± 22.0 nM	N D*	FRET-based enzymatic assay	ND*	ND*	7.9 ± 1.4 µM	ND*	ND*	ND*	Hit	Biarylphenyl benzamide scaffolds	[132]
436	Jun12 71	116.6 ± 16.0 nM	N D*	FRET-based enzymatic assay	ND*	ND*	4.6 ± 0.4 µM	ND*	ND*	ND*	Hit	Biarylphenyl benzamide scaffolds	[132]
437	Jun11 923	147.2 ± 7.0 nM	N D*	FRET-based enzymatic assay	ND*	ND*	11.6 ± 0.7 µM	ND*	ND*	ND*	Hit	Biarylphenyl benzamide scaffolds	[132]
438	Jun12 111	118.2 ± 12.0 nM	N D*	FRET-based enzymatic assay	ND*	ND*	8.23 ± 0.26 µM	ND*	ND*	ND*	Hit	Biarylphenyl benzamide scaffolds	[132]
439	Jun11 945	162.8 ± 0.5 nM	N D*	FRET-based enzymatic assay	ND*	ND*	13.5 ± 0.7 µM	ND*	ND*	ND*	Hit	Biarylphenyl benzamide scaffolds	[132]
440	Jun11 874	65.1 ± 1.4 nM	N D*	FRET-based enzymatic assay	ND*	ND*	7.0 ± 1.1 µM	ND*	ND*	ND*	Hit	Biarylphenyl benzamide scaffolds	[132]
441	Jun11 875	66.2 ± 2.0 nM	N D*	FRET-based enzymatic assay	ND*	ND*	4.5 ± 1.6 µM	ND*	ND*	ND*	Hit	Biarylphenyl benzamide scaffolds	[132]
442	Jun11 899	75.9 ± 2.0 nM	N D*	FRET-based enzymatic assay	ND*	ND*	6.5 ± 0.3 µM	ND*	ND*	ND*	Hit	Biarylphenyl benzamide scaffolds	[132]
443	Jun11 8910	367.1 ± 3.0 nM	N D*	FRET-based enzymatic assay	ND*	ND*	96.8 ± 3.6 µM	ND*	ND*	ND*	Hit	Biarylphenyl benzamide scaffolds	[132]
444	Jun11 898	132.6 ± 4.0 nM	N D*	FRET-based enzymatic assay	ND*	ND*	19.1 ± 4.2 µM	ND*	ND*	ND*	Hit	Biarylphenyl benzamide scaffolds	[132]
445	Jun11 931	99.3 ± 4.0 nM	N D*	FRET-based enzymatic assay	ND*	ND*	9.4 ± 1.0 µM	ND*	ND*	ND*	Hit	Biarylphenyl benzamide scaffolds	[132]
446	Jun11 9415	143.2 ± 9.0 nM	N D*	FRET-based enzymatic assay	ND*	ND*	7.6 ± 0.3 µM	ND*	ND*	ND*	Hit	Biarylphenyl benzamide scaffolds	[132]
447	Jun11 903	202.2 ± 8.0 nM	N D*	FRET-based enzym	ND*	ND*	7.7 ± 0.4 µM	ND*	ND*	ND*	Hit	Biarylphenyl benzamide scaffolds	[132]

				atic assay									
448	Jun11 932	83.7 ± 6.0 nM	N D*	FRET-based enzymatic assay	ND*	ND*	16.0 ± 1.2 µM	ND*	ND*	ND*	Hit	Biarylphenyl benzamide scaffolds	[132]
449	Jun11 34	109.0 ± 7.0 nM	N D*	FRET-based enzymatic assay	ND*	ND*	37.1 ± 4.4 µM	ND*	ND*	ND*	Hit	Biarylphenyl benzamide scaffolds	[132]
450	Jun11 933	103.0 ± 7.0 nM	N D*	FRET-based enzymatic assay	ND*	ND*	13.8 ± 1.9 µM	ND*	ND*	ND*	Hit	Biarylphenyl benzamide scaffolds	[132]
451	Jun11 943	167.3 ± 7.0 nM	N D*	FRET-based enzymatic assay	ND*	ND*	9.1 ± 0.3 µM	ND*	ND*	ND*	Hit	Biarylphenyl benzamide scaffolds	[132]
452	Jun12 18	1.95 ± 0.08 µM	N D*	FRET-based enzymatic assay	ND*	ND*	89.8 ± 3.0 µM	ND*	ND*	ND*	Hit	Biarylphenyl benzamide scaffolds	[132]
453	Jun11 941	151.1 ± 4.0 nM	N D*	FRET-based enzymatic assay	1.8 ± 0.3 µM	Flip GFP	54.8 ± 8.3 µM	ND*	ND*	ND*	Hit	Biarylphenyl benzamide scaffolds	[132]
454	Jun11 942	178.5 ± 8.0 nM	N D*	FRET-based enzymatic assay	2.2 ± 0.2 µM	Flip GFP	160.4 ± 59.2 µM	ND*	ND*	ND*	Hit	Biarylphenyl benzamide scaffolds	[132]
455	Jun12 12	390.3 ± 25.0 nM	N D*	FRET-based enzymatic assay	ND*	ND*	17.8 ± 0.3 µM	ND*	ND*	ND*	Hit	Biarylphenyl benzamide scaffolds	[132]
456	Jun12 15	181.7 ± 6.0 nM	N D*	FRET-based enzymatic assay	4.7 ± 0.5 µM	Flip GFP	77.6 ± 5.5 µM	ND*	ND*	ND*	Hit	Biarylphenyl benzamide scaffolds	[132]
457	Jun12 47	165.3 ± 9.0 nM	N D*	FRET-based enzymatic assay	>60 µM	Flip GFP	>125 µM	ND*	ND*	ND*	Hit	Biarylphenyl benzamide scaffolds	[132]
458	Jun12 382	443.4 ± 44 nM	N D*	FRET-based enzymatic assay	ND*	ND*	31.0 ± 2.1 µM	ND*	ND*	ND*	Hit	Biarylphenyl benzamide scaffolds	[132]
459	Jun12 46	463.6 ± 39.1 nM	N D*	FRET-based enzymatic assay	ND*	ND*	39.6 ± 4.7 µM	ND*	ND*	ND*	Hit	Biarylphenyl benzamide scaffolds	[132]
460	Jun12 11	152.7 ± 6.0 nM	N D*	FRET-based enzymatic assay	ND*	ND*	9.8 ± 1.0 µM	ND*	ND*	ND*	Hit	Biarylphenyl benzamide scaffolds	[132]
461	Jun12 44	81.9 ± 8.0 nM	N D*	FRET-based enzymatic assay	ND*	ND*	9.0 ± 0.7 µM	ND*	ND*	ND*	Hit	Biarylphenyl benzamide scaffolds	[132]

462	Jun12 51	74.7 ± 4.0 nM	N D*	FRET-based enzym atic assay	ND*	ND*	7.6 ± 0.4 µM	ND*	ND*	ND*	Hit	Biarylphen yl benzamide scaffolds	[132]
463	Jun12 52	116.4 ± 10.0 nM	N D*	FRET-based enzym atic assay	ND*	ND*	8.2 ± 0.2 µM	ND*	ND*	ND*	Hit	Biarylphen yl benzamide scaffolds	[132]
464	Jun12 54	111.8 ± 11.0 nM	N D*	FRET-based enzym atic assay	ND*	ND*	9.7 ± 2.2 µM	ND*	ND*	ND*	Hit	Biarylphen yl benzamide scaffolds	[132]
465	Jun12 57	113.5 ± 8.0 nM	N D*	FRET-based enzym atic assay	ND*	ND*	18.0 ± 1.0 µM	ND*	ND*	ND*	Hit	Biarylphen yl benzamide scaffolds	[132]
466	Jun12 1210	81.9 ± 5.7 nM	N D*	FRET-based enzym atic assay	13.6 ± 1.9 µM	Flip GFP	>125 µM	ND*	ND*	ND*	Hit	Biarylphen yl benzamide scaffolds	[132]
467	Jun12 149	73.8 ± 3.0 nM	N D*	FRET-based enzym atic assay	ND*	ND*	6.3 ± 1.1 µM	ND*	ND*	ND*	Hit	Biarylphen yl benzamide scaffolds	[132]
468	Jun12 129	90.9 ± 4.6 nM	N D*	FRET-based enzym atic assay	ND*	ND*	8.3 ± 1.2 µM	ND*	ND*	ND*	Hit	Biarylphen yl benzamide scaffolds	[132]
469	Jun12 1910	66.4 ± 3.0 nM	N D*	FRET-based enzym atic assay	1.1 ± 0.2 µM	Flip GFP	17.7 ± 2.1 µM	ND*	ND*	ND*	Hit	Biarylphen yl benzamide scaffolds	[132]
470	Jun12 511	91.5 ± 17.0 nM	N D*	FRET-based enzym atic assay	ND*	ND*	6.2 ± 0.2 µM	ND*	ND*	ND*	Hit	Biarylphen yl benzamide scaffolds	[132]
471	Jun12 145	108.5 ± 6.2 nM	N D*	FRET-based enzym atic assay	1.3 ± 0.3 µM	Flip GFP	31.3 ± 5.4 µM	ND*	ND*	ND*	Hit	Biarylphen yl benzamide scaffolds	[132]
472	Jun12 681	120.2 ± 5.1 nM	N D*	FRET-based enzym atic assay	1.8 ± 0.3 µM	Flip GFP	114.5 ± 3.4 µM	ND*	ND*	ND*	Hit	Biarylphen yl benzamide scaffolds	[132]
473	Jun12 1911	73.2 ± 6.4 nM	N D*	FRET-based enzym atic assay	>60 µM	Flip GFP	>125 µM	ND*	ND*	ND*	Hit	Biarylphen yl benzamide scaffolds	[132]
474	Jun12 336	118.2 ± 6.4 nM	N D*	FRET-based enzym atic assay	ND*	ND*	>125 µM	ND*	ND*	ND*	Hit	Biarylphen yl benzamide scaffolds	[132]
475	Jun12 467	73.26 ± 6 nM	N D*	FRET-based enzym atic assay	1.7 ± 0.4 µM	Flip GFP	165.2 ± 26.5 µM	ND*	ND*	ND*	Hit	Biarylphen yl benzamide scaffolds	[132]
476	jun12 351	98.8 ± 5.2 nM	N D*	FRET-based enzym	2.0 ± 0.4 µM	Flip GFP	>125 µM	ND*	ND*	ND*	Hit	Biarylphen yl benzamide scaffolds	[132]

				atic assay									
477	Jun12 199	108.8 ± 10.2 nM	N D*	FRET-based enzymatic assay	0.8 ± 0.2 µM	Flip GFP	63.2 ± 11.3 µM	ND*	ND*	ND*	Hit	Biarylphenyl benzamide scaffolds	[132]
478	Jun12 197	102.7 ± 10.2 nM	N D*	FRET-based enzymatic assay	0.6 ± 0.1 µM	Flip GFP	43.7 ± 5.1 µM	ND*	ND*	ND*	Hit	Biarylphenyl benzamide scaffolds	[132]
479	Jun12 603	112.2 ± 6.0 nM	N D*	FRET-based enzymatic assay	2.4 ± 0.4 µM	Flip GFP	61.0 ± 19.3 µM	ND*	ND*	ND*	Hit	Biarylphenyl benzamide scaffolds	[132]
480	Jun12 446	132.5 ± 4.2 nM	N D*	FRET-based enzymatic assay	ND*	ND*	8.6 ± 0.5 µM	ND*	ND*	ND*	Hit	Biarylphenyl benzamide scaffolds	[132]
481	Jun12 278	88.1 ± 11.5 nM	N D*	FRET-based enzymatic assay	1.0 ± 0.4 µM	Flip GFP	37.3 ± 3.8 µM	ND*	ND*	ND*	Hit	Biarylphenyl benzamide scaffolds	[132]
482	Jun12 162	98.3 ± 7.0 nM	N D*	FRET-based enzymatic assay	2.1 ± 0.2 µM	Flip GFP	42.3 ± 2.3 µM	ND*	ND*	ND*	Hit	Biarylphenyl benzamide scaffolds	[132]
483	Jun12 208	91.6 ± 6.0 nM	N D*	FRET-based enzymatic assay	16.9 ± 3.3 µM	Flip GFP	167.8 ± 15.8 µM	ND*	ND*	ND*	Hit	Biarylphenyl benzamide scaffolds	[132]
484	Jun12 235	162.2 ± 10.0 nM	N D*	FRET-based enzymatic assay	ND*	ND*	16.5 ± 6.8 µM	ND*	ND*	ND*	Hit	Biarylphenyl benzamide scaffolds	[132]
485	Jun12 281	111.9 ± 9.0 nM	N D*	FRET-based enzymatic assay	1.8± 0.5 µM	Flip GFP	59.2 ± 10.0 µM	ND*	ND*	ND*	Hit	Biarylphenyl benzamide scaffolds	[132]
486	Jun12 303	121.5 ± 4.0 nM	N D*	FRET-based enzymatic assay	2.6 ± 0.2 µM	Flip GFP	56.8 ± 7.4 µM	ND*	ND*	ND*	Hit	Biarylphenyl benzamide scaffolds	[132]
487	Jun12 284	96.6 ± 6.0 nM	N D*	FRET-based enzymatic assay	2.1 ± 0.4 µM	Flip GFP	30.4 ± 9.5 µM	ND*	ND*	ND*	Hit	Biarylphenyl benzamide scaffolds	[132]
488	Jun12 165	145.0 ± 10.0 nM	N D*	FRET-based enzymatic assay	ND*	ND*	>125 µM	ND*	ND*	ND*	Hit	Biarylphenyl benzamide scaffolds	[132]
489	Jun12 168	72.4 ± 3.0 nM	N D*	FRET-based enzymatic assay	9.7 ± 1.5 µM	Flip GFP	34.5 ± 10.2 µM	ND*	ND*	ND*	Hit	Biarylphenyl benzamide scaffolds	[132]
490	Jun12 163	102.0 ± 7.0 nM	N D*	FRET-based enzymatic assay	ND*	ND*	>125 µM	ND*	ND*	ND*	Hit	Biarylphenyl benzamide scaffolds	[132]

491	Jun12 164	92.4 ± 4.0 nM	N D*	FRET-based enzymatic assay	ND*	ND*	>125 µM	ND*	ND*	ND*	Hit	Biarylphenyl benzamide scaffolds	[132]
492	Jun12 198	142. 0 ± 19.0 nM	N D*	FRET-based enzymatic assay	ND*	ND*	>125 µM	ND*	ND*	ND*	Hit	Biarylphenyl benzamide scaffolds	[132]
493	Jun12 242	90.47 ± 4.0 nM	N D*	FRET-based enzymatic assay	>60 µM	Flip GFP	>125 µM	ND*	ND*	ND*	Hit	Biarylphenyl benzamide scaffolds	[132]
494	Jun12 147	110.3 ± 9.0 nM	N D*	FRET-based enzymatic assay	2.1 ± 0.5 µM	Flip GFP	65.0 ± 9.2 µM	ND*	ND*	ND*	Hit	Biarylphenyl benzamide scaffolds	[132]
495	Jun12 759	206.7 ± 8.2 nM	N D*	FRET-based enzymatic assay	ND*	ND*	80.3 ± 14.6 µM	ND*	ND*	ND*	Hit	Biarylphenyl benzamide scaffolds	[132]
496	Jun12 379	147.8 ± 10.0 nM	N D*	FRET-based enzymatic assay	2.8 ± 0.5 µM	Flip GFP	194.2 ± 15.9 µM	ND*	ND*	ND*	Hit	Biarylphenyl benzamide scaffolds	[132]
497	Jun12 528	98.4 ± 6.2 nM	N D*	FRET-based enzymatic assay	4.5 ± 1.2 µM	Flip GFP	>125 µM	ND*	ND*	ND*	Hit	Biarylphenyl benzamide scaffolds	[132]
498	Jun12 529	96.8 ± 13.5 nM	N D*	FRET-based enzymatic assay	2.1 ± 0.2 µM	Flip GFP	28.1 ± 4.7 µM	ND*	ND*	ND*	Hit	Biarylphenyl benzamide scaffolds	[132]
499	Jun12 531	112.4 ± 10.3 nM	N D*	FRET-based enzymatic assay	3.3 ± 0.5 µM	Flip GFP	49.9 ± 5.3 µM	ND*	ND*	ND*	Hit	Biarylphenyl benzamide scaffolds	[132]
500	Jun12 584	123.9 ± 4.3 nM	N D*	FRET-based enzymatic assay	1.6 ± 0.3 µM	Flip GFP	49.1 ± 2.7 µM	ND*	ND*	ND*	Hit	Biarylphenyl benzamide scaffolds	[132]
501	Jun12 602	116.7 ± 9.2 nM	N D*	FRET-based enzymatic assay	14.7 ± 2.5 µM	Flip GFP	442.6 ± 38.4 µM	ND*	ND*	ND*	Hit	Biarylphenyl benzamide scaffolds	[132]
502	Jun12 703	83.1 ± 4.2 nM	N D*	FRET-based enzymatic assay	4.9 ± 0.8 µM	Flip GFP	ND*	ND*	ND*	ND*	Hit	Biarylphenyl benzamide scaffolds	[132]
503	Jun12 713	86.70 ± 6 nM	N D*	FRET-based enzymatic assay	ND*	ND*	107.2 ± 6.2 µM	ND*	ND*	ND*	Hit	Biarylphenyl benzamide scaffolds	[132]
504	Jun12 395	121.9 ± 8.0 nM	N D*	FRET-based enzymatic assay	2.9 ± 0.3 µM	Flip GFP	109.38 ± 16.16 µM	ND*	ND*	ND*	Hit	Biarylphenyl benzamide scaffolds	[132]
505	Jun12 827	324.9 ± 46.3 nM	N D*	FRET-based enzym	ND*	ND*	23.7 ± 2.0 µM	ND*	ND*	ND*	Hit	Biarylphenyl benzamide scaffolds	[132]

				atic assay									
506	Jun12 831	202.5 ± 18.3 nM	N D*	FRET-based enzymatic assay	ND*	ND*	27.8 ± 1.3 µM	ND*	ND*	ND*	Hit	Biarylphenyl benzamide scaffolds	[132]
507	Jun12 833	207.9 ± 9.3 nM	N D*	FRET-based enzymatic assay	ND*	ND*	17.6 ± 4.1 µM	ND*	ND*	ND*	Hit	Biarylphenyl benzamide scaffolds	[132]
508	Jun12 834	263.8 ± 26.0 nM	N D*	FRET-based enzymatic assay	ND*	ND*	158.8 ± 10.9 µM	ND*	ND*	ND*	Hit	Biarylphenyl benzamide scaffolds	[132]
509	Jun12 835	232.7 ± 10.4 nM	N D*	FRET-based enzymatic assay	ND*	ND*	33.1 ± 2.6 µM	ND*	ND*	ND*	Hit	Biarylphenyl benzamide scaffolds	[132]
510	Jun12 836	336.1 ± 14.2 nM	N D*	FRET-based enzymatic assay	ND*	ND*	70.5 ± 4.3 µM	ND*	ND*	ND*	Hit	Biarylphenyl benzamide scaffolds	[132]
511	Jun12 837	435.7 ± 78.6 nM	N D*	FRET-based enzymatic assay	ND*	ND*	10.3 ± 0.6 µM	ND*	ND*	ND*	Hit	Biarylphenyl benzamide scaffolds	[132]
512	Jun13 14	222.7 ± 23.2 nM	N D*	FRET-based enzymatic assay	2.8 ±0.6 µM	Flip GFP	45.8 ± 3.6 µM	ND*	ND*	ND*	Hit	Biarylphenyl benzamide scaffolds	[132]
513	Jun12 839	270.4 ± 12.3 nM	N D*	FRET-based enzymatic assay	ND*	ND*	14.7 ± 1.0 µM	ND*	ND*	ND*	Hit	Biarylphenyl benzamide scaffolds	[132]
514	Jun12 682	106.8 ± 5.0 nM	N D*	FRET-based enzymatic assay	1.1 ± 0.1 µM	Flip GFP	61.3 ± 4.5 µM	ND*	ND*	ND*	Experimental	Biarylphenyl benzamide scaffolds	[132]
515	Jun12 924	240.2 ± 10.0 nM	N D*	FRET-based enzymatic assay	ND*	ND*	180.6 ± 9.2 µM	ND*	ND*	ND*	Hit	Biarylphenyl benzamide scaffolds	[132]
516	Jun12 763	164.6 ± 23.0 nM	N D*	FRET-based enzymatic assay	ND*	ND*	>125 µM	ND*	ND*	ND*	Hit	Biarylphenyl benzamide scaffolds	[132]
517	A0	2.71 ± 0.18 µM	N D*	Enzymatic assay	ND*	ND*	ND*	ND*	ND*	ND*	Lead	Hit identification using HTS	[127]
518	1	16.60 ± 3.18 µM	N D*	Enzymatic assay	ND*	ND*	ND*	ND*	ND*	ND*	Hit	Optimization of HTS hit A0	[127]
519	2	>20 µM	N D*	Enzymatic assay	ND*	ND*	ND*	ND*	ND*	ND*	Hit	Optimization of HTS hit A0	[127]
520	3	>20 µM	N D*	Enzymatic assay	ND*	ND*	ND*	ND*	ND*	ND*	Hit	Optimization of HTS hit A0	[127]

521	4	>20 μM	ND*	Enzymatic assay	ND*	ND*	ND*	ND*	ND*	ND*	Hit	Optimization of HTS hit A0	[127]
522	5	>20 μM	ND*	Enzymatic assay	ND*	ND*	ND*	ND*	ND*	ND*	Hit	Optimization of HTS hit A0	[127]
523	6	7.09 ± 0.43 μM	ND*	Enzymatic assay	ND*	ND*	ND*	ND*	ND*	ND*	Hit	Optimization of HTS hit A0	[127]
524	7	>20 μM	ND*	Enzymatic assay	ND*	ND*	ND*	ND*	ND*	ND*	Hit	Optimization of HTS hit A0	[127]
525	8	>20 μM	ND*	Enzymatic assay	ND*	ND*	ND*	ND*	ND*	ND*	Hit	Optimization of HTS hit A0	[127]
526	9	>20 μM	ND*	Enzymatic assay	ND*	ND*	ND*	ND*	ND*	ND*	Hit	Optimization of HTS hit A0	[127]
527	XR8-89	0.163 ± 0.045 μM	ND*	Enzymatic assay	>40 μM	Cell-based assay (FlipGFP)	>40 μM	ND*	ND*	ND*	Hit	Optimization of HTS hit A0	[127]
528	10	1.07 ± 0.13 μM	ND*	Enzymatic assay	13.63 ± 3.13 μM	Cell-based assay (FlipGFP)	>40 μM	ND*	ND*	ND*	Hit	Optimization of HTS hit A0	[127]
529	11	8.15 ± 0.50 μM	ND*	Enzymatic assay	ND*	ND*	ND*	ND*	ND*	ND*	Hit	Optimization of HTS hit A0	[127]
530	12	6.08 ± 0.44 μM	ND*	Enzymatic assay	ND*	ND*	ND*	ND*	ND*	ND*	Hit	Optimization of HTS hit A0	[127]
531	13	14.11 ± 0.70 μM	ND*	Enzymatic assay	ND*	ND*	ND*	ND*	ND*	ND*	Hit	Optimization of HTS hit A0	[127]
532	14	>20 μM	ND*	Enzymatic assay	ND*	ND*	ND*	ND*	ND*	ND*	Hit	Optimization of HTS hit A0	[127]
533	15	19.82 ± 0.85 μM	ND*	Enzymatic assay	ND*	ND*	ND*	ND*	ND*	ND*	Hit	Optimization of HTS hit A0	[127]
534	16	13.75 ± 0.52 μM	ND*	Enzymatic assay	ND*	ND*	ND*	ND*	ND*	ND*	Hit	Optimization of HTS hit A0	[127]
535	17	2.63 ± 9.14 μM	ND*	Enzymatic assay	ND*	ND*	ND*	ND*	ND*	ND*	Hit	Optimization of HTS hit A0	[127]
536	18	>20 μM	ND*	Enzymatic assay	ND*	ND*	ND*	ND*	ND*	ND*	Hit	Optimization of HTS hit A0	[127]
537	19	>20 μM	ND*	Enzymatic assay	ND*	ND*	ND*	ND*	ND*	ND*	Hit	Optimization of HTS hit A0	[127]
538	20	3.58 ± 0.29 μM	ND*	Enzymatic assay	ND*	ND*	ND*	ND*	ND*	ND*	Hit	Optimization of HTS hit A0	[127]
539	21	4.61 ± 0.33 μM	ND*	Enzymatic assay	ND*	ND*	ND*	ND*	ND*	ND*	Hit	Optimization of HTS hit A0	[127]

540	22	3.51 ± 0.31 µM	ND*	Enzymatic assay	ND*	ND*	ND*	ND*	ND*	ND*	Hit	Optimization of HTS hit A0	[127]
541	23	>20 µM	ND*	Enzymatic assay	ND*	ND*	ND*	ND*	ND*	ND*	Hit	Optimization of HTS hit A0	[127]
542	24	16.33 ± 1.47 µM	ND*	Enzymatic assay	ND*	ND*	ND*	ND*	ND*	ND*	Hit	Optimization of HTS hit A0	[127]
543	25	4.82 ± 0.22 µM	ND*	Enzymatic assay	ND*	ND*	ND*	ND*	ND*	ND*	Hit	Optimization of HTS hit A0	[127]
544	26	5.09 ± 0.49 µM	ND*	Enzymatic assay	ND*	ND*	ND*	ND*	ND*	ND*	Hit	Optimization of HTS hit A0	[127]
545	27	8.77 ± 0.08 µM	ND*	Enzymatic assay	ND*	ND*	ND*	ND*	ND*	ND*	Hit	Optimization of HTS hit A0	[127]
546	28	0.144 ± 0.018 µM	ND*	Enzymatic assay	12.44 ± 2.19 µM	Cell-based assay (FlipGFP)	30.67 ± 6.63 µM	ND*	ND*	ND*	Hit	Optimization of HTS hit A0	[127]
547	29	0.141 ± 0.054 µM	ND*	Enzymatic assay	8.00 ± 2.00 µM	Cell-based assay (FlipGFP)	32.51 ± 5.41 µM	ND*	ND*	ND*	Hit	Optimization of HTS hit A0	[127]
548	30	0.342 ± 0.224 µM	ND*	Enzymatic assay	10.59 ± 3.46 µM	Cell-based assay (FlipGFP)	>40 µM	ND*	ND*	ND*	Hit	Optimization of HTS hit A0	[127]
549	31	0.672 ± 0.224 µM	ND*	Enzymatic assay	ND*	ND*	ND*	ND*	ND*	ND*	Hit	Optimization of HTS hit A0	[127]
550	32	0.275 ± 0.058 µM	ND*	Enzymatic assay	10.8 ± 1.85 µM	Cell-based assay (FlipGFP)	33.47 ± 1.60 µM	ND*	ND*	ND*	Hit	Optimization of HTS hit A0	[127]
551	33	0.231 ± 0.019 µM	ND*	Enzymatic assay	8.79 ± 2.19 µM	Cell-based assay (FlipGFP)	30.67 ± 6.63 µM	ND*	ND*	ND*	Hit	Optimization of HTS hit A0	[127]
552	34	0.436 ± 0.036 µM	ND*	Enzymatic assay	ND*	ND*	ND*	ND*	ND*	ND*	Hit	Optimization of HTS hit A0	[127]
553	35	1.86 ± 0.68 µM	ND*	Enzymatic assay	ND*	ND*	ND*	ND*	ND*	ND*	Hit	Optimization of HTS hit A0	[127]
554	36	1.40 ± 0.09 µM	ND*	Enzymatic assay	ND*	ND*	ND*	ND*	ND*	ND*	Hit	Optimization of HTS hit A0	[127]

555	37	0.095 ± 0.034 µM	ND*	Enzymatic assay	4.22 ± 0.89 µM	Cell-based assay (FlipGFP)	17.76 ± 0.68 µM	ND*	ND*	ND*	Hit	Optimization of HTS hit A0	[127]
556	38	0.074 ± 9.025 µM	ND*	Enzymatic assay	4.30 ± 1.21 µM	Cell-based assay (FlipGFP)	25.30 ± 9.87 µM	ND*	ND*	ND*	Hit	Optimization of HTS hit A0	[127]
557	39	0.078 ± 0.014 µM	ND*	Enzymatic assay	2.41 ± 0.12 µM	Cell-based assay (FlipGFP)	12.91 ± 3.55 µM	105 mL/min/gprot	ND*	ND*	Experimental	Optimization of HTS hit A0	[127]
558	(S)-39	0.035 ± 0.006 µM	ND*	Enzymatic assay	ND*	ND*	ND*	ND*	ND*	ND*	Hit	Optimization of HTS hit A0	[127]
559	(R)-39	4.266 ± 0.013 µM	ND*	Enzymatic assay	ND*	ND*	ND*	ND*	ND*	ND*	Hit	Optimization of HTS hit A0	[127]
560	40	0.169 ± 0.035 µM	ND*	Enzymatic assay	6.09 ± 0.45 µM	Cell-based assay (FlipGFP)	>40 µM	ND*	ND*	ND*	Hit	Optimization of HTS hit A0	[127]
561	41	0.523 ± 0.147 µM	ND*	Enzymatic assay	6.64 ± 1.73 µM	Cell-based assay (FlipGFP)	>40 µM	ND*	ND*	ND*	Hit	Optimization of HTS hit A0	[127]
562	42	0.070 ± 0.025µM	ND*	Enzymatic assay	4.11 ± 1.82 µM	Cell-based assay (FlipGFP)	18.79 ± 0.71 µM	ND*	ND*	ND*	Hit	Optimization of HTS hit A0	[127]
563	43	0.044 ± 0.007 µM	ND*	Enzymatic assay	2.33 ± 0.33 µM	Cell-based assay (FlipGFP)	9.40 ± 0.54 µM	ND*	ND*	ND*	Hit	Optimization of HTS hit A0	[127]
564	44	0.052 ± 0.008 µM	ND*	Enzymatic assay	3.81 ± 0.59 µM	Cell-based assay (FlipGFP)	11.64 ± 0.35 µM	ND*	ND*	ND*	Hit	Optimization of HTS hit A0	[127]
565	45	0.246 ± 0.044 µM	ND*	Enzymatic assay	3.13 ± 0.75 µM	Cell-based assa	>40 µM	2503 mL/min/gprot	ND*	ND*	Experimental	Optimization of HTS hit A0	[127]

						y (Flip GFP)							
566	(S)-45	0.097 ± 0.013 µM	N D*	Enzymatic assay	ND*	ND*	ND*	ND*	ND*	ND*	Hit	Optimization of HTS hit A0	[127]
567	(R)-45	>20 µM	N D*	Enzymatic assay	ND*	ND*	ND*	ND*	ND*	ND*	Hit	Optimization of HTS hit A0	[127]
568	46	0.511 ± 0.090 µM	N D*	Enzymatic assay	5.33 ± 1.54 µM	Cell-based assay (Flip GFP)	>40	ND*	ND*	ND*	Hit	Optimization of HTS hit A0	[127]
569	47	0.594 ± 0.108 µM	N D*	Enzymatic assay	5.52 ± 0.77 µM	Cell-based assay (Flip GFP)	>40	ND*	ND*	ND*	Hit	Optimization of HTS hit A0	[127]
570	48	0.714 ± 0.128 µM	N D*	Enzymatic assay	ND*	ND*	ND*	ND*	ND*	ND*	Hit	Optimization of HTS hit A0	[127]
571	49	1.482 ± 0.234 µM	N D*	Enzymatic assay	ND*	ND*	ND*	ND*	ND*	ND*	Hit	Optimization of HTS hit A0	[127]
572	50	1.355 ± 0.230 µM	N D*	Enzymatic assay	ND*	ND*	ND*	ND*	ND*	ND*	Hit	Optimization of HTS hit A0	[127]
573	51	1.741 ± 0.545 µM	N D*	Enzymatic assay	ND*	ND*	ND*	ND*	ND*	ND*	Hit	Optimization of HTS hit A0	[127]
574	Jun9-53-1	2.71 µM	N N	FRET assay	ND*	Vero-E6	32.18 µM	ND*	ND*	ND*	Hit	QSAR of GRL-0617	[128]
575	Jun9-53-2	0.89 µM	N D*	FRET assay	25.19 µM	Vero-E6	25.70 ± 0.46 µM	ND*	ND*	ND*	Hit	QSAR of GRL-0617	[128]
576	Jun9-44-3	0.76 µM	N D*	FRET assay	11.23 ± 6.72 µM	Calu-3/Vero-E6	27.71 ± 1.05 µM	ND*	ND*	ND*	Hit	QSAR of GRL-0617	[128]
577	Jun9-28-6	2.66 µM	N D*	FRET assay	5.17 µM	Huh7-ACE2 cells	ND*	ND*	ND*	ND*	Hit	QSAR of GRL-0617	[128]
578	Jun9-26-5	>20 µM	N D*	FRET assay	ND*	ND*	ND*	ND*	ND*	ND*	Hit	QSAR of GRL-0617	[128]
579	Jun9-25-5 (Jun9-13-9)	4.98 µM	N D*	FRET assay	3.10 µM	Huh7-ACE2/Vero E6	38.76 µM	ND*	ND*	ND*	Hit	QSAR of GRL-0617	[128]

580	Jun9-26-2	>20 µM	ND*	FRET assay	ND*	ND*	ND*	ND*	ND*	ND*	Hit	QSAR of GRL-0617	[128]
581	Jun9-13-7	7.29 µM	ND*	FRET assay	ND*	Vero E6	23.27 µM	ND*	ND*	ND*	Hit	QSAR of GRL-0617	[128]
582	Jun9-29-1	>20 µM	ND*	FRET assay	ND*	ND*	ND*	ND*	ND*	ND*	Hit	QSAR of GRL-0617	[128]
583	Jun9-13-8	71.8 µM	ND*	FRET assay	ND*	ND*	ND*	ND*	ND*	ND*	Hit	QSAR of GRL-0617	[128]
584	Jun9-13-4	>20 µM	ND*	FRET assay	ND*	ND*	ND*	ND*	ND*	ND*	Hit	QSAR of GRL-0617	[128]
585	Jun9-13-6	>20 µM	ND*	FRET assay	ND*	ND*	ND*	ND*	ND*	ND*	Hit	QSAR of GRL-0617	[128]
586	Jun9-26-5	>20 µM	ND*	FRET assay	ND*	ND*	ND*	ND*	ND*	ND*	Hit	QSAR of GRL-0617	[128]
587	Jun9-29-6	26.9 µM	ND*	FRET assay	ND*	ND*	ND*	ND*	ND*	ND*	Hit	QSAR of GRL-0617	[128]
588	Jun9-29-7	42.9 µM	ND*	FRET assay	ND*	ND*	ND*	ND*	ND*	ND*	Hit	QSAR of GRL-0617	[128]
589	Jun9-14-5	>20 µM	ND*	FRET assay	ND*	ND*	ND*	ND*	ND*	ND*	Hit	QSAR of GRL-0617	[128]
590	Jun9-29-3	>20 µM	ND*	FRET assay	ND*	ND*	ND*	ND*	ND*	ND*	Hit	QSAR of GRL-0617	[128]
591	Jun9-25-4	49.3 µM	ND*	FRET assay	ND*	ND*	ND*	ND*	ND*	ND*	Hit	QSAR of GRL-0617	[128]
592	Jun9-26-2	>20 µM	ND*	FRET assay	ND*	ND*	ND*	ND*	ND*	ND*	Hit	QSAR of GRL-0617	[128]

593	Jun9-29-4	>20 μM	N D*	FRET assay	ND*	ND*	ND*	ND*	ND*	ND*	Hit	QSAR of GRL-0617	[128]
594	Jun9-47-2	6.72 μM	N D*	FRET assay	ND*	Vero E6	62.08 ± 2.53 μM	ND*	ND*	ND*	Hit	QSAR of GRL-0617	[128]
595	Jun9-47-3	2.44 μM	N D*	FRET assay	ND*	Vero E6	118.6 ± 2.14 μM	ND*	ND*	ND*	Hit	QSAR of GRL-0617	[128]
596	Jun9-87-1	0.87 μM	N D*	FRET assay	ND*	ND*	ND*	ND*	ND*	ND*	Hit	QSAR of GRL-0617	[128]
597	Jun9-87-2	0.90 μM	N D*	FRET assay	ND*	ND*	ND*	ND*	ND*	ND*	Hit	QSAR of GRL-0617	[128]
598	Jun9-87-3	0.80 μM	N D*	FRET assay	ND*	ND*	ND*	ND*	ND*	ND*	Hit	QSAR of GRL-0617	[128]
599	Jun9-81-2	1.44 μM	N D*	FRET assay	ND*	ND*	ND*	ND*	ND*	ND*	Hit	QSAR of GRL-0617	[128]
600	Jun9-81-3	1.85 μM	N D*	FRET assay	ND*	ND*	ND*	ND*	ND*	ND*	Hit	QSAR of GRL-0617	[128]
601	Jun9-81-1	19.23 μM	N D*	FRET assay	ND*	ND*	ND*	ND*	ND*	ND*	Hit	QSAR of GRL-0617	[128]
602	Jun9-75-5	0.56 μM	N D*	FRET assay	ND*	ND*	ND*	ND*	ND*	ND*	Experi mental	QSAR of GRL-0617	[128]
603	Jun9-75-3	8.89 μM	N D*	FRET assay	ND*	ND*	ND*	ND*	ND*	ND*	Hit	QSAR of GRL-0617	[128]
604	Jun9-75-2	1.54 μM	N D*	FRET assay	ND*	ND*	ND*	ND*	ND*	ND*	Hit	QSAR of GRL-0617	[128]
605	Jun9-87-1	0.87 μM	N D*	FRET assay	ND*	ND*	ND*	ND*	ND*	ND*	Hit	QSAR of GRL-0617	[128]

606	Jun9-87-2	0.90 µM	ND*	FRET assay	ND*	ND*	ND*	ND*	ND*	ND*	Hit	QSAR of GRL-0617	[128]
607	Jun9-87-3	0.80 µM	ND*	FRET assay	ND*	ND*	ND*	ND*	ND*	ND*	Hit	QSAR of GRL-0617	[128]
608	Jun9-81-2	1.44 µM	ND*	FRET assay	ND*	ND*	ND*	ND*	ND*	ND*	Hit	QSAR of GRL-0617	[128]
609	Jun9-81-3	1.85 µM	ND*	FRET assay	ND*	ND*	ND*	ND*	ND*	ND*	Hit	QSAR of GRL-0617	[128]
610	Jun9-81-1	19.23 µM	ND*	FRET assay	ND*	ND*	ND*	ND*	ND*	ND*	Hit	QSAR of GRL-0617	[128]
611	Jun9-75-5	0.56 µM	ND*	FRET assay	ND*	ND*	ND*	ND*	ND*	ND*	Hit	QSAR of GRL-0617	[128]
612	ZX-136-2	9.29 µM	ND*	FRET assay	ND*	ND*	ND*	ND*	ND*	ND*	Hit	QSAR of GRL-0617	[128]
613	ZX-136-3	10.11 µM	ND*	FRET assay	ND*	ND*	ND*	ND*	ND*	ND*	Hit	QSAR of GRL-0617	[128]
614	ZX-136-4	>20 µM	ND*	FRET assay	ND*	ND*	ND*	ND*	ND*	ND*	Hit	QSAR of GRL-0617	[128]
615	ZX-136-5	8.32 µM	ND*	FRET assay	ND*	ND*	ND*	ND*	ND*	ND*	Hit	QSAR of GRL-0617	[128]
616	ZX-136-6	11.71 µM	ND*	FRET assay	ND*	ND*	ND*	ND*	ND*	ND*	Hit	QSAR of GRL-0617	[128]
617	ZX-136-7	1.30 µM	ND*	FRET assay	ND*	ND*	ND*	ND*	ND*	ND*	Hit	QSAR of GRL-0617	[128]
618	Jun9-80-4	1.58 µM	ND*	FRET assay	ND*	ND*	ND*	ND*	ND*	ND*	Hit	QSAR of GRL-0617	[128]

619	ZX-136-8	5.13 µM	ND*	FRET assay	ND*	ND*	ND*	ND*	ND*	ND*	Hit	QSAR of GRL-0617	[128]
620	ZX-136-9	>20 µM	ND*	FRET assay	ND*	ND*	ND*	ND*	ND*	ND*	Hit	QSAR of GRL-0617	[128]
621	ZX-136-10	14.35 µM	ND*	FRET assay	ND*	ND*	ND*	ND*	ND*	ND*	Hit	QSAR of GRL-0617	[128]
622	ZX-128	2.72 µM	ND*	FRET assay	ND*	ND*	ND*	ND*	ND*	ND*	Hit	QSAR of GRL-0617	[128]
623	ZX-129	>20 µM	ND*	FRET assay	ND*	ND*	ND*	ND*	ND*	ND*	Hit	QSAR of GRL-0617	[128]
624	Jun9-75-4	0.62 µM	ND*	FRET assay	ND*	ND*	ND*	ND*	ND*	ND*	Hit	QSAR of GRL-0617	[128]
625	Jun9-84-2	2.47 µM	ND*	FRET assay	ND*	ND*	ND*	ND*	ND*	ND*	Hit	QSAR of GRL-0617	[128]
626	Jun9-84-3	0.67 µM	ND*	FRET assay	ND*	ND*	ND*	ND*	ND*	ND*	Hit	QSAR of GRL-0617	[128]
627	Jun9-84-4	1.30 µM	ND*	FRET assay	ND*	ND*	ND*	ND*	ND*	ND*	Hit	QSAR of GRL-0617	[128]
628	Jun9-84-5	>20 µM	ND*	FRET assay	ND*	ND*	ND*	ND*	ND*	ND*	Hit	QSAR of GRL-0617	[128]
629	Jun9-84-6	>20 µM	ND*	FRET assay	ND*	ND*	ND*	ND*	ND*	ND*	Hit	QSAR of GRL-0617	[128]
630	Jun9-84-7	5.62 µM	ND*	FRET assay	ND*	ND*	ND*	ND*	ND*	ND*	Hit	QSAR of GRL-0617	[128]
631	Jun9-85-1	0.66 µM	ND*	FRET assay	ND*	ND*	ND*	ND*	ND*	ND*	Hit	QSAR of GRL-0617	[128]

632	Jun9-85-2	1.15 µM	ND*	FRET assay	ND*	ND*	ND*	ND*	ND*	ND*	Hit	QSAR of GRL-0617	[128]
633	Jun9-85-4	2.84 µM	ND*	FRET assay	ND*	ND*	ND*	ND*	ND*	ND*	Hit	QSAR of GRL-0617	[128]
634	Jun9-85-5	16.59 µM	ND*	FRET assay	ND*	ND*	ND*	ND*	ND*	ND*	Hit	QSAR of GRL-0617	[128]
635	Jun9-85-6	4.42 µM	ND*	FRET assay	ND*	ND*	ND*	ND*	ND*	ND*	Hit	QSAR of GRL-0617	[128]
636	Jun9-85-7	6.41 µM	ND*	FRET assay	ND*	ND*	ND*	ND*	ND*	ND*	Hit	QSAR of GRL-0617	[128]
637	Jun9-85-8	>20 µM	ND*	FRET assay	ND*	ND*	ND*	ND*	ND*	ND*	Hit	QSAR of GRL-0617	[128]
638	Jun9-86-1	3.99 µM	ND*	FRET assay	ND*	ND*	ND*	ND*	ND*	ND*	Hit	QSAR of GRL-0617	[128]
639	Jun9-86-2	6.49 µM	ND*	FRET assay	ND*	ND*	ND*	ND*	ND*	ND*	Hit	QSAR of GRL-0617	[128]
640	Jun9-86-3	7.01 µM	ND*	FRET assay	ND*	ND*	ND*	ND*	ND*	ND*	Hit	QSAR of GRL-0617	[128]
641	Jun9-86-4	9.22 µM	ND*	FRET assay	ND*	ND*	ND*	ND*	ND*	ND*	Hit	QSAR of GRL-0617	[128]
642	Jun9-86-5	6.12 µM	ND*	FRET assay	ND*	ND*	ND*	ND*	ND*	ND*	Hit	QSAR of GRL-0617	[128]
643	Jun9-86-6	0.37 µM	ND*	FRET assay	ND*	ND*	ND*	ND*	ND*	ND*	Hit	QSAR of GRL-0617	[128]
644	Jun9-86-7	>20 µM	ND*	FRET assay	ND*	ND*	ND*	ND*	ND*	ND*	Hit	QSAR of GRL-0617	[128]

645	Jun9-86-8	5.50 µM	ND*	FRET assay	ND*	ND*	ND*	ND*	ND*	ND*	Hit	QSAR of GRL-0617	[128]
646	Jun9-87-1	0.87 µM	ND*	FRET assay	ND*	Vero-E6	14.43 ± 0.77 µM	ND*	ND*	ND*	Hit	QSAR of GRL-0617	[128]
647	Jun9-87-2	0.90 µM	ND*	FRET assay	ND*	Vero-E6	60.96 ± 6.79 µM	ND*	ND*	ND*	Hit	QSAR of GRL-0617	[128]
648	Jun9-87-3	0.80 µM	ND*	FRET assay	ND*	Vero-E6	25.97 ± 2.99 µM	ND*	ND*	ND*	Hit	QSAR of GRL-0617	[128]
649	Jun 10-69-2	3.99 ± 0.74 µM	ND*	FRET assay	ND*	ND*	ND*	ND*	ND*	ND*	Hit	QSAR of GRL-0617	[128]
650	VIR251	ND*	ND*	Enzymatic assay	~5 µM	ND*	ND*	ND*	ND*	ND*	Hit	Covalent peptidomimetics	[129]
651	Compound 1	ND*	ND*	Enzymatic assay	2.69 ± 0.34 µM	ND*	ND*	ND*	ND*	ND*	Hit	Covalent peptidomimetics	[129]
652	Compound 2	0.39 µM	ND*	Enzymatic assay	3.61 ± 0.52 µM	Flip GFP assay/CCk8 assay	~179.9 µM	ND*	ND*	ND*	Experimental	Covalent peptidomimetics	[129]
653	Compound 3	ND*	ND*	Enzymatic assay	2.97 ± 0.16 µM	Flip GFP assay/CCk8 assay	58.32 ± 22.11 µM	ND*	ND*	ND*	Hit	Covalent peptidomimetics	[129]
654	Compound 4	ND*	ND*	Enzymatic assay	>200 µM	Flip GFP assay/CCk8 assay	>200 µM	ND*	ND*	ND*	Hit	Covalent peptidomimetics	[129]
655	Compound 6	5.0 ± 1.9 µM	ND*	Enzymatic assay	21.0 ± 1.9 µM	Vero-E6	ND*	ND*	ND*	ND*	Experimental	Covalent peptidomimetics	[129]
656	7724772	23.5 ± 1.7 µM	ND*	Enzymatic assay	ND*	ND*	ND*	ND*	ND*	ND*	Hit	Covalent peptidomimetics	[113]
657	6577871	100.7 ± 4.76 µM	ND*	Enzymatic assay	ND*	ND*	ND*	ND*	ND*	ND*	Hit	Covalent peptidomimetics	[113]
658	9247873	>200 µM	ND*	Enzymatic assay	ND*	ND*	ND*	ND*	ND*	ND*	Hit	Covalent peptidomimetics	[113]
659	GNZL-P1	2.8310 µM	ND*	Enzymatic assay	µM	ND*	ND*	ND*	ND*	ND*	Hit	GRL-0617 analogue	[130]

660	GNZ L-P2	6.9000 µM	N D*	Enzym atic assay	µM	ND*	ND*	ND*	ND*	ND*	Hit	GRL-0617 analogue	[130]
661	GNZ L-P3	0.1858 µM	N D*	Enzym atic assay	µM	ND*	ND*	ND*	ND*	ND*	Hit	GRL-0617 analogue	[130]
662	GNZ L-P4	0.0363 µM	Ve ro E6	Enzym atic assay	µM	ND*	24.57 µM	ND*	ND*	ND*	Hit	GRL-0617 analogue	[130]
663	GNZ L-P5	0.0210 µM	N D*	Enzym atic assay	µM	ND*	ND*	ND*	ND*	ND*	Hit	GRL-0617 analogue	[130]
664	GNZ L-P13	0.0900 µM	N D*	Enzym atic assay	µM	ND*	ND*	ND*	ND*	ND*	Hit	GRL-0617 analogue	[130]
665	GNZ L-P17	0.00291 µM	Ve ro E6	Enzym atic assay	µM	ND*	36.68 µM	ND*	ND*	ND*	Hit	GRL-0617 analogue	[130]
666	GNZ L-P20	0.1150 µM	N D*	Enzym atic assay	µM	ND*	ND*	ND*	ND*	ND*	Hit	GRL-0617 analogue	[130]
667	GNZ L-P25	0.4700 µM	N D*	Enzym atic assay	µM	ND*	ND*	ND*	ND*	ND*	Hit	GRL-0617 analogue	[130]
668	GNZ L-P28	0.01464 µM	N D*	Enzym atic assay	µM	ND*	ND*	ND*	ND*	ND*	Hit	GRL-0617 analogue	[130]
669	GNZ L-P29	0.0420 µM	Ve ro E6	Enzym atic assay	µM	ND*	12.23 µM	ND*	ND*	ND*	Hit	GRL-0617 analogue	[130]
670	GNZ L-P30	0.0440 µM	N D*	Enzym atic assay	µM	ND*	ND*	ND*	ND*	ND*	Hit	GRL-0617 analogue	[130]
671	GNZ L-P31	0.00846 µM	Ve ro E6	Enzym atic assay	µM	ND*	157.4 µM	ND*	ND*	ND*	Hit	GRL-0617 analogue	[130]
672	GNZ L-P35	0.00815 µM	Ve ro E6	Enzym atic assay	µM	ND*	67.67 µM	ND*	ND*	ND*	Hit	GRL-0617 analogue	[130]
673	GNZ L-P36	0.00645 µM	Ve ro E6	Enzym atic assay	111. 0 ± 9.95 nM	ND*	888.41 µM	ND*	ND*	ND*	Experi mental	GRL-0617 analogue	[130]

674	S-217622	ND*	ND*	ND*	171.9 ± 8.65 nM	ND*	ND*	ND*	ND*	ND*	Lead	GRL-0617 analogue	[130]
675	WEHI-P1	2.6 µM	ND*	Enzymatic assay	>20 µM	FRET assay	ND*	ND*	ND*	ND*	Hit	Methoxynaphtalene containing	[144]
676	WEHI-P2	397 nM	ND*	Enzymatic assay	11 µM	FRET assay	ND*	ND*	ND*	ND*	Hit	Methoxynaphtalene containing	[144]
677	WEHI-P4	19 nM	ND*	Enzymatic assay	0.31 µM	FRET assay	ND*	ND*	ND*	ND*	Hit	Methoxynaphtalene containing	[144]
678	WEHI-P8	12 nM	ND*	Enzymatic assay	0.36 µM	FRET assay	ND*	ND*	16.8	ND*	Experimental	Methoxynaphtalene containing	[144]
679	WEHI-P70	98 nM	ND*	Enzymatic assay	298 nM	FRET assay	ND*	ND*	ND*	ND*	Hit	Methoxynaphtalene containing	[144]
680	WEHI-P24		ND*	Enzymatic assay	ND*	ND*	ND*	ND*	ND*	ND*	Hit	Methoxynaphtalene containing	[144]
681	Sennoside A	7.01 µM	ND*	Enzymatic assay	ND*	ND*	ND*	ND*	ND*	ND*	Hit	Biochemical HTS screening	[131]
682	Semapimod (EOS)	1.21 µM	ND*	Enzymatic assay	ND*	ND*	ND*	ND*	ND*	ND*	Hit	Biochemical HTS screening	[131]
683	Semapimod (FhG)	5.23 µM	ND*	Enzymatic assay	ND*	ND*	ND*	ND*	ND*	ND*	Hit	Biochemical HTS screening	[131]
684	SRT 1720	>20 µM	ND*	Enzymatic assay	ND*	ND*	ND*	ND*	ND*	ND*	Hit	Biochemical HTS screening	[131]
685	PR-619	1.39 µM	ND*	Enzymatic assay	ND*	ND*	ND*	ND*	ND*	ND*	Lead	Biochemical HTS screening	[131]
686	CPI-169 (FhG)	14.17 µM	ND*	Enzymatic assay	ND*	ND*	ND*	ND*	ND*	ND*	Hit	Biochemical HTS screening	[131]
687	CPI-169 (EOS)	19.8 µM	ND*	Enzymatic assay	ND*	ND*	ND*	ND*	ND*	ND*	Hit	Biochemical HTS screening	[131]
688	DOM_SIM_710	>20 µM	ND*	Enzymatic assay	ND*	ND*	ND*	ND*	ND*	ND*	Hit	Biochemical HTS screening	[131]
689	Purpurogallin	>20 µM	ND*	Enzymatic assay	ND*	ND*	ND*	ND*	ND*	ND*	Hit	Biochemical HTS screening	[131]

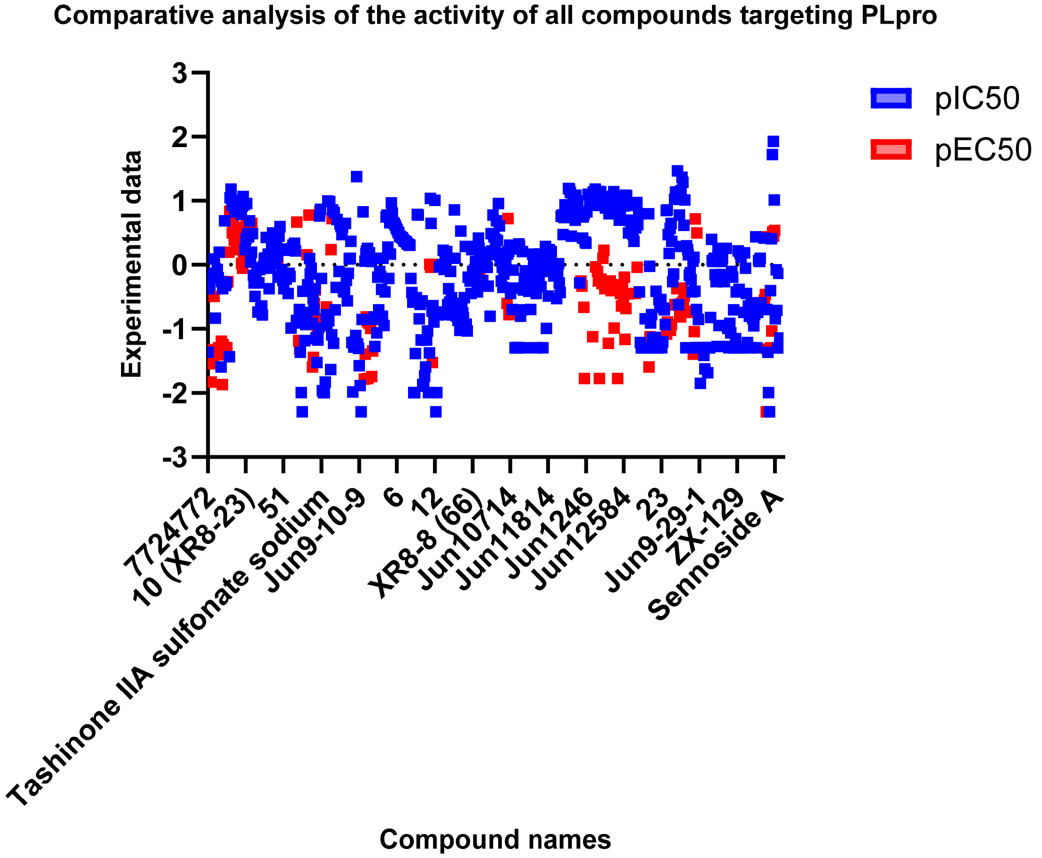

Figure S2. Comparative analysis of activity of all reported compounds targeting PLpro.

References

1. Bojadzic, D., et al., *Small-molecule inhibitors of the coronavirus spike: ACE2 protein–protein interaction as blockers of viral attachment and entry for SARS-CoV-2.* ACS infectious diseases, 2021. **7**(6): p. 1519-1534.
2. Bastos, R.S., et al., *Design and identification of inhibitors for the spike-ACE2 target of SARS-CoV-2.* International Journal of Molecular Sciences, 2023. **24**(10): p. 8814.
3. David, A.B., et al., *Identification of SARS-CoV-2 receptor binding inhibitors by in vitro screening of drug libraries.* Molecules, 2021. **26**(11): p. 3213.
4. Shahhamzehei, N., et al., *Identification of nitrile-containing isoquinoline-related natural product derivatives as coronavirus entry inhibitors in silico and in vitro.* Biomedicine & Pharmacotherapy, 2024. **180**: p. 117517.
5. Shin, Y.H., et al., *Inhibition of ACE2-Spike Interaction by an ACE2 Binder Suppresses SARS-CoV-2 Entry.* Angewandte Chemie International Edition, 2022. **61**(11): p. e202115695.
6. Munawar, M., et al., *Design and Evaluation of ATN-161 Analogues as Fusion Inhibitors Targeting Integrins in SARS-CoV-2.* International Journal of Peptide Research and Therapeutics, 2025. **31**(4): p. 1-25.

7. Wang, L., et al., *Discovery of potential small molecular SARS-CoV-2 entry blockers targeting the spike protein.* Acta Pharmacologica Sinica, 2022. **43**(4): p. 788-796.
8. Yuan, S., et al., *Clofazimine broadly inhibits coronaviruses including SARS-CoV-2.* Nature, 2021. **593**(7859): p. 418-423.
9. Li, Z., et al., *A vimentin-targeting oral compound with host-directed antiviral and anti-inflammatory actions addresses multiple features of COVID-19 and related diseases.* MBio, 2021. **12**(5): p. 10.1128/mbio. 02542-21.
10. Chen, Y., et al., *Sertraline is an effective SARS-CoV-2 entry inhibitor targeting the spike protein.* Journal of Virology, 2022. **96**(24): p. e01245-22.
11. Ratnapriya, S., et al., *Broad tricyclic ring inhibitors block SARS-CoV-2 spike function required for viral entry.* ACS infectious diseases, 2022. **8**(10): p. 2045-2058.
12. Zhang, X., et al., *Clofazimine derivatives as potent broad-spectrum antiviral agents with dual-target mechanism.* European journal of medicinal chemistry, 2022. **234**: p. 114209.
13. Acharya, A., et al., *Discovery and evaluation of entry inhibitors for SARS-CoV-2 and its emerging variants.* Journal of virology, 2021. **95**(24): p. 10.1128/jvi. 01437-21.
14. Shi, Y., et al., *Thiol-based chemical probes exhibit antiviral activity against SARS-CoV-2 via allosteric disulfide disruption in the spike glycoprotein.* Proceedings of the National Academy of Sciences, 2022. **119**(6): p. e2120419119.
15. Pringle, E.S., et al., *Thiopurines inhibit coronavirus Spike protein processing and incorporation into progeny virions.* PLoS Pathogens, 2022. **18**(9): p. e1010832.
16. Rodriguez, Y., et al., *Small molecules targeting SARS-CoV-2 spike glycoprotein receptor-binding domain.* ACS omega, 2022. **7**(33): p. 28779-28789.
17. Park, S.B., et al., *Targeting the fusion process of SARS-CoV-2 infection by small molecule inhibitors.* MBio, 2022. **13**(1): p. e03238-21.
18. Tietjen, I., et al., *The natural stilbenoid (–)-hopeaphenol inhibits cellular entry of SARS-CoV-2 USA-WA1/2020, B. 1.1. 7, and B. 1.351 variants.* Antimicrobial Agents and Chemotherapy, 2021. **65**(12): p. 10.1128/aac. 00772-21.
19. Taha, Z., et al., *Identification of FDA-approved bifonazole as a SARS-CoV-2 blocking agent following a bioreporter drug screen.* Molecular Therapy, 2022. **30**(9): p. 2998-3016.
20. Oh, C.-k., et al., *Targeted protein S-nitrosylation of ACE2 inhibits SARS-CoV-2 infection.* Nature chemical biology, 2023. **19**(3): p. 275-283.
21. Yang, J., et al., *Fluorogenic reporter enables identification of compounds that inhibit SARS-CoV-2.* Nature microbiology, 2023. **8**(1): p. 121-134.
22. Gkekas, I., et al., *AI Promoted Virtual Screening, Structure-Based Hit Optimization, and Synthesis of Novel COVID-19 S-RBD Domain Inhibitors.* Journal of Chemical Information and Modeling, 2024. **64**(22): p. 8562-8585.
23. Shapira, T., et al., *A TMPRSS2 inhibitor acts as a pan-SARS-CoV-2 prophylactic and therapeutic.* Nature, 2022. **605**(7909): p. 340-348.

24. Mahoney, M., et al., *A novel class of TMPRSS2 inhibitors potently block SARS-CoV-2 and MERS-CoV viral entry and protect human epithelial lung cells.* Proceedings of the National Academy of Sciences, 2021. **118**(43): p. e2108728118.
25. Ferková, S., et al., *Optimizing the Pharmacokinetics and Selectivity of TMPRSS2 Inhibitors.* European Journal of Medicinal Chemistry, 2025: p. 117579.
26. Hoffmann, M., et al., *Nafamostat mesylate blocks activation of SARS-CoV-2: new treatment option for COVID-19.* Antimicrobial agents and chemotherapy, 2020. **64**(6): p. 10.1128/aac. 00754-20.
27. Fraser, B.J., et al., *Structure, activity and inhibition of human TMPRSS2, a protease implicated in SARS-CoV-2 activation.* BioRxiv, 2021: p. 2021.06. 23.449282.
28. Wettstein, L., et al., *Peptidomimetic inhibitors of TMPRSS2 block SARS-CoV-2 infection in cell culture.* Communications biology, 2022. **5**(1): p. 681.
29. Chen, Y., et al., *A high-throughput screen for TMPRSS2 expression identifies FDA-approved compounds that can limit SARS-CoV-2 entry.* Nature communications, 2021. **12**(1): p. 3907.
30. Sun, Q., et al., *Bardoxolone and bardoxolone methyl, two Nrf2 activators in clinical trials, inhibit SARS-CoV-2 replication and its 3C-like protease.* Signal transduction and targeted therapy, 2021. **6**(1): p. 212.
31. Hempel, T., et al., *Synergistic inhibition of SARS-CoV-2 cell entry by otamixaban and covalent protease inhibitors: pre-clinical assessment of pharmacological and molecular properties.* Chemical science, 2021. **12**(38): p. 12600-12609.
32. Chen, Y., et al., *Tafenoquine and its derivatives as inhibitors for the severe acute respiratory syndrome coronavirus 2.* Journal of Biological Chemistry, 2022. **298**(3): p. 101658.
33. Ma, X.R., et al., *MPI8 is potent against SARS-CoV-2 by inhibiting dually and selectively the SARS-CoV-2 main protease and the host cathepsin L.* ChemMedChem, 2022. **17**(1): p. e202100456.
34. Meewan, I., et al., *Discovery of triple inhibitors of both SARS-CoV-2 proteases and human cathepsin L.* Pharmaceuticals, 2022. **15**(6): p. 744.
35. Sacco, M.D., et al., *Structure and inhibition of the SARS-CoV-2 main protease reveal strategy for developing dual inhibitors against Mpro and cathepsin L.* Science Advances, 2020. **6**(50): p. eabe0751.
36. Zhao, M.-M., et al., *Novel cleavage sites identified in SARS-CoV-2 spike protein reveal mechanism for cathepsin L-facilitated viral infection and treatment strategies.* Cell discovery, 2022. **8**(1): p. 53.
37. Mondal, S., et al., *Dual inhibitors of main protease (MPro) and Cathepsin L as potent antivirals against SARS-CoV2.* Journal of the American Chemical Society, 2022. **144**(46): p. 21035-21045.
38. Mao, B., et al., *Obatoclax inhibits SARS-CoV-2 entry by altered endosomal acidification and impaired cathepsin and furin activity in vitro.* Emerging Microbes & Infections, 2022. **11**(1): p. 483-497.

39. Mellott, D.M., et al., *A clinical-stage cysteine protease inhibitor blocks SARS-CoV-2 infection of human and monkey cells.* ACS chemical biology, 2021. **16**(4): p. 642-650.
40. Jin, Z., et al., *Structure of Mpro from SARS-CoV-2 and discovery of its inhibitors.* Nature, 2020. **582**(7811): p. 289-293.
41. Ahmed, M.S., et al., *FDA approved drugs with antiviral activity against SARS-CoV-2: From structure-based repurposing to host-specific mechanisms.* Biomedicine & Pharmacotherapy, 2023. **162**: p. 114614.
42. Allerton, C.M., et al., *A second-generation oral SARS-CoV-2 main protease inhibitor clinical candidate for the treatment of COVID-19.* Journal of Medicinal Chemistry, 2024.
43. Dayan Elshan, N., et al., *Discovery of CMX990: A Potent SARS-CoV-2 3CL Protease Inhibitor Bearing a Novel Warhead.* Journal of medicinal chemistry, 2024. **67**(4): p. 2369-2378.
44. Porzberg, M.R., et al., *Peptidomimetic Phenoxymethyl Ketone Warheads as Potent Dual-Mode Inhibitors against SARS-CoV-2 Mpro and Cathepsin.* Journal of Medicinal Chemistry, 2025.
45. Previti, S., et al., *Structure-based lead optimization of peptide-based vinyl methyl ketones as SARS-CoV-2 main protease inhibitors.* European Journal of Medicinal Chemistry, 2023. **247**: p. 115021.
46. Chen, P., et al., *A Structural Comparison of Oral SARS-CoV-2 Drug Candidate Ibuzatrelvir Complexed with the Main Protease (Mpro) of SARS-CoV-2 and MERS-CoV.* JACS Au, 2024. **4**(8): p. 3217-3227.
47. Ashraf-Uz-Zaman, M., et al., *Design, synthesis, X-ray crystallography, and biological activities of covalent, non-peptidic inhibitors of SARS-CoV-2 main protease.* ACS infectious diseases, 2024. **10**(2): p. 715-731.
48. Morris, A., et al., *Discovery of SARS-CoV-2 main protease inhibitors using a synthesis-directed de novo design model.* Chemical Communications, 2021. **57**(48): p. 5909-5912.
49. Boby, M.L., et al., *Open science discovery of potent noncovalent SARS-CoV-2 main protease inhibitors.* Science, 2023. **382**(6671): p. eabo7201.
50. Chodera, J., et al., *COVID Moonshot: Open Science Discovery of SARS-CoV-2 Main Protease Inhibitors by Combining Crowdsourcing, High-Throughput Experiments, Computational Simulations, and Machine Learning.* 2020.
51. Hazemann, J., et al., *Accelerating the Hit-To-Lead Optimization of a SARS-CoV-2 Mpro Inhibitor Series by Combining High-Throughput Medicinal Chemistry and Computational Simulations.* Journal of Medicinal Chemistry, 2025.
52. Previti, S., et al., *Identification of Dual Inhibitors Targeting Main Protease (Mpro) and Cathepsin L as Potential Anti-SARS-CoV-2 Agents.* ACS Medicinal Chemistry Letters, 2024. **15**(5): p. 602-609.
53. Brier, L., et al., *Novel dithiocarbamates selectively inhibit 3CL protease of SARS-CoV-2 and other coronaviruses.* European journal of medicinal chemistry, 2023. **250**: p. 115186.

54. Qin, B., et al., *Acrylamide fragment inhibitors that induce unprecedented conformational distortions in enterovirus 71 3C and SARS-CoV-2 main protease.* Acta Pharmaceutica Sinica B, 2022. **12**(10): p. 3924-3933.
55. Yi, Y., et al., *Schaftoside inhibits 3CLpro and PLpro of SARS-CoV-2 virus and regulates immune response and inflammation of host cells for the treatment of COVID-19.* Acta Pharmaceutica Sinica B, 2022. **12**(11): p. 4154-4164.
56. Gao, S., et al., *Discovery and crystallographic studies of trisubstituted piperazine derivatives as non-covalent SARS-CoV-2 main protease inhibitors with high target specificity and low toxicity.* Journal of medicinal chemistry, 2022. **65**(19): p. 13343-13364.
57. Vatansever, E.C., et al., *Bepridil is potent against SARS-CoV-2 in vitro.* Proceedings of the National Academy of Sciences, 2021. **118**(10): p. e2012201118.
58. Pelliccia, S., et al., *Easy access to α-ketoamides as SARS-CoV-2 and MERS Mpro inhibitors via the PADAM oxidation route.* European journal of medicinal chemistry, 2022. **244**: p. 114853.
59. Fàbrega-Ferrer, M., et al., *Structure and inhibition of SARS-CoV-1 and SARS-CoV-2 main proteases by oral antiviral compound AG7404.* Antiviral research, 2022. **208**: p. 105458.
60. Kühl, N., et al., *Discovery of potent benzoxaborole inhibitors against SARS-CoV-2 main and dengue virus proteases.* European journal of medicinal chemistry, 2022. **240**: p. 114585.
61. Samrat, S.K., et al., *Allosteric inhibitors of the main protease of SARS-CoV-2.* Antiviral research, 2022. **205**: p. 105381.
62. Zhong, B., et al., *Oridonin inhibits SARS-CoV-2 by targeting its 3C-Like protease.* Small science, 2022. **2**(6): p. 2100124.
63. Ma, Y., et al., *A multi-pronged evaluation of aldehyde-based tripeptidyl main protease inhibitors as SARS-CoV-2 antivirals.* European Journal of Medicinal Chemistry, 2022. **240**: p. 114570.
64. Alugubelli, Y.R., et al., *A systematic exploration of boceprevir-based main protease inhibitors as SARS-CoV-2 antivirals.* European Journal of Medicinal Chemistry, 2022. **240**: p. 114596.
65. Fu, L., et al., *Mechanism of microbial metabolite leupeptin in the treatment of COVID-19 by traditional Chinese medicine herbs.* MBio, 2021. **12**(5): p. 10.1128/mbio. 02220-21.
66. Dou, X., et al., *Discovery of 2-(furan-2-ylmethylene) hydrazine-1-carbothioamide derivatives as novel inhibitors of SARS-CoV-2 main protease.* European Journal of Medicinal Chemistry, 2022. **238**: p. 114508.
67. Luttens, A., et al., *Ultralarge virtual screening identifies SARS-CoV-2 main protease inhibitors with broad-spectrum activity against coronaviruses.* Journal of the American Chemical Society, 2022. **144**(7): p. 2905-2920.

68. Quan, B.-X., et al., *An orally available Mpro inhibitor is effective against wild-type SARS-CoV-2 and variants including Omicron.* Nature Microbiology, 2022. **7**(5): p. 716-725.
69. Cantrelle, F.X., et al., *NMR spectroscopy of the main protease of SARS-CoV-2 and fragment-based screening identify three protein hotspots and an antiviral fragment.* Angewandte Chemie International Edition, 2021. **60**(48): p. 25428-25435.
70. Dampalla, C.S., et al., *Structure-guided design of conformationally constrained cyclohexane inhibitors of severe acute respiratory syndrome coronavirus-2 3CL protease.* Journal of medicinal chemistry, 2021. **64**(14): p. 10047-10058.
71. Wang, Z., et al., *Identification of proteasome and caspase inhibitors targeting SARS-CoV-2 Mpro.* Signal Transduction and Targeted Therapy, 2021. **6**(1): p. 214.
72. Zhang, C.-H., et al., *Potent noncovalent inhibitors of the main protease of SARS-CoV-2 from molecular sculpting of the drug perampanel guided by free energy perturbation calculations.* ACS central science, 2021. **7**(3): p. 467-475.
73. Deshmukh, M.G., et al., *Structure-guided design of a perampanel-derived pharmacophore targeting the SARS-CoV-2 main protease.* Structure, 2021. **29**(8): p. 823-833. e5.
74. Dai, W., et al., *Structure-based design of antiviral drug candidates targeting the SARS-CoV-2 main protease.* Science, 2020. **368**(6497): p. 1331-1335.
75. Qiao, J., et al., *SARS-CoV-2 M^{pro} inhibitors with antiviral activity in a transgenic mouse model.* Science, 2021. **371**(6536): p. 1374-1378.
76. Han, S.H., et al., *Structure-based optimization of ML300-derived, noncovalent inhibitors targeting the severe acute respiratory syndrome coronavirus 3CL protease (SARS-CoV-2 3CLpro).* Journal of medicinal chemistry, 2021. **65**(4): p. 2880-2904.
77. Stille, J.K., et al., *Design, synthesis and in vitro evaluation of novel SARS-CoV-2 3CLpro covalent inhibitors.* European journal of medicinal chemistry, 2022. **229**: p. 114046.
78. Shcherbakov, D., et al., *Design and evaluation of bispidine-based SARS-CoV-2 main protease inhibitors.* ACS medicinal chemistry letters, 2021. **13**(1): p. 140-147.
79. Kneller, D.W., et al., *Malleability of the SARS-CoV-2 3CL Mpro active-site cavity facilitates binding of clinical antivirals.* Structure, 2020. **28**(12): p. 1313-1320. e3.
80. Günther, S., et al., *X-ray screening identifies active site and allosteric inhibitors of SARS-CoV-2 main protease.* Science, 2021. **372**(6542): p. 642-646.
81. Chamakuri, S., et al., *DNA-encoded chemistry technology yields expedient access to SARS-CoV-2 Mpro inhibitors.* Proceedings of the National Academy of Sciences, 2021. **118**(36): p. e2111172118.
82. Du, R., et al., *Discovery of chebulagic acid and punicalagin as novel allosteric inhibitors of SARS-CoV-2 3CLpro.* Antiviral research, 2021. **190**: p. 105075.
83. Tao, X., et al., *Allosteric inhibition of SARS-CoV-2 3CL protease by colloidal bismuth subcitrate.* Chemical science, 2021. **12**(42): p. 14098-14102.

84. Iketani, S., et al., *Lead compounds for the development of SARS-CoV-2 3CL protease inhibitors.* Nature communications, 2021. **12**(1): p. 2016.
85. Rathnayake, A.D., et al., *3C-like protease inhibitors block coronavirus replication in vitro and improve survival in MERS-CoV–infected mice.* Science translational medicine, 2020. **12**(557): p. eabc5332.
86. Cooper, M.S., et al., *Diastereomeric resolution yields highly potent inhibitor of SARS-CoV-2 main protease.* Journal of Medicinal Chemistry, 2022. **65**(19): p. 13328-13342.
87. Hu, X., et al., *Structural basis for the inhibition of coronaviral main proteases by a benzothiazole-based inhibitor.* Viruses, 2022. **14**(9): p. 2075.
88. Hattori, S.-i., et al., *A small molecule compound with an indole moiety inhibits the main protease of SARS-CoV-2 and blocks virus replication.* Nature communications, 2021. **12**(1): p. 668.
89. Gurard-Levin, Z.A., et al., *Evaluation of SARS-CoV-2 3C-like protease inhibitors using self-assembled monolayer desorption ionization mass spectrometry.* Antiviral research, 2020. **182**: p. 104924.
90. Vuong, W., et al., *Feline coronavirus drug inhibits the main protease of SARS-CoV-2 and blocks virus replication.* Nature communications, 2020. **11**(1): p. 4282.
91. Amporndanai, K., et al., *Inhibition mechanism of SARS-CoV-2 main protease by ebselen and its derivatives.* Nature communications, 2021. **12**(1): p. 3061.
92. Hoffman, R.L., et al., *Discovery of ketone-based covalent inhibitors of coronavirus 3CL proteases for the potential therapeutic treatment of COVID-19.* Journal of medicinal chemistry, 2020. **63**(21): p. 12725-12747.
93. Drayman, N., et al., *Masitinib is a broad coronavirus 3CL inhibitor that blocks replication of SARS-CoV-2.* Science, 2021. **373**(6557): p. 931-936.
94. Shang, W., et al., *In vitro and in vivo evaluation of the main protease inhibitor FB2001 against SARS-CoV-2.* Antiviral research, 2022. **208**: p. 105450.
95. Boras, B., et al., *Preclinical characterization of an intravenous coronavirus 3CL protease inhibitor for the potential treatment of COVID19.* Nature communications, 2021. **12**(1): p. 6055.
96. Wang, L., et al., *Discovery of novel SARS-CoV-2 3CL protease covalent inhibitors using deep learning-based screen.* European journal of medicinal chemistry, 2022. **244**: p. 114803.
97. Zhang, C.-H., et al., *Optimization of triarylpyridinone inhibitors of the main protease of SARS-CoV-2 to low-nanomolar antiviral potency.* ACS medicinal chemistry letters, 2021. **12**(8): p. 1325-1332.
98. Kitamura, N., et al., *Expedited approach toward the rational design of noncovalent SARS-CoV-2 main protease inhibitors.* Journal of medicinal chemistry, 2021. **65**(4): p. 2848-2865.
99. Huff, S., et al., *Discovery and mechanism of SARS-CoV-2 main protease inhibitors.* Journal of medicinal chemistry, 2021. **65**(4): p. 2866-2879.

100. Malla, T.R., et al., *Penicillin derivatives inhibit the SARS-CoV-2 main protease by reaction with its nucleophilic cysteine.* Journal of medicinal chemistry, 2022. **65**(11): p. 7682-7696.
101. Zhang, J.-W., et al., *Discovery of 9, 10-dihydrophenanthrene derivatives as SARS-CoV-2 3CLpro inhibitors for treating COVID-19.* European journal of medicinal chemistry, 2022. **228**: p. 114030.
102. Cannalire, R., et al., *Targeting SARS-CoV-2 proteases and polymerase for COVID-19 treatment: state of the art and future opportunities.* Journal of medicinal chemistry, 2020. **65**(4): p. 2716-2746.
103. Pan, F., et al., *Development of pyrimidone derivatives as nonpeptidic and noncovalent 3-chymotrypsin-like protease (3CLpro) inhibitors with anti-coronavirus activities.* Bioorganic Chemistry, 2025. **154**: p. 107988.
104. Hou, N., et al., *Development of highly potent noncovalent inhibitors of SARS-CoV-2 3CLpro.* ACS central science, 2023. **9**(2): p. 217-227.
105. Ghosh, A.K., et al., *Exploration of P1 and P4 modifications of nirmatrelvir: Design, synthesis, biological evaluation, and X-ray structural studies of SARS-CoV-2 Mpro inhibitors.* Eur J Med Chem, 2024. **267**: p. 116132.
106. Guma, S.D., et al., *Design, synthesis, and biological evaluation of thioamide-linked spiropyrrolidine derivatives as novel 3CLpro inhibitors against SARS-CoV-2.* Bioorganic & Medicinal Chemistry, 2025: p. 118239.
107. Unoh, Y., et al., *Discovery of The Clinical Candidate S-892216: A Second-Generation of SARS-CoV-2 3CL Protease Inhibitor for Treating COVID-19.* bioRxiv, 2025: p. 2025.02. 26.639208.
108. Mao, L., et al., *Olgotrelvir, a dual inhibitor of SARS-CoV-2 Mpro and cathepsin L, as a standalone antiviral oral intervention candidate for COVID-19.* Med, 2024. **5**(1): p. 42-61. e23.
109. Vankadara, S., et al., *A warhead substitution study on the coronavirus main protease inhibitor nirmatrelvir.* ACS medicinal chemistry letters, 2022. **13**(8): p. 1345-1350.
110. Bai, B., et al., *Peptidomimetic nitrile warheads as SARS-CoV-2 3CL protease inhibitors.* RSC medicinal chemistry, 2021. **12**(10): p. 1722-1730.
111. Liu, H., et al., *Development of optimized drug-like small molecule inhibitors of the SARS-CoV-2 3CL protease for treatment of COVID-19.* Nature communications, 2022. **13**(1): p. 1891.
112. Yang, K.S., et al., *A quick route to multiple highly potent SARS-CoV-2 main protease inhibitors.* ChemMedChem, 2021. **16**(6): p. 942-948.
113. Freitas, B.T., et al., *Characterization and noncovalent inhibition of the deubiquitinase and deISGylase activity of SARS-CoV-2 papain-like protease.* ACS infectious diseases, 2020. **6**(8): p. 2099-2109.
114. Fu, Z., et al., *The complex structure of GRL0617 and SARS-CoV-2 PLpro reveals a hot spot for antiviral drug discovery.* Nature communications, 2021. **12**(1): p. 488.

115. Garnsey, M.R., et al., *Discovery of SARS-CoV-2 papain-like protease (PLpro) inhibitors with efficacy in a murine infection model.* Science Advances, 2024. **10**(35): p. eado4288.
116. Swaim, C.D., et al., *6-Thioguanine blocks SARS-CoV-2 replication by inhibition of PLpro.* Iscience, 2021. **24**(10).
117. Ma, C., et al., *Drug-repurposing screening identified tropifexor as a SARS-CoV-2 papain-like protease inhibitor.* ACS infectious diseases, 2022. **8**(5): p. 1022-1030.
118. Zhao, Y., et al., *High-throughput screening identifies established drugs as SARS-CoV-2 PLpro inhibitors.* Protein & cell, 2021. **12**(11): p. 877-888.
119. Srinivasan, V., et al., *Antiviral activity of natural phenolic compounds in complex at an allosteric site of SARS-CoV-2 papain-like protease.* Communications biology, 2022. **5**(1): p. 805.
120. Shan, H., et al., *Development of potent and selective inhibitors targeting the papain-like protease of SARS-CoV-2.* Cell chemical biology, 2021. **28**(6): p. 855-865. e9.
121. Ma, C., et al., *Discovery of SARS-CoV-2 papain-like protease inhibitors through a combination of high-throughput screening and a FlipGFP-based reporter assay.* ACS central science, 2021. **7**(7): p. 1245-1260.
122. Sanders, B.C., et al., *Potent and selective covalent inhibition of the papain-like protease from SARS-CoV-2.* Nature communications, 2023. **14**(1): p. 1733.
123. Shen, Z., et al., *Design of SARS-CoV-2 PLpro inhibitors for COVID-19 antiviral therapy leveraging binding cooperativity.* Journal of medicinal chemistry, 2021. **65**(4): p. 2940-2955.
124. Hersi, F., et al., *Discovery of novel papain-like protease inhibitors for potential treatment of COVID-19.* European Journal of Medicinal Chemistry, 2023. **254**: p. 115380.
125. Napolitano, V., et al., *Acriflavine, a clinically approved drug, inhibits SARS-CoV-2 and other betacoronaviruses.* Cell Chemical Biology, 2022. **29**(5): p. 774-784. e8.
126. Jadhav, P., et al., *Design of quinoline SARS-CoV-2 papain-like protease inhibitors as oral antiviral drug candidates.* Nature Communications, 2025. **16**(1): p. 1604.
127. Zheng, M., et al., *Discovery and characterization of novel potent non-covalent small molecule inhibitors targeting papain-like protease from SARS-CoV-2.* Acta Pharmaceutica Sinica. B, 2024. **14**(7): p. 3286.
128. Wang, J., *Compositions and methods for inhibiting Mpro and PLpro protease activity and for preventing and treating SARS-CoV-2 infection*. 2024, Google Patents.
129. Wang, Q., et al., *Structure-based design of potent peptidomimetic inhibitors covalently targeting SARS-CoV-2 papain-like protease.* International Journal of Molecular Sciences, 2023. **24**(10): p. 8633.
130. Lu, Y., et al., *Discovery of orally bioavailable SARS-CoV-2 papain-like protease inhibitor as a potential treatment for COVID-19.* Nature Communications, 2024. **15**(1): p. 10169.
131. Kuzikov, M., et al., *Thiol-Reactive or Redox-Active: Revising a Repurposing Screen Led to a New Invalidation Pipeline and Identified a True Noncovalent Inhibitor*

Against Papain-like Protease from SARS-CoV-2. ACS Pharmacology & Translational Science, 2024. **8**(1): p. 66-77.
132. Tan, B., et al., *Design of a SARS-CoV-2 papain-like protease inhibitor with antiviral efficacy in a mouse model.* Science, 2024. **383**(6690): p. 1434-1440.
133. Velma, G.R., et al., *Non-Covalent Inhibitors of SARS-CoV-2 Papain-Like Protease (PLpro): In Vitro and In Vivo Antiviral Activity.* Journal of Medicinal Chemistry, 2024. **67**(16): p. 13681-13702.
134. Yuan, S., et al., *Targeting papain-like protease for broad-spectrum coronavirus inhibition.* Protein & Cell, 2022. **13**(12): p. 940-953.
135. Liu, N., et al., *Design and Evaluation of a Novel Peptide–Drug Conjugate Covalently Targeting SARS-CoV-2 Papain-like Protease.* Journal of Medicinal Chemistry, 2022. **65**(1): p. 876-884.
136. Klemm, T., et al., *Mechanism and inhibition of the papain-like protease, PLpro, of SARS-CoV-2.* The EMBO journal, 2020. **39**(18): p. e106275.
137. Kuo, C.-J., et al., *Kinetic characterization and inhibitor screening for the proteases leading to identification of drugs against SARS-CoV-2.* Antimicrobial Agents and chemotherapy, 2021. **65**(4): p. 10.1128/aac. 02577-20.
138. Xu, Y., et al., *Repurposing clinically approved drugs for COVID-19 treatment targeting SARS-CoV-2 papain-like protease.* International journal of biological macromolecules, 2021. **188**: p. 137-146.
139. Chen, X., et al., *Investigating derivatives of tanshinone IIA sulfonate sodium and chloroxine for their inhibition activities against the SARS-CoV-2 papain-like protease.* ACS omega, 2022. **7**(51): p. 48416-48426.
140. Osipiuk, J., et al., *Structure of papain-like protease from SARS-CoV-2 and its complexes with non-covalent inhibitors.* Nature communications, 2021. **12**(1): p. 743.
141. Weglarz-Tomczak, E., et al., *Identification of ebselen and its analogues as potent covalent inhibitors of papain-like protease from SARS-CoV-2.* Scientific reports, 2021. **11**(1): p. 3640.
142. Stasiulewicz, A., et al., *SARS-CoV-2 papain-like protease potential inhibitors—in silico quantitative assessment.* International journal of molecular sciences, 2021. **22**(8): p. 3957.
143. Sousa, B.K., et al., *Discovery of Non-Covalent Inhibitors for SARS-CoV-2 PLpro: Integrating Virtual Screening, Synthesis, and Experimental Validation.* ACS Medicinal Chemistry Letters, 2024. **15**(12): p. 2140-2149.
144. M. Bader, S., et al., *A novel PLpro inhibitor improves outcomes in a pre-clinical model of long COVID.* Nature Communications, 2025. **16**(1): p. 2900.
